# Supplementary material for: Health effects associated with exposure of children to physical violence, psychological violence and neglect: a Burden of Proof study
Source: Nat Hum Behav. 2025 Apr 10;9(6):1217–36. doi: 10.1038/s41562-025-02143-3 (PMC12185353; doi:10.1038/s41562-025-02143-3)
Supplement: Supplementary file 1 — Supplementary methods and results. [file 41562_2025_2143_MOESM1_ESM.pdf]

nature human behaviour

Supplementary information https://doi.org/10.1038/s41562-025-02143-3

Health effects associated with exposure of children to physical violence, psychological violence and neglect: a Burden of Proof study

In the format provided by the authors and unedited

Supplementary Information: Data Sources and Supplementary Results for “Health effects associated with exposure of children to physical violence, psychological violence, and neglect: a Burden of Proof study"

This appendix provides detailed information on input data sources and supplementary results for the publication titled “Health effects associated with exposure of children to physical violence, psychological violence, and neglect: a Burden of Proof study.”

Table of Contents

Section 1: Exposure Definitions....6

Table S1. Definitions of included risk factors. 6

Table S2. Summarized violence exposure definitions for violence against children in included studies. 7

Section 2: Data Inputs 10

Section 2.1: Studies for childhood physical violence, psychological violence, and neglect....10

Table S3. Summary characteristics of studies analyzed for childhood physical abuse, psychological abuse, and neglect 10

Section 3: GBD Outcome Definitions 38

Section 3.1: Standard GBD outcome definitions....38

Table S4. Definitions of included outcomes....38

Section 3.2: Additional description of accepted definitions and measurement tools for depressive and anxiety disorders....40

Table S5. Depressive and anxiety disorder accepted diagnostic interview and symptom scale measurement tools. 40

Section 3.3: Additional description of accepted definitions for substance use disorders....41

Section 4: Sensitivity Analyses 43

Section 4.1: Sensitivity analyses results for childhood violence exposures and outcomes....43

Table S6. Sensitivity analysis results for childhood violence exposure and major depressive disorder 43

Table S7. Sensitivity analysis results for childhood violence exposure and drug use disorders 45

Table S8. Sensitivity analysis results for childhood violence exposure and alcohol use disorders....46

Table S9. Sensitivity analysis results for childhood violence exposure and eating disorders 48

Table S10. Sensitivity analysis results for childhood violence exposure and ischemic heart disease....48

Table S11. Sensitivity analysis results for childhood violence exposure and anxiety disorders 49
Table S12. Sensitivity analysis results for childhood violence exposure and type 2 diabetes 50
Table S13. Sensitivity analysis results for childhood violence exposure and self-harm 52
Table S14. Sensitivity analysis results for childhood violence exposure and asthma 53
Table S15. Sensitivity analysis results for childhood violence exposure and gynecological diseases 53
Table S16. Sensitivity analysis results for childhood violence exposure and maternal abortion and miscarriage 54
Table S17. Sensitivity analysis results for childhood violence exposure and sexually transmitted infections without HIV 54
Table S18. Sensitivity analysis results for childhood violence exposure and schizophrenia 55
Section 4.2: Sensitivity analyses forest plots for childhood physical violence exposures and the corresponding outcomes 56
Figure S1. Sensitivity analysis results for childhood physical violence exposure (females only) and outcomes 56
Figure S2. Sensitivity analysis results for childhood physical violence exposure (males only) and outcomes 57
Figure S3. Sensitivity analysis results for perpetrator-specific childhood physical violence exposure and outcomes 58
Figure S4. Sensitivity analysis results for childhood physical violence exposure by any perpetrator and outcomes 59
Figure S5. Sensitivity analysis results for childhood physical violence exposure and alternative outcome definitions 60
Figure S6. Sensitivity analysis results for childhood physical violence exposure (no adjustment) and outcomes 61
Figure S7. Sensitivity analysis results for childhood physical violence exposure (no trimming) and outcomes 62
Section 4.3: Sensitivity analyses forest plots for psychological childhood violence exposures and corresponding outcomes 63
Figure S8. Sensitivity analysis results for childhood psychological violence exposure and alternative outcome definitions 63
Figure S9. Sensitivity analysis results for childhood psychological violence exposure (females only) and outcomes 64
Figure S10. Sensitivity analysis results for childhood psychological violence exposure (males only) and outcomes 65

Figure S11. Sensitivity analysis results for perpetrator-specific childhood psychological violence exposure and outcomes 66

Figure S12. Sensitivity analysis results for childhood psychological violence exposure by any perpetrator and outcomes 67

Figure S13. Sensitivity analysis results for childhood psychological violence exposure (no adjustment) and outcomes 68

Figure S14. Sensitivity analysis results for childhood psychological violence exposure (no trimming) and outcomes 69

Section 4.4: Sensitivity analyses forest plots for childhood neglect exposures and corresponding outcomes 70

Figure S15. Sensitivity analysis results for childhood neglect (females only) and outcomes 70

Figure S16. Sensitivity analysis results for childhood neglect (males only) and outcomes 71

Figure S17. Sensitivity analysis results for perpetrator-specific childhood neglect and outcomes 72

Figure S18. Sensitivity analysis results for childhood neglect by any perpetrator and outcomes 73

Figure S19. Sensitivity analysis results for childhood neglect and alternative outcome definitions 74

Figure S20. Sensitivity analysis results for childhood neglect (no adjustment) and outcomes 75

Figure S21. Sensitivity analysis results for childhood neglect (no trimming) and outcomes 76

Section 5: Model Characteristics 76

Table S19: Gamma solution for each modeled relationship 77

Table S20: Model characteristics of childhood violence and corresponding health outcomes 77

Section 6: PRISMA and GATHER Checklists 79

Section 6.1: PRISMA 79

Table S21. PRISMA 2020 abstract checklist 79

Table S22. PRISMA 2020 checklist 80

Section 6.2: GATHER 84

Table S23. GATHER checklist 84

Section 7: Data Identification and Assessment 85

Section 7.1: Literature searches 85

PubMed Search String 85

Embase Search String 86
Cumulative Index to Nursing and Allied Health Literature (CINAHL) Search String 87
PsycINFO Search String 88
Global Index Medicus Search String 90
Cochrane Search String 91
Web of Science Core Collection Search String 92
Section 7.2: Inclusion and exclusion criteria 93
Table S24: Inclusion and exclusion criteria applied during screening. 93
Table S25: Inclusion and exclusion criteria applied during data cleaning. 94
Section 7.3: Data extraction 94
Table S26: Data extraction template 94
Section 7.4: Systematic review and meta-analysis citation searching 98
Section 8: Study Quality and Bias Assessment 99
Section 8.1: Definitions of bias covariates 99
Table S27. Standard bias covariates created across all input datasets. 99
Table S28. Adjustment bias covariates created across all input datasets. 99
Table S29. Exposure definition bias covariates. 100
Table S30. Outcome definition bias covariates. 100
Section 8.2: Bias covariates for each risk-outcome pairs 102
Table S31. Bias Covariates for childhood physical abuse and corresponding outcomes. 102
Table S32. Bias Covariates for childhood psychological abuse and corresponding outcomes. 113
Table S33. Bias Covariates for childhood neglect and corresponding outcomes. 118
Section 9: Primary Analysis Funnel Plots 125
Section 9.1: Primary analysis funnel plots for childhood physical violence and outcomes 125
Figure S22. Primary analysis funnel plot for childhood physical violence and maternal abortion and miscarriage 125
Figure S23. Primary analysis funnel plot for childhood physical violence and alcohol use disorders 125
Figure S24. Primary analysis funnel plot for childhood physical violence and anxiety disorders 126
Figure S25. Primary analysis funnel plot for childhood physical violence and asthma 126
Figure S26. Primary analysis funnel plot for childhood physical violence and major depression disorder 127

Figure S27. Primary analysis funnel plot for childhood physical violence and diabetes ... 127
Figure S28. Primary analysis funnel plot for childhood physical violence and drug use disorders ... 128
Figure S29. Primary analysis funnel plot for childhood physical violence and eating disorders ... 128
Figure S30. Primary analysis funnel plot for childhood physical violence and gynecological diseases ... 129
Figure S31. Primary analysis funnel plot for childhood physical violence and ischemic heart disease ... 129
Figure S32. Primary analysis funnel plot for childhood physical violence and migraines .. 130
Figure S33. Primary analysis funnel plot for childhood physical violence and schizophrenia ... 130
Figure S34. Primary analysis funnel plot for childhood physical violence and self-harm .. 131
Figure S35. Primary analysis funnel plot for childhood physical violence and sexually transmitted infections (excluding HIV) ... 131
Figure S36. Primary analysis funnel plot for childhood physical violence and stroke ... 132
Section 9.2: Primary analysis funnel plots for childhood psychological abuse and outcomes ... 132
Figure S37. Primary analysis funnel plot for childhood psychological violence and alcohol use disorder ... 132
Figure S38. Primary analysis funnel plot for childhood psychological violence and anxiety disorder ... 133
Figure S39. Primary analysis funnel plot for childhood psychological violence and asthma ... 133
Figure S40. Primary analysis funnel plot for childhood psychological violence and major depression disorder ... 134
Figure S41. Primary analysis funnel plot for childhood psychological violence and diabetes ... 134
Figure S42. Primary analysis funnel plot for childhood psychological violence and drug use disorders ... 135
Figure S43. Primary analysis funnel plot for childhood psychological violence and gynecological diseases ... 135
Figure S44. Primary analysis funnel plot for childhood psychological violence and ischemic heart disease ... 136
Figure S45. Primary analysis funnel plot for childhood psychological violence and migraines ... 136

Figure S46. Primary analysis funnel plot for childhood psychological violence and schizophrenia 137

Figure S47. Primary analysis funnel plot for childhood psychological violence and self-harm 137

Section 9.3: Primary analysis funnel plots for childhood neglect and outcomes 138

Figure S48. Primary analysis funnel plot for childhood neglect and alcohol use disorders 138

Figure S49. Primary analysis funnel plot for childhood neglect and anxiety disorders 138

Figure S50. Primary analysis funnel plot for childhood neglect and asthma 138

Figure S51. Primary analysis funnel plot for childhood neglect and major depression disorder 139

Figure S52. Primary analysis funnel plot for childhood neglect and diabetes 140

Figure S53. Primary analysis funnel plot for childhood neglect and drug use disorders 140

Figure S54. Primary analysis funnel plot for childhood neglect and schizophrenia 141

Figure S55. Primary analysis funnel plot for childhood neglect and self-harm 141

Figure S56. Primary analysis funnel plot for childhood neglect and sexually transmitted infections (excluding HIV) 142

Section 10: Data for the Primary Analyses 143

Table S34. Data inputs included in the primary analyses 143

Section 1: Exposure Definitions

The definitions listed in Table S1 of childhood physical abuse, childhood psychological abuse, and childhood neglect were adapted using the International Classification of Violence against Children (ICVAC).

Table S1. Definitions of included risk factors.

Risk factor name	Definition
**Childhood Physical Abuse**	Any deliberate, unwanted, and non-essential act that uses physical force against the body of a child aged younger than 18 years old, and that results in or has a high likelihood of resulting in injury, pain, or psychological suffering. It includes, but is not limited to, severe assault and minor assault.
**Childhood Psychological Abuse**	Any deliberate, unwanted, and non-essential act, verbal and non-verbal, that harms or has a high likelihood of harming the development of a child younger than 18 years old, including long-term physiological harm and mental health consequences. It

	includes, but is not limited to, terrorizing, harassing, spurning, humiliating, and controlling a child.
**Childhood Neglect**	The deliberate, unwanted, and non-essential failure to meet a child's physical or psychological needs, protect a child from danger, or obtain medical, educational, or other services when those responsible for the child's care have the means, knowledge, and access to services to do so. It includes, but is not limited to, physical neglect, psychological neglect, neglect of a child's physical or mental health, educational neglect, and abandonment.

Table S2. Summarized violence exposure definitions for violence against children in included studies.

**Physical violence exposure definitions**
Experienced physical abuse (undefined) during childhood
Experienced physical abuse, excluding spanking, during childhood
Experienced non-accidental physical injury inflicted by a caretaker and/or adult during childhood
Experienced being 1) slapped, 2) spanked with an object, 3) pushed, grabbed, or shoved, or 4) kicked, bit, punched, choked, burnt, or otherwise physically attacked during childhood
Experiencing physical punishment during childhood
Experienced being pushed, grabbed, shoved, or hit hard enough to leave bruises or injuries
Experienced being kicked or hit with hands or object
Substantiated physical abuse as reported to central registries
Experienced being slapped, hit, kicked, punched or beaten
Experienced threats of, attempted, or actual physical harm, including being pushed, grabbed, shoved, kicked, bitten, punched, spanked, choked, burned, hit, or otherwise physically attacked
Experienced being pushed, grabbed, shoved, slapped, or having something thrown at them
Experienced being hit by an adult caregiver
Experienced being slapped, spanked, shoved, physically attacked, burned, choked, punched, or having something thrown at them
Experienced being pushed, grabbed, shoved, kicked, bitten, punched, or hit with something
Experienced being kicked, bitten, punched, physically attacked, choked, or burned
Experienced being shot or stabbed

**Psychological violence definition**
Experienced psychological abuse (undefined)
Experienced verbal abuse or threats

Witnessed interparental/domestic violence
Experienced being sworn at, insulted, verbally abused, threatened, or acted in any other way that would incite fear of being hurt or injured
Experienced being yelled at, insulted or threatened
Experienced being verbally abused, subordinated to other children, punishment without reason, or being blackmailed
Experienced emotional trauma, including hostility, coldness, or scapegoating behavior
Witnessed interparental/domestic violence or threats of violence
Felt that they were not wanted or that they were hated
Experienced humiliation, ridicule, or mental cruelty by a parent
Experienced verbal assaults on their sense of worth/well-being or humiliating or demeaning behavior
Experienced being yelled or screamed at
Experienced being yelled, screamed, sworn at, insulted, humiliated, threatened, or abandoned

**Neglect definitions**
Experienced neglect (unspecified)
Experienced physical neglect (unspecified)
Experienced emotional neglect (unspecified)
Experienced substantiated neglect
Experienced being made to do age-inappropriate chore, being unsupervised at a young age, not receiving adequate medical care, or not having basic necessities while their caregiver did
Never experienced feeling like their family wanted them to succeed, thought they were important, believed in them, or were supportive
Experiencing physical neglect (being made to do age-inappropriate chores, being unsupervised at a young age, not having clothing or school supplies because money was spent on the adults, were made to go hungry, or failed to receive medical care) or emotional neglect (never feeling like their family wanted them to succeed, thought they were special/important, believed in them, or could support them)
Experienced a failure to provide conditions essential for health physical and emotional development
Did not have enough food at home or had to wear dirty/torn clothes due to lack of options
Was left at home alone when an adult should have been present, or their basic needs were not met

Experienced parental disinterest in material care, health, schoolwork, and friendships
Did not have enough food or caregivers were too drunk/intoxicated to appropriately care for the child

Section 2: Data Inputs

Section 2.1: Studies for childhood physical violence, psychological violence, and neglect

Table S3. Summary characteristics of studies analyzed for childhood physical abuse, psychological abuse, and neglect

Author	Year	Location	Study Design	Type of Violence	Exposure Assessment Method	Exposure Recall	Type of Perpetrator	Follow-up Years	Outcome	Type of Outcome	Outcome Assessment Method	Age Summary	Gender	Sample Size	Number of Cases (Exposed Group)	Number of Cases (Unexposed Group)
Broekhof	2023	Trondelag	Prospective Cohort	Emotional Neglect	Self-Report or Self-Administered Survey	Lifetime	Family Member or Caregiver		Alcohol Use Disorder	Incidence	administrative medical records or disease registries	15.91 (0.03)	Women	4129	6	17
Laflair	2013	USA	Prospective Cohort	Neglect	Self-Report or Self-Administered Survey	Lifetime	Family Member or Caregiver	4	Hazardous Alcohol Involvement (Abuse or Dependence)	Incidence	self-report	18-99	Women	11750		
Fenton	2013	USA	Prospective Cohort	Physical Neglect	Self-Report or Self-Administered Survey	Lifetime	Family Member or Caregiver	3	Alcohol Dependence	Incidence	self-report	18–99	Combined Men/Women	27712	1105	5951
Fenton	2013	USA	Prospective Cohort	Emotional Neglect	Self-Report or Self-Administered Survey	Lifetime	Family Member or Caregiver	3	Alcohol Dependence	Incidence	self-report	18–99	Combined Men/Women	27712	454	4277
Kascakova	2022	Czechia	Retrospective Cohort	Psychological Neglect	Self-Report or Self-Administered Survey	Lifetime	Anyone or Not-Specified		Alcohol Use Disorder	Incident	self-report	46.4 (17.4)	Combined Men/Women	1800		
Kascakova	2022	Czechia	Retrospective Cohort	Physical Neglect	Self-Report or Self-Administered Survey	Lifetime	Anyone or Not-Specified		Alcohol Use Disorder	Incident	self-report	46.4 (17.4)	Combined Men/Women	1800		
Kascakova	2022	Czechia	Retrospective Cohort	Psychological Neglect	Self-Report or Self-Administered Survey	Lifetime	Anyone or Not-Specified		Alcohol Use Disorder	Incident	self-report	46.2 (16.6)	Combined Men/Women	1018		
Kascakova	2022	Czechia	Retrospective Cohort	Physical Neglect	Self-Report or Self-Administered Survey	Lifetime	Anyone or Not-Specified		Alcohol Use Disorder	Incident	self-report	46.2 (16.6)	Combined Men/Women	1018		
Kisely	2020	Australia	Prospective Cohort	Neglect	Routinely Collected/Administrative Data	Lifetime	Anyone or Not-Specified	21	Heavy Alcohol Use In The Last Month	Incidence	self-report	21–21	Combined Men/Women	3762	10	
Tenhave	2019	Netherlands	Prospective Cohort	Neglect	Self-Report or Self-Administered Survey	Lifetime	Anyone or Not-Specified	3	Anxiety Disorders	Incidence	self-report	18–64	Combined Men/Women	9304		
Widom	1999	USA	Prospective Cohort	Neglect	Routinely Collected/Administrative Data	Lifetime	Family Member or Caregiver	20	Current Ptsd	Incidence	self-report	28.72 (3.84)	Combined Men/Women	1063	94	54

Author	Year	Location	Study Design	Type of Violence	Exposure Assessment Method	Exposure Recall	Type of Perpetrator	Follow-up Years	Outcome	Type of Outcome	Outcome Assessment Method	Age Summary	Gender	Sample Size	Number of Cases (Exposed Group)	Number of Cases (Unexposed Group)
Fujiwara	2011	Japan	Case-Control	Neglect	Self-Report or Self-Administered Survey	Lifetime	Anyone or Not Specified		Anxiety Disorder	Mortality	self-report	50.8 (0.6)	Combined Men/Women			
Young	2011	Scotland	Prospective Cohort	Neglect	Self-Report or Self-Administered Survey	Lifetime	Family Member or Caregiver	4	Anxiety Disorder	Incidence	self-report	15–15	Combined Men/Women			
Raposo	2014	USA	Prospective Cohort	Neglect	Self-Report or Self-Administered Survey	Lifetime	Family Member or Caregiver	4	Anxiety Disorder	Incidence	self-report	65–99	Combined Men/Women	7177		
Danese	2023	USA	Prospective Cohort	Neglect	Self-Report or Self-Administered Survey ; Routinely Collected/Administrative Data	Lifetime	Family Member or Caregiver	40	Anxiety Disorder	Incidence	self-report	41.2 (3.5)	Combined Men/Women	435		
Danese	2023	USA	Prospective Cohort	Neglect	Self-Report or Self-Administered Survey ; Routinely Collected/Administrative Data	Lifetime	Family Member or Caregiver	40	Anxiety Disorder	Incidence	self-report	41.2 (3.5)	Combined Men/Women	754		
Danese	2023	USA	Prospective Cohort	Neglect	Self-Report or Self-Administered Survey ; Routinely Collected/Administrative Data	Lifetime	Family Member or Caregiver	40	Anxiety Disorder	Incidence	self-report	41.2 (3.5)	Combined Men/Women	514		
Elbarazi	2023	Egypt	Prospective Cohort	Emotional Neglect	Self-Report or Self-Administered Survey	Lifetime	Anyone or Not-Specified	1	Anxiety	Incidence	self-report	19.03 (0.46)	Combined Men/Women	319		
Elbarazi	2023	Egypt	Prospective Cohort	Physical Neglect	Self-Report or Self-Administered Survey	Lifetime	Anyone or Not-Specified	1	Anxiety	Incidence	self-report	19.03 (0.46)	Combined Men/Women	319		
Hovens	2015	Netherlands	Retrospective Cohort	Neglect	Self-Report or Self-Administered Survey	Lifetime	Caregiver	2	Anxiety Disorder	Incidence	self-report	42.6 (13.9)	Combined Men/Women	1007	14	
Hovens	2015	Netherlands	Retrospective Cohort	Neglect	Self-Report or Self-Administered Survey	Lifetime	Caregiver	2	Anxiety Disorder	Incidence	self-report	42.6 (13.9)	Combined Men/Women	1007	10	
Kisely	2020	Australia	Prospective Cohort	Neglect	Routinely Collected/Administrative Data	Lifetime	Anyone or Not-Specified	30	Any Anxiety Disorder (Last 12 Months)	Incidence	self-report	30–30	Combined Men/Women	2861		
Kisely	2020	Australia	Prospective Cohort	Neglect	Routinely Collected/Administrative Data	Lifetime	Anyone or Not-Specified	30	Any Post-Traumatic Stress Disorder (Last 12 Months)	Incidence	self-report	30–30	Combined Men/Women	2861		
Abajobir	2017	Australia	Prospective Cohort	Neglect	Routinely Collected/Administrative Data	Lifetime	Family Member or Caregiver	21	Asthma	Incidence	self-report; physician diagnosis	21–21	Combined Men/Women	3762		
Kascakova	2022	Czechia	Retrospective Cohort	Psychological Neglect	Self-Report or Self-Administered Survey	Lifetime	Anyone or Not-Specified		Asthma	Incidence	self-report	46.2 (16.6)	Combined Men/Women	1018		

Author	Year	Location	Study Design	Type of Violence	Exposure Assessment Method	Exposure Recall	Type of Perpetrator	Follow-up Years	Outcome	Type of Outcome	Outcome Assessment Method	Age Summary	Gender	Sample Size	Number of Cases (Exposed Group)	Number of Cases (Unexposed Group)
Kascakova	2022	Czechia	Retrospective Cohort	Physical Neglect	Self-Report or Self-Administered Survey	Lifetime	Anyone or Not-Specified		Asthma	Incidence	self-report	46.2 (16.6)	Combined Men/Women	1018		
Kascakova	2022	Czechia	Retrospective Cohort	Psychological Neglect	Self-Report or Self-Administered Survey	Lifetime	Anyone or Not-Specified		Asthma	Incidence	self-report	46.4 (17.4)	Combined Men/Women	1800		
Kascakova	2022	Czechia	Retrospective Cohort	Physical Neglect	Self-Report or Self-Administered Survey	Lifetime	Anyone or Not-Specified		Asthma	Incidence	self-report	46.4 (17.4)	Combined Men/Women	1800		
Han	2022	Multiple	Prospective Cohort	Physical Neglect	Self-Report or Self-Administered Survey	Lifetime	Anyone or Not-Specified	11	Asthma	Incidence	self-report; physician diagnosis	54.2 (7.9)	Combined Men/Women	81105		
Han	2022	Multiple	Prospective Cohort	Emotional Neglect	Self-Report or Self-Administered Survey	Lifetime	Anyone or Not-Specified	11	Asthma	Incidence	self-report; physician diagnosis	54.2 (7.9)	Combined Men/Women	81105		
Zhang	2023	China	Prospective Cohort	Emotional Neglect	Self-Report or Self-Administered Survey	Lifetime	Anyone or Not-Specified	4	Depression	Incidence	self-report	60.7 (9.5)	Combined Men/Women	14484		
Widom	2023	USA	Case-Control	Neglect	Routinely Collected/Administrative Data		Family Member or Caregiver		Depression	Mortality	self-report; biomarker	41.2	Combined Men/Women	675	86	49
Widom	2007	USA	Prospective Cohort	Neglect	Routinely Collected/Administrative Data	Lifetime	Family Member or Caregiver		Current Major Depressive Disorder (Mdd) Diagnosis		self-report; interview	28.7 (3.8)	Combined Men/Women	1737	86	
Brown	1999	New York	Prospective Cohort	Neglect	Self-Report or Self-Administered Survey ; Routinely Collected/Administrative Data	Lifetime	Caregiver	17	Adult Depressive Disorders	Incidence	self-report	18–99	Combined Men/Women	597	5	28
Comijs	2013	Netherlands	Case-Control	Emotional Neglect	Self-Report or Self-Administered Survey	Lifetime	Family Member or Caregiver		Depressive Disorders	Mortality	self-report	70.5	Combined Men/Women	508	156	
Young	2011	Scotland	Prospective Cohort	Neglect	Self-Report or Self-Administered Survey	Lifetime	Family Member or Caregiver	4	Depressive Disorder	Incidence	self-report	15–15	Combined Men/Women			
Kisely	2020	Australia	Prospective Cohort	Neglect	Routinely Collected/Administrative Data	Lifetime	Anyone or Not-Specified	30	Any Depressive Disorder (Last 12 Months)	Incidence	self-report	30–30	Combined Men/Women	2861		
Lemasters	2021	Pakistan	Prospective Cohort	Neglect (Physical or Emotional)	Self-Report or Self-Administered Survey	Lifetime	Anyone or Not-Specified	3	Major Depressive Episodes (Mde)	Incidence	self-report	26.7	Women	1072		124
Ebert	2019	Belgium	Prospective Cohort	Neglect	Self-Report or Self-Administered Survey	Lifetime	Anyone or Not-Specified	1	Major Depressive Disorder	Incidence	self-report	19	Combined Men/Women	2242		
Xiao	2022	Yunnan	Case-Control	Physical Neglect	Self-Report or Self-Administered Survey	Lifetime	Anyone or Not-Specified		Any Depressive Disorder	Mortality	physician diagnosis	13.47 (1.71)	Combined Men/Women	1134	267	

Author	Year	Location	Study Design	Type of Violence	Exposure Assessment Method	Exposure Recall	Type of Perpetrator	Follow-up Years	Outcome	Type of Outcome	Outcome Assessment Method	Age Summary	Gender	Sample Size	Number of Cases (Exposed Group)	Number of Cases (Unexposed Group)
Xiao	2022	Yunnan	Case-Control	Emotional Neglect	Self-Report or Self-Administered Survey	Lifetime	Anyone or Not Specified		Any Depressive Disorder	Mortality	physician diagnosis	13.47 (1.71)	Combined Men/Women	1134	321	
Danese	2023	USA	Prospective Cohort	Neglect	Self-Report or Self-Administered Survey ; Routinely Collected/Administrative Data	Lifetime	Family Member or Caregiver	40	Depression	Incidence	self-report	41.2 (3.5)	Combined Men/Women	435		
Danese	2023	USA	Prospective Cohort	Neglect	Self-Report or Self-Administered Survey ; Routinely Collected/Administrative Data	Lifetime	Family or Caregiver	40	Depression	Incidence	self-report	41.2 (3.5)	Combined Men/Women	754		
Danese	2023	USA	Prospective Cohort	Neglect	Self-Report or Self-Administered Survey ; Routinely Collected/Administrative Data	Lifetime	Family Member or Caregiver	40	Depression	Incidence	self-report	41.2 (3.5)	Combined Men/Women	514		
Kang	2023	Republic Of Korea	Case-Control	Emotional Neglect	Self-Report or Self-Administered Survey	Lifetime	Anyone or Not-Specified		Depression	Mortality	physician diagnosis	19–65	Combined Men/Women	100	41	3
Kang	2023	Republic Of Korea	Case-Control	Physical Neglect	Self-Report or Self-Administered Survey	Lifetime	Anyone or Not-Specified		Depression	Mortality	physician diagnosis	19–65	Combined Men/Women	126	46	3
Zhou	2023	Beijing	Case-Control	Emotional Neglect	Self-Report or Self-Administered Survey	Lifetime	Anyone or Not-Specified		Major Depressive Disorder	Mortality	administrative medical records or disease registries; physician diagnosis	30.5 (9.29)	Men	210	41	64
Zhou	2023	Beijing	Case-Control	Physical Neglect	Self-Report or Self-Administered Survey	Lifetime	Anyone or Not-Specified		Major Depressive Disorder	Mortality	administrative medical records or disease registries; physician diagnosis	30.5 (9.29)	Men	210	33	72
Zhou	2023	Beijing	Case-Control	Emotional Neglect	Self-Report or Self-Administered Survey	Lifetime	Anyone or Not-Specified		Major Depressive Disorder	Mortality	administrative medical records or disease registries; physician diagnosis	30.5 (9.29)	Women	370	80	105
Zhou	2023	Beijing	Case-Control	Physical Neglect	Self-Report or Self-Administered Survey	Lifetime	Anyone or Not-Specified		Major Depressive Disorder	Mortality	administrative medical records or disease registries;	30.5 (9.29)	Women	370	50	135

Author	Year	Location	Study Design	Type of Violence	Exposure Assessment Method	Exposure Recall	Type of Perpetrator	Follow-up Years	Outcome	Type of Outcome	Outcome Assessment Method	Age Summary	Gender	Sample Size	Number of Cases (Exposed Group)	Number of Cases (Unexposed Group)
											physician diagnosis					
Elbarazi	2023	Egypt	Prospective Cohort	Emotional Neglect	Self-Report or Self-Administered Survey	Lifetime	Anyone or Not-Specified	1	Depression	Incidence	self-report	19.03 (0.46)	Combined Men/Women	319		
Elbarazi	2023	Egypt	Prospective Cohort	Physical Neglect	Self-Report or Self-Administered Survey	Lifetime	Anyone or Not-Specified	1	Depression	Incidence	self-report	19.03 (0.46)	Combined Men/Women	319		
Hovens	2015	Netherlands	Retrospective Cohort	Neglect	Self-Report or Self-Administered Survey	Lifetime	Caregiver	2	Depressive Disorder	Incidence	self-report	42.6 (13.9)	Combined Men/Women	1038	18	
Hovens	2015	Netherlands	Retrospective Cohort	Neglect	Self-Report or Self-Administered Survey	Lifetime	Caregiver	2	Depressive Disorder	Incidence	self-report	42.6 (13.9)	Combined Men/Women	1038	20	
Galloeag	2017	Rio Grande Do Sul	Prospective Cohort	Neglect	Self-Report or Self-Administered Survey	Lifetime	Anyone or Not-Specified	18	Major Depression	Incidence	self-report	18	Women	1954	18	176
Galloeag	2017	Rio Grande Do Sul	Prospective Cohort	Neglect	Self-Report or Self-Administered Survey	Lifetime	Anyone or Not-Specified	18	Major Depression	Incidence	self-report	18	Men	1761	6	51
Houtepen	2020	Bristol, City Of	Prospective Cohort	Emotional Neglect	Self-Report or Self-Administered Survey ; Parental Report	Lifetime	Family Member or Caregiver	18	Depression	Incidence	self-report	22–22	Combined Men/Women	9959		
Widom	2023	USA	Case-Control	Neglect	Routinely Collected/Administrative Data		Family Member or Caregiver		Diabetes	Mortality	self-report; biomarker	41.2	Combined Men/Women	675	55	43
Kascakova	2022	Czechia	Retrospective Cohort	Psychological Neglect	Self-Report or Self-Administered Survey	Lifetime	Anyone or Not-Specified		Diabetes Mellitus	Incident	self-report	46.2 (16.6)	Combined Men/Women	1018		
Kascakova	2022	Czechia	Retrospective Cohort	Physical Neglect	Self-Report or Self-Administered Survey	Lifetime	Anyone or Not-Specified		Diabetes Mellitus	Incident	self-report	46.2 (16.6)	Combined Men/Women	1018		
Kascakova	2022	Czechia	Retrospective Cohort	Psychological Neglect	Self-Report or Self-Administered Survey	Lifetime	Anyone or Not-Specified		Diabetes Mellitus	Incident	self-report	46.4 (17.4)	Combined Men/Women	1800		
Kascakova	2022	Czechia	Retrospective Cohort	Physical Neglect	Self-Report or Self-Administered Survey	Lifetime	Anyone or Not-Specified		Diabetes Mellitus	Incident	self-report	46.4 (17.4)	Combined Men/Women	1800		
Sanderson	2023	USA	Prospective Cohort	Physical Neglect, Emotional Neglect	Self-Report or Self-Administered Survey	Lifetime	Anyone or Not-Specified	7.7	Type 2 Diabetes	Incidence	self-report; physician diagnosis	40–79	Women	11420	183	2019
Sanderson	2023	USA	Prospective Cohort	Physical Neglect, Emotional Neglect	Self-Report or Self-Administered Survey	Lifetime	Anyone or Not-Specified	7.7	Type 2 Diabetes	Incidence	self-report; physician diagnosis	40–79	Men	6905	86	1088
Zhu	2023	China	Prospective Cohort	Emotional Neglect	Self-Report or Self-Administered Survey	Lifetime	Anyone or Not-Specified	7	Diabetes	Incidence	self-report; physician diagnosis; biomarker	45–99	Men	4409		
Zhu	2023	China	Prospective Cohort	Emotional Neglect	Self-Report or Self-Administered Survey	Lifetime	Anyone or Not-Specified	7	Diabetes	Incidence	self-report; physician	45–99	Women	4770		

Author	Year	Location	Study Design	Type of Violence	Exposure Assessment Method	Exposure Recall	Type of Perpetrator	Follow-up Years	Outcome	Type of Outcome	Outcome Assessment Method	Age Summary	Gender	Sample Size	Number of Cases (Exposed Group)	Number of Cases (Unexposed Group)
Thomas	2008	Multiple	Prospective Cohort	Physical Neglect	Self-Report or Self-Administered Survey	Lifetime	Anyone or Not-Specified	38	Type 2 Diabetes Mellitus or Hba1c >= 6	Incidence	diagnosis; biomarker physician diagnosis; biomarker	45–45	Combined Men/Women	9310		
Duncan	2015	USA	Prospective Cohort	Neglect	Self-Report or Self-Administered Survey	Lifetime	Family Member or Caregiver	15	Diabetes	Incidence	self-report; physician diagnosis; biomarker	24–34	Women		116	329
Duncan	2015	USA	Prospective Cohort	Neglect	Self-Report or Self-Administered Survey	Lifetime	Family Member or Caregiver	15	Diabetes	Incidence	self-report; physician diagnosis; biomarker	24–34	Women		91	329
Duncan	2015	USA	Prospective Cohort	Neglect	Self-Report or Self-Administered Survey	Lifetime	Family Member or Caregiver	15	Diabetes	Incidence	self-report; physician diagnosis; biomarker	24–34	Men		72	231
Duncan	2015	USA	Prospective Cohort	Neglect	Self-Report or Self-Administered Survey	Lifetime	Family Member or Caregiver	15	Diabetes	Incidence	self-report; physician diagnosis; biomarker	24–34	Men		83	231
Harrington	2011	USA	Prospective Cohort	Neglect	Self-Report or Self-Administered Survey	Lifetime	Parent or Guardian	3	Drug Use	Incidence	self-report	18–99	Combined Men/Women	26935	58	
Schwartz	2024	France	Prospective Cohort	Emotional Neglect	Self-Report or Self-Administered Survey	Lifetime	Family Member or Caregiver	1	Illegal Drug Use	Incidence	self-report	18–30	Combined Men/Women	1052		
Schwartz	2024	France	Prospective Cohort	Physical Neglect	Self-Report or Self-Administered Survey	Lifetime	Family Member or Caregiver	1	Illegal Drug Use	Incidence	self-report	18–30	Combined Men/Women	1052		
Huang	2011	USA	Prospective Cohort	Neglect	Self-Report or Self-Administered Survey	Lifetime	Family Member or Caregiver	7	Illicit Drug Use In The Past Year	Incidence	self-report	21.8 (0.12)	Combined Men/Women	4882	364	
Conroy	2009	Australia	Case-Control	Neglect	Self-Report or Self-Administered Survey	Lifetime	Anyone or Not-Specified		Opioid Dependency	Mortality	enrollment in a pharmacotherapy program as clinical determination of opioid dependence	35	Women	571	294	85
Conroy	2009	Australia	Case-Control	Neglect	Self-Report or Self-Administered Survey	Lifetime	Anyone or Not-Specified		Opioid Dependency	Mortality	enrollment in a pharmacotherapy program as clinical	35	Men	742	457	131

Author	Year	Location	Study Design	Type of Violence	Exposure Assessment Method	Exposure Recall	Type of Perpetrator	Follow-up Years	Outcome	Type of Outcome	Outcome Assessment Method	Age Summary	Gender	Sample Size	Number of Cases (Exposed Group)	Number of Cases (Unexposed Group)
											determination of opioid dependence					
Abajobir	2017	Australia	Prospective Cohort	Neglect	Routinely Collected/Administrative Data	Lifetime	Caregiver	21	Injecting Drug Use	Incidence	self-report	20.6	Men			118
Abajobir	2017	Australia	Prospective Cohort	Neglect	Routinely Collected/Administrative Data	Lifetime	Caregiver	21	Injecting Drug Use	Incidence	self-report	20.6	Women			91
Mall	2020	South Africa	Case-Control	Neglect	Self-Report or Self-Administered Survey	Lifetime	Caregiver		Schizophrenia	Mortality	physician diagnosis	36.1 (9.13)	Combined Men/Women	2097	733	
Abajobir	2017	Australia	Prospective Cohort	Neglect	Routinely Collected/Administrative Data	Lifetime	Anyone or Not-Specified	21	Any Dsm-Iv Type Psychosis (Past 12 Months)	Incidence	self-report	20.6	Combined Men/Women	2558		
Chatziioannidis	2019	Greece	Case-Control	Neglect	Self-Report or Self-Administered Survey	Lifetime	Father		Schizophrenia And Other Psychotic Disorders	Mortality	physician diagnosis	40 (10)	Combined Men/Women	124	11	
Alkema	2023	Netherlands	Case-Control	Emotional Neglect	Self-Report or Self-Administered Survey	Lifetime	Anyone or Not-Specified		Schizophrenia	Mortality	physician diagnosis	18–99	Combined Men/Women	4037		
Alkema	2023	Netherlands	Case-Control	Physical Neglect	Self-Report or Self-Administered Survey	Lifetime	Anyone or Not-Specified		Schizophrenia	Mortality	physician diagnosis	18–99	Combined Men/Women	4037		
Kiselydmedres	2022	Australia	Prospective Cohort	Neglect	Self-Report or Self-Administered Survey ; Routinely Collected/Administrative Data	Lifetime	Anyone or Not-Specified	30	Any Self-Harm	Incidence	self-report	30–30	Combined Men/Women			
Geng	2023	Shandong	Case-Control	Emotional Neglect	Self-Report or Self-Administered Survey	Lifetime	Anyone or Not-Specified		Self-Injury	Mortality	administrative medical records or disease registries; physician diagnosis	12–20	Combined Men/Women	414	82	56
Brown	1999	New York	Prospective Cohort	Neglect	Self-Report or Self-Administered Survey ; Routinely Collected/Administrative Data	Lifetime	Caregiver	17	Adult Suicide Attempt	Incidence	self-report	18–99	Combined Men/Women	597	2	13
Enns	2006	Netherlands	Prospective Cohort	Emotional Neglect	Self-Report or Self-Administered Survey	Lifetime	Anyone or Not-Specified	3	Suicidal Attempts	Incidence	self-report	18–64	Combined Men/Women	5670		
Thompson	2019	USA	Prospective Cohort	Neglect	Self-Report or Self-Administered Survey	Lifetime	Parent or Adult Caregiver	13	Suicide Attempt	Incidence	self-report	15.03 (0.11)	Combined Men/Women	9421		

Author	Year	Location	Study Design	Type of Violence	Exposure Assessment Method	Exposure Recall	Type of Perpetrator	Follow-up Years	Outcome	Type of Outcome	Outcome Assessment Method	Age Summary	Gender	Sample Size	Number of Cases (Exposed Group)	Number of Cases (Unexposed Group)
Bruffaerts	2010	Multiple	Retrospective Cohort	Neglect	Self-Report or Self-Administered Survey	Lifetime	Anyone or Not-Specified		Suicide Attempt	Incidence	self-report	20–29	Combined Men/Women	109377		
Bruffaerts	2010	Multiple	Retrospective Cohort	Neglect	Self-Report or Self-Administered Survey	Lifetime	Anyone or Not-Specified		Suicide Attempt	Incidence	self-report	30–99	Combined Men/Women	109377		
Rajapakse	2020	Sri Lanka	Case-Control	Physical Neglect	Self-Report or Self-Administered Survey	Lifetime	Anyone or Not-Specified		Self-Poisoning	Mortality	self-report; administrative medical records or disease registries	25	Women	403	10	
London	2017	USA	Prospective Cohort	Neglect	Self-Report or Self-Administered Survey	Lifetime	Family Member or Caregiver	14	Stis (Sexually Transmitted Infections)	Incidence	self-report; physician diagnosis	24–32	Combined Men/Women	12288	53	
Wilson	2009	USA	Prospective Cohort	Neglect	Routinely Collected/Administrative Data	Lifetime	Anyone or Not-Specified; Partner		Chlamydia	Incidence	self-report	41.2	Combined Men/Women	670	34	30
Wilson	2009	USA	Prospective Cohort	Neglect	Routinely Collected/Administrative Data	Lifetime	Anyone or Not-Specified		Genital Herpes	Incidence	self-report	41.2	Combined Men/Women	670	12	8
Wilson	2009	USA	Prospective Cohort	Neglect	Routinely Collected/Administrative Data	Lifetime	Anyone or Not-Specified		Gonorrhea	Incidence	self-report	41.2		670	42	32
Wilson	2009	USA	Prospective Cohort	Neglect	Routinely Collected/Administrative Data	Lifetime	Anyone or Not Specified		Syphilis	Incidence	self-report	41.2	Combined Men/Women			
Haydon	2011	USA	Prospective Cohort	Supervision Neglect	Self-Report or Self-Administered Survey	Lifetime	Parent or Adult Caregiver	14	Test-Identified Current Std	Incidence	self-report; biomarker	15.9	Men	3967		
Haydon	2011	USA	Prospective Cohort	Physical Neglect	Self-Report or Self-Administered Survey	Lifetime	Parent or Adult Caregiver	14	Test-Identified Current Std	Incidence	self-report; biomarker	15.9	Men	3967		
Haydon	2011	USA	Prospective Cohort	Supervision Neglect	Self-Report or Self-Administered Survey	Lifetime	Parent or Adult Caregiver	14	Test-Identified Current Std	Incidence	self-report; biomarker	15.9	Women	4955		
Haydon	2011	USA	Prospective Cohort	Physical Neglect	Self-Report or Self-Administered Survey	Lifetime	Parent or Adult Caregiver	14	Test-Identified Current Std	Incidence	self-report; biomarker	15.9	Women	4955		
Kerkar	2021	Louisiana	Retrospective Cohort	Physical	Self-Report or Self-Administered Survey	Lifetime	Anyone or Not Specified		Miscarriage At Any Pregnancy	Incidence	self-report	18–45	Women	1050		
Kerkar	2021	Louisiana	Retrospective Cohort	Physical	Self-Report or Self-Administered Survey	Lifetime	Anyone or Not-Specified		Miscarriage At Any Pregnancy	Incidence	self-report	18–45	Women	1050		

Author	Year	Location	Study Design	Type of Violence	Exposure Assessment Method	Exposure Recall	Type of Perpetrator	Follow-up Years	Outcome	Type of Outcome	Outcome Assessment Method	Age Summary	Gender	Sample Size	Number of Cases (Exposed Group)	Number of Cases (Unexposed Group)
Abajobir	2018	Australia	Prospective Cohort	Physical	Routinely Collected/Administrative Data	Lifetime	Family Member or Caregiver	21	Pregnancy Miscarriage	Incidence	self-report	20.6	Women	1544	10	126
Abajobir	2018	Australia	Prospective Cohort	Physical	Routinely Collected/Administrative Data	Lifetime	Family Member or Caregiver	21	Termination Of Pregnancy	Incidence	self-report	20.6	Women	1542	7	157
Demakakos	2020	England	Retrospective Cohort	Physical	Self-Report or Self-Administered Survey	Lifetime	Anyone or Not-Specified		Recurrent Miscarriage	Incidence	self-report	55–89	Women	2795		
Demakakos	2020	England	Retrospective Cohort	Physical	Self-Report or Self-Administered Survey	Lifetime	Anyone or Not-Specified		Single Miscarriage	Incidence	self-report	55–89	Women	2795		
Fenton	2013	USA	Prospective Cohort	Physical	Self-Report or Self-Administered Survey	Lifetime	Family Member or Caregiver	3	Alcohol Dependence	Incidence	self-report	18–99	Combined Men/Women	27712	1208	3556
Roustit	2009	France	Retrospective Cohort	Physical	Self-Report or Self-Administered Survey	Lifetime	Anyone or Not Specified		Alcohol Dependence	Incidence	self-report	18–99	Combined Men/Women	3023		
Kascakova	2022	Czechia	Retrospective Cohort	Physical	Self-Report or Self-Administered Survey	Lifetime	Anyone or Not-Specified		Alcohol Use Disorder	Incident	self-report	46.4 (17.4)	Combined Men/Women	1800		
Kascakova	2022	Czechia	Retrospective Cohort	Physical	Self-Report or Self-Administered Survey	Lifetime	Anyone or Not-Specified		Alcohol Use Disorder	Incident	self-report	46.2 (16.6)	Combined Men/Women	1018		
Najman	2022	Australia	Prospective Cohort	Physical	Self-Report or Self-Administered Survey	Lifetime	Anyone or Not-Specified	30	Alcohol Use Disorder	Incidence	self-report	30–30	Combined Men/Women	2474		
Telfar	2023	New Zealand	Prospective Cohort	Physical	Self-Report or Self-Administered Survey	Lifetime	Family Member or Caregiver	40	Alcohol Abuse/Dependence	Incidence	self-report		Combined Men/Women			
Telfar	2023	New Zealand	Prospective Cohort	Physical	Self-Report or Self-Administered Survey	Lifetime	Family Member or Caregiver	40	Alcohol Abuse/Dependence	Incidence	self-report		Combined Men/Women			
Broekhof	2023	Trondelag	Prospective Cohort	Physical	Self-Report or Self-Administered Survey	Lifetime	Anyone or Not-Specified		Alcohol Use Disorder	Incidence	administrative medical records or disease registries	15.91 (0.03)	Women	4129	9	14
Broekhof	2023	Trondelag	Prospective Cohort	Physical	Self-Report or Self-Administered Survey	Lifetime	Anyone or Not-Specified		Alcohol Use Disorder	Incidence	administrative medical records or disease registries	15.85 (0.03)	Men	4070	5	22
Libby	2004	USA	Prospective Cohort	Physical	Self-Report or Self-Administered Survey	Lifetime	Family Member or Caregiver; Acquaintance		Alcohol Dependence	Incidence	self-report	34.29 (0.08)	Combined Men/Women			
Libby	2004	USA	Prospective Cohort	Physical	Self-Report or Self-Administered Survey	Lifetime	Family Member or Caregiver; Acquaintance		Alcohol Dependence	Incidence	self-report	33.76 (0.08)	Combined Men/Women			

Author	Year	Location	Study Design	Type of Violence	Exposure Assessment Method	Exposure Recall	Type of Perpetrator	Follow-up Years	Outcome	Type of Outcome	Outcome Assessment Method	Age Summary	Gender	Sample Size	Number of Cases (Exposed Group)	Number of Cases (Unexposed Group)
Tanaka	2015	Canada	Prospective Cohort	Physical	Self-Report or Self-Administered Survey	Lifetime	Adult	18	Alcohol Problem	Incidence	self-report	21–35	Men	924		
Tanaka	2015	Canada	Prospective Cohort	Physical	Self-Report or Self-Administered Survey	Lifetime	Adult	18	Alcohol Problem	Incidence	self-report	21–35	Women	969		
Tenhave	2019	Netherlands	Prospective Cohort	Physical	Self-Report or Self-Administered Survey	Lifetime	Anyone or Not Specified	3	Anxiety Disorders	Incidence	self-report	18–64	Combined Men/Women	9304		
Bhattarai	2023	Canada	Prospective Cohort	Physical	Self-Report or Self-Administered Survey	Lifetime	Family Member or Caregiver	2	Anxiety	Incidence	self-report	18.1 (1.6)	Combined Men/Women			
Yu	2023	United Kingdom	Prospective Cohort	Physical	Self-Report or Self-Administered Survey	Lifetime	Family Member or Caregiver	16	Anxiety Disorder	Incidence	self-report; administrative medical records or disease registries	55.852 (7.77)	Combined Men/Women	126064		
Elbarazi	2023	Egypt	Prospective Cohort	Physical	Self-Report or Self-Administered Survey	Lifetime	Anyone or Not-Specified	1	Anxiety	Incidence	self-report	19.03 (0.46)	Combined Men/Women	319		
Fergusson	2008	New Zealand	Prospective Cohort	Physical	Self-Report or Self-Administered Survey	Lifetime	Family Member or Caregiver	25	Anxiety	Incidence	self-report	16–25	Combined Men/Women	1001		
Widom	1999	USA	Prospective Cohort	Physical	Routinely Collected/Administrative Data	Lifetime	Anyone or Not-Specified	20	Current Ptsd	Incidence	self-report	28.72 (3.84)	Combined Men/Women	1196	21	54
Fujiwara	2011	Japan	Case-Control	Physical	Self-Report or Self-Administered Survey	Lifetime	Anyone or Not-Specified		Anxiety Disorder	Mortality	self-report	50.8 (0.6)	Combined Men/Women			
Raposo	2014	USA	Prospective Cohort	Physical	Self-Report or Self-Administered Survey	Lifetime	Family Member or Caregiver	4	Anxiety Disorder	Incidence	self-report	65–99	Combined Men/Women	7177		
Danese	2023	USA	Prospective Cohort	Physical	Self-Report or Self-Administered Survey ; Routinely Collected/Administrative Data	Lifetime	Anyone or Not-Specified	40	Anxiety Disorder	Incidence	self-report	41.2 (3.5)	Combined Men/Women	435		
Danese	2023	USA	Prospective Cohort	Physical	Self-Report or Self-Administered Survey ; Routinely Collected/Administrative Data	Lifetime	Anyone or Not-Specified	40	Anxiety Disorder	Incidence	self-report	41.2 (3.5)	Combined Men/Women	754		
Danese	2023	USA	Prospective Cohort	Physical	Self-Report or Self-Administered Survey ; Routinely Collected/Administrative Data	Lifetime	Anyone or Not-Specified	40	Anxiety Disorder	Incidence	self-report	41.2 (3.5)	Combined Men/Women	514		

Author	Year	Location	Study Design	Type of Violence	Exposure Assessment Method	Exposure Recall	Type of Perpetrator	Follow-up Years	Outcome	Type of Outcome	Outcome Assessment Method	Age Summary	Gender	Sample Size	Number of Cases (Exposed Group)	Number of Cases (Unexposed Group)
Hovens	2015	Netherlands	Retrospective Cohort	Physical	Self-Report or Self-Administered Survey	Lifetime	Anyone or Not-Specified	2	Anxiety Disorder	Incidence	self-report	42.6 (13.9)	Combined Men/Women	1007	3	
Hovens	2015	Netherlands	Retrospective Cohort	Physical	Self-Report or Self-Administered Survey	Lifetime	Anyone or Not-Specified	2	Anxiety Disorder	Incidence	self-report	42.6 (13.9)	Combined Men/Women	1007	2	
Kisely	2021	Australia	Prospective Cohort	Physical	Self-Report or Self-Administered Survey ; Routinely Collected/Administrative Data	Lifetime	Anyone or Not-Specified	30	Any Anxiety Disorder (Last 30 Days)	Incidence	self-report	30	Combined Men/Women			
Kisely	2021	Australia	Prospective Cohort	Physical	Self-Report or Self-Administered Survey ; Routinely Collected/Administrative Data	Lifetime	Anyone or Not-Specified	30	Any Post-Traumatic Disorder (Last 30 Days)	Incidence	self-report	30	Combined Men/Women			
Njoroge	2023	New York	Prospective Cohort	Physical	Self-Report or Self-Administered Survey	Lifetime	Anyone or Not-Specified		Asthma	Incidence	self-report; physician diagnosis	53.1 (14.2)	Combined Men/Women	454	32	69
Sun	2024	China	Prospective Cohort	Physical	Self-Report or Self-Administered Survey	Lifetime	Anyone or Not-Specified	7	Asthma	Incidence	self-report; physician diagnosis	61.48 (9.38)	Combined Men/Women	12277		
Abajobir	2017	Australia	Prospective Cohort	Physical	Routinely Collected/Administrative Data	Lifetime	Family Member or Caregiver	21	Asthma	Incidence	self-report; physician diagnosis	21–21	Combined Men/Women	3762		
Kascakova	2022	Czechia	Retrospective Cohort	Physical	Self-Report or Self-Administered Survey	Lifetime	Anyone or Not-Specified		Asthma	Incidence	self-report	46.2 (16.6)	Combined Men/Women	1018		
Kascakova	2022	Czechia	Retrospective Cohort	Physical	Self-Report or Self-Administered Survey	Lifetime	Anyone or Not-Specified		Asthma	Incidence	self-report	46.4 (17.4)	Combined Men/Women	1800		
Han	2022	Multiple	Prospective Cohort	Physical	Self-Report or Self-Administered Survey	Lifetime	Anyone or Not-Specified	11	Asthma	Incidence	self-report; physician diagnosis	54.2 (7.9)	Combined Men/Women	81105		
Coogan	2013	USA	Prospective Cohort	Physical	Self-Report or Self-Administered Survey	Lifetime	Anyone or Not-Specified	16	Adult-Onset Asthma	Incidence	self-report	21–99	Women		228	411
Coogan	2013	USA	Prospective Cohort	Physical	Self-Report or Self-Administered Survey	Lifetime	Anyone or Not-Specified	16	Adult-Onset Asthma	Incidence	self-report	21–99	Women		406	411
Xiang	2021	USA	Prospective Cohort	Physical	Self-Report or Self-Administered Survey	Lifetime	Parent	8	Major Depressive Disorder	Incidence	self-report	65.4	Combined Men/Women	16946	307	860
Xiao	2022	Yunnan	Case-Control	Physical	Self-Report or Self-Administered Survey	Lifetime	Anyone or Not-Specified		Any Depressive Disorder	Mortality	physician diagnosis	13.47 (1.71)	Combined Men/Women	1134	150	
Su	2022	Canada	Prospective Cohort	Physical	Self-Report or Self-Administered Survey	Lifetime	Anyone or Not-Specified	6	Major Depression	Incidence	self-report	50.6 (13.8)	Combined Men/Women	1351		
Merza	2015	Hungary	Case-Control	Physical	Self-Report or Self-Administered Survey	Lifetime	Family Member or Caregiver		Major Depressive Disorder	Mortality	physician diagnosis	44.3 (5.91)	Combined Men/Women	124	14	

Author	Year	Location	Study Design	Type of Violence	Exposure Assessment Method	Exposure Recall	Type of Perpetrator	Follow-up Years	Outcome	Type of Outcome	Outcome Assessment Method	Age Summary	Gender	Sample Size	Number of Cases (Exposed Group)	Number of Cases (Unexposed Group)
Li	2024	China	Retrospective Cohort	Physical	Self-Report or Self-Administered Survey	Lifetime	Anyone or Not-Specified	4	Depressive Symptoms	Incidence	self-report	61.48 (9.38)	Combined Men/Women	12277	1513	2992
Bhattarai	2023	Canada	Prospective Cohort	Physical	Self-Report or Self-Administered Survey	Lifetime	Family Member or Caregiver	2	Depression	Incidence	self-report	18.1 (1.6)	Combined Men/Women			
Kang	2023	Republic Of Korea	Case-Control	Physical	Self-Report or Self-Administered Survey	Lifetime	Anyone or Not-Specified		Depression	Mortality	physician diagnosis	19–65	Combined Men/Women	124	44	3
Yu	2023	United Kingdom	Prospective Cohort	Physical	Self-Report or Self-Administered Survey	Lifetime	Family Member or Caregiver	16	Depression	Incidence	self-report; administrative medical records or disease registries	55.852 (7.77)	Combined Men/Women	126064		
Widom	2023	USA	Case-Control	Physical	Routinely Collected/Administrative Data		Anyone or Not-Specified		Depression	Mortality	self-report; biomarker	41.2	Combined Men/Women	403	19	49
Elbarazi	2023	Egypt	Prospective Cohort	Physical	Self-Report or Self-Administered Survey	Lifetime	Anyone or Not-Specified	1	Depression	Incidence	self-report	19.03 (0.46)	Combined Men/Women	319		
Fergusson	2008	New Zealand	Prospective Cohort	Physical	Self-Report or Self-Administered Survey	Lifetime	Family Member or Caregiver	25	Depression	Incidence	self-report	16–25	Combined Men/Women	1001		
Roustit	2009	France	Retrospective Cohort	Physical	Self-Report or Self-Administered Survey	Lifetime	Anyone or Not-Specified		Depression	Incidence	self-report	18–99	Combined Men/Women	3023		
Widom	2007	USA	Prospective Cohort	Physical	Routinely Collected/Administrative Data	Lifetime	Anyone or Not-Specified		Current Major Depressive Disorder (Mdd) Diagnosis		self-report; interview	28.7 (3.8)	Combined Men/Women	1302	15	
Andrews	1995	United Kingdom	Prospective Cohort	Physical	Self-Report or Self-Administered Survey	Lifetime	Family Member or Caregiver	3	Depression	Incidence	self-report		Women	101	8	8
Brown	1999	New York	Prospective Cohort	Physical	Self-Report or Self-Administered Survey ; Routinely Collected/Administrative Data	Lifetime	Anyone or Not-Specified	17	Adult Depressive Disorders	Incidence	self-report	18–99	Combined Men/Women		7	28
Wise	2001	Massachusetts	Case-Control	Physical	Self-Report or Self-Administered Survey	Lifetime	Anyone or Not-Specified		Depressive Disorders	Mortality	self-report; physician diagnosis	36–45	Women	520	92	51
Mullen	1996	New Zealand	Retrospective Cohort	Physical	Self-Report or Self-Administered Survey	Lifetime	Family Member or Caregiver; Parent		Depression	Incidence	self-report	18–65	Women	497		
Comijs	2013	Netherlands	Case-Control	Physical	Self-Report or Self-Administered Survey	Lifetime	Anyone or Not-Specified		Depressive Disorders	Mortality	self-report	70.5	Combined Men/Women	508	60	

Author	Year	Location	Study Design	Type of Violence	Exposure Assessment Method	Exposure Recall	Type of Perpetrator	Follow-up Years	Outcome	Type of Outcome	Outcome Assessment Method	Age Summary	Gender	Sample Size	Number of Cases (Exposed Group)	Number of Cases (Unexposed Group)
Kisely	2021	Australia	Prospective Cohort	Physical	Self-Report or Self-Administered Survey ; Routinely Collected/Administrative Data	Lifetime	Anyone or Not-Specified	30	Any Depressive Disorder (Last 30 Days)	Incidence	self-report	30	Combined Men/Women			
Ebert	2019	Belgium	Prospective Cohort	Physical	Self-Report or Self-Administered Survey	Lifetime	Anyone or Not-Specified	1	Major Depressive Disorder	Incidence	self-report	19	Combined Men/Women	2242		
Danese	2023	USA	Prospective Cohort	Physical	Self-Report or Self-Administered Survey ; Routinely Collected/Administrative Data	Lifetime	Anyone or Not-Specified	40	Depression	Incidence	self-report	41.2 (3.5)	Combined Men/Women	435		
Danese	2023	USA	Prospective Cohort	Physical	Self-Report or Self-Administered Survey ; Routinely Collected/Administrative Data	Lifetime	Anyone or Not-Specified	40	Depression	Incidence	self-report	41.2 (3.5)	Combined Men/Women	754		
Danese	2023	USA	Prospective Cohort	Physical	Self-Report or Self-Administered Survey ; Routinely Collected/Administrative Data	Lifetime	Anyone or Not-Specified	40	Depression	Incidence	self-report	41.2 (3.5)	Combined Men/Women	514		
Zhou	2023	Beijing	Case-Control	Physical	Self-Report or Self-Administered Survey	Lifetime	Anyone or Not-Specified		Major Depressive Disorder	Mortality	administrative medical records or disease registries; physician diagnosis	30.5 (9.29)	Women	370	12	173
Zhou	2023	Beijing	Case-Control	Physical	Self-Report or Self-Administered Survey	Lifetime	Anyone or Not-Specified		Major Depressive Disorder	Mortality	administrative medical records or disease registries; physician diagnosis	30.5 (9.29)	Men	173	9	96
Chapman	2004	California	Retrospective Cohort	Physical	Self-Report or Self-Administered Survey	Lifetime	Anyone or Not-Specified		Depressive Disorders	Incidence	self-report	56.6	Women			
Chapman	2004	California	Retrospective Cohort	Physical	Self-Report or Self-Administered Survey	Lifetime	Anyone or Not-Specified		Depressive Disorders	Incidence	self-report	56.6	Men			
Hovens	2015	Netherlands	Retrospective Cohort	Physical	Self-Report or Self-Administered Survey	Lifetime	Anyone or Not-Specified	2	Depressive Disorder	Incidence	self-report	42.6 (13.9)	Combined Men/Women	1038	7	
Hovens	2015	Netherlands	Retrospective Cohort	Physical	Self-Report or Self-Administered Survey	Lifetime	Anyone or Not-Specified	2	Depressive Disorder	Incidence	self-report	42.6 (13.9)	Combined Men/Women	1038	6	

Author	Year	Location	Study Design	Type of Violence	Exposure Assessment Method	Exposure Recall	Type of Perpetrator	Follow-up Years	Outcome	Type of Outcome	Outcome Assessment Method	Age Summary	Gender	Sample Size	Number of Cases (Exposed Group)	Number of Cases (Unexposed Group)
Galloeag	2017	Rio Grande Do Sul	Prospective Cohort	Physical	Self-Report or Self-Administered Survey	Lifetime	Family Member or Caregiver	18	Major Depression	Incidence	self-report	18	Women	1954	27	167
Galloeag	2017	Rio Grande Do Sul	Prospective Cohort	Physical	Self-Report or Self-Administered Survey	Lifetime	Family Member or Caregiver	18	Major Depression	Incidence	self-report	18	Men	1761	5	51
Houtepen	2020	Bristol, City Of	Prospective Cohort	Physical	Self-Report or Self-Administered Survey ; Parental Report	Lifetime	Family Member or Caregiver	18	Depression	Incidence	self-report	22–22	Combined Men/Women	9959		
Afifi	2013	USA	Retrospective Cohort	Physical	Self-Report or Self-Administered Survey	Lifetime	Family Member or Caregiver; Other Adult Living In Home		Diabetes	Incidence	self-report	20–99	Combined Men/Women	20607	120	1654
Monnat	2015	USA	Retrospective Cohort	Physical	Self-Report or Self-Administered Survey	Lifetime	Anyone or Not-Specified		Diabetes	Incidence	self-report	43.9 (12.55)	Combined Men/Women	52250		
Njoroge	2023	New York	Prospective Cohort	Physical	Self-Report or Self-Administered Survey	Lifetime	Anyone or Not-Specified		Diabetes	Incidence	self-report; physician diagnosis	53.1 (14.2)	Combined Men/Women	454	45	150
Gaston	2023	USA	Prospective Cohort	Physical	Self-Report or Self-Administered Survey	Lifetime	Anyone or Not-Specified	6	Type 2 Diabetes	Incidence	self-report; physician diagnosis	55.2 (0.04)	Women			
Widom	2023	USA	Case-Control	Physical	Routinely Collected/Administrative Data		Anyone or Not-Specified		Diabetes	Mortality	self-report; biomarker	41.2	Combined Men/Women	403	14	43
Thomas	2008	Multiple	Prospective Cohort	Physical	Self-Report or Self-Administered Survey	Lifetime	Family Member or Caregiver	38	Type 2 Diabetes Mellitus or Hba1c >= 6	Incidence	physician diagnosis; biomarker	45–45	Combined Men/Women	9310		
Kascakova	2022	Czechia	Retrospective Cohort	Physical	Self-Report or Self-Administered Survey	Lifetime	Anyone or Not-Specified		Diabetes Mellitus	Incident	self-report	46.2 (16.6)	Combined Men/Women	1018		
Kascakova	2022	Czechia	Retrospective Cohort	Physical	Self-Report or Self-Administered Survey	Lifetime	Anyone or Not-Specified		Diabetes Mellitus	Incident	self-report	46.4 (17.4)	Combined Men/Women	1800		
Shields	2016	Canada	Retrospective Cohort	Physical	Self-Report or Self-Administered Survey	Lifetime	Anyone or Not-Specified		Diabetes	Incidence	self-report		Combined Men/Women	21878		
Zhu	2023	China	Prospective Cohort	Physical	Self-Report or Self-Administered Survey	Lifetime	Anyone or Not-Specified	7	Diabetes	Incidence	self-report; physician diagnosis; biomarker	45–99	Men	4409		
Zhu	2023	China	Prospective Cohort	Physical	Self-Report or Self-Administered Survey	Lifetime	Anyone or Not-Specified	7	Diabetes	Incidence	self-report; physician diagnosis; biomarker	45–99	Women	4770		

Author	Year	Location	Study Design	Type of Violence	Exposure Assessment Method	Exposure Recall	Type of Perpetrator	Follow-up Years	Outcome	Type of Outcome	Outcome Assessment Method	Age Summary	Gender	Sample Size	Number of Cases (Exposed Group)	Number of Cases (Unexposed Group)
Rich-Edwards	2010	USA	Prospective Cohort	Physical	Self-Report or Self-Administered Survey	Lifetime	Parent, Step-Parent, or Adult Guardian	16	Type 2 Diabetes	Incidence	self-report	34.64	Women	43990	338	828
Rich-Edwards	2010	USA	Prospective Cohort	Physical	Self-Report or Self-Administered Survey	Lifetime	Parent, Step-Parent, or Adult Guardian	16	Type 2 Diabetes	Incidence	self-report	34.64	Women	49167	633	828
Rich-Edwards	2010	USA	Prospective Cohort	Physical	Self-Report or Self-Administered Survey	Lifetime	Parent, Step-Parent, or Adult Guardian	16	Type 2 Diabetes	Incidence	self-report	34.64	Women	37276	272	828
Duncan	2015	USA	Prospective Cohort	Physical	Self-Report or Self-Administered Survey	Lifetime	Family Member or Caregiver	15	Diabetes	Incidence	self-report; physician diagnosis; biomarker	24–34	Women		83	450
Duncan	2015	USA	Prospective Cohort	Physical	Self-Report or Self-Administered Survey	Lifetime	Family Member or Caregiver	15	Diabetes	Incidence	self-report; physician diagnosis; biomarker	24–34	Women		79	450
Duncan	2015	USA	Prospective Cohort	Physical	Self-Report or Self-Administered Survey	Lifetime	Family Member or Caregiver	15	Diabetes	Incidence	self-report; physician diagnosis; biomarker	24–34	Men		74	351
Duncan	2015	USA	Prospective Cohort	Physical	Self-Report or Self-Administered Survey	Lifetime	Family Member or Caregiver	15	Diabetes	Incidence	self-report; physician diagnosis; biomarker	24–34	Men		61	351
Lown	2019	USA	Retrospective Cohort	Physical	Self-Report or Self-Administered Survey	Lifetime	Anyone or Not-Specified		Type 2 Diabetes Mellitus	Incidence	self-report	14–56	Women	3726	89	339
Lown	2019	USA	Retrospective Cohort	Physical	Self-Report or Self-Administered Survey	Lifetime	Anyone or Not-Specified		Type 2 Diabetes Mellitus	Incidence	self-report	14–56	Men	3332	56	311
Scheidell	2018	USA	Prospective Cohort	Physical	Self-Report or Self-Administered Survey	Lifetime	Family Member or Caregiver	14	Cocaine Use	Incidence	self-report	24–34	Combined Men/Women	12288		
Duncan	2008	USA	Retrospective Cohort	Physical	Self-Report or Self-Administered Survey	Prior To 16 or Between 6-12 For Early Home Environment Section.	Anyone or Not-Specified; Family Member or Caregiver		Cannabis Abuse or Dependence	Incidence	self-report	22.64 (4.44)	Combined Men/Women	819	30	

Author	Year	Location	Study Design	Type of Violence	Exposure Assessment Method	Exposure Recall	Type of Perpetrator	Follow-up Years	Outcome	Type of Outcome	Outcome Assessment Method	Age Summary	Gender	Sample Size	Number of Cases (Exposed Group)	Number of Cases (Unexposed Group)
Cohen	2001	New York	Prospective Cohort	Physical	Self-Report or Self-Administered Survey ; Routinely Collected/Administrative Data	Lifetime	Family Member or Caregiver	17	Drug Abuse In Early Adulthood	Incidence	self-report	22	Combined Men/Women	664		
Huang	2011	USA	Prospective Cohort	Physical	Self-Report or Self-Administered Survey	Lifetime	Family Member or Caregiver	7	Illicit Drug Use In The Past Year	Incidence	self-report	21.8 (0.12)	Combined Men/Women	4882	287	
Kisely	2021	Australia	Prospective Cohort	Physical	Routinely Collected/Administrative Data	Lifetime	Anyone or Not-Specified	30	Any Substance Use Disorder	Incidence	physician diagnosis; diagnostic interview	30–30	Combined Men/Women			
Najman	2022	Australia	Prospective Cohort	Physical	Self-Report or Self-Administered Survey	Lifetime	Anyone or Not-Specified	30	Drug Use Disorder	Incidence	self-report	30–30	Combined Men/Women	2474		
Telfar	2023	New Zealand	Prospective Cohort	Physical	Self-Report or Self-Administered Survey	Lifetime	Family Member or Caregiver	40	Cannabis Abuse/Dependence	Incidence	self-report		Combined Men/Women			
Broekhof	2023	Trondelag	Prospective Cohort	Physical	Self-Report or Self-Administered Survey	Lifetime	Anyone or Not-Specified		Drug Use Disorder	Incidence	administrative medical records or disease registries	15.91 (0.03)	Women	4129	4	15
Broekhof	2023	Trondelag	Prospective Cohort	Physical	Self-Report or Self-Administered Survey	Lifetime	Anyone or Not-Specified		Drug Use Disorder	Incidence	administrative medical records or disease registries	15.85 (0.03)	Men	4070	28	39
Libby	2004	USA	Prospective Cohort	Physical	Self-Report or Self-Administered Survey	Lifetime	Family Member or Caregiver; Acquaintance		Drug Dependence	Incidence	self-report	33.76 (0.08)	Combined Men/Women			
Libby	2004	USA	Prospective Cohort	Physical	Self-Report or Self-Administered Survey	Lifetime	Family Member or Caregiver; Acquaintance		Drug Dependence	Incidence	self-report	34.29 (0.08)	Combined Men/Women			
Conroy	2009	Australia	Case-Control	Physical	Self-Report or Self-Administered Survey	Lifetime	Anyone or Not-Specified		Opioid-Dependency	Mortality	enrollment in a pharmacotherapy program as clinical determination of opioid dependence	35	Men	742	338	220
Conroy	2009	Australia	Case-Control	Physical	Self-Report or Self-Administered Survey	Lifetime	Anyone or Not Specified		Opioid-Dependency	Mortality	enrollment in a pharmacotherapy program as	35	Women	571	223	156

Author	Year	Location	Study Design	Type of Violence	Exposure Assessment Method	Exposure Recall	Type of Perpetrator	Follow-up Years	Outcome	Type of Outcome	Outcome Assessment Method	Age Summary	Gender	Sample Size	Number of Cases (Exposed Group)	Number of Cases (Unexposed Group)
											clinical determination of opioid dependence					
Tanaka	2015	Canada	Prospective Cohort	Physical	Self-Report or Self-Administered Survey	Lifetime	Adult	18	Drug Use	Incidence	self-report	21–35	Women	969		
Tanaka	2015	Canada	Prospective Cohort	Physical	Self-Report or Self-Administered Survey	Lifetime	Adult	18	Drug Use	Incidence	self-report	21–35	Women	969		
Tanaka	2015	Canada	Prospective Cohort	Physical	Self-Report or Self-Administered Survey	Lifetime	Adult	18	Drug Use	Incidence	self-report	21–35	Men	924		
Rayworth	2004	Massachusetts	Case-Control	Physical	Self-Report or Self-Administered Survey	Lifetime	Anyone or Not-Specified		Eating Disorders	Mortality	self-report	36–48	Women	617	24	17
Andrews	1995	United Kingdom	Prospective Cohort	Physical	Self-Report or Self-Administered Survey	Lifetime	Family Member or Caregiver	3	Bulimia	Incidence	self-report	15–28	Women	75	4	5
Mullen	1996	New Zealand	Retrospective Cohort	Physical	Self-Report or Self-Administered Survey	Lifetime	Family Member or Caregiver; Parent		Eating Disorder	Incidence	self-report	18–65	Women	497		
Talmon	2021	USA	Prospective And Retrospective (Different Models)	Physical	Self-Report or Self-Administered Survey ; Routinely Collected/Administrative Data	Lifetime	Anyone or Not-Specified	16	Anorexia Nervosa	Incidence	physician diagnosis	41.2 (3.54)	Combined Men/Women	700	13	8
Talmon	2021	USA	Prospective And Retrospective (Different Models)	Physical	Self-Report or Self-Administered Survey ; Routinely Collected/Administrative Data	Lifetime	Anyone or Not-Specified	16	Bulimia Nervosa	Incidence	physician diagnosis	41.2 (3.54)	Combined Men/Women	700	7	1
Harris	2018	USA	Prospective Cohort	Physical	Self-Report or Self-Administered Survey	Lifetime	Family Member or Caregiver		Laparoscopically-Confirmed Endometriosis	Incidence	self-report; administrative medical records or disease registries; physician diagnosis	25–42	Women		1071	1066
Liebermann	2018	Multiple	Case-Control	Physical	Self-Report or Self-Administered Survey	Lifetime	Anyone or Not-Specified		Endometriosis	Mortality	physician diagnosis	37.3 (7.3)	Women	842	128	
Ito	2021	Aichi	Case-Control	Physical	Self-Report or Self-Administered Survey	Lifetime	Family Member or Caregiver		Premenstrual Syndrome (Pms)	Mortality	self-report	35.1 (10.3)	Women	3815		

Author	Year	Location	Study Design	Type of Violence	Exposure Assessment Method	Exposure Recall	Type of Perpetrator	Follow-up Years	Outcome	Type of Outcome	Outcome Assessment Method	Age Summary	Gender	Sample Size	Number of Cases (Exposed Group)	Number of Cases (Unexposed Group)
Harlow	2005	Massachusetts	Case-Control	Physical	Self-Report or Self-Administered Survey	Lifetime	Anyone or Not-Specified		Vulvodynia	Mortality	self-report; physician diagnosis	18–64	Women	104		
Bertone-Johnson	2014	USA	Case-Control	Physical	Self-Report or Self-Administered Survey	Lifetime	Family Member or Caregiver		Premenstrual Syndrome	Mortality	self-report; physician diagnosis	34	Women	3288	104	
Boynton-Jarrett	2011	USA	Prospective Cohort	Physical	Self-Report or Self-Administered Survey	Lifetime	Anyone or Not-Specified		Uterine Leiomyomata	Incidence	self-report	34 (5)	Women		3151	3214
Monnat	2015	USA	Retrospective Cohort	Physical	Self-Report or Self-Administered Survey	Lifetime	Anyone or Not-Specified		Heart Attack	Incidence	self-report	43.9 (12.55)	Combined Men/Women	52250		
Dong	2004	California	Retrospective Cohort	Physical	Self-Report or Self-Administered Survey	Lifetime	Family Member or Caregiver; Parent or Other Adult In The Household	2	Ischemic Heart Disease	Incidence	self-report	56 (15.2)	Combined Men/Women	17337	623	1205
Akasaki	2021	Greater London	Prospective Cohort	Physical	Self-Report or Self-Administered Survey	Lifetime	Anyone or Not-Specified	12.9	Coronary Heart Disease	Incidence	administrative medical records or disease registries; physician diagnosis	43.6	Combined Men/Women	5149	10	
Kascakova	2022	Czechia	Retrospective Cohort	Physical	Self-Report or Self-Administered Survey	Lifetime	Anyone or Not-Specified		Ischemic Heart Disease	Incident	self-report	46.2 (16.6)	Combined Men/Women	1018		
Kascakova	2022	Czechia	Retrospective Cohort	Physical	Self-Report or Self-Administered Survey	Lifetime	Anyone or Not-Specified		Ischemic Heart Disease	Incident	self-report	46.4 (17.4)	Combined Men/Women	1800		
Gelaye	2016	Peru	Retrospective Cohort	Physical	Self-Report or Self-Administered Survey	Lifetime	Anyone or Not-Specified		Migraine	Incidence	self-report	28.1	Women	2970	377	240
Karmakar	2017	USA	Prospective Cohort	Physical	Self-Report or Self-Administered Survey	Lifetime	Family Member or Caregiver	14	Migraine Headaches	Incidence	self-report; physician diagnosis	24–32	Combined Men/Women	13593	427	1500
Kascakova	2022	Czechia	Retrospective Cohort	Physical	Self-Report or Self-Administered Survey	Lifetime	Anyone or Not-Specified		Migraine	Incdient	self-report	46.2 (16.6)	Combined Men/Women	1018		
Kascakova	2022	Czechia	Retrospective Cohort	Physical	Self-Report or Self-Administered Survey	Lifetime	Anyone or Not-Specified		Migraine	Incdient	self-report	46.4 (17.4)	Combined Men/Women	1800		
Brennenstuhl	2015	Canada	Retrospective Cohort	Physical	Self-Report or Self-Administered Survey	Lifetime	Anyone or Not-Specified		Migraine	Incidence	self-report; physician diagnosis	46.4 (17.3)	Men	10358		
Brennenstuhl	2015	Canada	Retrospective Cohort	Physical	Self-Report or Self-Administered Survey	Lifetime	Anyone or Not-Specified		Migraine	Incidence	self-report; physician diagnosis	47.5 (17.7)	Women	12638		

Author	Year	Location	Study Design	Type of Violence	Exposure Assessment Method	Exposure Recall	Type of Perpetrator	Follow-up Years	Outcome	Type of Outcome	Outcome Assessment Method	Age Summary	Gender	Sample Size	Number of Cases (Exposed Group)	Number of Cases (Unexposed Group)
Trotta	2023	Multiple	Case-Control	Physical	Self-Report or Self-Administered Survey	Lifetime	Family Member or Caregiver		Psychotic Disorder	Mortality	self-report	33.97 (12.58)	Combined Men/Women	2112	265	616
Alkema	2023	Netherlands	Case-Control	Physical	Self-Report or Self-Administered Survey	Lifetime	Anyone or Not-Specified		Schizophrenia	Mortality	physician diagnosis	18–99	Combined Men/Women	4037		
Mansueto	2022	Toscana	Case-Control	Physical	Self-Report or Self-Administered Survey	Lifetime	Anyone or Not-Specified		Childhood Physical Abuse	Mortality	administrative medical records or disease registries; physician diagnosis	43.07 (10.9)	Women	132	8	36
Mansueto	2022	Toscana	Case-Control	Physical	Self-Report or Self-Administered Survey	Lifetime	Anyone or Not-Specified		Psychosis	Mortality	administrative medical records or disease registries; physician diagnosis	43.07 (10.9)	Men	102	10	24
Chatziioannidis	2019	Greece	Case-Control	Physical	Self-Report or Self-Administered Survey	Lifetime	Mother		Schizophrenia And Other Psychotic Disorders	Mortality	physician diagnosis	40 (10)	Combined Men/Women	124	14	
Chatziioannidis	2019	Greece	Case-Control	Physical	Self-Report or Self-Administered Survey	Lifetime	Father		Schizophrenia And Other Psychotic Disorders	Mortality	physician diagnosis	40 (10)	Combined Men/Women	124	14	
Kiselydmedres	2022	Australia	Prospective Cohort	Physical	Self-Report or Self-Administered Survey ; Routinely Collected/Administrative Data	Lifetime	Anyone or Not-Specified	30	Any Self-Harm	Incidence	self-report	30–30				
Salzinger	2007	New York	Prospective Cohort	Physical	Routinely Collected/Administrative Data	Lifetime	Anyone or Not-Specified	6	Suicide Attempt	Incidence	self-report	16.5 (0.53)	Combined Men/Women	153		
Archambault	2023	Canada	Prospective Cohort	Physical	Routinely Collected/Administrative Data ; Clinical Examination	Lifetime	Anyone or Not-Specified	5	Intentional Self-Injury	Incidence	administrative medical records or disease registries; physician diagnosis	18–18	Combined Men/Women	27435		
Geng	2023	Shandong	Case-Control	Physical	Self-Report or Self-Administered Survey	Lifetime	Anyone or Not-Specified		Self-Injury	Mortality	administrative medical records or disease registries; physician diagnosis	12–20	Combined Men/Women	414	43	95

Author	Year	Location	Study Design	Type of Violence	Exposure Assessment Method	Exposure Recall	Type of Perpetrator	Follow-up Years	Outcome	Type of Outcome	Outcome Assessment Method	Age Summary	Gender	Sample Size	Number of Cases (Exposed Group)	Number of Cases (Unexposed Group)
Calegaro	2023	Rio Grande Do Sul	Prospective Cohort	Physical	Self-Report or Self-Administered Survey	Last 6 Months	Parents	22	Lifetime Suicide Attempts	Incidence	self-report	22–22	Combined Men/Women	3050		
Bhattarai	2023	Canada	Prospective Cohort	Physical	Self-Report or Self-Administered Survey	Lifetime	Family Member or Caregiver	2	Non-Suicidal Self-Harm	Incidence	self-report	18.1 (1.6)	Combined Men/Women			
Roustit	2009	France	Retrospective Cohort	Physical	Self-Report or Self-Administered Survey	Lifetime	Anyone or Not-Specified		Lifetime Suicide Attempt	Incidence	self-report	18–99	Combined Men/Women	3023		
Brown	1999	New York	Prospective Cohort	Physical	Self-Report or Self-Administered Survey ; Routinely Collected/Administrative Data	Lifetime	Anyone or Not-Specified	17	Adult Suicide Attempt	Incidence	self-report	18–99	Combined Men/Women	602	5	18
Mullen	1996	New Zealand	Retrospective Cohort	Physical	Self-Report or Self-Administered Survey	Lifetime	Family Member or Caregiver; Parent		Ever Suicide Attempt	Incidence	self-report	18–65	Women	497		
Enns	2006	Netherlands	Prospective Cohort	Physical	Self-Report or Self-Administered Survey	Lifetime	Anyone or Not-Specified	3	Suicidal Attempts	Incidence	self-report	18–64	Combined Men/Women	5670		
Johnson	2002	New York	Prospective Cohort	Physical	Routinely Collected/Administrative Data	Lifetime	Anyone or Not-Specified; Parent	18	Suicide Attempts	Incidence	self-report	22 (3)	Combined Men/Women	623	5	16
Thompson	2019	USA	Prospective Cohort	Physical	Self-Report or Self-Administered Survey	Lifetime	Parent or Adult Caregiver	13	Suicide Attempts	Incidence	self-report	15.03 (0.11)	Combined Men/Women	9421		
Russell	2019	Multiple	Prospective Cohort	Physical	Self-Report or Self-Administered Survey	Lifetime	Anyone or Not-Specified		Self-Harm	Incidence	self-report	0–16	Combined Men/Women	4308		
Bruffaerts	2010	Multiple	Retrospective Cohort	Physical	Self-Report or Self-Administered Survey	Lifetime	Anyone or Not-Specified		Suicide Attempt	Incidence	self-report	20–29	Combined Men/Women	109377		
Bruffaerts	2010	Multiple	Retrospective Cohort	Physical	Self-Report or Self-Administered Survey	Lifetime	Anyone or Not-Specified		Suicide Attempt	Incidence	self-report	30–99	Combined Men/Women	109377		
Fried	2013	USA	Prospective Cohort	Physical	Self-Report or Self-Administered Survey	Lifetime	Guardian	8	Suicide Attempts	Incidence	self-report	18–26	Combined Men/Women	1648	32	49
Fried	2013	USA	Prospective Cohort	Physical	Self-Report or Self-Administered Survey	Lifetime	Guardian	8	Suicide Attempts	Incidence	self-report	18–26	Combined Men/Women	1728	23	23
Rajapakse	2020	Sri Lanka	Case-Control	Physical	Self-Report or Self-Administered Survey	Lifetime	Family Member or Caregiver; Other Household Member		Self-Poisoning	Mortality	self-report; administrative medical records or disease registries	25	Combined Men/Women	686	7	

Author	Year	Location	Study Design	Type of Violence	Exposure Assessment Method	Exposure Recall	Type of Perpetrator	Follow-up Years	Outcome	Type of Outcome	Outcome Assessment Method	Age Summary	Gender	Sample Size	Number of Cases (Exposed Group)	Number of Cases (Unexposed Group)
Widom	2012	USA	Prospective Cohort	Physical	Routinely Collected/Administrative Data	Lifetime	Anyone or Not-Specified	38	Syphilis	Incidence	physician diagnosis; biomarker	41.2	Combined Men/Women	807	57	234
Wilson	2009	USA	Prospective Cohort	Physical	Routinely Collected/Administrative Data	Lifetime	Anyone or Not-Specified	30	Chlamydia	Incidence	self-report; physician diagnosis	41.2	Combined Men/Women			
Wilson	2009	USA	Prospective Cohort	Physical	Routinely Collected/Administrative Data	Lifetime	Anyone or Not-Specified	30	Genital Herpes	Incidence	self-report; physician diagnosis	41.2	Combined Men/Women			
Wilson	2009	USA	Prospective Cohort	Physical	Routinely Collected/Administrative Data	Lifetime	Anyone or Not-Specified	30	Gonorrhea	Incidence	self-report; physician diagnosis	41.2	Combined Men/Women			
London	2017	USA	Prospective Cohort	Physical	Self-Report or Self-Administered Survey	Lifetime	Anyone or Not-Specified	14	Stis (Sexually Transmitted Infections)	Incidence	self-report; physician diagnosis	24–32	Combined Men/Women	12288	17	
Campbell	2016	Multiple	Retrospective Cohort	Physical	Self-Report or Self-Administered Survey	Lifetime	Parents or Adults In The Home		Stroke	Incidence	self-report	18–99	Combined Men/Women	48526		
Njoroge	2023	New York	Prospective Cohort	Physical	Self-Report or Self-Administered Survey	Lifetime	Anyone or Not-Specified		Stroke	Incidence	self-report; physician diagnosis	53.1 (14.2)	Combined Men/Women	454	7	20
Goodwin	2004	USA	Cross-Sectional	Physical	Self-Report or Self-Administered Survey	Lifetime	Anyone or Not-Specified		Stroke	Incidence	self-report	15–54	Combined Men/Women	8098	4	7
Fenton	2013	USA	Prospective Cohort	Psychological	Self-Report or Self-Administered Survey	Lifetime	Family Member or Caregiver	3	Alcohol Dependence	Incidence	self-report	18–99	Combined Men/Women	27712	1002	3748
Roustit	2009	France	Retrospective Cohort	Witness Interparental Violence	Self-Report or Self-Administered Survey	Lifetime	Family Member or Caregiver		Alcohol Dependence	Incidence	self-report	18–99	Combined Men/Women	3023		
Kascakova	2022	Czechia	Retrospective Cohort	Psychological	Self-Report or Self-Administered Survey	Lifetime	Anyone or Not-Specified		Alcohol Use Disorder	Incident	self-report	46.2 (16.6)	Combined Men/Women	1018		
Kascakova	2022	Czechia	Retrospective Cohort	Psychological	Self-Report or Self-Administered Survey	Lifetime	Anyone or Not-Specified		Alcohol Use Disorder	Incident	self-report	46.4 (17.4)	Combined Men/Women	1800		
Najman	2022	Australia	Prospective Cohort	Psychological	Self-Report or Self-Administered Survey	Lifetime	Anyone or Not-Specified	30	Alcohol Use Disorder	Incidence	self-report	30–30	Combined Men/Women	2474		
Broekhof	2023	Trondelag	Prospective Cohort	Psychological	Self-Report or Self-Administered Survey	Lifetime	Family Member or Caregiver		Alcohol Use Disorder	Incidence	administrative medical records or disease registries	15.91 (0.03)	Women	4129	5	18
Broekhof	2023	Trondelag	Prospective Cohort	Psychological	Self-Report or Self-Administered Survey	Lifetime	Family Member or Caregiver		Alcohol Use Disorder	Incidence	administrative medical records or disease registries	15.85 (0.03)	Men	4070	2	25

Author	Year	Location	Study Design	Type of Violence	Exposure Assessment Method	Exposure Recall	Type of Perpetrator	Follow-up Years	Outcome	Type of Outcome	Outcome Assessment Method	Age Summary	Gender	Sample Size	Number of Cases (Exposed Group)	Number of Cases (Unexposed Group)
Kisely	2021	Australia	Prospective Cohort	Psychological	Routinely Collected/Administrative Data	Lifetime	Anyone or Not-Specified	30	Alcohol Use Disorder	Incidence	self-report; cidi-auto interview, a computerized protocol		Combined Men/Women			
Laflair	2013	USA	Prospective Cohort	Witnessed Domestic Violence	Self-Report or Self-Administered Survey	Lifetime	Family Member or Caregiver	4	Severe Alcohol Involvement (Dependence or Abuse)	Incidence	self-report	18–99	Women	11750		
Tenhave	2019	Netherlands	Prospective Cohort	Psychological	Self-Report or Self-Administered Survey	Lifetime	Anyone or Not-Specified	3	Anxiety Disorders	Incidence	self-report	18–64	Combined Men/Women	9304		
Soenke	2010	USA	Retrospective Cohort	Psychological	Self-Report or Self-Administered Survey	Lifetime	Anyone or Not-Specified		Generalized Anxiety Disorder	Incidence	self-report	20.26 (2.45)	Women	396		
Yu	2023	United Kingdom	Prospective Cohort	Psychological	Self-Report or Self-Administered Survey	Lifetime	Family Member or Caregiver	16	Anxiety Disorder	Incidence	self-report; administrative medical records or disease registries	55.852 (7.77)	Combined Men/Women	126064		
Elbarazi	2023	Egypt	Prospective Cohort	Psychological	Self-Report or Self-Administered Survey	Lifetime	Anyone or Not Specified	1	Anxiety	Incidence	self-report	19.03 (0.46)	Combined Men/Women	319		
Raposo	2014	USA	Prospective Cohort	Psychological	Self-Report or Self-Administered Survey	Lifetime	Family Member or Caregiver	4	Anxiety Disorder	Incidence	self-report	65–99	Combined Men/Women	7177		
Hovens	2015	Netherlands	Retrospective Cohort	Psychological	Self-Report or Self-Administered Survey	Lifetime	Anyone or Not-Specified	2	Anxiety Disorder	Incidence	self-report	42.6 (13.9)	Combined Men/Women	1007	6	
Hovens	2015	Netherlands	Retrospective Cohort	Psychological	Self-Report or Self-Administered Survey	Lifetime	Anyone or Not-Specified	2	Anxiety Disorder	Incidence	self-report	42.6 (13.9)	Combined Men/Women	1007	4	
Kisely	2021	Australia	Prospective Cohort	Psychological	Self-Report or Self-Administered Survey ; Routinely Collected/Administrative Data	Lifetime	Anyone or Not-Specified	30	Any Anxiety Disorder (Last 30 Days)	Incidence	self-report	30	Combined Men/Women			
Kisely	2021	Australia	Prospective Cohort	Psychological	Self-Report or Self-Administered Survey ; Routinely Collected/Administrative Data	Lifetime	Anyone or Not-Specified	30	Any Post-Traumatic Disorder (Last 30 Days)	Incidence	self-report	30	Combined Men/Women			
Njoroge	2023	New York	Prospective Cohort	Psychological	Self-Report or Self-Administered Survey	Lifetime	Anyone or Not-Specified		Asthma	Incidence	self-report; physician diagnosis	53.1 (14.2)	Combined Men/Women	454	23	78
Abajobir	2017	Australia	Prospective Cohort	Psychological	Routinely Collected/Administrative Data	Lifetime	Anyone or Not-Specified	21	Asthma	Incidence	self-report; physician diagnosis	21–21	Combined Men/Women	3762		

Author	Year	Location	Study Design	Type of Violence	Exposure Assessment Method	Exposure Recall	Type of Perpetrator	Follow-up Years	Outcome	Type of Outcome	Outcome Assessment Method	Age Summary	Gender	Sample Size	Number of Cases (Exposed Group)	Number of Cases (Unexposed Group)
Kascakova	2022	Czechia	Retrospective Cohort	Psychological	Self-Report or Self-Administered Survey	Lifetime	Anyone or Not-Specified		Asthma	Incidence	self-report	46.2 (16.6)	Combined Men/Women	1018		
Kascakova	2022	Czechia	Retrospective Cohort	Psychological	Self-Report or Self-Administered Survey	Lifetime	Anyone or Not-Specified		Asthma	Incidence	self-report	46.4 (17.4)	Combined Men/Women	1800		
Han	2022	Multiple	Prospective Cohort	Psychological	Self-Report or Self-Administered Survey	Lifetime	Anyone or Not-Specified	11	Asthma	Incidence	self-report; physician diagnosis	54.2 (7.9)	Combined Men/Women	81105		
Xiao	2022	Yunnan	Case-Control	Psychological	Self-Report or Self-Administered Survey	Lifetime	Anyone or Not-Specified		Any Depressive Disorder	Mortality	physician diagnosis	13.47 (1.71)	Combined Men/Women	1134	279	
Su	2022	Canada	Prospective Cohort	Psychological	Self-Report or Self-Administered Survey	Lifetime	Anyone or Not-Specified	6	Major Depression	Incidence	self-report	50.6 (13.8)	Combined Men/Women	1351		
Kang	2023	Republic Of Korea	Case-Control	Psychological	Self-Report or Self-Administered Survey	Lifetime	Anyone or Not-Specified		Depression	Mortality	physician diagnosis	19–65	Combined Men/Women	104	42	3
Zhang	2023	China	Prospective Cohort	Witnessing Domestic Violence	Self-Report or Self-Administered Survey	Lifetime	Household Member	4	Depression	Incidence	self-report	60.7 (9.5)	Combined Men/Women	14484		
Yu	2023	United Kingdom	Prospective Cohort	Psychological	Self-Report or Self-Administered Survey	Lifetime	Family Member or Caregiver	16	Depression	Incidence	self-report; administrative medical records or disease registries	55.852 (7.77)	Combined Men/Women	126064		
Elbarazi	2023	Egypt	Prospective Cohort	Psychological	Self-Report or Self-Administered Survey	Lifetime	Anyone or Not-Specified	1	Depression	Incidence	self-report	19.03 (0.46)	Combined Men/Women	319		
Roustit	2009	France	Retrospective Cohort	Witnessed Domestic Violence	Self-Report or Self-Administered Survey	Lifetime	Family Member or Caregiver		Depression	Incidence	self-report	18–99	Combined Men/Women	3023		
Mullen	1996	New Zealand	Retrospective Cohort	Psychological	Self-Report or Self-Administered Survey	Lifetime	Family Member or Caregiver; Parent		Depression	Incidence	self-report	18–65	Women	497		
Comijs	2013	Netherlands	Case-Control	Psychological	Self-Report or Self-Administered Survey	Lifetime	Anyone or Not-Specified		Depressive Disorders	Mortality	self-report	70.5	Combined Men/Women	508	99	
Kisely	2021	Australia	Prospective Cohort	Psychological	Self-Report or Self-Administered Survey ; Routinely Collected/Administrative Data	Lifetime	Anyone or Not-Specified	30	Any Depressive Disorder (Last 30 Days)	Incidence	self-report	30	Combined Men/Women			
Ebert	2019	Belgium	Prospective Cohort	Psychological	Self-Report or Self-Administered Survey	Lifetime	Anyone or Not-Specified	1	Major Depressive Disorder	Incidence	self-report	19	Combined Men/Women	2242		
Zhou	2023	Beijing	Case-Control	Psychological	Self-Report or Self-Administered Survey	Lifetime	Anyone or Not-Specified		Major Depressive Disorder	Mortality	administrative medical records or disease registries;	30.5 (9.29)	Women	370	26	159

Author	Year	Location	Study Design	Type of Violence	Exposure Assessment Method	Exposure Recall	Type of Perpetrator	Follow-up Years	Outcome	Type of Outcome	Outcome Assessment Method	Age Summary	Gender	Sample Size	Number of Cases (Exposed Group)	Number of Cases (Unexposed Group)
Zhou	2023	Beijing	Case-Control	Psychological	Self-Report or Self-Administered Survey	Lifetime	Anyone or Not Specified		Major Depressive Disorder	Mortality	physician diagnosis administrative medical records or disease registries; physician diagnosis	30.5 (9.29)	Men	210	17	88
Chapman	2004	California	Retrospective Cohort	Psychological	Self-Report or Self-Administered Survey	Lifetime	Family Member or Caregiver		Depressive Disorders	Incidence	self-report	56.6	Women			
Chapman	2004	California	Retrospective Cohort	Psychological	Self-Report or Self-Administered Survey	Lifetime	Family Member or Caregiver		Depressive Disorders	Incidence	self-report	56.6	Men			
Vaeth	2010	USA	Cross-Sectional	Witnessed Domestic Violence	Self-Report or Self-Administered Survey	Lifetime	Family Member or Caregiver	5	Depression	Incidence	self-report	50 (0.8)	Women	1040		
Vaeth	2010	USA	Cross-Sectional	Witnessed Domestic Violence	Self-Report or Self-Administered Survey	Lifetime	Family Member or Caregiver	5	Depression	Incidence	self-report	52 (0.83)	Men	1040		
Hovens	2015	Netherlands	Retrospective Cohort	Psychological	Self-Report or Self-Administered Survey	Lifetime	Anyone or Not-Specified	2	Depressive Disorder	Incidence	self-report	42.6 (13.9)	Combined Men/Women	1038	11	
Hovens	2015	Netherlands	Retrospective Cohort	Psychological	Self-Report or Self-Administered Survey	Lifetime	Anyone or Not-Specified	2	Depressive Disorder	Incidence	self-report	42.6 (13.9)	Combined Men/Women	1038	7	
Galloeag	2017	Rio Grande Do Sul	Prospective Cohort	Psychological	Self-Report or Self-Administered Survey	Lifetime	Family Member or Caregiver	18	Major Depression	Incidence	self-report	18	Women	1954	101	94
Galloeag	2017	Rio Grande Do Sul	Prospective Cohort	Psychological	Self-Report or Self-Administered Survey	Lifetime	Family Member or Caregiver	18	Major Depression	Incidence	self-report	18	Men	1761	11	47
Houtepen	2020	Bristol, City Of	Prospective Cohort	Psychological	Self-Report or Self-Administered Survey ; Parental Report	Lifetime	Family Member or Caregiver	18	Depression	Incidence	self-report	22–22	Combined Men/Women	9959		
Monnat	2015	USA	Retrospective Cohort	Psychological	Self-Report or Self-Administered Survey	Lifetime	Anyone or Not-Specified		Diabetes	Incidence	self-report	43.9 (12.55)	Combined Men/Women	52250		
Njoroge	2023	New York	Prospective Cohort	Psychological	Self-Report or Self-Administered Survey	Lifetime	Anyone or Not-Specified		Diabetes	Incidence	self-report; physician diagnosis	53.1 (14.2)	Combined Men/Women	454	32	163
Gaston	2023	USA	Prospective Cohort	Psychological	Self-Report or Self-Administered Survey	Lifetime	Anyone or Not-Specified	6	Type 2 Diabetes	Incidence	self-report; physician diagnosis	55.2 (0.04)	Women			
Kascakova	2022	Czechia	Retrospective Cohort	Psychological	Self-Report or Self-Administered Survey	Lifetime	Anyone or Not-Specified		Diabetes Mellitus	Incident	self-report	46.2 (16.6)	Combined Men/Women	1018		

Author	Year	Location	Study Design	Type of Violence	Exposure Assessment Method	Exposure Recall	Type of Perpetrator	Follow-up Years	Outcome	Type of Outcome	Outcome Assessment Method	Age Summary	Gender	Sample Size	Number of Cases (Exposed Group)	Number of Cases (Unexposed Group)
Kascakova	2022	Czechia	Retrospective Cohort	Psychological	Self-Report or Self-Administered Survey	Lifetime	Anyone or Not-Specified		Diabetes Mellitus	Incident	self-report	46.4 (17.4)	Combined Men/Women	1800		
Zhu	2023	China	Prospective Cohort	Psychological	Self-Report or Self-Administered Survey	Lifetime	Anyone or Not-Specified	7	Diabetes	Incidence	self-report; physician diagnosis; biomarker	45–99	Men	4409		
Zhu	2023	China	Prospective Cohort	Psychological	Self-Report or Self-Administered Survey	Lifetime	Anyone or Not-Specified	7	Diabetes	Incidence	self-report; physician diagnosis; biomarker	45–99	Women	4770		
Thomas	2008	Multiple	Prospective Cohort	Psychological	Self-Report or Self-Administered Survey	Lifetime	Family Member or Caregiver	38	Type 2 Diabetes Mellitus or Hba1c >= 6	Incidence	physician diagnosis; biomarker	45–45	Combined Men/Women	9310		
Duncan	2015	USA	Prospective Cohort	Psychological	Self-Report or Self-Administered Survey	Lifetime	Family Member or Caregiver	15	Diabetes	Incidence	self-report; physician diagnosis; biomarker	24–34	Women		94	488
Duncan	2015	USA	Prospective Cohort	Psychological	Self-Report or Self-Administered Survey	Lifetime	Family or Caregiver	15	Diabetes	Incidence	self-report; physician diagnosis; biomarker	24–34	Women		31	488
Duncan	2015	USA	Prospective Cohort	Psychological	Self-Report or Self-Administered Survey	Lifetime	Family Member or Caregiver	15	Diabetes	Incidence	self-report; physician diagnosis; biomarker	24–34	Men		47	429
Duncan	2015	USA	Prospective Cohort	Psychological	Self-Report or Self-Administered Survey	Lifetime	Family Member or Caregiver	15	Diabetes	Incidence	self-report; physician diagnosis; biomarker	24–34	Men		10	429
Scheidell	2018	USA	Prospective Cohort	Psychological	Self-Report or Self-Administered Survey	Lifetime	Family Member or Caregiver	14	Cocaine Use	Incidence	self-report	24–34	Combined Men/Women	12288		
Schwartz	2024	France	Prospective Cohort	Psychological	Self-Report or Self-Administered Survey	Lifetime	Family Member or Caregiver	1	Illegal Drug Use	Incidence	self-report	18–30	Combined Men/Women	1052		
Broekhof	2023	Trondelag	Prospective Cohort	Psychological	Self-Report or Self-Administered Survey	Lifetime	Family Member or Caregiver		Drug Use Disorder	Incidence	administrative medical records or disease registries	15.85 (0.03)	Men	4070	3	64
Kisely	2021	Australia	Prospective Cohort	Psychological	Routinely Collected/Administrative Data	Lifetime	Anyone or Not-Specified	30	Any Substance Use Disorder	Incidence	physician diagnosis; diagnostic interview	30–30	Combined Men/Women			

Author	Year	Location	Study Design	Type of Violence	Exposure Assessment Method	Exposure Recall	Type of Perpetrator	Follow-up Years	Outcome	Type of Outcome	Outcome Assessment Method	Age Summary	Gender	Sample Size	Number of Cases (Exposed Group)	Number of Cases (Unexposed Group)
Harrington	2011	USA	Prospective Cohort	Witnessed Domestic Violence	Self-Report or Self-Administered Survey	Lifetime	Family Member or Caregiver	3	Drug Use	Incidence	self-report	18–99	Combined Men/Women	26935	164	
Najman	2022	Australia	Prospective Cohort	Psychological	Self-Report or Self-Administered Survey	Lifetime	Anyone or Not-Specified	30	Drug Use Disorder	Incidence	self-report	30–30	Combined Men/Women	2474		
Conroy	2009	Australia	Case-Control	Psychological	Self-Report or Self-Administered Survey	Lifetime	Anyone or Not-Specified		Opioid Dependency	Mortality	enrollment in a pharmacotherapy program as clinical determination of opioid dependence	35	Men	742	352	236
Conroy	2009	Australia	Case-Control	Psychological	Self-Report or Self-Administered Survey	Lifetime	Anyone or Not-Specified		Opioid Dependency	Mortality	enrollment in a pharmacotherapy program as clinical determination of opioid dependence	35	Women	571	227	152
Scheidell	2018	USA	Prospective Cohort	Psychological	Self-Report or Self-Administered Survey	Lifetime	Family Member or Caregiver	14	Cocaine Use In Emerging Adulthood	Incidence	self-report	18–26	Combined Men/Women	12288	182	680
Bertone-Johnson	2014	USA	Case-Control	Psychological	Self-Report or Self-Administered Survey	Lifetime	Family Member or Caregiver		Premenstrual Syndrome	Mortality	self-report; physician diagnosis	34	Women	3288	137	
Liebermann	2018	Multiple	Case-Control	Psychological	Self-Report or Self-Administered Survey	Lifetime	Anyone or Not-Specified		Endometriosis	Mortality	physician diagnosis	37.3 (7.3)	Women	842	185	
Ito	2021	Aichi	Case-Control	Psychological	Self-Report or Self-Administered Survey	Lifetime	Family Member or Caregiver		Premenstrual Syndrome (Pms)	Mortality	self-report	35.1 (10.3)	Women	3815		
Monnat	2015	USA	Retrospective Cohort	Psychological	Self-Report or Self-Administered Survey	Lifetime	Anyone or Not-Specified		Heart Attack	Incidence	self-report	43.9 (12.55)	Combined Men/Women	52250		
Kascakova	2022	Czechia	Retrospective Cohort	Psychological	Self-Report or Self-Administered Survey	Lifetime	Anyone or Not-Specified		Ischemic Heart Disease	Incident	self-report	46.2 (16.6)	Combined Men/Women	1018		
Kascakova	2022	Czechia	Retrospective Cohort	Psychological	Self-Report or Self-Administered Survey	Lifetime	Anyone or Not-Specified		Ischemic Heart Disease	Incident	self-report	46.4 (17.4)	Combined Men/Women	1800		
Dong	2004	California	Retrospective Cohort	Psychological	Self-Report or Self-Administered Survey	Lifetime	Family Member or Caregiver; Parent or Other Adult In The Household	2	Ischemic Heart Disease	Incidence	self-report	56 (15.2)	Combined Men/Women	17337	247	1582

Author	Year	Location	Study Design	Type of Violence	Exposure Assessment Method	Exposure Recall	Type of Perpetrator	Follow-up Years	Outcome	Type of Outcome	Outcome Assessment Method	Age Summary	Gender	Sample Size	Number of Cases (Exposed Group)	Number of Cases (Unexposed Group)
Karmakar	2017	USA	Prospective Cohort	Psychological	Self-Report or Self-Administered Survey	Lifetime	Family Member or Caregiver	14	Migraine Headaches	Incidence	self-report; physician diagnosis	24–32	Combined Men/Women	13493	1116	811
Kascakova	2022	Czechia	Retrospective Cohort	Psychological	Self-Report or Self-Administered Survey	Lifetime	Anyone or Not-Specified		Migraine	Incdient	self-report	46.2 (16.6)	Combined Men/Women	1018		
Kascakova	2022	Czechia	Retrospective Cohort	Psychological	Self-Report or Self-Administered Survey	Lifetime	Anyone or Not-Specified		Migraine	Incdient	self-report	46.4 (17.4)	Combined Men/Women	1800		
Brennenstuhl	2015	Canada	Retrospective Cohort	Witnessing Domestic Violence	Self-Report or Self-Administered Survey	Lifetime	Family Member or Caregiver		Migraine	Incidence	self-report; physician diagnosis	46.4 (17.3)	Men	10358		
Brennenstuhl	2015	Canada	Retrospective Cohort	Witnessing Domestic Violence	Self-Report or Self-Administered Survey	Lifetime	Family Member or Caregiver		Migraine	Incidence	self-report; physician diagnosis	47.5 (17.7)	Women	12638		
Murphy	2020	Denmark	Retrospective Cohort	Psychological	Self-Report or Self-Administered Survey ; Routinely Collected/Administrative Data	Lifetime	Anyone or Not Specified		Psychotic Disorders	Incidence	administrative medical records or disease registries	24 (0)	Combined Men/Women	2859		
Alkema	2023	Netherlands	Case-Control	Psychological	Self-Report or Self-Administered Survey	Lifetime	Anyone or Not-Specified		Schizophrenia	Mortality	physician diagnosis	18–99	Combined Men/Women	4037		
Abajobir	2017	Australia	Prospective Cohort	Psychological	Routinely Collected/Administrative Data	Lifetime	Anyone or Not-Specified	21	Dsm-Iv Type Psychosis (Past 12 Months)	Incidence	self-report	20.6	Combined Men/Women	2558		
Chatziioannidis	2019	Greece	Case-Control	Psychological	Self-Report or Self-Administered Survey	Lifetime	Father		Schizophrenia And Other Psychotic Disorders	Mortality	physician diagnosis	40 (10)	Combined Men/Women	124	18	
Trotta	2023	Multiple	Case-Control	Psychological	Self-Report or Self-Administered Survey	Lifetime	Family Member or Caregiver		Psychotic Disorder	Mortality	self-report	33.97 (12.58)	Combined Men/Women	2112	148	733
Kiselydmedres	2022	Australia	Prospective Cohort	Psychological	Self-Report or Self-Administered Survey ; Routinely Collected/Administrative Data	Lifetime	Anyone or Not-Specified	30	Any Self-Harm	Incidence	self-report	30–30	Combined Men/Women			
Roustit	2009	France	Retrospective Cohort	Witness Interparental Violence	Self-Report or Self-Administered Survey	Lifetime	Family Member or Caregiver		Lifetime Suicide Attempts	Incidence	self-report	18–99	Combined Men/Women	3023		
Mullen	1996	New Zealand	Retrospective Cohort	Psychological	Self-Report or Self-Administered Survey	Lifetime	Family Member or Caregiver; Parent		Ever Suicide Attempt	Incidence	self-report	18–65	Women	497		
Enns	2006	Netherlands	Prospective Cohort	Psychological	Self-Report or Self-Administered Survey	Lifetime	Anyone or Not-Specified	3	Suicidal Attempts	Incidence	self-report	18–64	Combined Men/Women	5670		

Author	Year	Location	Study Design	Type of Violence	Exposure Assessment Method	Exposure Recall	Type of Perpetrator	Follow-up Years	Outcome	Type of Outcome	Outcome Assessment Method	Age Summary	Gender	Sample Size	Number of Cases (Exposed Group)	Number of Cases (Unexposed Group)
Thompson	2019	USA	Prospective Cohort	Psychological	Self-Report or Self-Administered Survey	Lifetime	Parent or Adult Caregiver	13	Suicide Attempt	Incidence	self-report	15.03 (0.11)	Combined Men/Women	9421		
Russell	2019	Multiple	Prospective Cohort	Psychological	Self-Report or Self-Administered Survey	Lifetime	Anyone or Not-Specified		Self-Harm	Incidence	self-report	0–16	Combined Men/Women	4308		
Geng	2023	Shandong	Case-Control	Psychological	Self-Report or Self-Administered Survey	Lifetime	Anyone or Not-Specified		Self-Injury	Mortality	administrative medical records or disease registries; physician diagnosis	12–20	Combined Men/Women	414	77	61
Rajapakse	2020	Sri Lanka	Case-Control	Psychological	Self-Report or Self-Administered Survey	Lifetime	Family Member or Caregiver; Other Household Member		Self-Poisoning	Mortality	self-report; administrative medical records or disease registries	25	Combined Men/Women	686	12	

Section 3: GBD Outcome Definitions

Section 3.1: Standard GBD outcome definitions

The outcomes examined in this review were defined in accordance with the cause definitions used in the GBD study (Tables S3). We followed cause-specific research team guidance to accept GBD reference and alternate case definitions. For accepted alternate case definitions, we incorporated study-level bias covariates to detect if the use of an alternate definitions significantly biased final model results.

Table S4. Definitions of included outcomes.

Cause Grouping	Cause Name	Definition
**Mental health disorders**	Major depressive disorder	Major depressive disorder assessed according to DSM-4 (296.21–24, 296.31–34) and ICD-10 criteria (F32.0–9, F33.0–9). Diagnostic interviews and symptom scales were accepted according to the GBD criteria (Supplemental Table 4).
	Anxiety disorders	Anxiety disorders involving experiences of intense fear and distress in combination with other physiological symptoms.
	Schizophrenia	Schizophrenia is a chronic psychotic disorder that involves positive symptoms (eg, delusions, hallucinations) and negative symptoms (eg, flat affect, loss of interest). DSM-IV-TR (295.10-295.30, 295.60, 295.90) and ICD-10 (F20) criteria were used.
	Eating disorders	This aggregate cause incorporates deaths and disability from anorexia nervosa (AN) and bulimia nervosa (BN). These are characterised by abnormal eating behaviours and concerns over food, eating, and body image (DSM-IV-TR 307.1, 307.51; ICD-10 F50.0, F50.2).
**Substance use disorders**	Alcohol use disorder	A maladaptive pattern of substance use, leading to clinically significant impairment or distress, as manifested by three (or more) of the DSM-4 criteria for substance dependence occurring any time in a 12-month period.
	Drug use disorders	This aggregate cause incorporates death and disability resulting from opioid use disorder, amphetamine use disorder, cocaine use disorder, cannabis use disorder, and a residual category of other drug use disorders including deaths and disability due to dependence on hallucinogens, inhalants, solvents, and sedatives.

**Maternal disorders**	Maternal abortion and miscarriage	Abortion is defined as elective or medically indicated termination of pregnancy at any gestational age and miscarriage is defined as spontaneous loss of pregnancy before 24 weeks of gestation with complications requiring medical care.
**HIV/AIDS and Sexually Transmitted Infections**	Sexually transmitted infections excluding HIV	Sexually transmitted infections are viral, bacterial, or parasitic infections that are transmitted through sexual contact. This aggregate group includes syphilis, chlamydia, gonorrhea, genital herpes, trichomoniasis, and a residual category of other sexually transmitted infections including chancroid, granuloma inguinale, and unspecified sexually transmitted diseases.
**Injuries**	Self-harm	Self-harm is deliberate bodily damage inflicted on oneself resulting in death or injury (ICD-9: E950–E959, ICD-10: X60–X64.9, X66–X84.9, Y87.0).
**Headache disorders**	Migraine	Migraine is a disabling primary headache disorder, typically characterised by recurrent moderate or severe unilateral pulsatile headaches. The reference case definition is migraine in the last year satisfying the International Classification of Headache Disorders-3.
**Cardiovascular diseases**	Stroke	Stroke is defined according to WHO criteria of rapidly developed clinical signs of focal disturbance of cerebral function lasting > 24 hours or leading to death. Strokes are categorised as ischaemic, intracerebral, and subarachnoid events, depending on whether they are caused by a blockage or rupture of the blood vessels in the brain.
	Ischaemic Heart Disease	IHD is a disease of the coronary arteries, usually from atherosclerosis, leading to myocardial infraction or ischaemia, following the Fourth Universal Definition of Myocardial Infraction and, for stable angina, physician diagnosis. Incidence is estimated for any myocardial infraction.
**Diabetes and kidney diseases**	Diabetes mellitus	Diabetes mellitus is a chronic condition where either the pancreas does not produce enough insulin or the body is unable to metabolise insulin properly. Deaths and disability directly ascribed to diabetes are captured in the estimates for this cause. Other outcomes are captured under chronic kidney disease and risk estimates of elevated fasting plasma glucose.
**Chronic respiratory diseases**	Asthma	Asthma is a chronic lung disease characterised by reversible airway obstruction due to spasms and secretions in the bronchi usually resulting from an

		allergic reaction or hypersensitivity and causing difficulty in breathing.
**Other non-communicable diseases**	Gynaecological diseases	This aggregate cause incorporates death and disability resulting from uterine fibroids, polycystic ovarian syndrome, endometriosis, genital prolapse, premenstrual syndrome, menstrual disorders, and other gynaecological diseases (disorders of the breast, ovary, cervix, vagina, and vulva).

Section 3.2: Additional description of accepted definitions and measurement tools for depressive and anxiety disorders

For depressive and anxiety disorders specifically, we received an inventory of acceptable diagnostic interview and symptom scales from the mental health research team at IHME. These tools have been reviewed in terms of their validity and specificity to measuring the symptoms of these specific disorders. We included studies using accepted diagnostic interviews (reference) or symptom scales (accepted alternate). A list of acceptable tools is included in Table S4. For other mental disorders, we followed GBD case definitions and accepted studies measuring outcomes by use of International Disease Classification and Diagnostic and Statistical Manual of Mental Disorder criteria.

Table S5. Depressive and anxiety disorder accepted diagnostic interview and symptom scale measurement tools.

Measurement tool type	List of accepted tools
**Diagnostic interviews (reference method of measurement)**	- Structured Clinical Interview for DSM-IV Axis Disorders (SCID-I) (1 month, lifetime)<br>- Structured Clinical Interview for DSM-IV-TR Research Version, Non-patient edition (SCID-I/NP).<br>- Schedules for Clinical Assessment in Neuropsychiatry (SCAN)<br>- Mini International Neuropsychiatric Interview (MINI) (2 weeks, 2Y dysthymia)<br>- Mini International neuro-psychiatric Interview for children and Adolescents (MINI-KID)<br>- Diagnostic Interview Schedule (DIS) or Diagnostic Interview Schedule-IV (DIS-IV)<br>- Chinese modified Diagnostic Interview Schedule (DIS-CM)<br>- Composite International Diagnostic Interview (CIDI) (1 year, lifetime)<br>- University of Michigan Composite International Diagnostic Interview (UM-CIDI)<br>- Korean version of the Composite International Diagnostic Interview (K-CIDI)<br>- Munich-Composite International Diagnostic Interview (M-CIDI)<br>- Geriatric Mental State Schedule (GMS) (1 month) - AGECAT (Automated Geriatric Examination for Computer Assisted Taxonomy)

	- Primary Care Evaluation of Mental Disorders (PRIME-MD)<br>- Development and Well-Being Assessment (DAWBA)<br>- Schedule for Affective Disorders and Schizophrenia (SADS)<br>- Kiddie Schedule for Affective Disorders and Schizophrenia (K-SADS-PL)<br>- Comprehensive Psychopathological Rating Scale (CPRS)<br>- The Alcohol Use Disorder and Associated Disabilities Interview Schedule-IV (AUDADIS-IV) (1 year, lifetime)<br>- Diagnostic Interview for Children and Adolescents (DICA)<br>- SPIKE interview<br>- Clinical Interview Schedule-Revised (CIS-R) (1 week)<br>- Diagnostic Interview Schedule for Children Version IV (DISC)<br>- Diagnostic interview schedule for children-young child version (DISC-YC)<br>- Diagnostic Interview Schedule for Children, Parent Report (DISC-P)<br>- Present state examination (PSE)<br>- Child and adolescent psychiatric assessment (CAPA)<br>- Preschool age psychiatric assessment (PAPA)<br>- Children's Depression Rating Scale—Revised (CDRS-R)
**Symptom scales (alternate method of measurement)**	- Patient Health Questionnaire (PHQ)<br>- Revised Brief Patient Health Questionnaire (Brief PHQ-R)<br>- Beck Depression Inventory (BDI)<br>- Center for Epidemiologic Studies Depression Scale (CES-D)<br>- Center for Epidemiologic Studies Depression Scale for Children (CES-DC)<br>- Hospital Anxiety and Depression Scale (HADS)<br>- Depression Anxiety Stress Scale (DASS)<br>- Reynolds Adolescent Depression Scale (RADS)<br>- Child Depression Inventory (CDI)<br>- Duke Anxiety-Depression scale (DUKE-AD)<br>- Emotional State Questionnaire (EST-Q)<br>- Hopkins Symptom Checklist (HSCL) -DMI (25 items)<br>- Health & Daily Living Form (HDL)<br>- Child behavior checklist (CBCL)<br>- Hamilton depression rating scale (HAM-D)<br>- Harvard Department of Psychiatry National Depression Screening Day Scale (HANDS)<br>- Children's Depression Scale (CDS)<br>- Major Depression Inventory (MDI)<br>- Quick Inventory of Depressive Symptomology (QIDS)

Section 3.3: Additional description of accepted definitions for substance use disorders

For all the substance use disorders modeled in the GBD, the base reference case definitions are the DSM-4 criteria for substance dependence. Dependence is defined as a maladaptive pattern

of substance use, leading to clinically significant impairment or distress, as manifested by three (or more) of the following, occurring any time in a 12-month period:

- Tolerance, as defined by either of the following: (a) a need for markedly increased amounts of the substance to achieve intoxication or desired effect, or (b) markedly diminished effect with continued use of the same amount of the substance.
- Withdrawal, as manifested by either of the following: (a) the characteristic withdrawal syndrome for the substance, or (b) the same (or closely related) substance is taken to relieve or avoid withdrawal symptoms.
- The substance is often taken in larger amounts or over a longer period than intended.
- There is a persistent desire or unsuccessful efforts to cut down or control substance use.
- A great deal of time is spent in activities necessary to obtain the substance, use the substance, or recover from its effects.
- Important social, occupational, or recreational activities are given up or reduced because of substance use.
- The substance use is continued despite knowledge of having a persistent physical or psychological problem that is likely to have been caused or exacerbated by the substance (e.g., current cocaine use despite recognition of cocaine-induced depression, continued drinking despite recognition that an ulcer was made worse by alcohol consumption)

DSM-4 also provides the diagnostic criteria for substance abuse. The criteria for substance abuse focus on social and situational consequences of use and does not mention withdrawal or tolerance.

Section 4: Sensitivity Analyses

Section 4.1: Sensitivity analyses results for childhood violence exposures and outcomes

Table S6. Sensitivity analysis results for childhood violence exposure and major depressive disorder

Sensitivity analysis	% Trimming	Mean RR	95% UI for the mean RR without gamma	95% UI for the mean RR with gamma	BPRF	ROS	Star rating	Pub. bias	No. of studies (Obs.)	Selected bias covariates
**Neglect**										
Primary analysis	10%	1.60	1.33–1.92	0.74–3.45	0.84	-0.09	1	None	17 (27)	Risk of selection bias, Unadjusted for sex, Unadjusted for age, sex, and at least one other confounding variable, Males included in the effect size
	0%	1.82	1.40–2.38	0.50–6.68	0.61	-0.24	1	None	17 (27)	None
No SE adjustment applied	10%	1.62	1.36–1.94	0.76–3.47	0.86	-0.08	1	None	17 (27)	None
Any perpetrator analysis	10%	1.39	1.11–1.74	0.64–3.00	0.73	-0.16	1	None	9 (16)	Unadjusted for age, sex, and at least one other confounding variable
Perpetrator-specific analysis	10%	1.47	1.21–1.79	0.81–2.69	0.89	-0.06	1	None	8 (11)	Exposure defined including ages above 15
Female-only analysis	0%	1.35	0.87–2.09	0.35–5.19	0.43	NaN	0	None	4 (5)	None
Male-only analysis	0%	1.74	1.18–2.57	0.70–4.30	0.81	-0.1	1	None	3 (4)	None
Outcome sensitivity analysis - studies that refer to 'depression' broadly	10%	1.68	1.33–2.13	0.73–3.88	0.84	-0.09	1	None	11 (16)	Representativeness, Risk of selection bias, Family/household perpetrator
Outcome sensitivity analysis - studies that focus on major depressive disorder	10%	1.70	1.33–2.18	0.93–3.13	1.02	0.01	2	None	6 (11)	Unadjusted for age, Risk of reverse causation
**Physical abuse**										
Primary analysis	10%	1.54	1.42–1.67	1.09–2.16	1.16	0.072	2	None	26 (32)	None
	0%	1.63	1.46–1.82	0.93–2.86	1.02	0.01	2	None	26 (32)	Representativeness, Risk of reverse causation, Risk of selection bias, Unadjusted for age, Family/household perpetrator, Males included in the effect size

No SE adjustment applied	10%	1.54	1.42–1.67	1.09–2.16	1.16	0.072	2	None	26 (32)	Risk of reverse causation, Risk of selection bias, Unadjusted for sex, Family/household perpetrator
Any perpetrator analysis	10%	1.56	1.38–1.76	1.00–2.41	1.08	0.04	2	None	17 (22)	Unadjusted effect size
Perpetrator-specific analysis	0%	1.56	1.40–1.74	1.09–2.23	1.15	0.07	2	None	9 (10)	None
Female-only analysis	0%	2.22	1.98–2.49	1.69–2.90	1.77	0.29	3	None	7 (7)	None
Male-only analysis	0%	1.76	1.44–2.16	1.15–2.71	1.23	0.1	2	None	4 (4)	None
Outcome sensitivity analysis - studies that refer to 'depression' broadly	10%	1.57	1.40–1.76	1.01–2.44	1.08	0.04	2	None	17 (21)	None
Outcome sensitivity analysis - studies that focus on major depressive disorder	10%	1.65	1.51–1.81	1.34–2.04	1.39	0.16	3	None	10 (13)	None
**Psychological abuse**										
Primary analysis	10%	1.93	1.51–2.48	0.61–6.13	0.73	-0.15	1	None	17 (22)	Unadjusted for sex, Exposure is defined including ages above 15, Effect size for both sexes combined, Risk of reverse causation, Unadjusted for age, Family/household perpetrator, Effect size for both sexes combined
	0%	2.12	1.66–2.72	0.62–7.31	0.75	-0.14	1	None	17 (22)	Risk of reverse causation, Risk of selection bias, Unadjusted for sex, Unadjusted for age, Family/household perpetrator, Unadjusted for age, sex, and at least one other confounding variable
No SE adjustment applied	10%	1.93	1.51–2.48	0.61–6.13	0.73	-0.16	1	None	17 (22)	Risk of reverse causation, Risk of selection bias, Unadjusted for sex, Unadjusted for age, Family/household perpetrator, Exposure defined including ages above 15, Unadjusted for age, sex, and at least one other confounding variable
Any perpetrator analysis	10%	1.98	1.17–3.35	0.29–13.33	0.4	-0.46	1	None	9 (11)	Risk of reverse causation, Risk of selection bias, Outcome is defined including other disorders

Perpetrator-specific analysis	10%	1.64	1.58–1.71	1.51–1.78	1.53	0.21	3	None	8 (11)	Unadjusted for age, Males included in the effect size, Effect size for both sexes combined
Female-only analysis	0%	2.61	2.01–3.40	1.14–6.00	1.3	0.13	2	None	6 (6)	None
Male-only analysis	0%	1.92	1.13–3.24	0.36–10.11	0.47	-0.37	1	None	5 (5)	Representativeness
Outcome sensitivity analysis - studies that refer to 'depression' broadly	10%	1.84	1.35–2.51	0.48–7.00	0.6	-0.26	1	None	12 (15)	Risk of reverse causation, Risk of selection bias, Unadjusted for sex, Unadjusted for age, Family/household perpetrator, Exposure defined including ages above 15, Unadjusted for age, sex, and at least one other confounding variable
Outcome sensitivity analysis - studies that focus on major depressive disorder	0%	2.60	1.96–3.44	1.07–6.29	1.24	0.11	2	None	6 (9)	None

Table S7. Sensitivity analysis results for childhood violence exposure and drug use disorders

**Sensitivity analysis**	**% Trimming**	**Mean RR**	**95% UI for the mean RR without gamma**	**95% UI for the mean RR with gamma**	**BPRF**	**ROS**	**Star rating**	**Pub. bias**	**No. of studies (Obs.)**	**Selected bias covariates**
**Neglect**										
Primary analysis	0%	2.28	1.11–4.68	0.19–27.21	0.28	-0.63	1	None	5 (8)	Exposure defined as below age 15, Exposure defined including ages above 15, Representativeness, Risk of selection bias
No SE adjustment applied	0%	2.26	1.11–4.62	0.19–26.69	0.28	-0.63	1	None	5 (8)	Risk of selection bias, Exposure defined as below age 15, Males included in the effect size
Perpetrator-specific analysis	0%	1.50	1.02–2.20	0.47–4.77	0.57	-0.29	1	None	4 (6)	Exposure defined including ages above 15
Female-only analysis	0%	1.19	0.93–1.51	0.71–1.98	0.77	NaN	0	None	3 (3)	None
Male-only analysis	0%	8.66	0.30–250.78	0.00–192720.51	0	NaN	0	None	3 (3)	None
Outcome sensitivity analysis – use of non-marijuana illicit drugs	0%	1.50	1.02–2.20	0.47–4.77	0.57	-0.29	1	None	4 (6)	Exposure defined including ages above 15
**Physical abuse**										
Primary analysis	10%	1.40	1.24–1.58	0.96–2.03	1.02	0.01	2	None	11 (20)	Unadjusted for age, Unadjusted for age, sex, and at least one other confounding variable

	0%	1.50	1.19–1.89	0.57–3.93	0.67	-0.2	1	None	11 (20)	None
No SE adjustment applied	10%	1.39	1.24–1.56	0.98–1.98	1.04	0.02	2	None	11 (20)	Unadjusted for age, Unadjusted for age, sex, and at least one other confounding variable
Any perpetrator analysis	10%	1.45	1.25–1.68	1.00–2.11	1.06	0.03	2	None	6 (12)	Representativeness, Risk of selection bias, Males included in the effect size, Effect size for both sexes combined
Perpetrator-specific analysis	0%	1.52	1.14–2.01	0.63–3.66	0.72	-0.16	1	None	6 (10)	None
Female-only analysis	0%	1.43	1.09–1.87	0.76–2.70	0.84	-0.09	1	None	3 (4)	None
Male-only analysis	0%	1.93	1.18–3.15	0.47–7.85	0.59	-0.26	1	None	3 (4)	None
Outcome sensitivity analysis - cannabis dependence/use disorder	0%	1.17	0.67–2.02	0.26–5.19	0.33	NaN	0	None	3 (6)	None
Outcome sensitivity analysis - clinical drug dependence	0%	2.05	1.41–2.98	0.73–5.78	0.86	-0.08	1	None	3 (6)	None
**Psychological abuse**										
Primary analysis	10%	1.38	1.22–1.56	1.03–1.85	1.08	0.04	2	None	7 (10)	Risk of selection bias
	0%	1.53	1.25–1.87	0.77–3.02	0.86	-0.07	1	None	7 (10)	Risk of selection bias
Any perpetrator analysis	0%	1.37	1.05–1.80	0.68–2.77	0.76	-0.14	1	None	3 (5)	None
Perpetrator-specific analysis	0%	1.66	1.28–2.15	0.80–3.42	0.90	-0.05	1	None	4 (5)	Risk of selection bias
Outcome sensitivity analysis – use of non-marijuana illicit drugs	0%	2.05	1.71–2.45	1.36–3.08	1.46	0.19	3	None	4 (5)	None

Table S8. Sensitivity analysis results for childhood violence exposure and alcohol use disorders

Sensitivity analysis	% Trimming	Mean RR	95% UI for the mean RR without gamma	95% UI for the mean RR with gamma	BPRF	ROS	Star rating	Pub. bias	No. of studies (Obs.)	Selected bias covariates
**Neglect**										

Primary analysis	0%	1.84	0.82–4.15	0.11–29.67	0.18	NaN	0	None	5 (9)	Unadjusted for age, sex, and at least one other confounding variable
No SE adjustment applied	0%	2.43	0.97–6.08	0.13–45.85	0.21	NaN	0	None	4 (8)	None
Perpetrator-specific analysis	0%	2.43	0.61–9.60	0.04–139.10	0.08	NaN	0	None	3 (4)	None
Outcome sensitivity analysis - definition limited to clinical alcohol dependence	0%	2.43	0.97–6.08	0.13–45.85	0.21	NaN	0	None	4 (8)	None
**Physical abuse**										
Primary analysis	10%	1.54	1.33–1.79	0.96–2.46	1.04	0.02	2	None	8 (17)	Effect size for both sexes combined
	0%	1.64	1.37–1.95	0.90–2.97	0.99	0	1	None	8 (17)	Unadjusted for age, sex, and at least one other confounding variable, Effect size for both sexes combined
No SE adjustment applied	10%	1.60	1.39–1.84	1.04–2.47	1.11	0.052	2	None	8 (17)	Effect size for both sexes combined
Any perpetrator analysis	10%	1.45	1.16–1.81	0.77–2.74	0.85	-0.08	1	None	5 (11)	Unadjusted for age, sex, and at least one other confounding variable, Effect size for both sexes combined
Perpetrator-specific analysis	0%	1.70	1.51–1.92	1.33–2.18	1.38	0.16	3	None	4 (8)	None
Outcome sensitivity analysis - definition limited to alcohol abuse broadly	0%	1.15	0.94–1.40	0.72–1.82	0.78	NaN	0	None	4 (8)	None
Outcome sensitivity analysis - definition limited to clinical alcohol dependence	10%	1.7	1.53–1.88	1.34–2.15	1.39	0.17	3	None	7 (13)	None
**Psychological abuse**										
Primary analysis	0%	2.14	1.37–3.34	0.42–10.92	0.54	-0.31	1	None	7 (10)	Family/household perpetrator, Unadjusted for age, sex, and at least one other confounding variable, Males included in the effect size
No SE adjustment applied	0%	2.14	1.39–3.30	0.44–10.53	0.56	-0.29	1	None	7 (10)	Family/household perpetrator, Unadjusted for age, sex, and at least one other confounding variable,

										Males included in the effect size
Any perpetrator analysis	0%	1.91	0.90–4.07	0.21–17.04	0.3	NaN	0	None	3 (5)	None
Perpetrator-specific analysis	0%	2.22	1.43–3.44	0.61–8.09	0.75	-0.15	1	None	4 (5)	Effect size for both sexes combined
Outcome sensitivity analysis - definition limited to clinical alcohol dependence	0%	2.11	1.31–3.41	0.38–11.72	0.5	-0.35	1	None	6 (9)	Unadjusted for age, sex, and at least one other confounding variable

Table S9. Sensitivity analysis results for childhood violence exposure and eating disorders

Sensitivity analysis	% Trimming	Mean RR	95% UI for the mean RR without gamma	95% UI for the mean RR with gamma	BPRF	ROS	Star rating	Pub. bias	No. of studies (Obs.)	Selected bias covariates
**Physical abuse**										
Primary analysis	0%	2.13	1.46–3.13	0.91–5.02	1.04	0.02	2	None	4 (5)	None
No SE adjustment applied	0%	2.02	1.40–2.91	0.87–4.67	1	0	2	None	4 (5)	None
Female-only analysis	0%	2.28	1.53–3.41	0.96–5.43	1.1	0.05	2	None	3 (3)	None

Table S10. Sensitivity analysis results for childhood violence exposure and ischemic heart disease

Sensitivity analysis	% Trimming	Mean RR	95% UI for the mean RR without gamma	95% UI for the mean RR with gamma	BPRF	ROS	Star rating	Pub. bias	No. of studies (Obs.)	Selected bias covariates
**Physical abuse**										

Primary analysis	0%	1.45	1.28–1.65	1.10–1.92	1.15	0.07	2	None	4 (5)	None
Any perpetrator analysis	0%	1.36	1.08–1.70	0.83–2.21	0.9	-0.05	1	None	3 (4)	None
**Psychological abuse**										
Primary analysis	0%	1.69	0.98–2.93	0.34–8.32	0.44	NaN	0	None	3 (4)	None

Table S11. Sensitivity analysis results for childhood violence exposure and anxiety disorders

**Sensitivity analysis**	**% Trimming**	**Mean RR**	**95% UI for the mean RR without gamma**	**95% UI for the mean RR with gamma**	**BPRF**	**ROS**	**Star rating**	**Pub. bias**	**No. of studies (Obs.)**	**Selected bias covariates**
**Neglect**										
Primary analysis	10%	1.43	1.28–1.59	1.10–1.85	1.15	0.07	2	None	9 (14)	None
	0%	1.60	1.23–2.07	0.60–4.26	0.7	-0.18	1	None	9 (14)	None
No SE adjustment applied	10%	1.43	1.28–1.59	1.10–1.85	1.15	0.07	2	None	9 (14)	Representativeness, Unadjusted effect size
Any perpetrator analysis	0%	1.64	0.97–2.78	0.33–8.04	0.43	NaN	0	None	4 (6)	None
Perpetrator-specific analysis	0%	1.43	1.20–1.72	0.85–2.42	0.93	-0.04	1	None	5 (8)	None
Outcome sensitivity analysis - definition limited to only anxiety rather than PTSD	10%	1.39	1.24–1.56	1.07–1.81	1.11	0.05	2	None	8 (12)	None
**Physical abuse**										
Primary analysis	10%	1.26	1.14–1.39	0.93–1.70	0.98	-0.01	1	None	11 (15)	Family/household perpetrator, Exposure defined including ages above 15, Outcome is defined as PTSD
	0%	1.38	1.18–1.62	0.75–2.57	0.82	-0.1	1	None	11 (15)	Family/household perpetrator, Exposure defined including ages above 15
No SE adjustment applied	10%	1.26	1.14–1.39	0.93–1.70	0.98	-0.01	1	None	11 (15)	None
Any perpetrator analysis	10%	1.33	1.18–1.50	1.04–1.70	1.09	0.04	2	None	7 (11)	None
Perpetrator-specific analysis	0%	1.21	1.10–1.33	0.94–1.55	0.98	-0.01	1	None	4 (4)	None

Outcome sensitivity analysis - definition limited to only anxiety rather than PTSD	10%	1.19	1.11–1.28	0.98–1.45	1.01	0.01	2	None	10 (13)	Family/household perpetrator
**Psychological abuse**										
Primary analysis	0%	2.01	1.33–3.04	0.45–8.90	0.58	-0.27	1	None	7 (9)	None
No SE adjustment applied	0%	2.03	1.36–3.03	0.47–8.69	0.6	-0.26	1	None	7 (9)	Risk of selection bias, Unadjusted for age, Family/household perpetrator
Any perpetrator analysis	0%	2.26	1.20–4.25	0.29–17.63	0.4	-0.45	1	None	5 (7)	None
Outcome sensitivity analysis - definition limited to only anxiety rather than PTSD	0%	1.95	1.32–2.88	0.48–7.92	0.6	-0.26	1	None	7 (8)	Risk of selection bias, Unadjusted for age, Family/household perpetrator

Table S12. Sensitivity analysis results for childhood violence exposure and type 2 diabetes

**Sensitivity analysis**	**% Trimming**	**Mean RR**	**95% UI for the mean RR without gamma**	**95% UI for the mean RR with gamma**	**BPRF**	**ROS**	**Star rating**	**Pub. bias**	**No. of studies (Obs.)**	**Selected bias covariates**
**Neglect**										
Primary analysis	10%	1.07	0.93–1.23	0.72–1.60	0.76	NaN	0	None	6 (14)	None
	0%	1.18	0.92–1.51	0.51–2.71	0.58	NaN	0	None	6 (14)	None
No SE adjustment applied	10%	1.12	0.93–1.37	0.60–2.10	0.67	NaN	0	None	6 (14)	None
Any perpetrator analysis	0%	1.28	0.97–1.70	0.55–2.99	0.63	NaN	0	None	4 (9)	None
Perpetrator-specific analysis	0%	0.89	0.73–1.08	0.59–1.34	0.63	NaN	0	None	3 (7)	None
Female-only analysis	0%	1.07	0.93–1.23	0.74–1.53	0.79	NaN	0	None	3 (4)	None
Male-only analysis	0%	0.99	0.86–1.14	0.71–1.38	0.75	NaN	0	None	3 (4)	None
Outcome sensitivity analysis - definition limited to studies that refer to	10%	0.98	0.88–1.10	0.76–1.27	0.79	NaN	0	None	4 (11)	Unadjusted for age, sex, and at least one other confounding variable

‘diabetes’ broadly										
**Physical abuse**										
Primary analysis	10%	1.11	1.05–1.17	0.98–1.26	1	0	1	None	12 (21)	None
	0%	1.13	1.07–1.18	0.99–1.28	1.01	0.01	2	None	12 (21)	None
Any perpetrator analysis	10%	1.20	1.09–1.32	0.91–1.58	0.95	-0.02	1	None	8 (12)	None
Perpetrator-specific analysis	0%	1.08	1.02–1.16	0.95–1.24	0.97	-0.02	1	None	4 (9)	None
Female-only analysis	0%	1.13	1.02–1.25	0.84–1.53	0.88	-0.07	1	None	5 (8)	None
Male-only analysis	0%	1.16	1.00–1.34	0.84–1.60	0.88	-0.06	1	None	3 (4)	None
Outcome sensitivity analysis - definition limited to studies that refer to ‘diabetes’ broadly	10%	1.10	1.01–1.19	0.89–1.36	0.92	-0.04	1	None	8 (14)	Unadjusted for age, sex, and at least one other confounding variable, Unadjusted for sex
Outcome sensitivity analysis -limited to studies explicitly focused on type 2 diabetes	0%	1.20	1.07–1.34	0.89–1.62	0.93	-0.04	1	None	4 (7)	None
**Psychological abuse**										
Primary analysis	10%	1.13	1.05–1.23	0.91–1.41	0.95	-0.03	1	None	7 (13)	Unadjusted for sex
	0%	1.22	0.98–1.52	0.55–2.71	0.63	NaN	0	None	7 (13)	Unadjusted for sex
Any perpetrator analysis	0%	1.33	1.00–1.79	0.50–3.56	0.59	NaN	0	None	5 (7)	Unadjusted for sex
Female-only analysis	0%	1.16	1.07–1.26	0.98–1.38	1.01	0	2	None	3 (4)	None
Outcome sensitivity analysis - definition limited to studies that refer to	0%	1.29	0.92–1.81	0.41–4.08	0.49	NaN	0	None	5 (10)	Unadjusted for sex

‘diabetes’ broadly										

Table S13. Sensitivity analysis results for childhood violence exposure and self-harm

Sensitivity analysis	% Trimming	Mean RR	95% UI for the mean RR without gamma	95% UI for the mean RR with gamma	BPRF	ROS	Star rating	Pub. bias	No. of studies (Obs.)	Selected bias covariates
**Neglect**										
Primary analysis	0%	2.41	1.22–4.75	0.20–29.52	0.29	-0.61	1	None	7 (8)	Risk of reverse causation, Family/household perpetrator
No SE adjustment applied	0%	2.41	1.22–4.75	0.20–29.52	0.29	-0.61	1	None	7 (8)	Risk of reverse causation, Risk of selection bias, Exposure defined including ages above 15
Any perpetrator analysis	0%	3.30	1.66–6.56	0.32–33.63	0.47	-0.38	1	None	5 (6)	Risk of reverse causation
Perpetrator-specific analysis	0%	1.57	0.79–3.14	0.26–9.55	0.35	NaN	0	None	3 (4)	None
**Physical abuse**										
Primary analysis	10%	2	1.62–2.46	0.86–4.66	0.98	-0.01	1	None	16 (18)	Risk of reverse causation, Family/household perpetrator
	0%	2.50	1.88–3.32	0.65–9.58	0.81	-0.1	1	None	16 (18)	None
Any perpetrator analysis	10%	2.99	2.20–4.08	1.07–8.38	1.26	0.12	2	None	10 (11)	Unadjusted effect size, Unadjusted for age, sex, and at least one other confounding variable
Perpetrator-specific analysis	0%	1.61	1.38–1.89	1.08–2.39	1.16	0.07	2	None	6 (7)	Representativeness
**Psychological abuse**										
Primary analysis	0%	3.08	1.68–5.65	0.27–34.48	0.4	-0.45	1	None	8 (8)	Representativeness, Risk of reverse causation, Risk of selection bias, Unadjusted for age, Exposure defined including ages above 15
Any perpetrator analysis	0%	2.92	1.15–7.37	0.15–57.17	0.24	-0.71	1	None	4 (4)	Unadjusted for age, Exposure defined including ages above 15
Perpetrator-specific analysis	0%	4.66	1.94–11.23	0.23–93.03	0.38	-0.49	1	None	5 (5)	Unadjusted effect size, Risk of reverse causation

Table S14. Sensitivity analysis results for childhood violence exposure and asthma

Sensitivity analysis	% Trimming	Mean RR	95% UI for the mean RR without gamma	95% UI for the mean RR with gamma	BPRF	ROS	Star rating	Pub. bias	No. of studies (Obs.)	Selected bias covariates
**Neglect**										
Primary analysis	0%	1.47	1.17–1.86	0.80–2.72	0.88	-0.08	1	None	3 (7)	None
No SE adjustment applied	0%	1.54	1.19–1.99	0.76–3.11	0.85	-0.06	1	None	3 (7)	None
**Physical abuse**										
Primary analysis	0%	1.49	1.22–1.82	0.76–2.93	0.85	-0.08	1	None	6 (8)	Unadjusted for sex, Unadjusted for age
No SE adjustment applied	0%	1.49	1.22–1.82	0.76–2.92	0.84	-0.09	1	None	6 (8)	Unadjusted for age
Any perpetrator analysis	0%	1.54	1.21–1.96	0.70–3.40	0.79	-0.12	1	None	5 (7)	Unadjusted for sex
**Psychological abuse**										
Primary analysis	0%	2.01	1.36–2.99	0.59–6.83	0.72	-0.16	1	None	4 (5)	Unadjusted for age

Table S15. Sensitivity analysis results for childhood violence exposure and gynecological diseases

Sensitivity analysis	% Trimming	Mean RR	95% UI for the mean RR without gamma	95% UI for the mean RR with gamma	BPRF	ROS	Star rating	Pub. bias	No. of studies (Obs.)	Selected bias covariates
**Physical abuse**										
Primary analysis	0%	1.20	1.02–1.41	0.69–2.06	0.76	-0.14	1	None	6 (6)	Risk of selection bias, Family/household perpetrator
Any perpetrator analysis	0%	1.05	0.99–1.12	0.92–1.21	0.94	NaN	0	None	3 (3)	None
Perpetrator-specific analysis	0%	1.39	1.07–1.81	0.66–2.91	0.75	-0.15	1	None	3 (3)	None
**Psychological abuse**										

Primary analysis	0%	1.28	1.15–1.43	0.98–1.67	1.02	0.011	2	None	3 (3)	None

Table S16. Sensitivity analysis results for childhood violence exposure and maternal abortion and miscarriage

Sensitivity analysis	% Trimming	Mean RR	95% UI for the mean RR without gamma	95% UI for the mean RR with gamma	BPRF	ROS	Star rating	Pub. bias	No. of studies (Obs.)	Selected bias covariates
**Physical abuse**										
Primary analysis	0%	1.99	0.99–4.00	0.27–14.80	0.37	NaN	0	None	3 (6)	None
No SE adjustment applied	0%	1.93	0.96–3.88	0.26–14.55	0.36	NaN	0	None	3 (6)	None
Outcome sensitivity analysis - limited to cases of miscarriage	0%	1.75	1.03–2.97	0.41–7.47	0.52	-0.33	1	None	3 (5)	None

Table S17. Sensitivity analysis results for childhood violence exposure and sexually transmitted infections without HIV

Sensitivity analysis	% Trimming	Mean RR	95% UI for the mean RR without gamma	95% UI for the mean RR with gamma	BPRF	ROS	Star rating	Pub. bias	No. of studies (Obs.)	Selected bias covariates
**Neglect**										
Primary analysis	0%	1.13	0.93–1.39	0.71–1.81	0.76	NaN	0	None	3 (9)	None
No SE adjustment applied	0%	1.15	0.99–1.34	0.81–1.63	0.86	NaN	0	None	3 (9)	None
**Physical abuse**										
Primary analysis	0%	1.08	0.54–2.16	0.21–5.49	0.27	NaN	0	No	3 (5)	None
No SE adjustment applied	0%	1.26	0.68–2.35	0.28–5.62	0.36	NaN	0	No	3 (5)	None

Table S18. Sensitivity analysis results for childhood violence exposure and schizophrenia

Sensitivity analysis	% Trimming	Mean RR	95% UI for the mean RR without gamma	95% UI for the mean RR with gamma	BPRF	ROS	Star rating	Pub. bias	No. of studies (Obs.)	Selected bias covariates
**Neglect**										
Primary analysis	0%	2.49	1.08–5.72	0.22–28.78	0.32	-0.57	1	None	4 (5)	None
No SE adjustment applied	0%	2.41	1.09–5.35	0.23–24.95	0.34	-0.54	1	None	4 (5)	None
**Physical abuse**										
Primary analysis	0%	1.96	0.98–3.93	0.24–15.88	0.34	NaN	0	None	4 (6)	None
No SE adjustment applied	0%	2.07	1.03–4.13	0.25–17.19	0.35	-0.53	1	None	4 (6)	None
**Psychological abuse**										
Primary analysis	0%	2.68	1.71–4.22	0.75–9.64	0.92	-0.04	1	None	5 (6)	None
No SE adjustment applied	0%	2.66	1.68–4.21	0.72–9.83	0.89	-0.06	1	None	5 (6)	None
Any perpetrator analysis	0%	3.17	1.81–5.56	0.85–11.79	1.05	0.03	2	None	3 (3)	None

Section 4.2: Sensitivity analyses forest plots for childhood physical violence exposures and the corresponding outcomes

Figure S1. Sensitivity analysis results for childhood physical violence exposure (females only) and outcomes

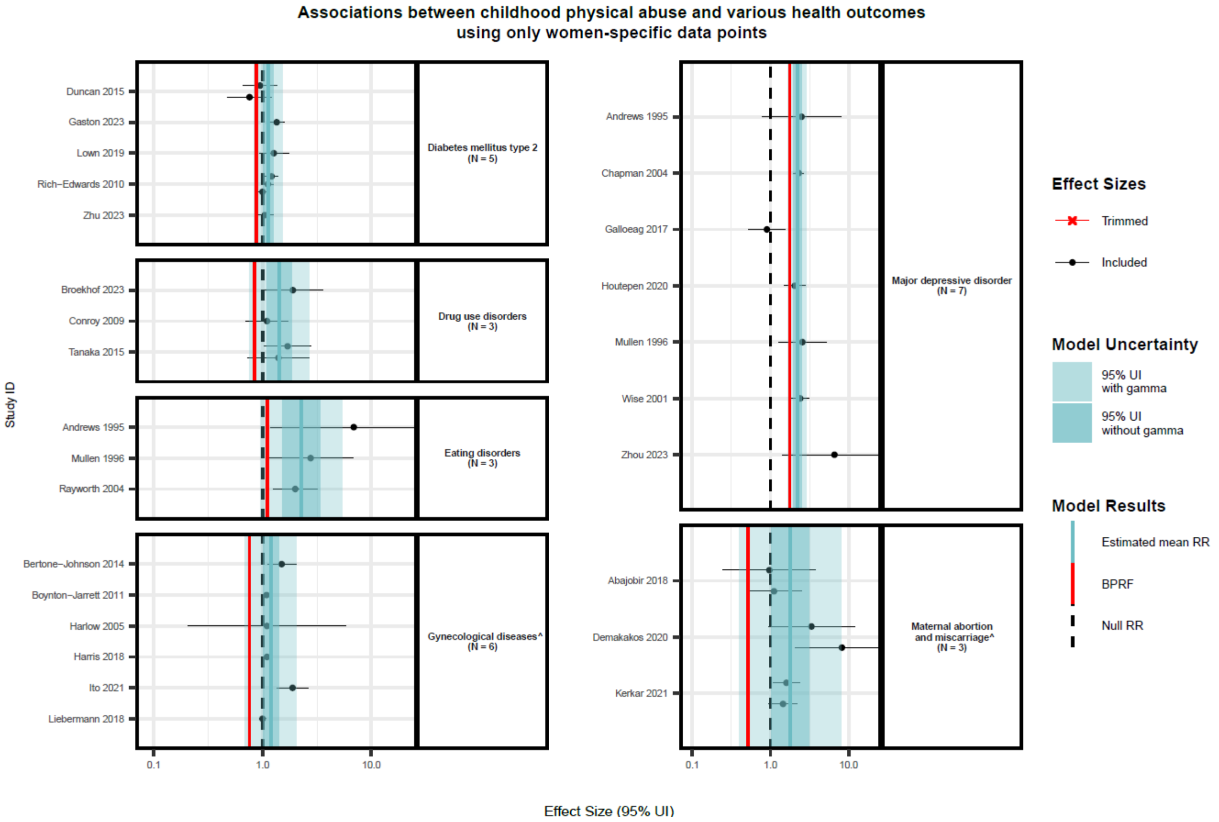

These forest plots present the estimated pooled relative risk, its 95% uncertainty intervals (UI), and the data points underlying the estimates for six health outcomes studied in association with childhood physical violence (females only). Each data point and horizontal line corresponds to a mean effect size and 95% UI from the included study identified on the y-axis. The color of the point indicates whether the point was detected and trimmed as an outlier. The light blue interval corresponds to the 95% UI of the pooled relative risk when incorporating between-study heterogeneity; the dark blue interval corresponds to the 95% UI of the pooled relative risk without between-study heterogeneity. The black vertical dotted line reflects the null relative risk value (one) and the red vertical line is the burden of proof function at the 5th quantile for this harmful risk-outcome association. We truncated the x-axis to make the scale more legible, so a handful of 95% UIs from the included studies extend beyond the plot margin. We included multiple observations from a single study when effects were reported by severity/frequency of the violence exposure, by different types of violent acts, and/or separately by sex or other subgroups.

Figure S2. Sensitivity analysis results for childhood physical violence exposure (males only) and outcomes

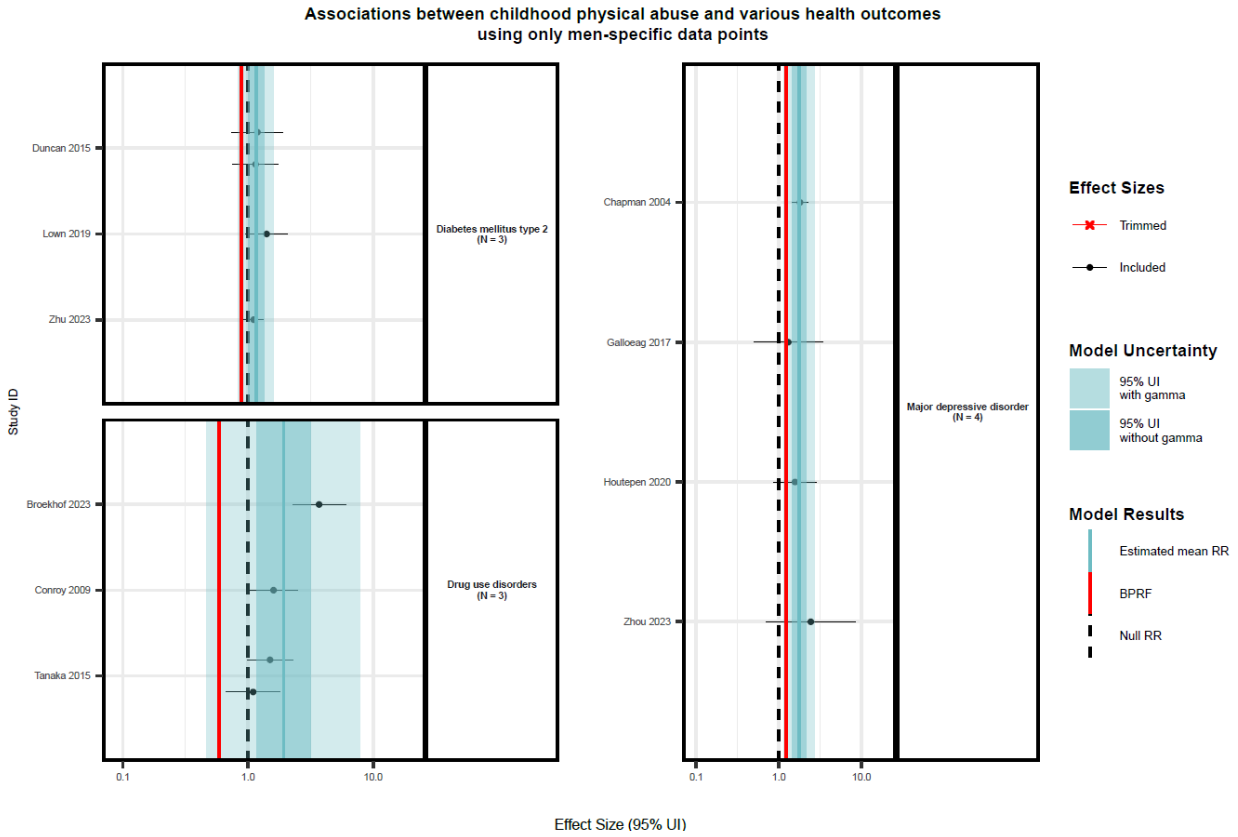

These forest plots present the estimated pooled relative risk, its 95% uncertainty intervals (UI), and the data points underlying the estimates for three health outcomes studied in association with childhood physical violence (males only). Each data point and horizontal line corresponds to a mean effect size and 95% UI from the included study identified on the y-axis. The color of the point indicates whether the point was detected and trimmed as an outlier. The light blue interval corresponds to the 95% UI of the pooled relative risk when incorporating between-study heterogeneity; the dark blue interval corresponds to the 95% UI of the pooled relative risk without between-study heterogeneity. The black vertical dotted line reflects the null relative risk value (one) and the red vertical line is the burden of proof function at the 5th quantile for this harmful risk-outcome association. We truncated the x-axis to make the scale more legible, so a handful of 95% UIs from the included studies extend beyond the plot margin. We included multiple observations from a single study when effects were reported by severity/frequency of the violence exposure, by different types of violent acts, and/or separately by sex or other subgroups.

Figure S3. Sensitivity analysis results for perpetrator-specific childhood physical violence exposure and outcomes

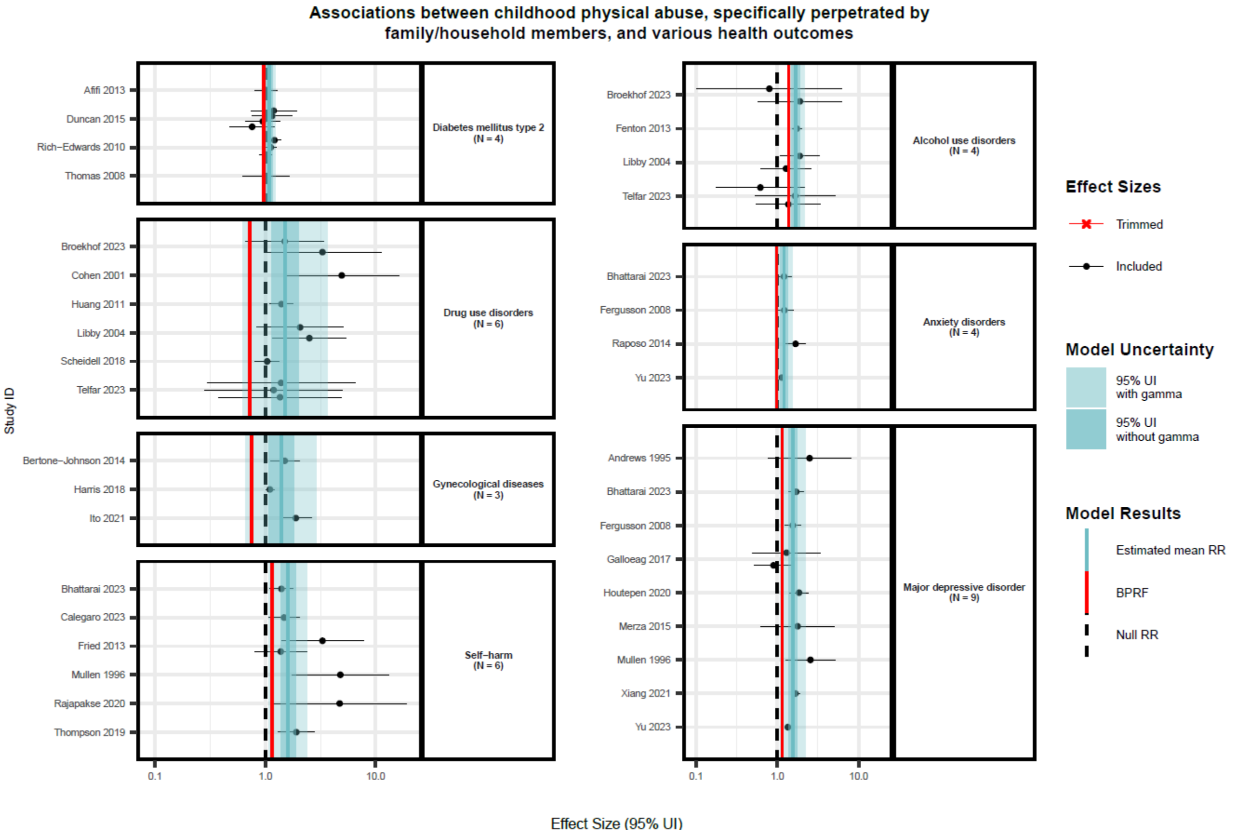

These forest plots present the estimated pooled relative risk, its 95% uncertainty intervals (UI), and the data points underlying the estimates for seven health outcomes studied in association with childhood physical violence perpetrated by family and/or household members. Each data point and horizontal line corresponds to a mean effect size and 95% UI from the included study identified on the y-axis. The color of the point indicates whether the point was detected and trimmed as an outlier. The light blue interval corresponds to the 95% UI of the pooled relative risk when incorporating between-study heterogeneity; the dark blue interval corresponds to the 95% UI of the pooled relative risk without between-study heterogeneity. The black vertical dotted line reflects the null relative risk value (one) and the red vertical line is the burden of proof function at the 5th quantile for this harmful risk-outcome association. We truncated the x-axis to make the scale more legible, so a handful of 95% UIs from the included studies extend beyond the plot margin. We included multiple observations from a single study when effects were reported by severity/frequency of the violence exposure, by different types of violent acts, and/or separately by sex or other subgroups.

Figure S4. Sensitivity analysis results for childhood physical violence exposure by any perpetrator and outcomes

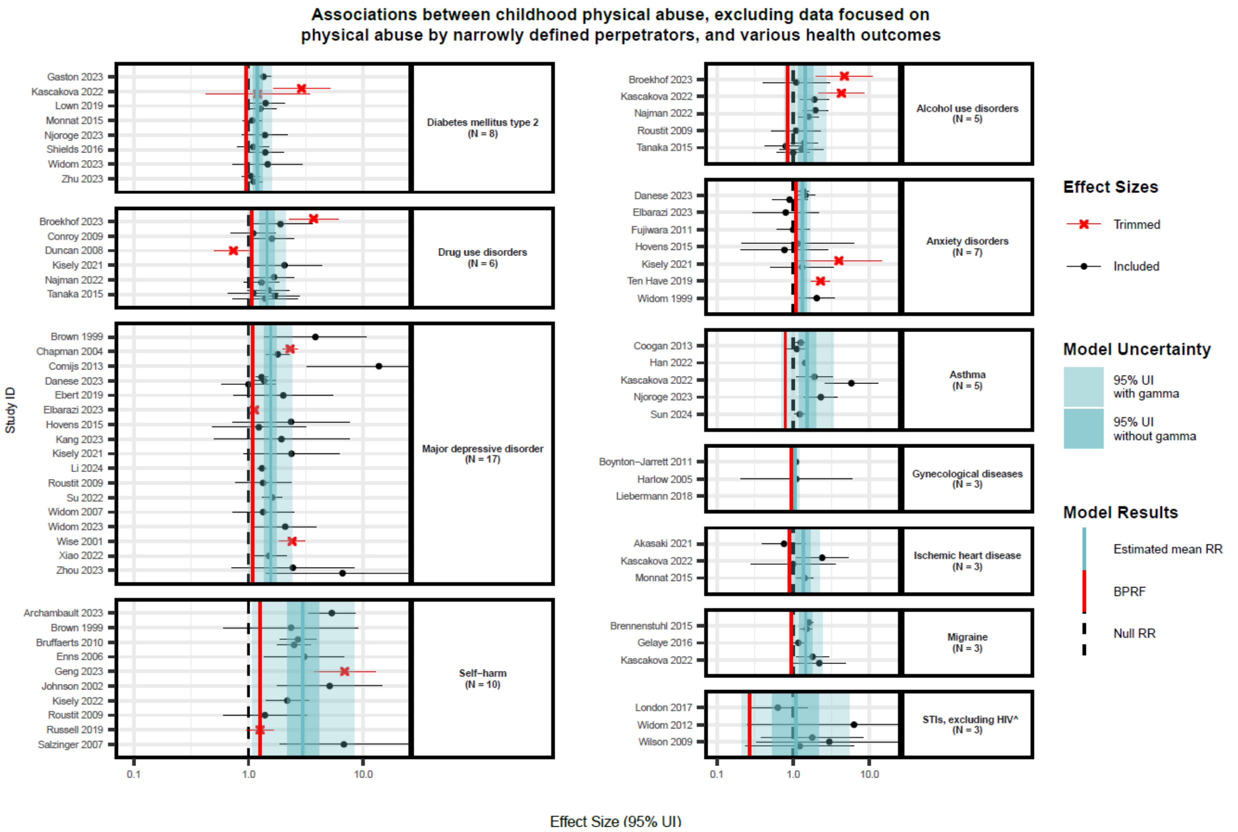

These forest plots present the estimated pooled relative risk, its 95% uncertainty intervals (UI), and the data points underlying the estimates for 11 health outcomes studied in association with childhood physical violence with unspecified perpetrators. Each data point and horizontal line corresponds to a mean effect size and 95% UI from the included study identified on the y-axis. The color of the point indicates whether the point was detected and trimmed as an outlier. The light blue interval corresponds to the 95% UI of the pooled relative risk when incorporating between-study heterogeneity; the dark blue interval corresponds to the 95% UI of the pooled relative risk without between-study heterogeneity. The black vertical dotted line reflects the null relative risk value (one) and the red vertical line is the burden of proof function at the 5th quantile for this harmful risk-outcome association. We truncated the x-axis to make the scale more legible, so a handful of 95% UIs from the included studies extend beyond the plot margin. We included multiple observations from a single study when effects were reported by severity/frequency of the violence exposure, by different types of violent acts, and/or separately by sex or other subgroups.

Figure S5. Sensitivity analysis results for childhood physical violence exposure and alternative outcome definitions

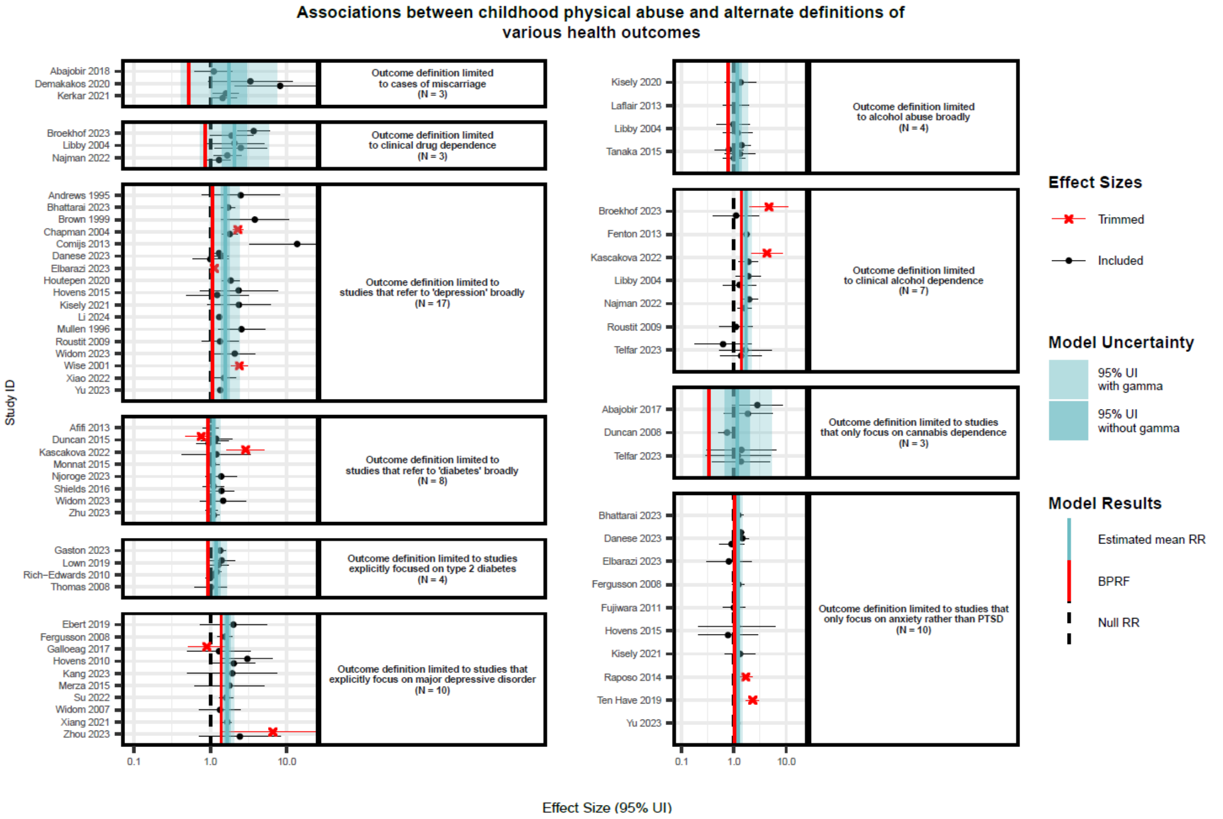

These forest plots present the estimated pooled relative risk, its 95% uncertainty intervals (UI), and the data points underlying the estimates for 10 health outcomes studied in association with childhood physical violence. Each data point and horizontal line corresponds to a mean effect size and 95% UI from the included study identified on the y-axis. The color of the point indicates whether the point was detected and trimmed as an outlier. The light blue interval corresponds to the 95% UI of the pooled relative risk when incorporating between-study heterogeneity; the dark blue interval corresponds to the 95% UI of the pooled relative risk without between-study heterogeneity. The black vertical dotted line reflects the null relative risk value (one) and the red vertical line is the burden of proof function at the 5th quantile for this harmful risk-outcome association. We truncated the x-axis to make the scale more legible, so a handful of 95% UIs from the included studies extend beyond the plot margin. We included multiple observations from a single study when effects were reported by severity/frequency of the violence exposure, by different types of violent acts, and/or separately by sex or other subgroups.

Figure S6. Sensitivity analysis results for childhood physical violence exposure (no adjustment) and outcomes

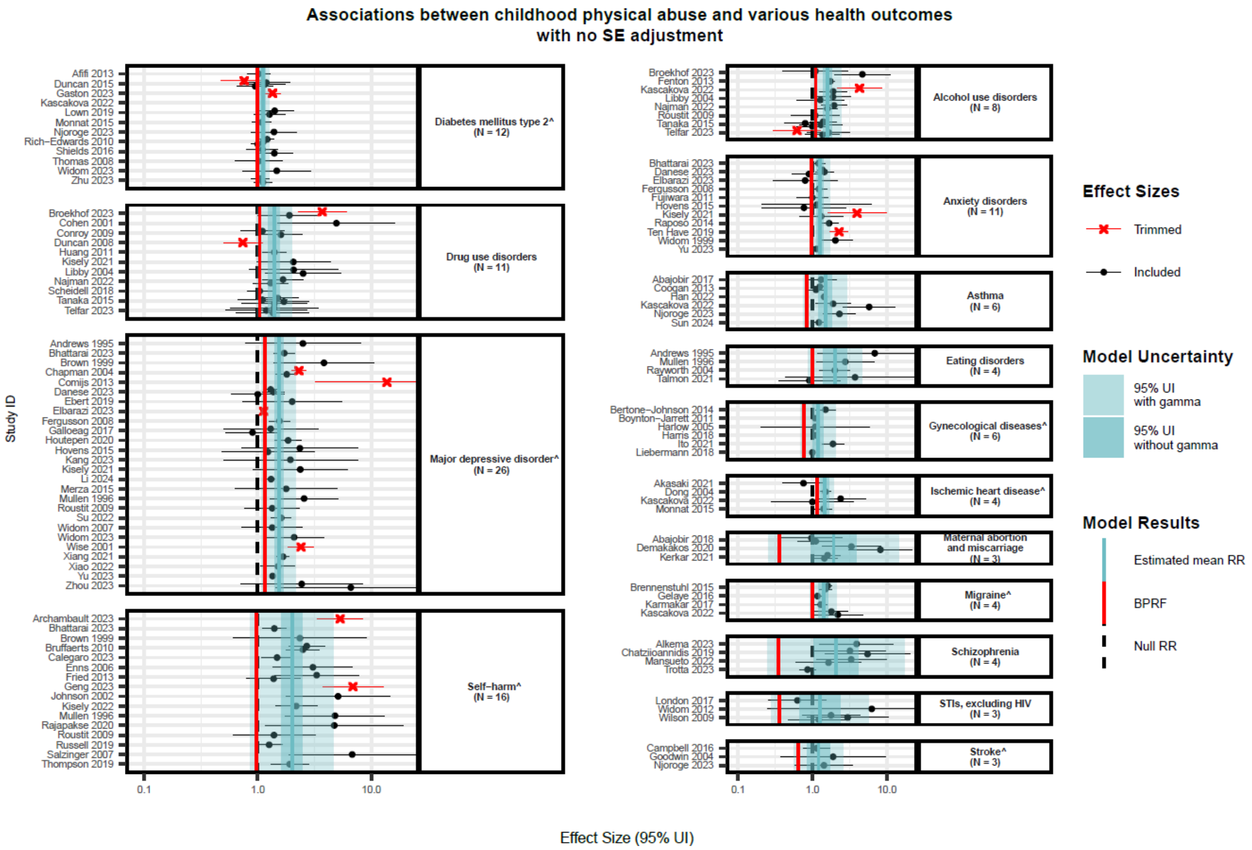

These forest plots present the estimated pooled relative risk, its 95% uncertainty intervals (UI), and the data points underlying the estimates for 15 health outcomes studied in association with childhood physical violence. Each data point and horizontal line corresponds to a mean effect size and 95% UI from the included study identified on the y-axis. The color of the point indicates whether the point was detected and trimmed as an outlier. The light blue interval corresponds to the 95% UI of the pooled relative risk when incorporating between-study heterogeneity; the dark blue interval corresponds to the 95% UI of the pooled relative risk without between-study heterogeneity. The black vertical dotted line reflects the null relative risk value (one) and the red vertical line is the burden of proof function at the 5th quantile for this harmful risk-outcome association. We truncated the x-axis to make the scale more legible, so a handful of 95% UIs from the included studies extend beyond the plot margin. We included multiple observations from a single study when effects were reported by severity/frequency of the violence exposure, by different types of violent acts, and/or separately by sex or other subgroups.

Figure S7. Sensitivity analysis results for childhood physical violence exposure (no trimming) and outcomes

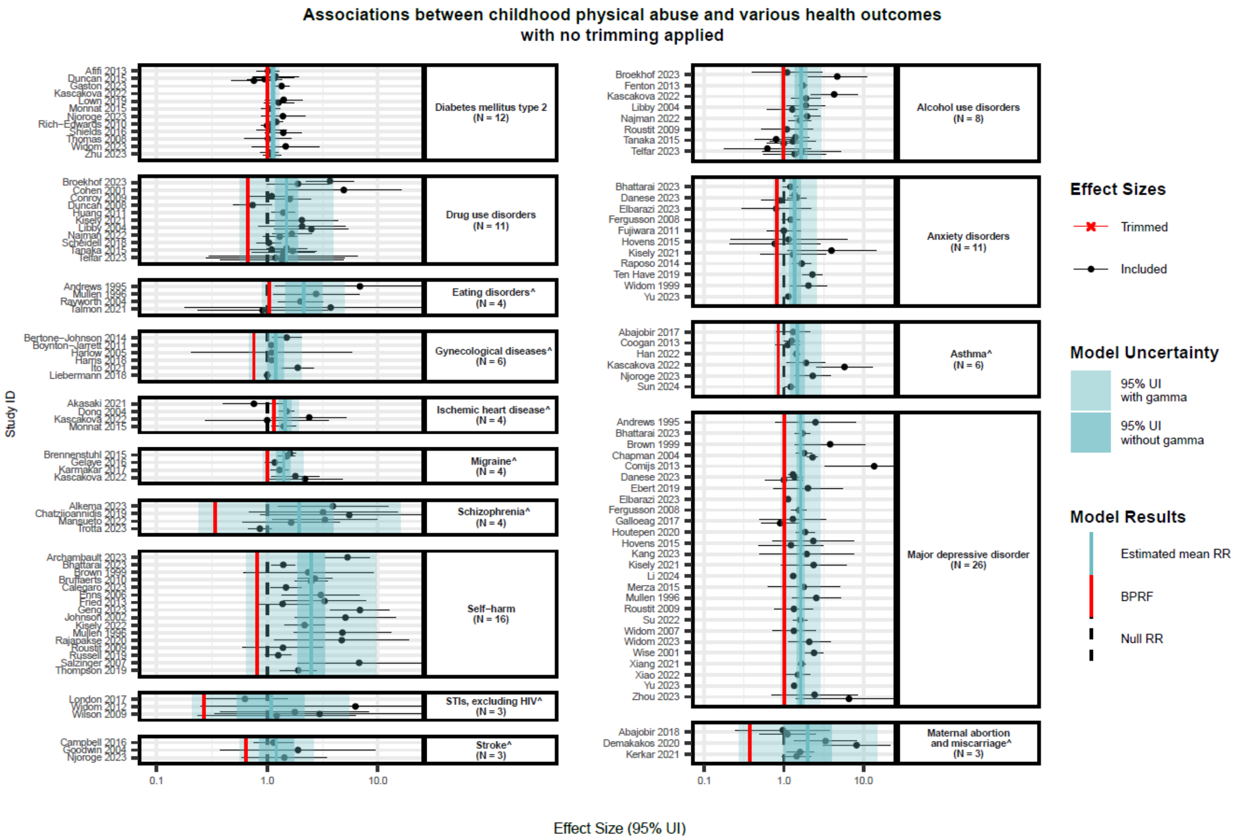

These forest plots present the estimated pooled relative risk, its 95% uncertainty intervals (UI), and the data points underlying the estimates for 15 health outcomes studied in association with childhood physical violence. Each data point and horizontal line corresponds to a mean effect size and 95% UI from the included study identified on the y-axis. The color of the point indicates whether the point was detected and trimmed as an outlier. The light blue interval corresponds to the 95% UI of the pooled relative risk when incorporating between-study heterogeneity; the dark blue interval corresponds to the 95% UI of the pooled relative risk without between-study heterogeneity. The black vertical dotted line reflects the null relative risk value (one) and the red vertical line is the burden of proof function at the 5th quantile for this harmful risk-outcome association. We truncated the x-axis to make the scale more legible, so a handful of 95% UIs from the included studies extend beyond the plot margin. We included multiple observations from a single study when effects were reported by severity/frequency of the violence exposure, by different types of violent acts, and/or separately by sex or other subgroups.

Section 4.3: Sensitivity analyses forest plots for psychological childhood violence exposures and corresponding outcomes

Figure S8. Sensitivity analysis results for childhood psychological violence exposure and alternative outcome definitions

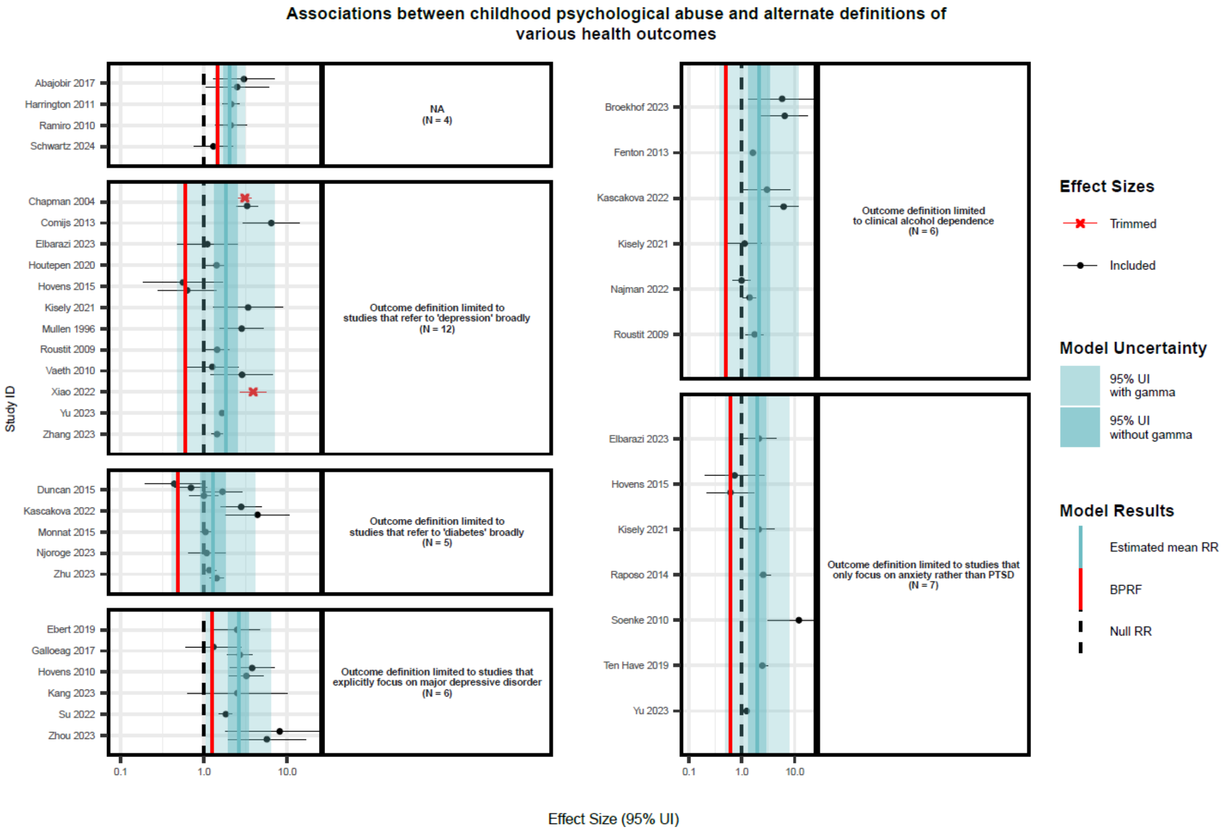

These forest plots present the estimated pooled relative risk, its 95% uncertainty intervals (UI), and the data points underlying the estimates for six health outcomes studied in association with childhood psychological violence. Each data point and horizontal line corresponds to a mean effect size and 95% UI from the included study identified on the y-axis. The color of the point indicates whether the point was detected and trimmed as an outlier. The light blue interval corresponds to the 95% UI of the pooled relative risk when incorporating between-study heterogeneity; the dark blue interval corresponds to the 95% UI of the pooled relative risk without between-study heterogeneity. The black vertical dotted line reflects the null relative risk value (one) and the red vertical line is the burden of proof function at the 5th quantile for this harmful risk-outcome association. We truncated the x-axis to make the scale more legible, so a handful of 95% UIs from the included studies extend beyond the plot margin. We included multiple observations from a single study when effects were reported by severity/frequency of the violence exposure, by different types of violent acts, and/or separately by sex or other subgroups.

Figure S9. Sensitivity analysis results for childhood psychological violence exposure (females only) and outcomes

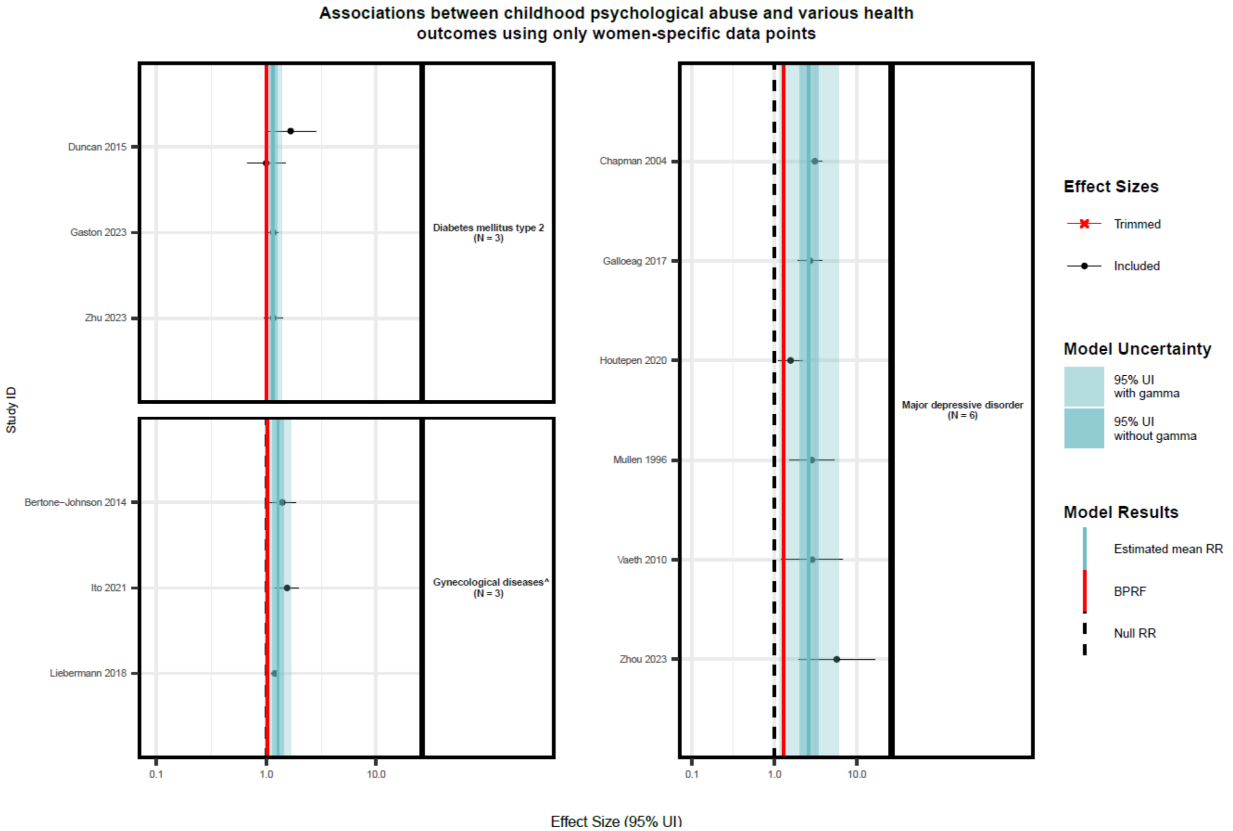

These forest plots present the estimated pooled relative risk, its 95% uncertainty intervals (UI), and the data points underlying the estimates for three health outcomes studied in association with childhood psychological violence (females only). Each data point and horizontal line corresponds to a mean effect size and 95% UI from the included study identified on the y-axis. The color of the point indicates whether the point was detected and trimmed as an outlier. The light blue interval corresponds to the 95% UI of the pooled relative risk when incorporating between-study heterogeneity; the dark blue interval corresponds to the 95% UI of the pooled relative risk without between-study heterogeneity. The black vertical dotted line reflects the null relative risk value (one) and the red vertical line is the burden of proof function at the 5th quantile for this harmful risk-outcome association. We truncated the x-axis to make the scale more legible, so a handful of 95% UIs from the included studies extend beyond the plot margin. We included multiple observations from a single study when effects were reported by severity/frequency of the violence exposure, by different types of violent acts, and/or separately by sex or other subgroups.

Figure S10. Sensitivity analysis results for childhood psychological violence exposure (males only) and outcomes

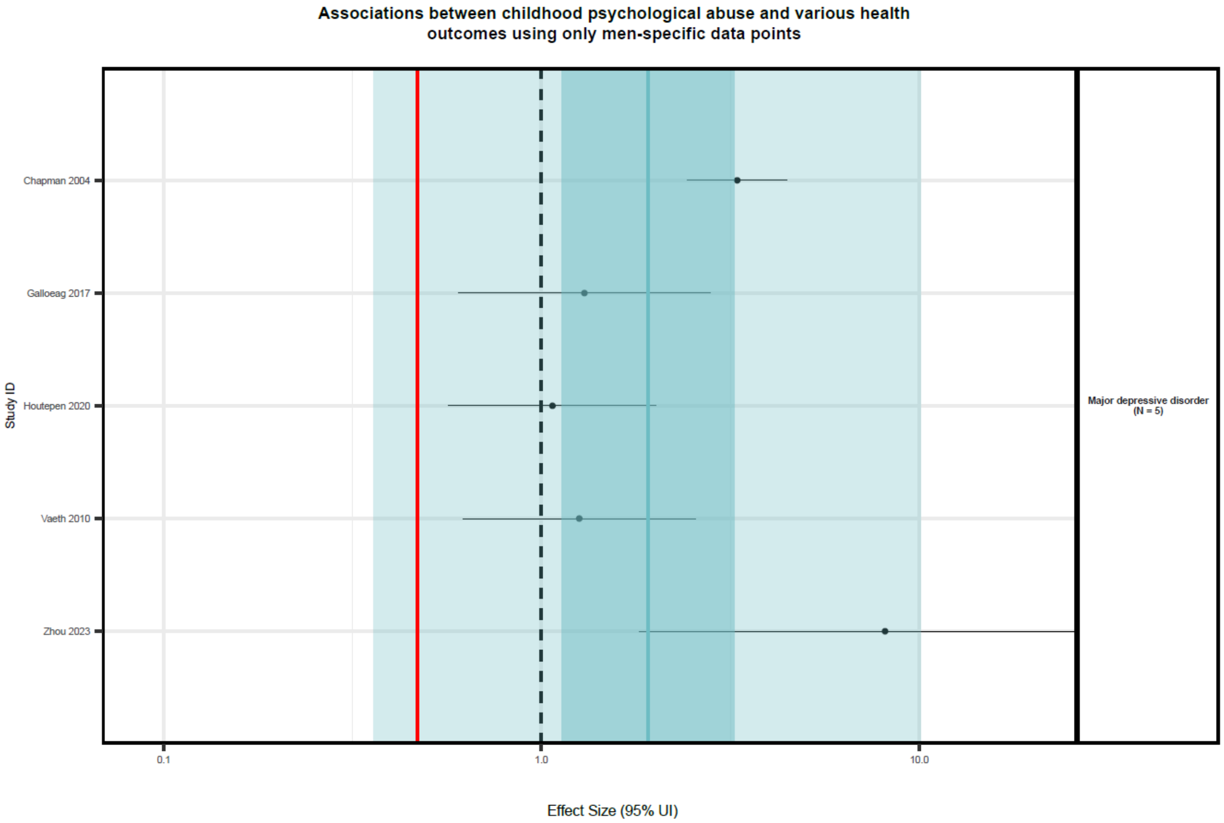

These forest plots present the estimated pooled relative risk, its 95% uncertainty intervals (UI), and the data points underlying the estimates for one health outcome studied in association with childhood psychological violence (males only). Each data point and horizontal line corresponds to a mean effect size and 95% UI from the included study identified on the y-axis. The color of the point indicates whether the point was detected and trimmed as an outlier. The light blue interval corresponds to the 95% UI of the pooled relative risk when incorporating between-study heterogeneity; the dark blue interval corresponds to the 95% UI of the pooled relative risk without between-study heterogeneity. The black vertical dotted line reflects the null relative risk value (one) and the red vertical line is the burden of proof function at the 5th quantile for this harmful risk-outcome association. We truncated the x-axis to make the scale more legible, so a handful of 95% UIs from the included studies extend beyond the plot margin. We included multiple observations from a single study when effects were reported by severity/frequency of the violence exposure, by different types of violent acts, and/or separately by sex or other subgroups.

Figure S11. Sensitivity analysis results for perpetrator-specific childhood psychological violence exposure and outcomes

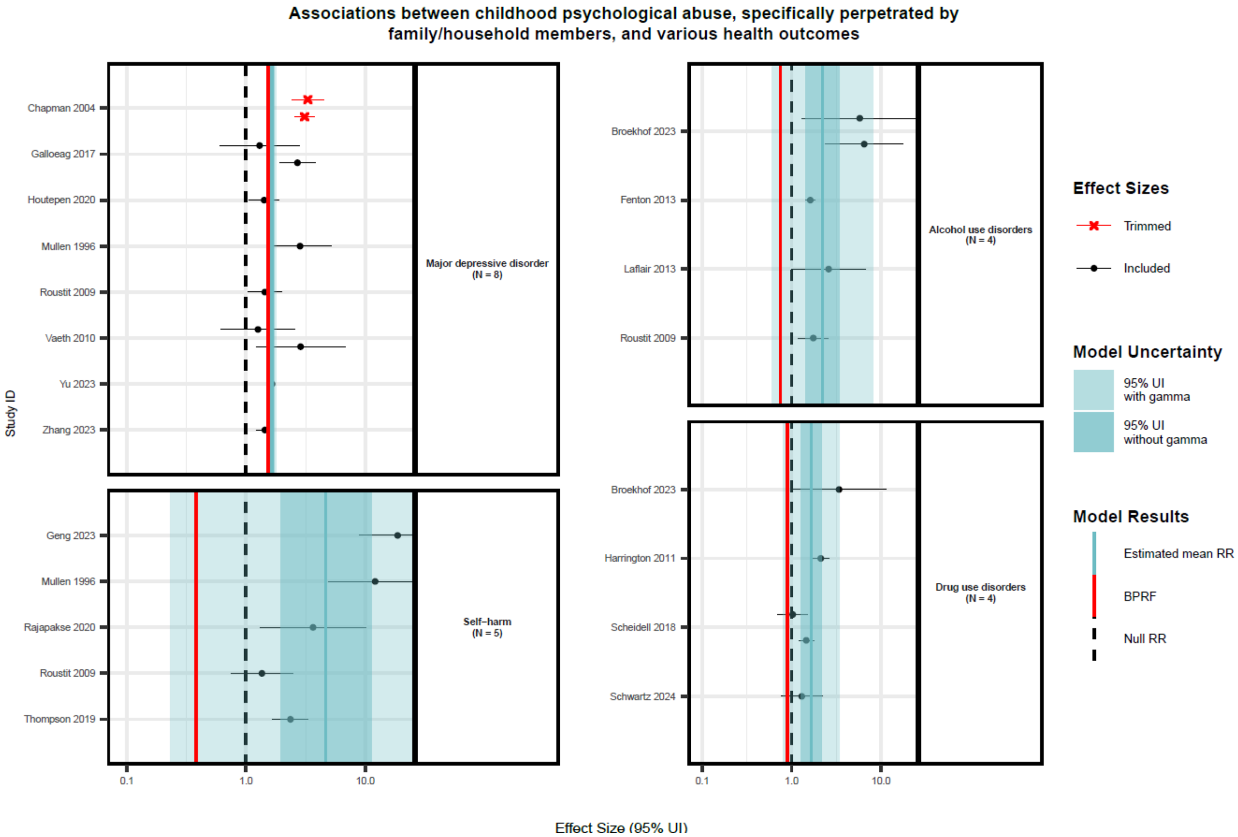

These forest plots present the estimated pooled relative risk, its 95% uncertainty intervals (UI), and the data points underlying the estimates for four health outcomes studied in association with childhood psychological violence perpetrated by a family/household member. Each data point and horizontal line corresponds to a mean effect size and 95% UI from the included study identified on the y-axis. The color of the point indicates whether the point was detected and trimmed as an outlier. The light blue interval corresponds to the 95% UI of the pooled relative risk when incorporating between-study heterogeneity; the dark blue interval corresponds to the 95% UI of the pooled relative risk without between-study heterogeneity. The black vertical dotted line reflects the null relative risk value (one) and the red vertical line is the burden of proof function at the 5th quantile for this harmful risk-outcome association. We truncated the x-axis to make the scale more legible, so a handful of 95% UIs from the included studies extend beyond the plot margin. We included multiple observations from a single study when effects were reported by severity/frequency of the violence exposure, by different types of violent acts, and/or separately by sex or other subgroups.

Figure S12. Sensitivity analysis results for childhood psychological violence exposure by any perpetrator and outcomes

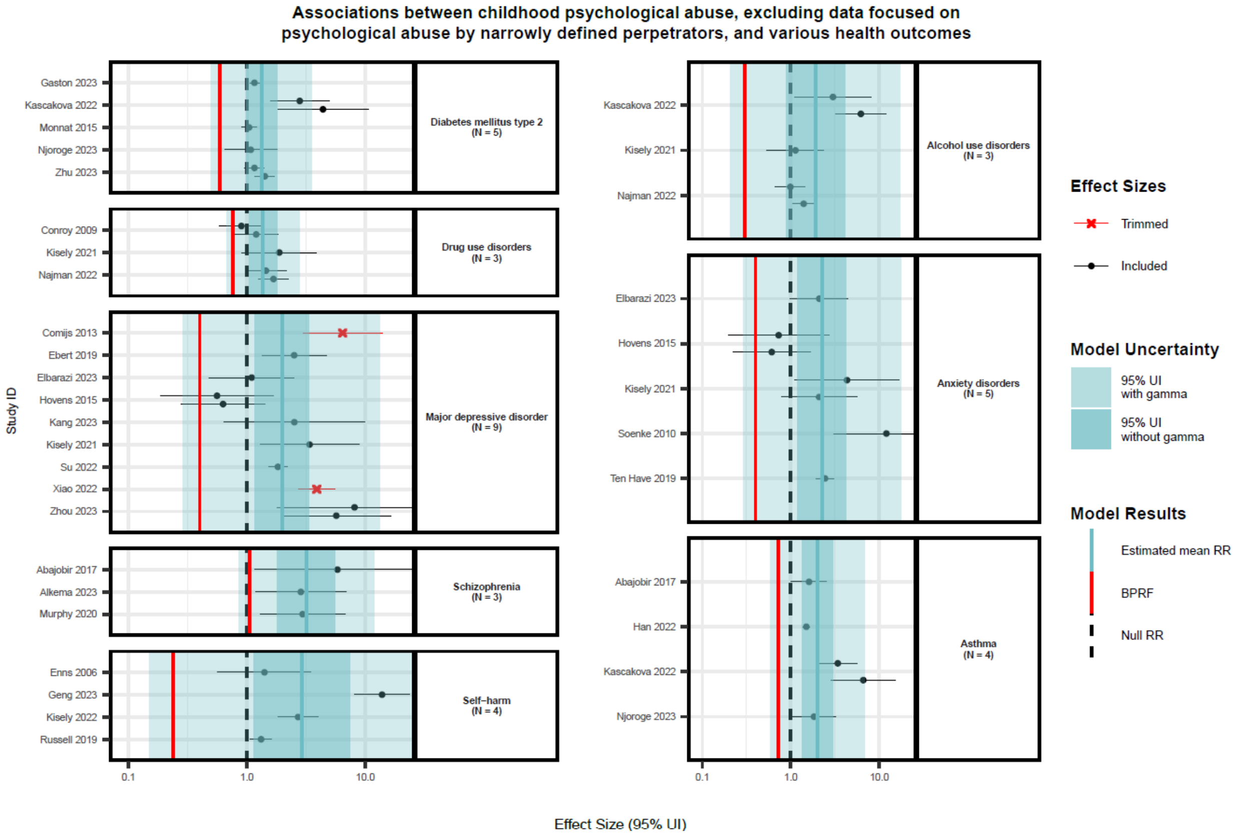

These forest plots present the estimated pooled relative risk, its 95% uncertainty intervals (UI), and the data points underlying the estimates for eight health outcomes studied in association with childhood psychological violence with unspecified perpetrators. Each data point and horizontal line corresponds to a mean effect size and 95% UI from the included study identified on the y-axis. The color of the point indicates whether the point was detected and trimmed as an outlier. The light blue interval corresponds to the 95% UI of the pooled relative risk when incorporating between-study heterogeneity; the dark blue interval corresponds to the 95% UI of the pooled relative risk without between-study heterogeneity. The black vertical dotted line reflects the null relative risk value (one) and the red vertical line is the burden of proof function at the 5th quantile for this harmful risk-outcome association. We truncated the x-axis to make the scale more legible, so a handful of 95% UIs from the included studies extend beyond the plot margin. We included multiple observations from a single study when effects were reported by severity/frequency of the violence exposure, by different types of violent acts, and/or separately by sex or other subgroups.

Figure S13. Sensitivity analysis results for childhood psychological violence exposure (no adjustment) and outcomes

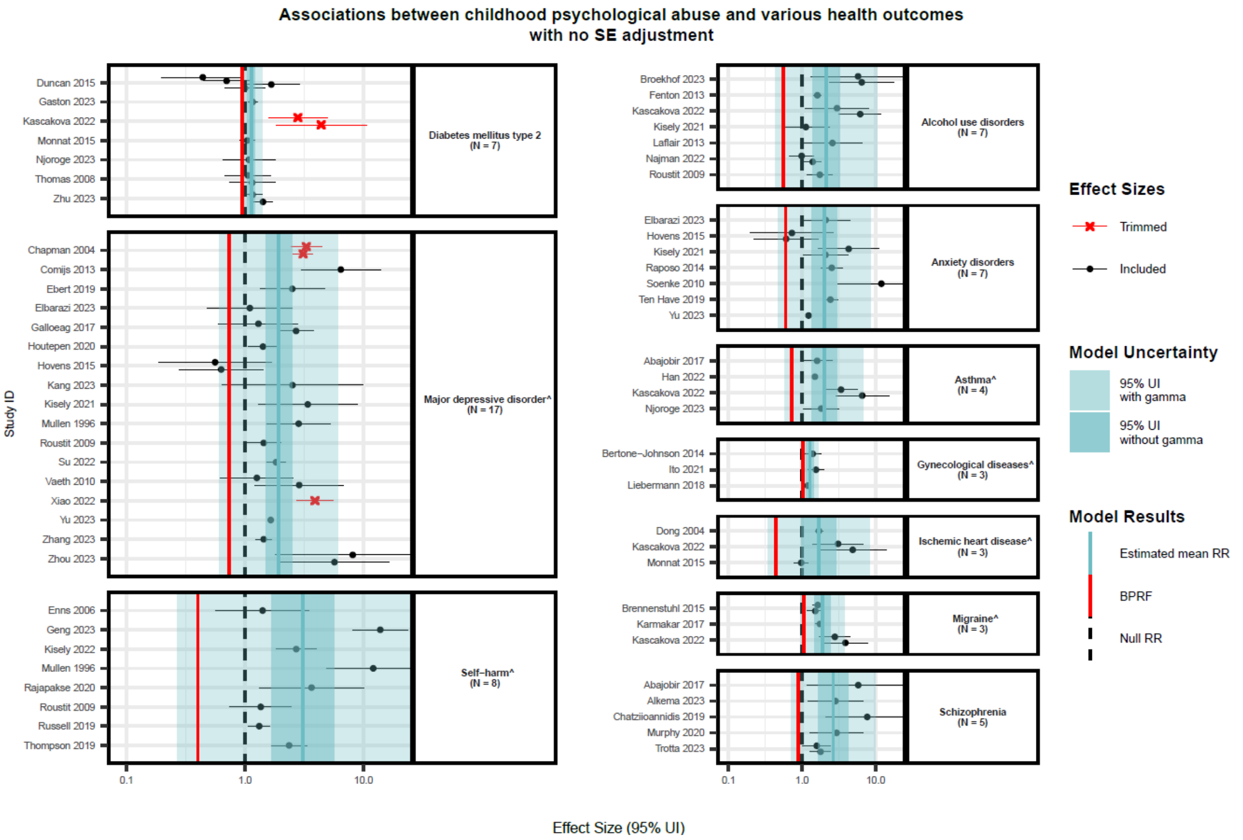

These forest plots present the estimated pooled relative risk, its 95% uncertainty intervals (UI), and the data points underlying the estimates for 10 health outcomes studied in association with childhood psychological violence. Each data point and horizontal line corresponds to a mean effect size and 95% UI from the included study identified on the y-axis. The color of the point indicates whether the point was detected and trimmed as an outlier. The light blue interval corresponds to the 95% UI of the pooled relative risk when incorporating between-study heterogeneity; the dark blue interval corresponds to the 95% UI of the pooled relative risk without between-study heterogeneity. The black vertical dotted line reflects the null relative risk value (one) and the red vertical line is the burden of proof function at the 5th quantile for this harmful risk-outcome association. We truncated the x-axis to make the scale more legible, so a handful of 95% UIs from the included studies extend beyond the plot margin. We included multiple observations from a single study when effects were reported by severity/frequency of the violence exposure, by different types of violent acts, and/or separately by sex or other subgroups.

Figure S14. Sensitivity analysis results for childhood psychological violence exposure (no trimming) and outcomes

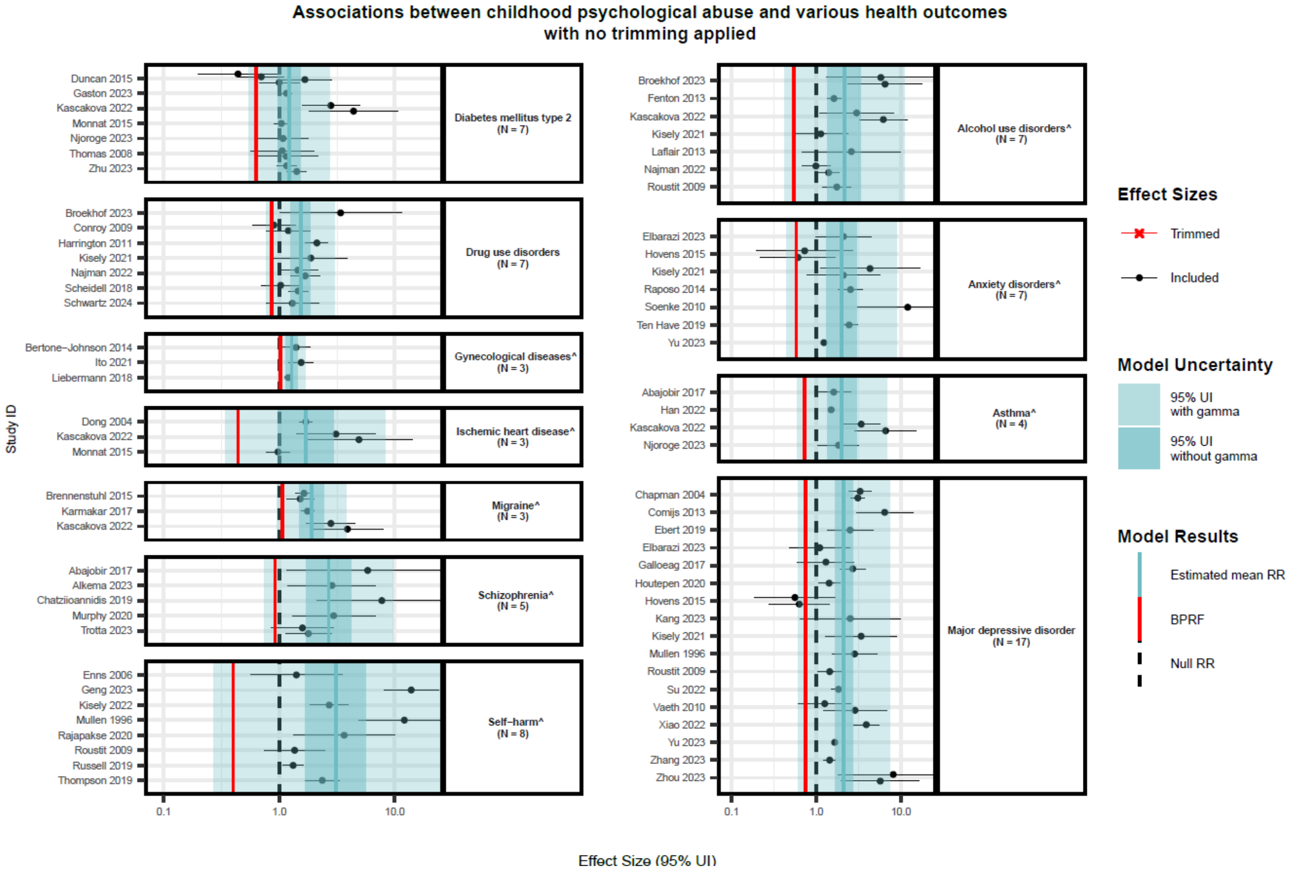

These forest plots present the estimated pooled relative risk, its 95% uncertainty intervals (UI), and the data points underlying the estimates for 11 health outcomes studied in association with childhood psychological violence. Each data point and horizontal line corresponds to a mean effect size and 95% UI from the included study identified on the y-axis. The color of the point indicates whether the point was detected and trimmed as an outlier. The light blue interval corresponds to the 95% UI of the pooled relative risk when incorporating between-study heterogeneity; the dark blue interval corresponds to the 95% UI of the pooled relative risk without between-study heterogeneity. The black vertical dotted line reflects the null relative risk value (one) and the red vertical line is the burden of proof function at the 5th quantile for this harmful risk-outcome association. We truncated the x-axis to make the scale more legible, so a handful of 95% UIs from the included studies extend beyond the plot margin. We included multiple observations from a single study when effects were reported by severity/frequency of the violence exposure, by different types of violent acts, and/or separately by sex or other subgroups.

Section 4.4: Sensitivity analyses forest plots for childhood neglect exposures and corresponding outcomes

Figure S15. Sensitivity analysis results for childhood neglect (females only) and outcomes

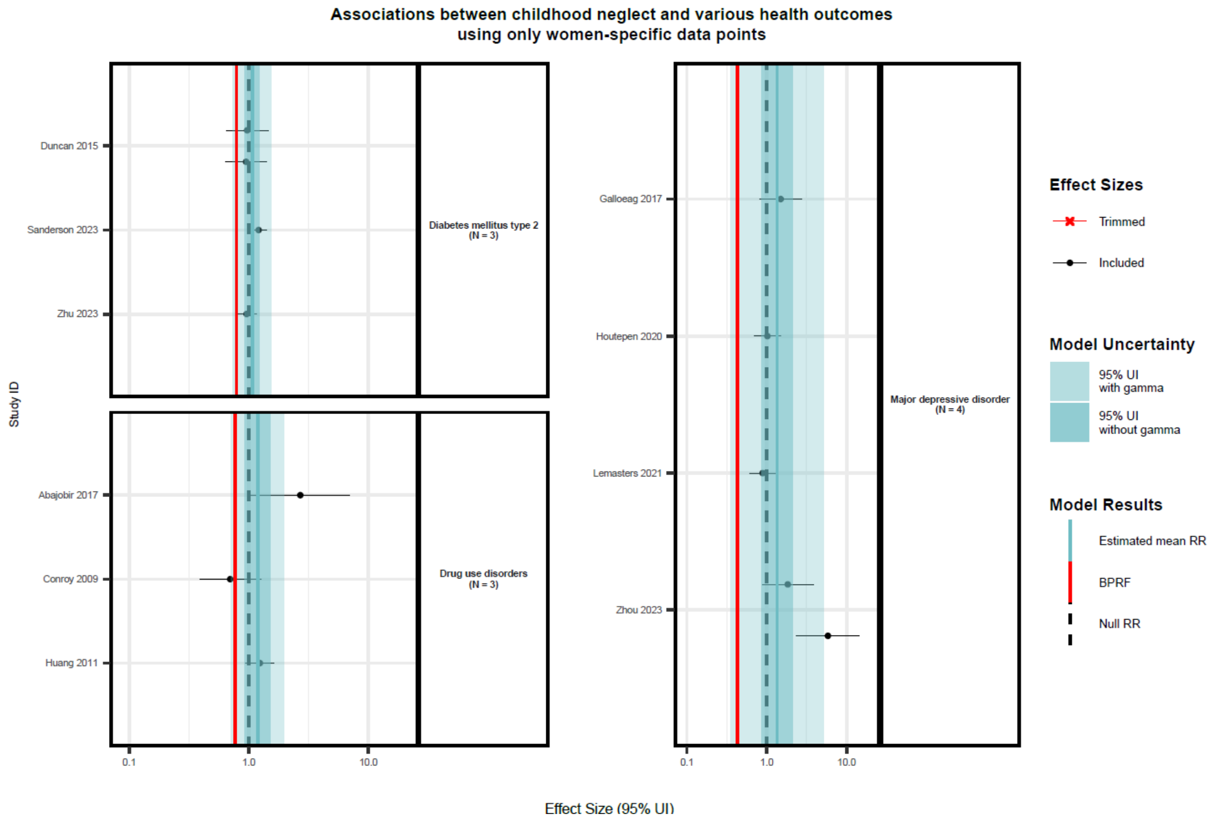

These forest plots present the estimated pooled relative risk, its 95% uncertainty intervals (UI), and the data points underlying the estimates for three health outcomes studied in association with childhood neglect (women only). Each data point and horizontal line corresponds to a mean effect size and 95% UI from the included study identified on the y-axis. The color of the point indicates whether the point was detected and trimmed as an outlier. The light blue interval corresponds to the 95% UI of the pooled relative risk when incorporating between-study heterogeneity; the dark blue interval corresponds to the 95% UI of the pooled relative risk without between-study heterogeneity. The black vertical dotted line reflects the null relative risk value (one) and the red vertical line is the burden of proof function at the 5th quantile for this harmful risk-outcome association. We truncated the x-axis to make the scale more legible, so a handful of 95% UIs from the included studies extend beyond the plot margin. We included multiple observations from a single study when effects were reported by severity/frequency of the violence exposure, by different types of violent acts, and/or separately by sex or other subgroups.

Figure S16. Sensitivity analysis results for childhood neglect (males only) and outcomes

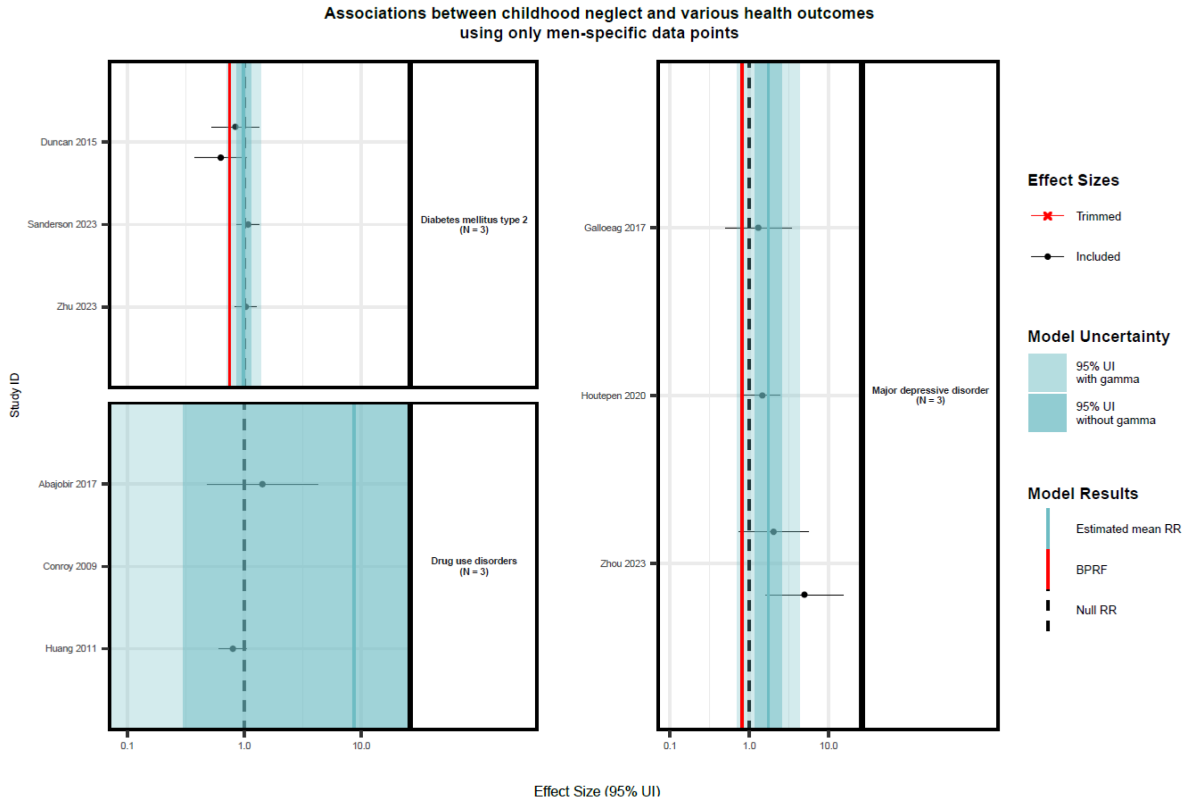

These forest plots present the estimated pooled relative risk, its 95% uncertainty intervals (UI), and the data points underlying the estimates for three health outcomes studied in association with childhood neglect (male only). Each data point and horizontal line corresponds to a mean effect size and 95% UI from the included study identified on the y-axis. The color of the point indicates whether the point was detected and trimmed as an outlier. The light blue interval corresponds to the 95% UI of the pooled relative risk when incorporating between-study heterogeneity; the dark blue interval corresponds to the 95% UI of the pooled relative risk without between-study heterogeneity. The black vertical dotted line reflects the null relative risk value (one) and the red vertical line is the burden of proof function at the 5th quantile for this harmful risk-outcome association. We truncated the x-axis to make the scale more legible, so a handful of 95% UIs from the included studies extend beyond the plot margin. We included multiple observations from a single study when effects were reported by severity/frequency of the violence exposure, by different types of violent acts, and/or separately by sex or other subgroups.

Figure S17. Sensitivity analysis results for perpetrator-specific childhood neglect and outcomes

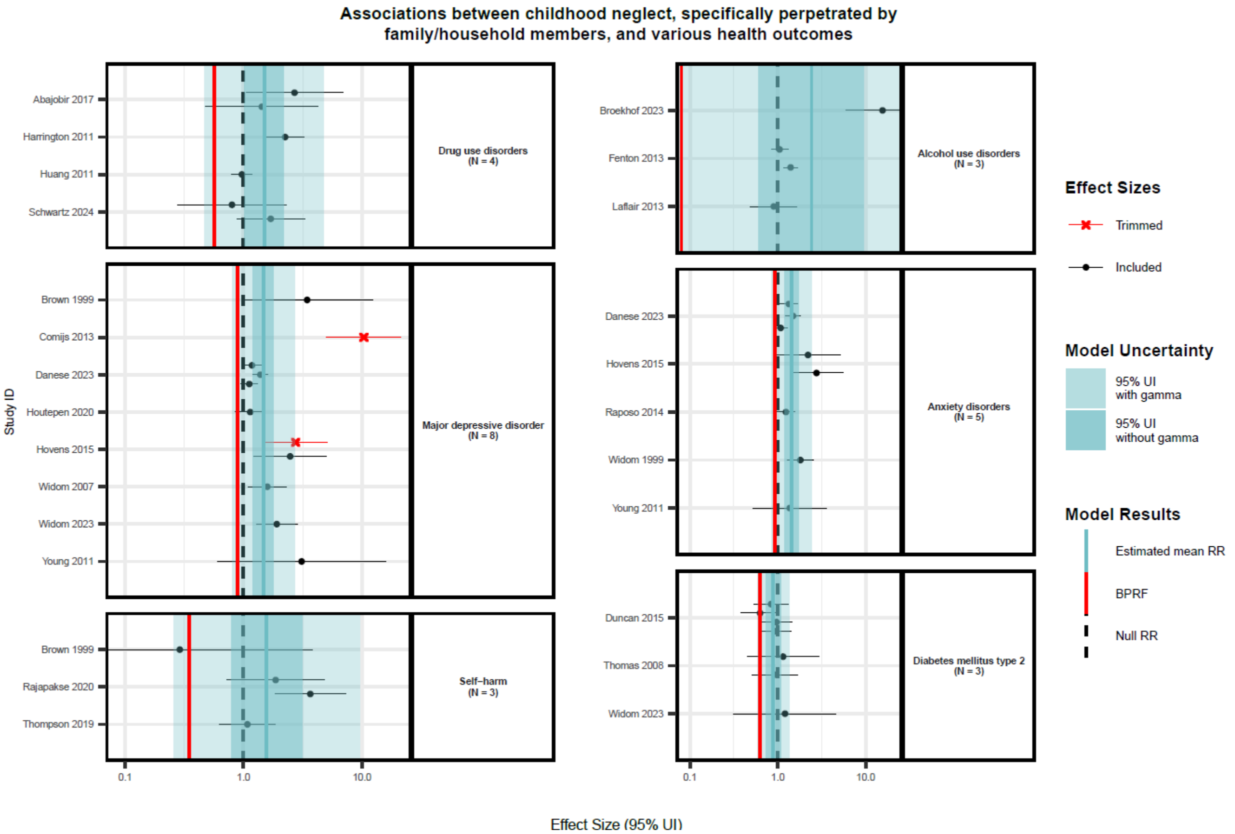

These forest plots present the estimated pooled relative risk, its 95% uncertainty intervals (UI), and the data points underlying the estimates for six health outcomes studied in association with childhood neglect perpetrated by a family/household member. Each data point and horizontal line corresponds to a mean effect size and 95% UI from the included study identified on the y-axis. The color of the point indicates whether the point was detected and trimmed as an outlier. The light blue interval corresponds to the 95% UI of the pooled relative risk when incorporating between-study heterogeneity; the dark blue interval corresponds to the 95% UI of the pooled relative risk without between-study heterogeneity. The black vertical dotted line reflects the null relative risk value (one) and the red vertical line is the burden of proof function at the 5th quantile for this harmful risk-outcome association. We truncated the x-axis to make the scale more legible, so a handful of 95% UIs from the included studies extend beyond the plot margin. We included multiple observations from a single study when effects were reported by severity/frequency of the violence exposure, by different types of violent acts, and/or separately by sex or other subgroups.

Figure S18. Sensitivity analysis results for childhood neglect by any perpetrator and outcomes

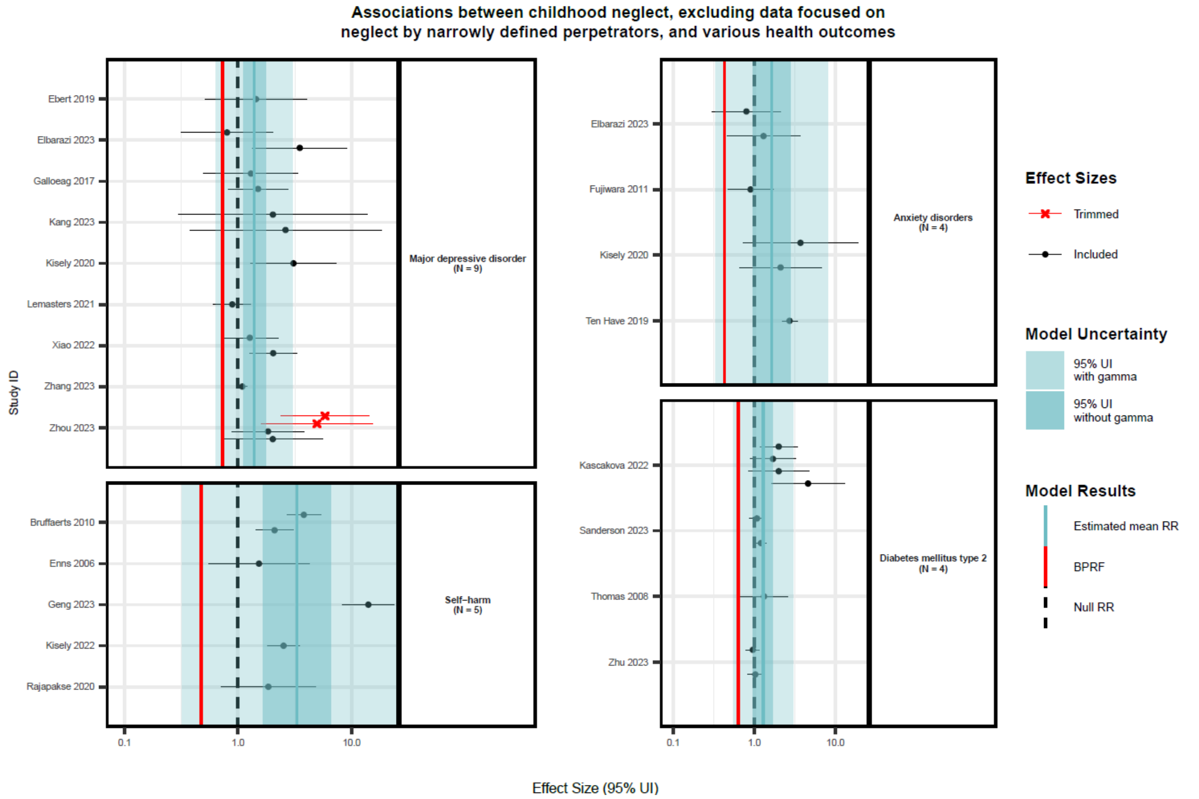

These forest plots present the estimated pooled relative risk, its 95% uncertainty intervals (UI), and the data points underlying the estimates for four health outcomes studied in association with childhood neglect with unspecified perpetrator. Each data point and horizontal line corresponds to a mean effect size and 95% UI from the included study identified on the y-axis. The color of the point indicates whether the point was detected and trimmed as an outlier. The light blue interval corresponds to the 95% UI of the pooled relative risk when incorporating between-study heterogeneity; the dark blue interval corresponds to the 95% UI of the pooled relative risk without between-study heterogeneity. The black vertical dotted line reflects the null relative risk value (one) and the red vertical line is the burden of proof function at the 5th quantile for this harmful risk-outcome association. We truncated the x-axis to make the scale more legible, so a handful of 95% UIs from the included studies extend beyond the plot margin. We included multiple observations from a single study when effects were reported by severity/frequency of the violence exposure, by different types of violent acts, and/or separately by sex or other subgroups.

Figure S19. Sensitivity analysis results for childhood neglect and alternative outcome definitions

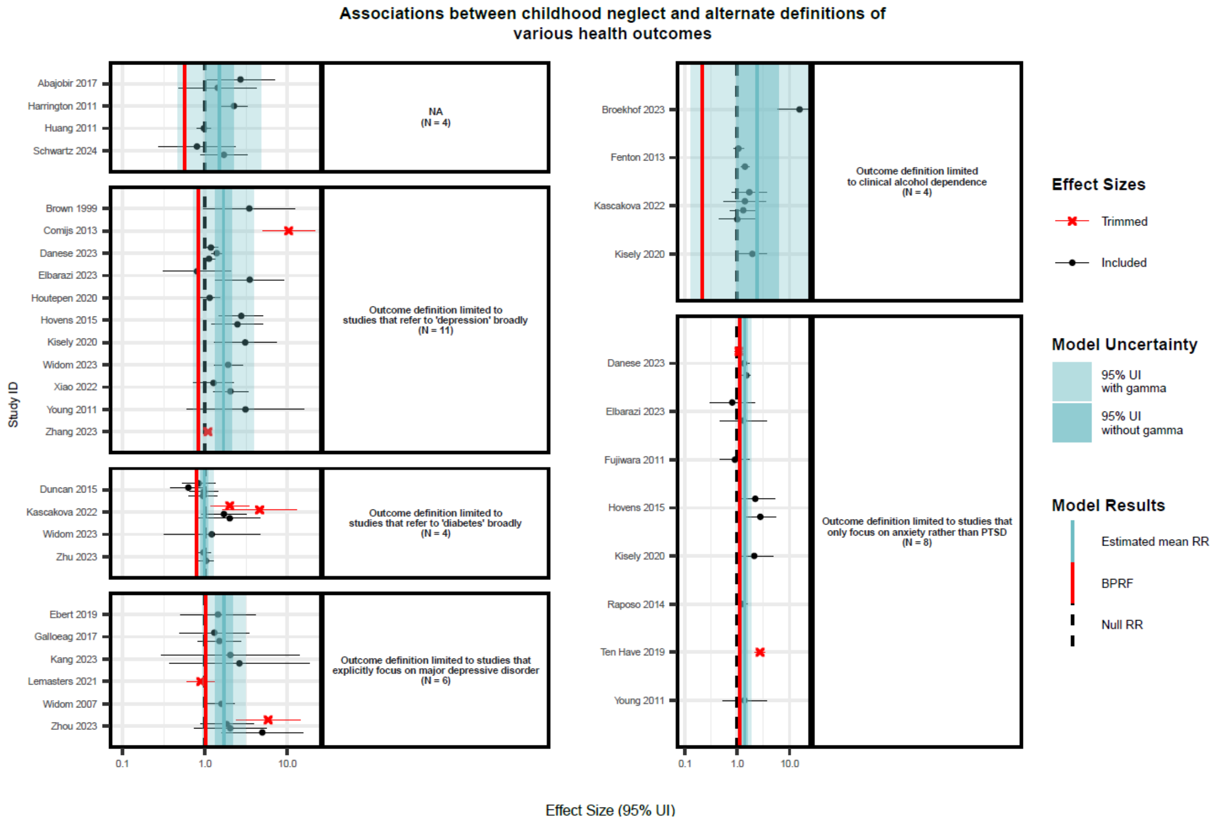

These forest plots present the estimated pooled relative risk, its 95% uncertainty intervals (UI), and the data points underlying the estimates for six health outcomes studied in association with childhood neglect. Each data point and horizontal line corresponds to a mean effect size and 95% UI from the included study identified on the y-axis. The color of the point indicates whether the point was detected and trimmed as an outlier. The light blue interval corresponds to the 95% UI of the pooled relative risk when incorporating between-study heterogeneity; the dark blue interval corresponds to the 95% UI of the pooled relative risk without between-study heterogeneity. The black vertical dotted line reflects the null relative risk value (one) and the red vertical line is the burden of proof function at the 5th quantile for this harmful risk-outcome association. We truncated the x-axis to make the scale more legible, so a handful of 95% UIs from the included studies extend beyond the plot margin. We included multiple observations from a single study when effects were reported by severity/frequency of the violence exposure, by different types of violent acts, and/or separately by sex or other subgroups.

Figure S20. Sensitivity analysis results for childhood neglect (no adjustment) and outcomes

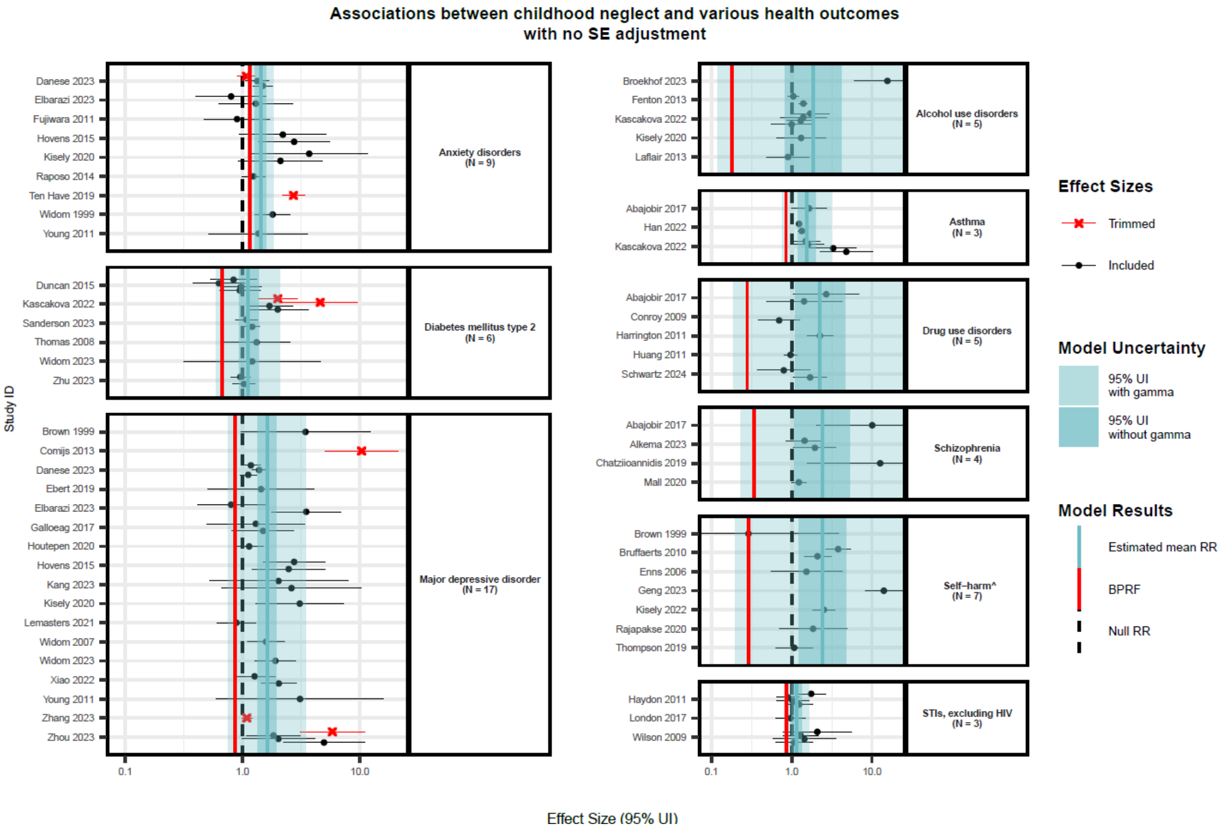

These forest plots present the estimated pooled relative risk, its 95% uncertainty intervals (UI), and the data points underlying the estimates for nine health outcomes studied in association with childhood neglect. Each data point and horizontal line corresponds to a mean effect size and 95% UI from the included study identified on the y-axis. The color of the point indicates whether the point was detected and trimmed as an outlier. The light blue interval corresponds to the 95% UI of the pooled relative risk when incorporating between-study heterogeneity; the dark blue interval corresponds to the 95% UI of the pooled relative risk without between-study heterogeneity. The black vertical dotted line reflects the null relative risk value (one) and the red vertical line is the burden of proof function at the 5th quantile for this harmful risk-outcome association. We truncated the x-axis to make the scale more legible, so a handful of 95% UIs from the included studies extend beyond the plot margin. We included multiple observations from a single study when effects were reported by severity/frequency of the violence exposure, by different types of violent acts, and/or separately by sex or other subgroups.

Figure S21. Sensitivity analysis results for childhood neglect (no trimming) and outcomes

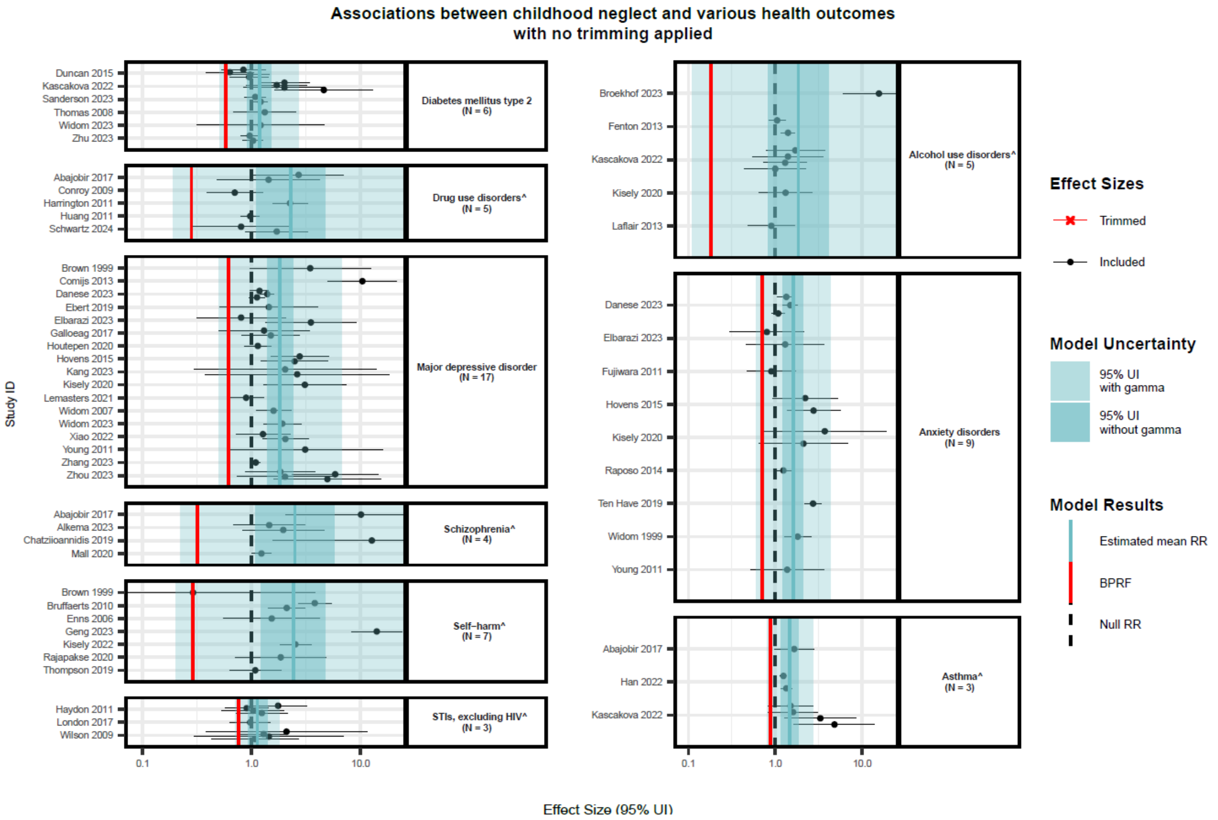

These forest plots present the estimated pooled relative risk, its 95% uncertainty intervals (UI), and the data points underlying the estimates for nine health outcomes studied in association with childhood neglect. Each data point and horizontal line corresponds to a mean effect size and 95% UI from the included study identified on the y-axis. The color of the point indicates whether the point was detected and trimmed as an outlier. The light blue interval corresponds to the 95% UI of the pooled relative risk when incorporating between-study heterogeneity; the dark blue interval corresponds to the 95% UI of the pooled relative risk without between-study heterogeneity. The black vertical dotted line reflects the null relative risk value (one) and the red vertical line is the burden of proof function at the 5th quantile for this harmful risk-outcome association. We truncated the x-axis to make the scale more legible, so a handful of 95% UIs from the included studies extend beyond the plot margin. We included multiple observations from a single study when effects were reported by severity/frequency of the violence exposure, by different types of violent acts, and/or separately by sex or other subgroups.

Section 5: Model Characteristics

Each risk factor and health outcome pair models were run when there were three or more unique studies that met our inclusion criteria. We applied 10% trimming for models that included more than ten observations. For all models, exposure types were dichotomous and there were no pre-selected covariates.

Table S19: Gamma solution for each modeled relationship

Exposure	Outcome	Gamma (SD)
Childhood Physical Violence	Diabetes Mellitus Type 2	0.000000292 (0.00175)
	Asthma	0.0402 (0.0342)
	Anxiety Disorders	0.00635 (0.0075)
	Gynecological diseases	0.0274 (0.0214)
	Maternal Abortion and Miscarriage	0.302 (0.31)
	Migraine	0.0111 (0.014)
	Alcohol Use Disorders	0.0141 (0.0186)
	Eating Disorders	0.0000171 (0.0762)
	Drug Use Disorders	0.00459 (0.0138)
	Ischemic Heart Disease	0.00000191 (0.00809)
	Major Depressive Disorder	0.012 (0.00823)
	Self-Harm	0.0723 (0.0514)
	STIs, excluding HIV	0.0362 (0.264)
	Schizophrenia	0.329 (0.341)
	Stroke	0.0000122 (0.0592)
Childhood Psychological Violence	Diabetes Mellitus Type 2	0.0019 (0.00433)
	Asthma	0.122 (0.113)
	Anxiety Disorders	0.216 (0.158)
	Gynecological diseases	0.00317 (0.00609)
	Alcohol Use Disorders	0.268 (0.187)
	Schizophrenia	0.0872 (0.142)
	Drug Use Disorders	0.00000195 (0.0092)
	Ischemic Heart Disease	0.203 (0.19)
	Major Depressive Disorder	0.163 (0.0838)
	Self-Harm	0.661 (0.382)
	Migraine	0.0343 (0.0366)
Childhood Neglect	Diabetes Mellitus Type 2	0.0104 (0.0135)
	Asthma	0.0225 (0.0304)
	Anxiety Disorders	0.000000972 (0.00725)
	Alcohol Use Disorders	0.761 (0.538)
	Schizophrenia	0.453 (0.463)
	Drug Use Disorders	0.615 (0.426)
	Major Depressive Disorder	0.0598 (0.0427)
	Self-Harm	0.652 (0.431)
	STIs, excluding HIV	0.00000423 (0.0235)

Table S20: Model characteristics of childhood violence and corresponding health outcomes

Exposure	Outcome	Exposure Type	Pre-selected covariates	Trimming Percentage

Childhood Physical Violence	Diabetes Mellitus Type 2	Dichotomous	None	10% trimming
	Asthma	Dichotomous	None	None
	Anxiety Disorders	Dichotomous	None	10% trimming
	Gynecological diseases	Dichotomous	None	None
	Maternal Abortion and Miscarriage	Dichotomous	None	None
	Migraine	Dichotomous	None	None
	Alcohol Use Disorders	Dichotomous	None	10% trimming
	Eating Disorders	Dichotomous	None	None
	Drug Use Disorders	Dichotomous	None	10% trimming
	Ischemic Heart Disease	Dichotomous	None	None
	Major Depressive Disorder	Dichotomous	None	10% trimming
	Self-Harm	Dichotomous	None	10% trimming
	Sexually Transmitted Infections excluding HIV	Dichotomous	None	None
	Schizophrenia	Dichotomous	None	None
	Stroke	Dichotomous	None	None
Childhood Psychological Violence	Diabetes Mellitus Type 2	Dichotomous	None	10% trimming
	Asthma	Dichotomous	None	None
	Anxiety Disorders	Dichotomous	None	None
	Gynecological diseases	Dichotomous	None	None
	Alcohol Use Disorders	Dichotomous	None	10% trimming
	Schizophrenia	Dichotomous	None	None
	Drug Use Disorders	Dichotomous	None	10% trimming
	Ischemic Heart Disease	Dichotomous	None	None
	Major Depressive Disorder	Dichotomous	None	10% trimming
	Self-Harm	Dichotomous	None	None
	Migraine	Dichotomous	None	None
Childhood Neglect	Diabetes Mellitus Type 2	Dichotomous	None	10% trimming
	Asthma	Dichotomous	None	None
	Anxiety Disorders	Dichotomous	None	10% trimming
	Alcohol Use Disorders	Dichotomous	None	None
	Schizophrenia	Dichotomous	None	None

	Drug Use Disorders	Dichotomous	None	None
	Major Depressive Disorder	Dichotomous	None	10% trimming
	Self-Harm	Dichotomous	None	None
	Sexually Transmitted Infections excluding HIV	Dichotomous	None	None

Section 6: PRISMA and GATHER Checklists

Section 6.1: PRISMA

Table S21. PRISMA 2020 abstract checklist

Section and Topic	Item #	Checklist item	Reported (Yes/No)
**TITLE**			
Title	1	Identify the report as a systematic review.	Yes.
**BACKGROUND**			
Objectives	2	Provide an explicit statement of the main objective(s) or question(s) the review addresses.	Yes.
**METHODS**			
Eligibility criteria	3	Specify the inclusion and exclusion criteria for the review.	Not in abstract. Covered in detail in the main text and supplementary information.
Information sources	4	Specify the information sources (e.g. databases, registers) used to identify studies and the date when each was last searched.	Not in abstract. Covered in detail in the main text and supplementary information.
Risk of bias	5	Specify the methods used to assess risk of bias in the included studies.	Not in abstract. Covered in detail in the main text and supplementary information.
Synthesis of results	6	Specify the methods used to present and synthesise results.	Yes, briefly. More detail provided in the main text and supplementary information.
**RESULTS**			
Included studies	7	Give the total number of included studies and participants and summarise relevant characteristics of studies.	Not in abstract. Covered in detail in the main text and supplementary information.
Synthesis of results	8	Present results for main outcomes, preferably indicating the number of included studies and participants for each. If meta-analysis was done, report the summary estimate and confidence/credible interval. If comparing groups, indicate the direction of the effect (i.e. which group is favoured).	Yes, briefly. More detail provided in the main text and supplementary information.
**DISCUSSION**			
Limitations of evidence	9	Provide a brief summary of the limitations of the evidence included in the review (e.g. study risk of bias, inconsistency and imprecision).	Not in abstract. Covered in detail in the main text and supplementary information.

Interpretation	10	Provide a general interpretation of the results and important implications.	Yes
**OTHER**			
Funding	11	Specify the primary source of funding for the review.	Not in abstract but described in the main text.
Registration	12	Provide the register name and registration number.	Not in abstract but reported in main text methods section.

Table S22. PRISMA 2020 checklist

Section and Topic	Item #	Checklist item	Location where item is reported
**TITLE**			
Title	1	Identify the report as a systematic review.	Manuscript Title and Methods Section Headings
**ABSTRACT**			
Abstract	2	See the PRISMA 2020 for Abstracts checklist.	Abstract section in main text
**INTRODUCTION**			
Rationale	3	Describe the rationale for the review in the context of existing knowledge.	Main section in-text
Objectives	4	Provide an explicit statement of the objective(s) or question(s) the review addresses.	Main section in-text
**METHODS**			
Eligibility criteria	5	Specify the inclusion and exclusion criteria for the review and how studies were grouped for the syntheses.	"Systematic Review" section in Methods & Section 8.2: Inclusion and Exclusion Criteria in Supplementary Information
Information sources	6	Specify all databases, registers, websites, organisations, reference lists and other sources searched or consulted to identify studies. Specify the date when each source was last searched or consulted.	"Systematic Review" section in Methods & Section 8.1: Literature searches in Supplementary Information
Search strategy	7	Present the full search strategies for all databases, registers and websites, including any filters and limits used.	"Systematic Review" section in Methods & Section 8.1: Literature searches in Supplementary Information
Selection process	8	Specify the methods used to decide whether a study met the inclusion criteria of the review, including how many reviewers screened each record and each report retrieved, whether they worked independently, and if applicable, details of automation tools used in the process.	"Systematic Review" section in Methods main text
Data collection process	9	Specify the methods used to collect data from reports, including how many reviewers collected data from each report, whether they worked independently, any processes for obtaining or confirming data from study investigators, and if applicable, details of automation tools used in the process.	"Systematic Review" section in Methods main text
Data items	10a	List and define all outcomes for which data were sought. Specify whether all results that were compatible with each outcome domain in each study were sought (e.g. for all measures, time points, analyses), and if not, the methods used to decide which results to collect.	"Health outcomes and data selection" section in Methods main text; Supplementary

			Information Section 2: Exposure Definitions; Supplementary Information Section 4: GBD Outcome Definitions
	10b	List and define all other variables for which data were sought (e.g. participant and intervention characteristics, funding sources). Describe any assumptions made about any missing or unclear information.	Section 8.3; Data Extraction in Supplementary Information
Study risk of bias assessment	11	Specify the methods used to assess risk of bias in the included studies, including details of the tool(s) used, how many reviewers assessed each study and whether they worked independently, and if applicable, details of automation tools used in the process.	“Testing and adjusting for biases across study designs and characteristics” in Methods section; Supplementary Information Section 9.1: Definitions of bias covariates
Effect measures	12	Specify for each outcome the effect measure(s) (e.g. risk ratio, mean difference) used in the synthesis or presentation of results.	Supplementary Information Section 3.1: Effect size data details for childhood physical violence, psychological violence, and neglect
Synthesis methods	13a	Describe the processes used to decide which studies were eligible for each synthesis (e.g. tabulating the study intervention characteristics and comparing against the planned groups for each synthesis (item #5)).	“Health outcomes and data Selection” in Methods main text
	13b	Describe any methods required to prepare the data for presentation or synthesis, such as handling of missing summary statistics, or data conversions.	Methods main text; Section 3 in Supplementary Information
	13c	Describe any methods used to tabulate or visually display results of individual studies and syntheses.	Methods main text; Sections 3 & 10 in Supplementary Information
	13d	Describe any methods used to synthesize results and provide a rationale for the choice(s). If meta-analysis was performed, describe the model(s), method(s) to identify the presence and extent of statistical heterogeneity, and software package(s) used.	“Estimating the burden of proof risk function” methods main text; Sections 10 & 6 in Supplementary Information
	13e	Describe any methods used to explore possible causes of heterogeneity among study results (e.g. subgroup analysis, meta-regression).	“Quantifying between-study heterogeneity” methods main text; Supplementary Information Section 5: Sensitivity Analyses
	13f	Describe any sensitivity analyses conducted to assess robustness of the synthesized results.	Results section in main text; Supplementary Information Section 5: Sensitivity Analyses
Reporting bias assessment	14	Describe any methods used to assess risk of bias due to missing results in a synthesis (arising from reporting biases).	“Testing and adjusting for biases across study designs and characteristics” in Methods main text; Section 9: Study Quality and Bias Assessment in Supplementary Information

Certainty assessment	15	Describe any methods used to assess certainty (or confidence) in the body of evidence for an outcome.	"Estimating the burden of proof risk function" in Methods main text
**RESULTS**			
Study selection	16a	Describe the results of the search and selection process, from the number of records identified in the search to the number of studies included in the review, ideally using a flow diagram.	PRISMA flow diagram (Section 1 in Supplementary Information); "Health outcomes and data selection" in methods main text
	16b	Cite studies that might appear to meet the inclusion criteria, but which were excluded, and explain why they were excluded.	PRISMA flow diagram (Section 1 in Supplementary Information)
Study characteristics	17	Cite each included study and present its characteristics.	Table S3: Summary characteristics of studies analyzed for childhood physical abuse, psychological abuse, and neglect (Section 3: Data Inputs) in Supplementary Information
Risk of bias in studies	18	Present assessments of risk of bias for each included study.	Section 9.2: Bias covariates for each risk-outcome pairs in Supplementary Information
Results of individual studies	19	For all outcomes, present, for each study: (a) summary statistics for each group (where appropriate) and (b) an effect estimate and its precision (e.g. confidence/credible interval), ideally using structured tables or plots.	Results section in main text; Table 2; Figures 2-4
Results of syntheses	20a	For each synthesis, briefly summarise the characteristics and risk of bias among contributing studies.	Section 3: Data inputs in Supplementary Information; Section 9: Study Quality and Bias Assessment in Supplementary Information
	20b	Present results of all statistical syntheses conducted. If meta-analysis was done, present for each the summary estimate and its precision (e.g. confidence/credible interval) and measures of statistical heterogeneity. If comparing groups, describe the direction of the effect.	Results section in main text; Table 2; Figures 2-4
	20c	Present results of all investigations of possible causes of heterogeneity among study results.	Section 5: Sensitivity Analyses in Supplementary Information
	20d	Present results of all sensitivity analyses conducted to assess the robustness of the synthesized results.	Section 5: Sensitivity Analyses in Supplementary Information
Reporting biases	21	Present assessments of risk of bias due to missing results (arising from reporting biases) for each synthesis assessed.	Results section main text; Section 9 in Supplementary Results
Certainty of evidence	22	Present assessments of certainty (or confidence) in the body of evidence for each outcome assessed.	Results section main text
**DISCUSSION**			
Discussion	23a	Provide a general interpretation of the results in the context of other evidence.	Discussion section main

			text
	23b	Discuss any limitations of the evidence included in the review.	Discussion section main text
	23c	Discuss any limitations of the review processes used.	Discussion section main text
	23d	Discuss implications of the results for practice, policy, and future research.	Discussion section main text
**OTHER INFORMATION**			
Registration and protocol	24a	Provide registration information for the review, including register name and registration number, or state that the review was not registered.	The systematic review was registered in PROSPERO (CRD42022299831); The entirety of the Global Burden of Diseases, Injuries, and Risk Factors Study has been registered and approved through the UW IRB, as detailed in Methods section main text.
	24b	Indicate where the review protocol can be accessed, or state that a protocol was not prepared.	Review protocol publication cited in Methods section main text; review was registered in PROSPERO (CRD42022299831)
	24c	Describe and explain any amendments to information provided at registration or in the protocol.	NA
Support	25	Describe sources of financial or non-financial support for the review, and the role of the funders or sponsors in the review.	Main text “Acknowledgments”
Competing interests	26	Declare any competing interests of review authors.	Main text “Competing interests”
Availability of data, code and other materials	27	Report which of the following are publicly available and where they can be found; template data collection forms; data extracted from included studies; data used for all analyses; analytic code; any other materials used in the review.	Main text “Data availability” and “Code availability” sections; data collection form template in Table S26: Data extraction template in Supplementary Information

Section 6.2: GATHER

Table S23. GATHER checklist

Item #	Checklist item	Reported on page #
Objectives and funding		
1	Define the indicator(s), populations (including age, sex, and geographic entities), and time period(s) for which estimates were made.	Methods
2	List the funding sources for the work.	Acknowledgements
Data Inputs		
For all data inputs from multiple sources that are synthesized as part of the study:		
3	Describe how the data were identified and how the data were accessed.	Methods
4	Specify the inclusion and exclusion criteria. Identify all ad-hoc exclusions.	Methods; Supplementary Information Section 8.2
5	Provide information on all included data sources and their main characteristics. For each data source used, report reference information or contact name/institution, population represented, data collection method, year(s) of data collection, sex and age range, diagnostic criteria or measurement method, and sample size, as relevant.	SI Table S3
6	Identify and describe any categories of input data that have potentially important biases (e.g., based on characteristics listed in item 5).	SI Section 9
For data inputs that contribute to the analysis but were not synthesized as part of the study:		
7	Describe and give sources for any other data inputs.	N/A
For all data inputs:		
8	Provide all data inputs in a file format from which data can be efficiently extracted (e.g., a spreadsheet rather than a PDF), including all relevant meta-data listed in item 5. For any data inputs that cannot be shared because of ethical or legal reasons, such as third-party ownership, provide a contact name or the name of the institution that retains the right to the data.	SI Section 3; SI Section 11
Data analysis		
9	Provide a conceptual overview of the data analysis method. A diagram may be helpful.	Methods
10	Provide a detailed description of all steps of the analysis, including mathematical formulae. This description should cover, as relevant, data cleaning, data pre-processing, data adjustments and weighting of data sources, and mathematical or statistical model(s).	Methods
11	Describe how candidate models were evaluated and how the final model(s) were selected.	Methods “Model validation”
12	Provide the results of an evaluation of model performance, if done, as well as the results of any relevant sensitivity analysis.	Methods “Model validation” section; SI section 5
13	Describe methods for calculating uncertainty of the estimates. State which sources of uncertainty were, and were not, accounted for in the uncertainty analysis.	Methods “Quantifying between-study heterogeneity” section
14	State how analytic or statistical source code used to generate estimates can be accessed.	Code availability
Results and Discussion		
15	Provide published estimates in a file format from which data can be efficiently extracted.	Table 2; Burden of Proof visualization tool (URL to be provided at resubmission)
16	Report a quantitative measure of the uncertainty of the estimates (e.g. uncertainty intervals).	Results; Table 2
17	Interpret results in light of existing evidence. If updating a previous set of estimates, describe the reasons for changes in estimates.	Discussion
18	Discuss limitations of the estimates. Include a discussion of any modelling assumptions or data limitations that affect interpretation of the estimates.	Discussion

Section 7: Data Identification and Assessment

The following systematic review methodology was adapted from the "Supplementary Information" of the publication titled "Health effects associated with exposure to intimate partner violence against women and childhood abuse: a burden of proof study". We conducted a systematic search of 7 databases (PubMed; Embase/Elsevier; Cumulative Index to Nursing and Allied Health Literature (CINAHL), PsychInfo; Global Index Medicus; Cochrane; Web of Science) to identify relevant literature published from 1 January 1970 to 31 January 2024. The date of our original search was September 30, 2021, and our search was updated to include literature published between 30 September and 31 January 2024 on February 26, 2024. The search strings applied to each database are reported in Section 6.1 and have been previously published in our review protocol[1]. Systematic reviews that were captured through our search were flagged for separate citation searching process (Supplementary Information Section 8.4). All sources were title/abstract and full text screened for acceptance based on the inclusion and exclusion criteria detailed in section 8.2.

Section 7.1: Literature searches

PubMed Search String

("Sex Offenses"[mh] OR "Violence"[mh:noexp] OR "Domestic Violence"[mh] OR "Gender-Based Violence"[mh] OR "Intimate Partner Violence"[mh] OR "Physical Abuse"[mh] OR "Rape"[mh] OR "Torture"[mh] OR "Workplace Violence"[mh] OR "Gun violence"[mh] OR "Battered Women"[mh] OR "Adult Survivors of Child abuse"[mh] OR "Exposure to Violence"[mh] OR "Emotional Abuse"[mh] OR "Sexual Harassment"[mh] OR "Harassment, Non-Sexual"[mh:noexp] OR "Aggression"[mh:noexp] OR "Coercion"[Mesh] OR "Dehumanization"[mh] OR "stalking"[mh] OR "adverse childhood experiences"[mh] OR violence[tiab] OR "sexual assault"[tiab] OR "sexual harassment"[tiab] OR "sexual abuse"[tiab] OR "sex abuse"[tiab] OR rape[tiab] OR "forced sex"[tiab] OR "sexual coercion"[tiab] OR "reproductive coercion"[tiab] OR "sex trafficking"[tiab] OR "sexual exploitation"[tiab] OR "forced marriage"[tiab] OR "child marriage*"[tiab] OR "early marriage*"[tiab] OR "child bride*"[tiab] OR CEFM[tiab] OR "female genital mutilation"[tiab] OR "female genital cutting"[tiab] OR "female circumcision"[tiab] OR "female genital circumcision"[tiab] OR infibulation*[tiab] OR clitoridectom*[tiab] OR clitorectom*[tiab] OR "ritual female genital surger*"[tiab] OR FGM[tiab] OR "physical abuse"[tiab] OR "psychological abuse"[tiab] OR "emotional abuse"[tiab] OR "economic abuse"[tiab] OR "financial abuse"[tiab] OR "verbal abuse"[tiab] OR maltreatment[tiab] OR "violent discipline"[tiab] OR "corporal punishment"[tiab] OR "adverse childhood experience*"[tiab] OR molestation[tiab] OR "child abuse"[tiab] OR "partner abuse"[tiab] OR "dating abuse"[tiab] OR "wife abuse"[tiab] OR "spouse abuse"[tiab] OR "domestic abuse"[tiab] OR "elder abuse"[tiab] OR "senior abuse"[tiab] OR "aged abuse"[tiab] OR victimization[tiab] OR dehumanization[tiab] OR victimisation[tiab] OR dehumanisation[tiab] OR stalking[tiab] OR cyberviolence[tiab] OR cybervictimization[tiab] OR cyberstalking[tiab])

AND

(Case-Control Studies[mh] OR Cross-Over Studies[mh] OR Cohort Studies[mh] OR Systematic Review[pt] OR Meta-Analysis[pt] OR "Twin Study"[pt] OR "systematic review"[tiab] OR "meta-

analysis”[tiab] OR “cohort”[tiab] OR “cross-over”[tiab] OR “case-control”[tiab] OR “prospective”[tiab] OR “retrospective”[tiab] OR “longitudinal”[tiab] OR “follow-up”[tiab] OR “followup”[tiab])
AND
("Statistics as Topic"[mh] OR Risk[mh] OR Odds Ratio[mh] OR “risk*”[tiab] OR “odds”[tiab] OR “cross-product ratio*”[tiab] OR “hazards ratio*”[tiab] OR “hazard ratio*”[tiab] OR statistic*[tiab] OR “HR”[tiab] OR “RR”[tiab] OR “aOR”[tiab] OR relation*[tiab] OR correlat*[tiab] OR associat*[tiab] OR likel*[tiab])
AND
("1970/01/01"[PDat] : "2024/01/31"[PDat])

Embase Search String

('sexual violence'/exp OR 'forced sex'/exp OR 'violence'/de OR 'domestic violence'/exp OR 'gender based violence'/exp OR 'partner violence'/exp OR 'dating violence'/exp OR 'physical abuse'/exp OR 'physical violence'/exp OR 'torture'/exp OR 'workplace violence'/exp OR 'gun violence'/exp OR 'battered woman'/exp OR 'child abuse survivor'/exp OR 'exposure to violence'/exp OR 'emotional abuse'/exp OR 'elderly abuse'/exp OR 'sexual harassment'/exp OR 'non-sexual harassment'/de OR 'aggression'/de OR 'verbal hostility'/exp OR 'coercion'/exp OR 'intimidation'/exp OR 'dehumanization'/exp OR 'stalking'/de OR 'childhood adversity'/exp OR 'maltreatment'/exp OR 'corporal punishment'/exp OR 'victimization'/exp OR (violence OR 'sexual assault' OR 'sexual harassment' OR 'sexual abuse' OR 'sex abuse' OR rape OR 'forced sex' OR 'sexual coercion' OR 'reproductive coercion' OR 'sex trafficking' OR 'sexual exploitation' OR 'forced marriage*' OR ‘child marriage*’ OR ‘early marriage*’ OR ‘child bride*’ OR CEFM OR 'female genital mutilation' OR 'female genital cutting' OR 'female circumcision' OR 'female genital circumcision' OR infibulation* OR clitoridectom* OR clitorectom* OR ‘ritual female genital surger*’ OR FGM OR 'physical abuse' OR 'psychological abuse' OR 'emotional abuse' OR 'economic abuse' OR ‘financial abuse’ OR 'verbal abuse' OR maltreatment OR 'violent discipline' OR 'corporal punishment' OR 'adverse childhood experience*' OR molestation OR 'child abuse' OR 'partner abuse' OR 'dating abuse' OR 'wife abuse' OR 'spouse abuse' OR 'domestic abuse' OR 'elder abuse' OR 'senior abuse' OR 'aged abuse' OR victimization OR dehumanisation OR victimisation OR dehumanization OR stalking OR cyberviolence OR cybervictimization OR cyberstalking):ti,ab,kw)
AND
('case control study'/exp OR 'crossover procedure'/exp OR 'cohort analysis'/exp OR 'systematic review'/exp OR 'systematic review (topic)'/exp OR 'meta analysis'/exp OR 'meta analysis (topic)'/exp OR 'twin study'/exp OR ('systematic review' OR meta-analysis OR cohort OR cross-over OR case-control OR prospective OR retrospective OR longitudinal OR follow-up OR followup):ti,ab,kw)
AND
('statistics'/exp OR 'statistical parameters'/exp OR 'risk'/exp OR 'risk ratio'/exp OR 'correlation'/exp OR 'statistical analysis'/exp OR ('risk*' OR 'odds' OR 'cross-product ratio*' OR

'hazards ratio*' OR 'hazard ratio*' OR statistic* OR 'HR' OR 'RR' OR 'aOR' OR relation* OR correlat* OR associat* OR likel*):ti,ab,kw)
AND
[1970-2024]/py
AND
[01-01-1970]/sd NOT [31-01-2024]/sd

Cumulative Index to Nursing and Allied Health Literature (CINAHL) Search String

((MH ("Sexual Abuse+" OR "Violence" OR "Domestic Violence+" OR "Gender-Based Violence" OR "Circumcision, Female" OR "Dating Violence" OR "Torture" OR "Workplace Violence" OR "Gun Violence" OR "Battered Women" OR "Child Abuse Survivors" OR "Exposure to Violence" OR "Emotional Abuse" OR "Sexual Harassment" OR "Aggression" OR "Verbal Abuse" OR "Coercion" OR "Dehumanization" OR "Stalking" OR "Adverse Childhood Experiences" OR "Sibling Violence" OR "School Violence" OR "Student Abuse")) OR TI(violence OR "sexual assault" OR "sexual harassment" OR "sexual abuse" OR "sex abuse" OR rape OR "forced sex" OR "sexual coercion" OR "reproductive coercion" OR "sex trafficking" OR "sexual exploitation" OR "forced marriage" OR “child marriage*” OR “early marriage*” OR “child bride*” OR CEFM OR "female genital mutilation" OR "female genital cutting" OR "female circumcision" OR "female genital circumcision" OR infibulation* OR clitoridectom* OR clitorectom* OR “ritual female genital surger*” OR FGM OR "physical abuse" OR "psychological abuse" OR "emotional abuse" OR "economic abuse" OR “financial abuse” OR "verbal abuse" OR maltreatment OR "violent discipline" OR "corporal punishment" OR "adverse childhood experience*" OR molestation OR "child abuse" OR "partner abuse" OR "dating abuse" OR "wife abuse" OR "spouse abuse" OR "domestic abuse" OR "elder abuse" OR "senior abuse" OR "aged abuse" OR victimization OR dehumanization OR victimisation OR dehumanisation OR stalking OR cyberviolence OR cybervictimization OR cyberstalking) OR AB(violence OR "sexual assault" OR "sexual harassment" OR "sexual abuse" OR "sex abuse" OR rape OR "forced sex" OR "sexual coercion" OR "reproductive coercion" OR "sex trafficking" OR "sexual exploitation" OR "forced marriage" OR “child marriage*” OR “early marriage*” OR “child bride*” OR CEFM OR "female genital mutilation" OR "female genital cutting" OR "female circumcision" OR "female genital circumcision" OR infibulation* OR clitoridectom* OR clitorectom* OR “ritual female genital surger*” OR FGM OR "physical abuse" OR "psychological abuse" OR "emotional abuse" OR "economic abuse" OR “financial abuse” OR "verbal abuse" OR maltreatment OR "violent discipline" OR "corporal punishment" OR "adverse childhood experience*" OR molestation OR "child abuse" OR "partner abuse" OR "dating abuse" OR "wife abuse" OR "spouse abuse" OR "domestic abuse" OR "elder abuse" OR "senior abuse" OR "aged abuse" OR victimization OR dehumanization OR victimisation OR dehumanisation OR stalking OR cyberviolence OR cybervictimization OR cyberstalking))
AND
((MH ("Case Control Studies+" OR "Crossover Design" OR "Systematic Review" OR "Meta Analysis" OR "Prospective Studies+" OR "Retrospective Design")) OR TI("systematic review" OR

meta-analysis OR cohort OR cross-over OR case-control OR prospective OR retrospective OR longitudinal OR follow-up OR followup) OR AB("systematic review" OR meta-analysis OR cohort OR cross-over OR case-control OR prospective OR retrospective OR longitudinal OR follow-up OR followup))
AND
((MH ("Statistics+" OR "Data Analysis, Statistical+")) OR TI(risk* OR odds OR "cross-product ratio*" OR "hazards ratio*" OR "hazard ratio*" OR statistic* OR "HR" OR "RR" OR "aOR" OR relation* OR correlat* OR associat* OR likel*) OR AB(risk* OR odds OR "cross-product ratio*" OR "hazards ratio*" OR "hazard ratio*" OR statistic* OR "HR" OR "RR" OR "aOR" OR relation* OR correlat* OR associat* OR likel*))
)
Limits: Publication Date: January 1970 – January 2024

PsycINFO Search String

(DE("Sexual Violence" OR "Sex Offenses" OR "Sexual Abuse" OR "Sexual Coercion" OR "Sex Trafficking" OR "Violence" OR "Domestic Violence" OR "Child Abuse" OR "Elder Abuse" OR "Circumcision" OR "Intimate Partner Violence" OR "Dating Violence" OR "Physical Abuse" OR "Physical Discipline" OR "Punishment" OR "Rape" OR "Acquaintance Rape" OR "Torture" OR "Workplace Violence" OR "Gun Violence" OR "Battered Females" OR "Exposure to Violence" OR "Emotional Abuse" OR "Sexual Harassment" OR "Aggressive Behavior" OR "Verbal Abuse" OR "Coercion" OR "Stalking" OR "Childhood Adversity" OR "School Violence" OR "Police Violence" OR "Victimization")
OR
MA("Sex Offenses" OR "Domestic Violence" OR "Gender-Based Violence" OR "Intimate Partner Violence" OR "Physical Abuse" OR "Rape" OR "Torture" OR "Workplace Violence" OR "Gun violence" OR "Battered Women" OR "Adult Survivors of Child abuse" OR "Exposure to Violence" OR "Emotional Abuse" OR "Sexual Harassment" OR "Coercion" OR "Dehumanization" OR "stalking" OR “adverse childhood experiences”)
OR
TI(violence OR "sexual assault" OR "sexual harassment" OR “sexual abuse” OR “sex abuse” OR rape OR “forced sex” OR “sexual coercion” OR “reproductive coercion” OR “sex trafficking” OR “sexual exploitation” OR “forced marriage*” OR “child marriage*” OR “early marriage*” OR “child bride*” OR CEFM OR “female genital mutilation” OR “female genital cutting” OR “female circumcision” OR “female genital circumcision” OR infibulation* OR clitoridectom* OR clitorectom* OR “ritual female genital surger*” OR FGM OR “physical abuse” OR “psychological abuse” OR “emotional abuse” OR “economic abuse” OR “financial abuse” OR "verbal abuse" OR maltreatment OR “violent discipline” OR “corporal punishment” OR “adverse childhood experience*” OR molestation OR “child abuse” OR “partner abuse” OR "dating abuse" OR “wife abuse” OR “spouse abuse” OR “domestic abuse” OR “elder abuse” OR “senior abuse” OR “aged abuse” OR victimization OR dehumanization OR victimisation OR dehumanisation OR stalking OR cyberviolence OR cybervictimization OR cyberstalking)

OR
AB(violence OR "sexual assault" OR "sexual harassment" OR “sexual abuse” OR “sex abuse” OR rape OR “forced sex” OR “sexual coercion” OR “reproductive coercion” OR “sex trafficking” OR “sexual exploitation” OR “forced marriage*” OR “child marriage*” OR “early marriage*” OR “child bride*” OR CEFM OR “female genital mutilation” OR “female genital cutting” OR “female circumcision” OR “female genital circumcision” OR infibulation* OR clitoridectom* OR clitorectom* OR “ritual female genital surger*” OR FGM OR “physical abuse” OR “psychological abuse” OR “emotional abuse” OR “economic abuse” OR “financial abuse” OR "verbal abuse" OR maltreatment OR “violent discipline” OR “corporal punishment” OR “adverse childhood experience*” OR molestation OR “child abuse” OR “partner abuse” OR "dating abuse" OR “wife abuse” OR “spouse abuse” OR “domestic abuse” OR “elder abuse” OR “senior abuse” OR “aged abuse” OR victimization OR dehumanization OR victimisation OR dehumanisation OR stalking OR cyberviolence OR cybervictimization OR cyberstalking)
OR
KW(violence OR "sexual assault" OR "sexual harassment" OR “sexual abuse” OR “sex abuse” OR rape OR “forced sex” OR “sexual coercion” OR “reproductive coercion” OR “sex trafficking” OR “sexual exploitation” OR “forced marriage*” OR “child marriage*” OR “early marriage*” OR “child bride*” OR CEFM OR “female genital mutilation” OR “female genital cutting” OR “female circumcision” OR “female genital circumcision” OR infibulation* OR clitoridectom* OR clitorectom* OR “ritual female genital surger*” OR FGM OR “physical abuse” OR “psychological abuse” OR “emotional abuse” OR “economic abuse” OR “financial abuse” OR "verbal abuse" OR maltreatment OR “violent discipline” OR “corporal punishment” OR “adverse childhood experience*” OR molestation OR “child abuse” OR “partner abuse” OR "dating abuse" OR “wife abuse” OR “spouse abuse” OR “domestic abuse” OR “elder abuse” OR “senior abuse” OR “aged abuse” OR victimization OR dehumanization OR victimisation OR dehumanisation OR stalking OR cyberviolence OR cybervictimization OR cyberstalking))
AND
(DE("Cohort Analysis" OR "Followup Studies" OR "Longitudinal Studies" OR "Retrospective Studies" OR "Prospective Studies" OR "Systematic Review" OR "Meta Analysis")
OR
MA("Case-Control Studies" OR "Cross-Over Studies" OR "Cohort Studies")
OR
TI("systematic review" OR meta-analysis OR cohort OR cross-over OR case-control OR prospective OR retrospective OR longitudinal OR follow-up OR followup)
OR
AB("systematic review" OR meta-analysis OR cohort OR cross-over OR case-control OR prospective OR retrospective OR longitudinal OR follow-up OR followup)
OR
KW("systematic review" OR meta-analysis OR cohort OR cross-over OR case-control OR prospective OR retrospective OR longitudinal OR follow-up OR followup))
AND

(DE("Statistics" OR "Statistical Analysis" OR "Risk Assessment" OR "Statistical Correlation")
OR
MA("Statistics as Topic" OR Risk OR Odds Ratio)
OR
TI(risk* OR odds OR "cross-product ratio*" OR "hazards ratio*" OR "hazard ratio*" OR statistic* OR "HR" OR "RR" OR "aOR" OR relation* OR correlat* OR associat* OR likel*)
OR
AB(risk* OR odds OR "cross-product ratio*" OR "hazards ratio*" OR "hazard ratio*" OR statistic* OR "HR" OR "RR" OR "aOR" OR relation* OR correlat* OR associat* OR likel*)
OR
KW(risk* OR odds OR "cross-product ratio*" OR "hazards ratio*" OR "hazard ratio*" OR statistic* OR "HR" OR "RR" OR "aOR" OR relation* OR correlat* OR associat* OR likel*))
Limits: Publication Date: January 1970 – January 2024

Global Index Medicus Search String

(((mh:(I01.198.240.748* OR Violence OR I01.198.240.856.350* OR I01.198.240.856.463 OR I01.198.240.856.575* OR I01.198.240.856.688 OR I01.198.240.748.640 OR I01.198.240.856.825 OR I01.198.240.856.912 OR I01.198.240.856.519 OR M01.975.155 OR M01.135.500 OR I01.880.735.900.869 OR I01.880.735.305 OR SP9.020.800.010 OR "Harassment, Non-Sexual" OR "Aggression" OR I01.880.604.316 OR Dehumanization OR F01.145.813.191* OR I01.880.735.223.500 OR I01.880.735.035)) OR (ti:(violence OR "sexual assault" OR "sexual harassment" OR "sexual abuse" OR "sex abuse" OR rape OR "forced sex" OR "sexual coercion" OR "reproductive coercion" OR "sex trafficking" OR "sexual exploitation" OR "forced marriage" OR "forced marriages" OR "child marriage" OR "child marriages" OR "early marriage" OR "early marriages" OR "child bride" OR "child brides" OR CEFM OR "female genital mutilation" OR "female genital cutting" OR "female circumcision" OR "female genital circumcision" OR infibulation* OR clitoridectom* OR clitorectom* OR "ritual female genital surgery" OR "ritual female genital surgeries" OR FGM OR "physical abuse" OR "psychological abuse" OR "emotional abuse" OR "economic abuse" OR "financial abuse" OR "verbal abuse" OR maltreatment OR "violent discipline" OR "corporal punishment" OR "adverse childhood experience" OR "adverse childhood experiences" OR molestation OR "child abuse" OR "partner abuse" OR "dating abuse" OR "wife abuse" OR "spouse abuse" OR "domestic abuse" OR "elder abuse" OR "senior abuse" OR "aged abuse" OR victimization OR dehumanization OR victimisation OR dehumanisation OR stalking)) OR (ab:(violence OR "sexual assault" OR "sexual harassment" OR "sexual abuse" OR "sex abuse" OR rape OR "forced sex" OR "sexual coercion" OR "reproductive coercion" OR "sex trafficking" OR "sexual exploitation" OR "forced marriage" OR "forced marriages" OR "child marriage" OR "child marriages" OR "early marriage" OR "early marriages" OR "child bride" OR "child brides" OR CEFM OR "female genital mutilation" OR "female genital cutting" OR "female circumcision" OR "female genital circumcision" OR infibulation* OR clitoridectom* OR clitorectom* OR "ritual female genital surgery" OR "ritual female genital surgeries" OR FGM OR "physical abuse" OR "psychological abuse" OR "emotional abuse" OR "economic abuse" OR

"financial abuse" OR "verbal abuse" OR maltreatment OR "violent discipline" OR "corporal punishment" OR "adverse childhood experience" OR "adverse childhood experiences" OR molestation OR "child abuse" OR "partner abuse" OR "dating abuse" OR "wife abuse" OR "spouse abuse" OR "domestic abuse" OR "elder abuse" OR "senior abuse" OR "aged abuse" OR victimization OR dehumanization OR victimisation OR dehumanisation OR stalking OR cyberviolence OR cybervictimization OR cyberstalking)))
AND
((mh:(E05.318.372.500.500* OR E05.318.370.150 OR E05.318.372.500.750* OR V03.850 OR L01.178.682.759.575 OR V03.600 OR E05.318.370.500* OR V03.900)) OR (ti:("systematic review" OR "meta-analysis" OR cohort OR "cross-over" OR "case-control" OR Prospective OR retrospective OR longitudinal OR "follow-up" OR followup)) OR (ab:("systematic review" OR "meta-analysis" OR cohort OR "cross-over" OR "case-control" OR Prospective OR retrospective OR longitudinal OR "follow-up" OR followup)))
AND
((mh:9E05.318.740* OR E05.318.740.600.800* OR E05.318.740.600.600)) OR (ti:(risk* OR odds OR "cross-product ratio" OR "cross-product ratios" OR "hazards ratio" OR "hazards ratios" OR "hazard ratio" OR "hazard ratios" OR statistic* OR "HR" OR "RR" OR "aOR" OR relation* OR correlat* OR associat* OR likel*) OR (ab:(risk* OR odds OR "cross-product ratio" OR "cross-product ratios" OR "hazards ratio" OR "hazards ratios" OR "hazard ratio" OR "hazard ratios" OR statistic* OR "HR" OR "RR" OR "aOR" OR relation* OR correlat* OR associat* OR likel*)))
)
AND
(year_cluster:[1970 TO 2024])

Cochrane Search String

([mh "Sex Offenses"] OR [mh ^"Violence"] OR [mh "Domestic Violence"] OR [mh "Gender-Based Violence"] OR [mh "Intimate Partner Violence"] OR [mh "Physical Abuse"] OR [mh "Rape"] OR [mh "Torture"] OR [mh "Workplace Violence"] OR [mh "Gun violence"] OR [mh "Battered Women"] OR [mh "Adult Survivors of Child abuse"] OR [mh "Exposure to Violence"] OR [mh "Emotional Abuse"] OR [mh "Sexual Harassment"] OR [mh ^"Harassment, Non-Sexual"] OR [mh ^"Aggression"] OR [mh "Coercion"] OR [mh "Dehumanization"] OR [mh "stalking"] OR [mh “adverse childhood experiences”] OR (violence OR "sexual assault" OR "sexual harassment" OR "sexual abuse" OR "sex abuse" OR rape OR "forced sex" OR "sexual coercion" OR "reproductive coercion" OR "sex trafficking" OR "sexual exploitation" OR ((forced OR child OR early) NEXT marriage*) OR (child NEXT bride*) OR CEFM OR "female genital mutilation" OR "female genital cutting" OR "female circumcision" OR "female genital circumcision" OR infibulation* OR clitoridectom* OR clitorectom* OR "ritual female genital surgery" OR "ritual female genital surgeries" OR FGM OR ((physical OR psychological OR emotional OR economic OR financial OR verbal) NEXT abuse) OR maltreatment OR "violent discipline" OR "corporal punishment" OR "adverse childhood experience" OR "adverse childhood experiences" OR molestation OR "child abuse" OR "partner abuse" OR "dating abuse" OR "wife abuse" OR "spouse abuse" OR

"domestic abuse" OR "elder abuse" OR "senior abuse" OR "aged abuse" OR victimization OR dehumanization OR victimisation OR dehumanisation OR stalking OR cyberviolence OR cybervictimization OR cyberstalking):ti,ab,kw)
AND
([mh "Case-Control Studies"] OR [mh "Cross-Over Studies"] OR [mh "Cohort Studies"] OR [mh "Systematic Review"] OR [mh "Meta-Analysis"] OR [mh "Twin Study"] OR ("systematic review" OR "meta-analysis" OR "cohort" OR "cross-over" OR "case-control" OR "prospective" OR "retrospective" OR "longitudinal" OR "follow-up" OR "followup"):ti,ab,kw)
AND
([mh "Statistics as Topic"] OR [mh Risk] OR [mh "Odds Ratio"] OR ("risk" OR "odds" OR "cross-product ratio" OR "cross-product ratios" OR "hazards ratio" OR "hazards ratios" OR "hazard ratio" OR "hazard ratios" OR statistic* OR "HR" OR "RR" OR "aOR" OR relation* OR correlat* OR associat* OR likel*):ti,ab,kw)
Limits: January 1970 – January 2024

Web of Science Core Collection Search String

(TS=(
(violence OR "sexual assault" OR "sexual harassment" OR "sexual abuse" OR "sex abuse" OR rape OR "forced sex" OR "sexual coercion" OR "reproductive coercion" OR "sex trafficking" OR "sexual exploitation" OR "forced marriage*" OR “forced marriage*” OR “child marriage*” OR “early marriage*” OR “child bride*” OR CEFM OR "female genital mutilation" OR "female genital cutting" OR "female circumcision" OR "female genital circumcision" OR infibulation* OR clitoridectom* OR clitorectom* OR “ritual female genital surger*” OR FGM OR "physical abuse" OR "psychological abuse" OR "emotional abuse" OR "economic abuse" OR “financial abuse” OR "verbal abuse" OR Maltreatment OR torture OR "violent discipline" OR "corporal punishment" OR "adverse childhood experience*" OR molestation OR "child abuse" OR "partner abuse" OR "dating abuse" OR "wife abuse" OR "battered wom*n" OR "spouse abuse" OR "domestic abuse" OR "elder abuse" OR "senior abuse" OR "aged abuse" OR victimization OR dehumanization OR victimisation OR dehumanisation OR stalking OR cyberviolence OR cybervictimization OR cyberstalking)
AND
("systematic review" OR "meta-analysis" OR cohort OR cross-over OR case-control OR prospective OR retrospective OR longitudinal OR follow-up OR followup)
AND
("risk*" OR "odds" OR "cross-product ratio*" OR "hazards ratio*" OR "hazard ratio*" OR statistic* OR "HR" OR "RR" OR "aOR" OR relation* OR correlat* OR associat* OR likel*)
))
AND
DOP=(1970-01-01/2024-01-31)

Section 7.2: Inclusion and exclusion criteria

Each review step (title/abstract screening, full-text screening, and data extraction) began with consensus building exercises across the review team. After training and consensus-building, the first two-thirds of titles/abstracts were reviewed by two independent reviewers, with conflicts resolved by project leaders. Upon confirmation of a low rate of total conflicts (<5% of total screened), the remainder of titles/abstracts were single screened. Non-English articles were screened by reviewers with proficiency in the language. Studies which met inclusion criteria during title/abstract screening were full text screened and excluded if found to meet any exclusion criteria. Two independent reviewers full text screened 10% of articles, with conflicts resolved by project leads. Upon confirming a low conflict rate (<5%), the remaining 90% of articles were single screened.

Table S24: Inclusion and exclusion criteria applied during screening.

Inclusion Criteria	Exclusion Criteria
**Study design:** case–control, cohort, or case-crossover studies.<br><br>**Participants:** Studies conducted in participant groups likely to be generalizable to the population of interest. Exposed groups are defined as any individual who has experienced a form of childhood physical abuse, childhood psychological abuse, and childhood neglect. Comparators will be non-exposed control groups, or study groups without reported exposure to a form of childhood physical abuse, childhood psychological abuse, and/or childhood neglect.<br><br>**Outcomes:** Studies reporting an estimate of association (either RR, risk ratio, odds ratio, hazard ratio or similar) or reporting cases and non-cases among those exposed and unexposed. If not provided directly, studies providing enough information to allow an estimate of RR to be calculated will meet inclusion criteria.	**Study design**: Cross-sectional, ecological, case series or case studies.<br><br>**Participants**: Studies conducted in subgroups identified only by convenience sampling or subgroups identified via a shared characteristic that is likely related to risk of exposure to violence or the reported health outcome (e.g., domestic violence shelter residents).<br><br>**Exposure measurement**: Studies that report only an aggregate measure of exposure combining exposure to a form of violence with other, non-eligible exposures (e.g., reports a composite score only) will be excluded. For these studies, we are unable to disentangle the effect of violence exposure from the effects of other hardships or exposure types, preventing their inclusion in our review.<br><br>**Does not meet minimum reporting criteria**: Studies missing essential data, that is, those that do not report effect sizes and uncertainty information (confidence intervals, sample sizes) or the data needed to impute an effect size with uncertainty information.

After the screening and extraction of articles identified, we introduced another layer of inclusion and exclusion criteria applied to data cleaning that pertain to our definitions of included risk factors (Table S23). Studies that met this set of criteria (n = 86) during data cleaning were applied into the models for final results.

Table S25: Inclusion and exclusion criteria applied during data cleaning.

Inclusion Criteria	Exclusion Criteria
**Exposure age:** Observations where the age of violence exposure is under 18 years old (childhood exposure).<br><br>**Studies with multiple forms of observations:** Violence exposure observation definitions that precisely match to the exact definitions of individual risk factors of childhood physical abuse, childhood psychological abuse, or childhood neglect (i.e. non-combination forms of exposure).<br><br>**Outcome definitions:** Exact outcome definitions to GBD outcome of interest and/or deviations to outcome definition without including other outcomes.	**Exposure age:** Observations where the age of violence exposure is 18 years or older (adulthood exposure).<br><br>**Studies with multiple forms of observations:** Violence exposure observation definitions that combine more than one form of violence exposure (i.e. exposure to childhood physical and/or psychological violence).<br><br>**Outcome definitions:** Aggregate outcome definitions that are non-GBD outcomes and/or include other health conditions.

Section 7.3: Data extraction

All studies were extracted using a modified data extraction template in Covidence data extraction 2.0. Table S24 details the fields extracted for each article.

Table S26: Data extraction template

Name		Definition
**Study locations**	**Location Name**	The country or IHME sub-national location where the study took place
	**Location ID**	The location ID corresponding to the country or IHME sub-national location of the study
**Specific Location**		Required if the study took place in a location smaller than the corresponding location name and ID
**Study Name**		Required if cohort is named, do not use the article title. This field is used to screen out duplicative data, so please ensure spelling is correct.
**Year Start**		Year the study started
**Year End**		Year the study ended (NOT publication year)

**Study Design**	**Prospective cohort**	Must select one
	**Retrospective cohort**	
	**Case-control**	
	**Case-cohort**	
	**Case-crossover**	
**Pooled cohort**		Cohort studies only: Yes if the reported effect size is from a pooled analysis and only pooled effect size has been reported, otherwise no
**Study Selection Criteria**		Please specify the selection criteria of the study that is used in the analysis
**Location Representative**		Specify if the participants were representative of the study's geography or not
**Cohort Study: Drop-out rate**		Study dropout rate (%) at the end of the study entered as a decimal
**Cohort Study: Drop-out rate assessment**		Specify how dropout rate was defined in the study.
**Cohort Study: Follow-up measure**		Cohort studies only: Type of follow up measure reported (eg, 'average participant follow-up was 126 days' then select 'mean').
**Cohort Study: Follow-up units**		Cohort studies only: enter units of follow-up duration reported (eg, 'average participant follow-up was 126 days' then enter 'days').
**Cohort Study: Follow-up value**		Cohort studies only: Enter the length of participant follow-up if reported (eg, 'average participant follow-up was 126 days' then enter 126).
**Case-control Study: Percent of participants for which data ascertained**		Percent of participants (%) from total, for which the study has included data entered as a decimal
**Case-control Study: Controls selected from community**		Were the controls selected from the community? Yes or No
**Exposure assessment method**		Self-report, routinely collected/ administrative data, clinical examination
**Exposure assessment instrument**		Specify the name of the exposure assessment instrument. For self-reported exposures, please specify the name of the questionnaire. If more than one instrument, specify all. If the instrument is not names/designed specifically for the study, write "study-specific instrument"
**Exposure assessment period**		How many times information on exposure to type of violence
**Exposure assessment value**		If "exposure assessment period" is multiple, specify the number of times that exposure was assessed (excluding baseline)
**Outcome assessment method**		Select how the study ascertained which participants experienced the outcome
**Outcome assessment instrument**		Specify the name of the outcome assessment instrument. For self-reported outcomes, please specify the name of the questionnaire.If more than one

	instrument, specify all. If the instrument is not names/designed specifically for the study, write “study-specific instrument”
**Effect size measure**	Select the form of effect size used in the study
**Uncertainty type**	
**Confidence interval level**	If uncertainty is reported as a confidence interval, this column represents the confidence level which is reported at (Eg. 95, 90, 99).
**Extractor notes**	Please use this field to include any notes about the study or your extraction not covered elsewhere.
**Outcome name**	Outcome that is measured in this model
**Outcome type**	Please specify if the outcome definition included incidence of or mortality from a disease endpoint.
**Outcome definition**	Please specify the definition for the outcome as reported in the study.
**Exposure definition**	Please specify the definition for the exposed participants exactly as reported in the study.
**Exposed level**	Enter level of exposure defined for exposed group. If study's exposure is binary (any vs. none), select 'any exposure' for exposed. For all other exposure levels, select other and enter level as reported (eg, 'exposed 3-5 times').
**Unexposed definition**	Provide a brief description of the unexposed group (i.e., the comparison group) as used in estimation of the relative risk
**Unexposed level**	Enter level of exposure defined for unexposed (comparator group). If the study's exposure is binary (any vs. none), select 'no exposure' for unexposed. For all other exposure levels, select other and enter level as reported (eg, 'exposed 1-2 times').
**Violence type**	Select all violence types included for this model
**Violence type combination**	Select HOW the violence types are combined in the model.<br>(1) AND: the model must specify that the exposure group experienced BOTH/ALL types of violence selected in the previous question<br>(2) AND/OR: the exposure group consists of people who experience EITHER type of violence<br>(3) ONLY: model specifies that the exposure group experienced selected violence type, but not other types of violence included in the study.<br><br>*NOTE: Use 'only' option when it has been confirmed that is the ONLY type of violence a participant experienced (eg, experienced sexual but NOT physical violence). Otherwise, if one type of violence assessed and exposure to others are unknown, use 'unknown'.*
**Perpetrator type**	Select all perpetrator types included in this model

**Temporality of Exposure**	**Lower (Age)**	Lower bound of age range provided of when participants experienced violence. If no lower age is provided, fill in this box with '0' and provide more information in the "other information" question for this model (e.g. "temporality of exposure defined as "Childhood and adolescence")
	**Upper (Age)**	Upper bound of age range provided of when participants experienced violence. If no upper age is provided, fill in this box with "99" and provide more information in the "other information" question for this model (e.g. "temporality of exposure defined as "Childhood and adolescence")
**Exposure Recall Type**		Specify whether the exposure occurred throughout lifetime, past year, or indicate other recall type.
**Percent Female**		For the sample of this model, what percent are female (0-1) on a per 1 basis (eg, 43% female should be recorded as 0.43). Enter 1 if sample is only female. Enter 0 if sample is only male. If sample includes both sexes and percent female not reported, enter 99.
**Ages**	**Lower**	Lower bound of age of participants included in this model at time of study. If the model includes participants of all ages from the study,this box will match the lower age box in section 2.
	**Upper**	Upper bound of age of participants included in this model at time of study. If the model includes participants of all ages from the study,this box will match the upper age box in section 2.
	**Mean**	Mean of age of participants included in this model.
	**SD**	SD of age of participants included in this model.
**Subgroup analysis**		**PER UNIQUE RISK-OUTCOME PAIR IN A STUDY:** Yes if this effect size is a sub-analysis reported IN ADDITION TO a main analysis from all participants (eg, study reports effect size for combined sexes and also effect sizes separately for males and females). If study only reports effect sizes from specific subgroups (eg, reports effect sizes from males and females separately, without reporting a combined effect size), select no.
**Subgroup analysis free text**		If a sub-analysis, describe stratifier (i.e. age, sex, etc.)
**Effect size**	**Mean**	Mean effect size of model
	**Lower (UI)**	Lower bound of effect size
	**Upper (UI)**	Upper bound of effect size
	**Other uncertainty value**	
**Effect size table #**		Table number where you found effect size from literature
**Sample size**	**Number of cases**	Enter number of participants with measured outcome for each group reported: exposed, unexposed, total

	**Number of participants**	Enter number of participants included in analysis for each group reported: exposed, unexposed, total
	**Person-time (cohort studies)**	Enter person-time for each group reported: exposed, unexposed, total
**Person-time units (cohort studies)**		If cohort study and person-time entered into sample size table, specify the units of person-time reported
**Confounders**		Select all confounders for the most adjusted model, write in any confounders (separated by comma) not included in pre-specified list
**Other information**		Any additional information

Section 7.4: Systematic review and meta-analysis citation searching

We title and abstract and full text screened systematic reviews and/or meta-analyses yielded by our searches according to the criteria outlined for all other articles. We then grouped accepted systematic reviews/meta-analyses by unique risk-outcome pair combination in order to extract the citations identified by these reviews. If more than one systematic review was identified for a given risk-outcome pair, one systematic review was selected for citation searching on the basis of publication recency, number of included underlying studies and study quality. Study quality was determined via adherence to PRISMA and GATHER guidelines and by the impact factor of the journal the study was published within.

Once a single systematic review was selected for citation searching per unique risk-outcome pair, we extracted the references identified within each review and de-duplicated them against our primary search records. All new articles from this search were then screened for inclusion with the same criteria as in our larger review.

Across all risk outcome pairs, this citation-searching step of our review yielded 57 new studies for extraction.

Section 8: Study Quality and Bias Assessment

Section 8.1: Definitions of bias covariates

Following GRADE criteria, the risk of bias criteria for individual studies included in our analyses captured representativeness of the study population, exposure and outcome measurement quality, control for confounding, selection bias, and risk of reverse causation. Because our analyses covered three distinct risk factors and many different health outcomes, we created a core set of bias covariates across all risk-outcomes pairs (Table S25) as well as additional bias covariates specific to effect size adjustment, exposure definition, and outcome definitions (Table S26; Table 27; Table S28). For all covariates, the reference value is zero while indication of the specific bias type was coded as a one. All covariates meeting eligibility requirements (i.e., at least two studies represented for each value of the covariate) were tested for significance using the selection algorithm in the MR-BRT tool. Minimum availability of two observations for each value of the covariate meant that a reduced set of covariates were able to be tested for risk-outcome pairs with low total study counts.

Table S27. Standard bias covariates created across all input datasets.

**Bias covariate name**	**Definition**	**Operationalized definition**
Representativeness	Study sample is not geographically representative of underlying location	0 = is representative;<br>1 = not representative
Risk of selection bias	Study is at risk for selection bias with loss to follow-up (cohorts) or percent without ascertained data (case-controls) > 20%	0 = no selection bias;<br>1 = selection bias risk
Risk of reverse causation	Study is at risk of reverse causation, as in the case with case-control studies	0 = no risk of reverse causation;<br>1 = risk present
Odds ratio	Study reports an odds ratio	0 = study reports relative risk<br>1 = study reports odds ratio

Table S28. Adjustment bias covariates created across all input datasets.

**Bias covariate name**	**Definition**	**Operationalized definition**
Unadjusted effect size	Study reports only a complete unadjusted effect size	0 = controls for any confounding;<br>1 = raw/crude effect size
Unadjusted for sex	Effect size is controlled for sex or is sex-specific	0 = accounts for sex;<br>1 = does not account for sex
Unadjusted for age	Effect size is controlled for age	0 = accounts for age;<br>1 = does not account for age
Unadjusted for age, sex, and at least one other confounding variable	Effect size controls for age, sex, and at least one other confounder	0 = controls for age, sex, and at least one other confounder;<br>1 = does not control for age or sex or at least one other confounder
Males included in the effect size	Does the effect size include males in its analytical sample?	0 = does not include males;<br>1 = includes males

Females only included in the effect size	Does the effect size only include females in its analytical sample?	0 = does not include females; 1 = includes females only
Effect size for both sexes combined	Does the effect size use an analytical sample that includes both sexes?	0 = not sex-specific effect size; 1 = sex-specific effect size

Table S29. Exposure definition bias covariates.

**Bias covariate name**	**Definition**	**Operationalized definition**
Family/household perpetrator	Is the perpetrator group limited to specifically family/household members or specifically non-family/household members?	0 = broader perpetrator group; 1 = restricted perpetrator group
Exposure defined as being younger than age 18	Is the exposure defined as exposure to violence during any point in childhood or at a younger group?	0 = upper age of exposure is 18; 1 = upper age of exposure is smaller than 18
Exposure defined including ages above 15	Is the exposure defined as exposure to violence during any point at ages above 15 years old?	0 = upper age of exposure is below 15 1 = upper age of exposure is above 15
Exposure defined as below age 15	Is the exposure defined as exposure to violence during any point at ages below 15 years old?	0 = upper age of exposure is above 15 1 = upper age of exposure is below 15
Exposure is ascertained from administrative source	Captures if exposure was ascertained from administrative databases (health systems, agency records, etc.), since many children experiencing abuse would not be reported/investigated through official channels	0 = self-reported data; 1 = used administrative source for exposure ascertainment

Table S30. Outcome definition bias covariates.

**Bias covariate name**	**Outcome**	**Definition**	**Operationalized definition**
Outcome is defined including other disorders	Major depressive disorder	What outcome definition does the study use?	0 = definition does not include these other disorders; 1 = definition includes bipolar, anxiety, and other mood disorders
Outcome is defined as a specific disorder	Diabetes Mellitus		0 = outcome is not a defined type of diabetes; 1 = outcome is type 1 or type 2 diabetes

	Substance use disorder; Drug use disorder; Alcohol use disorder; Drug use disorder sub-types		0 = outcome is dependence<br>1 = outcome is not limited to dependence
Outcome is defined as PTSD	Anxiety disorders		0 = outcome is not specifically PTSD<br>1 = outcome is PTSD
Outcome is defined as induced abortion	Maternal abortion and/or miscarriage		0 = outcome is miscarriage or unspecified abortion<br>1 = outcome is induced abortion

Section 8.2: Bias covariates for each risk-outcome pairs

We report the bias covariates marked and tested for each risk-outcome pair in Tables S29-S39. The value 1 indicates that the bias covariate applies to the study for that risk-outcome pair; 0 indicates that the bias covariate does not apply to the study for that risk-outcome pair; NA indicates that the bias covariate was not tested for that study for that risk-outcome pair.

Table S31. Bias Covariates for childhood physical abuse and corresponding outcomes.

**Study**	**Covariates**															
**Physical abuse & Major depressive disorder**																
	Representative	Risk of reverse causation	Risk of selection bias	Uncontrolled	Unadjusted for age	Family or household perpetrator	Exposure including ages above 15	Exposure limited to below 15	Effect size for women and men combined	Unadjusted for sex	Effect size includes men	Unadjusted for age, sex, and at least one other confounder	Aggregate outcome definition	Odds ratio	Administrative exposure ascertainment	Women-specific study
Xiang 2021	0	0	0	0	0	1	1	0	0	0	1	0	1	0	0	0
Xiao 2022	0	1	0	1	1	0	0	0	0	1	1	1	1	1	0	0
Su 2022	1	0	1	0	1	0	1	0	0	1	1	1	0	0	0	0
Merza 2015	1	1	0	1	1	1	1	0	0	1	1	1	0	1	0	0
Li 2024	0	0	0	0	0	0	1	0	0	0	1	0	1	1	0	0
Bhattarai 2023	0	0	0	0	0	1	1	0	0	0	1	0	1	1	0	0
Kang 2023	1	1	0	1	1	0	1	0	0	1	1	1	0	1	0	0
Yu 2023	0	0	1	0	0	1	1	0	0	0	1	0	1	1	0	0
Widom 2023	1	1	1	0	0	0	0	1	0	0	1	1	1	1	1	0

Elbarazi 2023	1	0	0	1	0	0	1	0	0	1	1	1	1	1	0	0
Fergusson 2008	0	0	0	0	0	1	1	0	0	0	1	0	0	1	0	0
Roustit 2009	1	0	1	0	0	0	1	0	0	0	1	0	1	1	0	0
Widom 2007	1	0	1	1	1	0	0	1	0	1	1	1	0	1	1	0
Andrews 1995	1	0	1	1	1	1	1	0	1	0	0	1	1	1	0	1
Brown 1999	1	0	0	0	0	0	1	0	0	0	1	0	1	1	0	0
Wise 2001	0	1	0	0	0	0	1	0	1	0	0	0	1	0	0	1
Mullen 1996	1	0	1	1	1	1	1	0	1	0	0	1	1	1	0	1
Comijs 2013	1	1	0	0	0	0	1	0	0	0	1	0	1	1	0	0
Kisely 2021	0	0	1	0	0	0	1	0	0	0	1	0	1	1	0	0
Ebert 2019	1	0	1	0	1	0	1	0	0	1	1	1	0	1	0	0
Danese 2023	1	0	1	1	1	0	0	1	0	1	1	1	1	0	0	0
Danese 2023	1	0	1	1	1	0	0	1	0	1	1	1	1	0	0	0
Danese 2023	1	0	1	1	1	0	0	1	0	1	1	1	1	0	0	0
Zhou 2023	1	1	0	0	1	0	1	0	1	0	0	1	0	1	0	0
Zhou 2023	1	1	0	0	1	0	1	0	1	0	1	1	0	1	0	0
Chapman 2004	1	0	1	0	1	0	1	0	1	0	0	1	1	1	0	0
Chapman 2004	1	0	1	0	1	0	1	0	1	0	1	1	1	1	0	0

Hovens 2015	1	0	0	0	0	0	1	0	0	0	1	0	1	1	0	0
Hovens 2015	1	0	0	0	0	0	1	0	0	0	1	0	1	1	0	0
Galloeag 2017	0	0	1	0	0	1	0	0	1	0	0	0	0	1	0	0
Galloeag 2017	0	0	1	0	0	1	0	0	1	0	1	0	0	1	0	0
Houtepen 2020	0	0	0	0	0	1	0	0	0	0	1	0	1	1	0	0

Physical abuse & Diabetes mellitus type 2

	Representative	Risk of selection bias	Unadjusted	Unadjusted for age	Family or household perpetrator	Exposure including ages above 15	Exposure limited to below 15	Effect size for women and men combined	Alternative outcome definition	Unadjusted for sex	Effect size includes men	Unadjusted for age, sex, and at least one other confounder	Odds ratio	Women-specific study
Afifi 2013	0	0	0	0	1	1	0	0	0	0	1	0	1	0
Monnat 2015	0	0	0	0	0	1	0	0	0	0	1	0	1	0
Njoroge 2023	1	1	1	1	0	1	0	0	0	1	1	1	1	0
Gaston 2023	0	0	0	0	0	1	0	1	1	0	0	0	0	1
Widom 2023	1	1	0	0	0	0	1	0	0	0	1	1	1	0
Thomas 2008	0	1	0	0	1	1	0	0	1	0	1	0	1	0
Kascakova 2022	0	0	0	1	0	1	0	0	0	1	1	1	1	0
Kascakova 2022	0	0	0	1	0	1	0	0	0	1	1	1	1	0

Shields 2016	0	0	0	0	0	1	0	0	0	0	1	0	1	0
Shields 2016	0	0	0	0	0	1	0	0	0	0	1	0	1	0
Zhu 2023	0	0	0	0	0	1	0	1	0	0	1	0	1	0
Zhu 2023	0	0	0	0	0	1	0	1	0	0	0	0	1	0
Rich-Edwards 2010	0	1	0	0	1	1	0	1	1	0	0	0	0	1
Rich-Edwards 2010	0	1	0	0	1	1	0	1	1	0	0	0	0	1
Rich-Edwards 2010	0	1	0	0	1	1	0	1	1	0	0	0	0	1
Duncan 2015	0	0	0	0	1	0	1	1	0	0	0	0	1	0
Duncan 2015	0	0	0	0	1	0	1	1	0	0	0	0	1	0
Duncan 2015	0	0	0	0	1	0	1	1	0	0	1	0	1	0
Duncan 2015	0	0	0	0	1	0	1	1	0	0	1	0	1	0
Lown 2019	0	1	1	1	0	1	0	1	1	0	0	1	1	0
Lown 2019	0	1	1	1	0	1	0	1	1	0	1	1	1	0
**Physical abuse & Self-harm**														
	Representative	Risk of reverse causation	Risk of selection bias	Unadjusted	Unadjusted for age	Family or household perpetrator	Exposure including ages above 15	Exposure limited to below 15	Effect size for women and men combined	Unadjusted for sex	Effect size includes men	Unadjusted for age, sex, and at least one other	Odds ratio	Administrative exposure ascertainment

												confounder		
Kiselydmedres 2022	1	0	1	0	0	0	0	0	1	0	0	0	1	0
Salzinger 2007	1	0	1	1	0	0	0	1	0	1	1	1	1	1
Archambault 2023	0	0	1	0	0	0	0	1	0	0	1	0	0	0
Geng 2023	1	1	0	1	1	0	1	0	0	1	1	1	1	0
Calegaro 2023	0	0	1	0	0	1	0	1	0	0	1	0	1	0
Bhattarai 2023	0	0	0	0	0	1	1	0	0	0	1	0	1	0
Roustit 2009	1	0	1	0	0	0	1	0	0	0	1	0	1	0
Brown 1999	1	0	0	0	0	0	1	0	0	0	1	0	1	0
Mullen 1996	1	0	1	1	1	1	1	0	1	0	0	1	1	0
Enns 2006	0	0	1	0	0	0	1	0	0	0	1	0	1	0
Johnson 2002	1	0	1	0	0	0	0	1	0	0	1	0	1	1
Thompson 2019	0	0	1	0	0	1	1	0	0	0	1	0	1	0
Russell 2019	1	0	0	0	1	0	0	1	0	0	1	1	0	0
Bruffaerts 2010	0	0	1	0	0	0	1	0	0	0	1	0	1	0
Bruffaerts 2010	0	0	1	0	0	0	1	0	0	0	1	0	1	0
Fried 2013	0	0	0	1	1	1	0	1	0	1	1	1	1	0
Fried 2013	0	0	0	1	1	1	0	1	0	1	1	1	1	0

Rajapakse 2020	1	1	0	0	0	1	1	0	0	0	1	0	1	0

**Physical abuse & Drug use disorders**										
	Representative	Risk of selection bias	Unadjusted for age	Family or household perpetrator	Exposure including ages above 15	Exposure limited to below 15	Effect size for women and men combined	Alternate outcome definition	Effect size includes men	Unadjusted for age, sex, and at least one other confounder
Scheidell 2018	0	0	0	1	1	0	0	1	1	0
Duncan 2008	1	1	1	0	1	0	0	0	1	1
Cohen 2001	0	0	0	1	1	0	0	1	1	0
Huang 2011	0	1	0	1	0	1	0	1	1	0
Kisely 2021	1	0	0	0	1	0	0	1	1	0
Najman 2022	0	1	0	0	1	0	0	0	1	0
Najman 2022	0	1	0	0	1	0	0	0	1	0
Telfar 2023	0	0	1	1	1	0	0	0	1	1
Telfar 2023	0	0	1	1	1	0	0	0	1	1
Telfar 2023	0	0	1	1	1	0	0	0	1	1
Broekhof 2023	0	1	0	0	1	0	1	0	0	1
Broekhof 2023	0	1	0	0	1	0	1	0	1	1
Libby 2004	1	1	0	1	0	1	0	0	1	0
Libby 2004	1	1	0	1	0	1	0	0	1	0

Conroy 2009	1	0	0	0	1	0	1	0	1	0
Conroy 2009	1	0	0	0	1	0	1	0	0	0
Tanaka 2015	0	1	0	0	1	0	1	1	0	0
Tanaka 2015	0	1	0	0	1	0	1	1	0	0
Tanaka 2015	0	1	0	0	1	0	1	1	1	0
Tanaka 2015	0	1	0	0	1	0	1	1	1	0

Physical abuse & Anxiety disorders

	Representative	Risk of selection bias	Unadjusted	Unadjusted for age	Family or household perpetrator	Exposure including ages above 15	Exposure limited to below 15	Unadjusted for age, sex, and at least one other confounder	PSTD outcome
Tenhave 2019	0	1	0	0	0	1	0	0	0
Bhattarai 2023	0	0	0	0	1	1	0	0	0
Yu 2023	0	1	0	0	1	1	0	0	0
Elbarazi 2023	1	0	1	0	0	1	0	1	0
Fergusson 2008	0	0	0	0	1	1	0	0	0
Widom 1999	1	1	1	1	0	0	1	1	1
Fujiwara 2011	1	1	0	0	0	1	0	0	0
Raposo 2014	0	0	0	1	1	1	0	1	0
Danese 2023	1	1	1	1	0	0	1	1	0
Danese 2023	1	1	1	1	0	0	1	1	0

Danese 2023	1	1	1	1	0	0	1	1	0
Hovens 2015	1	0	0	0	0	1	0	0	0
Hovens 2015	1	0	0	0	0	1	0	0	0
Kisely 2021	0	1	0	0	0	1	0	0	0
Kisely 2021	0	1	0	0	0	1	0	0	1

**Physical abuse & Alcohol use disorder**							
	Representative	Risk of selection bias	Family or household perpetrator	Effect size for women and men combined	Unadjusted for sex	Effect size includes men	Unadjusted for age, sex, and at least one other confounder
Fenton 2013	0	1	1	0	0	1	0
Roustit 2009	1	1	0	0	0	1	0
Kascakova 2022	0	0	0	0	1	1	1
Kascakova 2022	0	0	0	0	1	1	1
Najman 2022	0	1	0	0	0	1	0
Najman 2022	0	1	0	0	0	1	0
Telfar 2023	0	0	1	0	1	1	1
Telfar 2023	0	0	1	0	1	1	1
Telfar 2023	0	0	1	0	1	1	1
Broekhof 2023	0	1	0	1	0	0	1
Broekhof 2023	0	1	0	1	0	1	1

Libby 2004	1	1	1	0	0	1	0
Libby 2004	1	1	1	0	0	1	0
Tanaka 2015	0	1	0	1	0	1	0
Tanaka 2015	0	1	0	1	0	0	0
Tanaka 2015	0	1	0	1	0	0	0
Tanaka 2015	0	1	0	1	0	1	0

Physical abuse & Asthma

	Representative	Unadjusted for age	Exposure including ages above 15	Exposure limited to below 15	Unadjusted for sex
Njoroge 2023	1	1	1	0	1
Sun 2024	0	0	1	0	0
Abajobir 2017	1	0	0	1	1
Kascakova 2022	0	1	1	0	1
Kascakova 2022	0	1	1	0	1
Han 2022	0	0	1	0	0
Coogan 2013	0	0	1	0	0
Coogan 2013	0	0	0	1	0

Physical abuse & Gynecological disorders

	Risk of selection bias	Family or household perpetrator	Exposure including ages above 15	Exposure limited to below 15	Odds ratio
Harris 2018	1	1	1	0	0
Liebermann 2018	1	0	1	0	1

Ito 2021	1	1	1	0	1
Harlow 2005	1	0	0	1	1
Bertone-Johnson 2014	0	1	0	1	1
Boynton-Jarrett 2011	0	0	1	0	0

Physical abuse & Migraines

	Effect size for women and men combined	Unadjusted for sex	Effect size includes men
Gelaye 2016	1	0	0
Karmakar 2017	0	1	1
Kascakova 2022	0	1	1
Kascakova 2022	0	1	1
Brennenstuhl 2015	1	0	1
Brennenstuhl 2015	1	0	0

Physical abuse & Eating disorders

	Risk of selection bias
Rayworth 2004	0
Andrews 1995	1
Mullen 1996	1
Talmon 2021	0
Talmon 2021	0

Physical abuse & Ischemic heart disease	
	Representative
Monnat 2015	0
Dong 2004	1
Akasaki 2021	1
Kascakova 2022	0
Kascakova 2022	0

Physical abuse & Schizophrenia	
	Family or household perpetrator
Trotta 2023	1
Alkema 2023	0
Mansueto 2022	0
Mansueto 2022	0
Chatziioannidis 2019	1
Chatziioannidis 2019	1

Table S32. Bias Covariates for childhood psychological abuse and corresponding outcomes.

**Study**	**Covariates**										
**Psychological abuse & Major depressive disorder**											
	Representative	Risk of reverse causation	Risk of selection bias	Unadjusted	Unadjusted for age	Family or household perpetrator	Exposure including ages above 15	Effect size for women and men combined	Unadjusted for sex	Effect size includes men	Unadjusted for age, sex, and at least one other confounder	Aggregate outcome definition
Xiao 2022	0	1	0	1	1	0	0	0	1	1	1	1
Su 2022	1	0	1	0	1	0	1	0	1	1	1	0
Kang 2023	1	1	0	1	1	0	1	0	1	1	1	0
Zhang 2023	0	0	1	0	0	1	1	0	0	1	0	1
Yu 2023	0	0	1	0	0	1	1	0	0	1	0	1
Elbarazi 2023	1	0	0	1	0	0	1	0	1	1	1	1
Roustit 2009	1	0	1	0	0	1	1	0	0	1	0	1
Mullen 1996	1	0	1	1	1	1	1	1	0	0	1	1
Comijs 2013	1	1	0	0	0	0	1	0	0	1	0	1
Kisely 2021	0	0	1	0	0	0	1	0	0	1	0	1
Ebert 2019	1	0	1	0	1	0	1	0	1	1	1	0
Zhou 2023	1	1	0	0	1	0	1	1	0	0	1	0
Zhou 2023	1	1	0	0	1	0	1	1	0	1	1	0

Chapman 2004	1	0	1	0	1	1	1	1	0	0	1	1
Chapman 2004	1	0	1	0	1	1	1	1	0	1	1	1
Vaeth 2010	0	0	1	0	0	1	1	1	0	0	0	1
Vaeth 2010	0	0	1	0	0	1	1	1	0	1	0	1
Hovens 2015	1	0	0	0	0	0	1	0	0	1	0	1
Hovens 2015	1	0	0	0	0	0	1	0	0	1	0	1
Galloeag 2017	0	0	1	0	0	1	0	1	0	0	0	0
Galloeag 2017	0	0	1	0	0	1	0	1	0	1	0	0
Houtepen 2020	0	0	0	0	0	1	0	0	1	1	1	1

Psychological abuse & Self-harm

	Representative	Risk of reverse causation	Risk of selection bias	Unadjusted	Unadjusted for age	Family or household perpetrator	Exposure including ages above 15
Kiselydmedres 2022	1	0	1	0	0	0	0
Roustit 2009	1	0	1	0	0	1	1
Mullen 1996	1	0	1	1	1	1	1
Enns 2006	0	0	1	0	0	0	1
Thompson 2019	0	0	1	0	0	1	1
Russell 2019	1	0	0	0	1	0	0
Geng 2023	1	1	0	1	1	0	1

Rajapakse 2020	1	1	0	0	0	1	1

**Psychological abuse & Anxiety disorders**						
	Representative	Risk of selection bias	Unadjusted	Unadjusted for age	Family or household perpetrator	Unadjusted for age, sex, and at least one other confounder
Tenhave 2019	0	1	0	0	0	0
Soenke 2010	1	0	1	1	0	1
Yu 2023	0	1	0	0	1	0
Elbarazi 2023	1	0	1	0	0	1
Raposo 2014	0	0	0	1	1	1
Hovens 2015	1	0	0	0	0	0
Hovens 2015	1	0	0	0	0	0
Kisely 2021	0	1	0	0	0	0
Kisely 2021	0	1	0	0	0	0
**Psychological abuse & Diabetes mellitus type 2**						
	Risk of selection bias	Family or household perpetrator	Effect size for women and men combined	Alternative outcome definition	Unadjusted for sex	Effect size includes men
Monnat 2015	0	0	0	0	0	1
Njoroge 2023	1	0	0	0	1	1
Gaston 2023	0	0	1	1	0	0
Kascakova 2022	0	0	0	0	1	1
Kascakova 2022	0	0	0	0	1	1
Zhu 2023	0	0	1	0	0	1

Zhu 2023	0	0	1	0	0	0
Thomas 2008	1	1	0	1	0	1
Thomas 2008	1	1	0	1	0	1
Duncan 2015	0	1	1	0	0	0
Duncan 2015	0	1	1	0	0	0
Duncan 2015	0	1	1	0	0	1
Duncan 2015	0	1	1	0	0	1

Psychological abuse & Schizophrenia

	Risk of reverse causation	Risk of selection bias	Unadjusted	Family or household perpetrator	Exposure including ages above 15	Exposure limited to below 15
Murphy 2020	0	1	1	0	0	1
Alkema 2023	1	0	0	0	1	0
Abajobir 2017	0	1	0	0	0	1
Chatziioannidis 2019	1	0	1	1	1	0
Trotta 2023	1	0	0	1	1	0
Trotta 2023	1	0	0	1	0	1

Psychological abuse & Alcohol use disorder

	Risk of selection bias	Family or household perpetrator	Effect size for women and men combined	Effect size includes men	Unadjusted for age, sex, and at least one other confounder
Fenton 2013	1	1	0	1	0
Roustit 2009	1	1	0	1	0

Kascakova 2022	0	0	0	1	1
Kascakova 2022	0	0	0	1	1
Najman 2022	1	0	0	1	0
Najman 2022	1	0	0	1	0
Broekhof 2023	1	1	1	0	1
Broekhof 2023	1	1	1	1	1
Kisely 2021	1	0	0	1	0
Laflair 2013	0	1	1	0	0
**Psychological abuse & Drug use disorders**					
	Representative	Risk of selection bias	Family or household perpetrator	Effect size for women and men combined	Alternative outcome definition
Schwartz 2024	0	0	1	0	1
Broekhof 2023	0	1	1	1	0
Kisely 2021	1	0	0	0	1
Harrington 2011	0	1	1	0	1
Najman 2022	0	1	0	0	0
Najman 2022	0	1	0	0	0
Conroy 2009	1	0	0	1	0
Conroy 2009	1	0	0	1	0
Scheidell 2018	0	0	1	0	1

Scheidell 2018	0	0	1	0	1

Psychological abuse & Asthma		
	Representative	Unadjusted for age
Njoroge 2023	1	1
Abajobir 2017	1	0
Kascakova 2022	0	1
Kascakova 2022	0	1
Han 2022	0	0

Table S33. Bias Covariates for childhood neglect and corresponding outcomes.

Study	Covariates													
Neglect & Major depressive disorder														
	Representativeness	Risk of revere causation	Risk of selection bias	Uncontrolled effect size	Uncontrolled for age	Family/household perpetrators	Exposure including ages above 15	Exposure limited to below 15	Effect size for women and men combined	Uncontrolled for sex	Effect size includes men	Uncontrolled for age, sex, and at least one other confounder	Aggregate outcome definition	Administrative exposure ascertainment
Zhang 2023	0	0	1	0	0	0	1	0	0	0	1	0	1	0
Widom 2023	1	1	1	0	0	1	0	1	0	0	1	1	1	1
Widom 2007	1	0	1	1	1	1	0	1	0	1	1	1	0	1
Brown 1999	1	0	0	1	1	1	1	0	0	1	1	1	1	0

Comijs 2013	1	1	0	0	0	1	1	0	0	0	1	0	1	0
Young 2011	1	0	1	0	0	1	0	1	0	0	1	0	1	0
Kisely 2020	1	0	1	0	0	0	1	0	0	0	1	0	1	1
Lemasters 2021	1	0	1	0	0	0	1	0	1	0	0	0	0	0
Ebert 2019	1	0	1	0	1	0	1	0	0	1	1	1	0	0
Xiao 2022	0	1	0	1	1	0	0	0	0	1	1	1	1	0
Xiao 2022	0	1	0	1	1	0	0	0	0	1	1	1	1	0
Danese 2023	1	0	1	1	1	1	0	1	0	1	1	1	1	0
Danese 2023	1	0	1	1	1	1	0	1	0	1	1	1	1	0
Danese 2023	1	0	1	1	1	1	0	1	0	1	1	1	1	0
Kang 2023	1	1	0	1	1	0	1	0	0	1	1	1	0	0
Kang 2023	1	1	0	1	1	0	1	0	0	1	1	1	0	0
Zhou 2023	1	1	0	0	1	0	1	0	1	0	1	1	0	0
Zhou 2023	1	1	0	0	1	0	1	0	1	0	1	1	0	0
Zhou 2023	1	1	0	0	1	0	1	0	1	0	0	1	0	0
Zhou 2023	1	1	0	0	1	0	1	0	1	0	0	1	0	0
Elbarazi 2023	1	0	0	1	0	0	1	0	0	1	1	1	1	0
Elbarazi 2023	1	0	0	1	0	0	1	0	0	1	1	1	1	0

Hovens 2015	1	0	0	0	0	1	1	0	0	0	1	0	1	0
Hovens 2015	1	0	0	0	0	1	1	0	0	0	1	0	1	0
Galloeag 2017	0	0	1	0	0	0	0	0	1	0	0	0	0	0
Galloeag 2017	0	0	1	0	0	0	0	0	1	0	1	0	0	0
Houtepen 2020	0	0	0	0	0	1	0	0	0	1	1	1	1	0

**Neglect & Anxiety disorders**										
	Representative	Risk of selection bias	Uncontrolled	Unadjusted for age	Family or household perpetrator	Exposure including ages above 15	Exposure limited to below 15	Unadjusted for age, sex, and at least one other confounder	Administrative exposure ascertainment	PSTD outcome definition
Tenhave 2019	0	1	0	0	0	1	0	0	0	0
Widom 1999	1	1	1	1	1	0	1	1	1	1
Fujiwara 2011	1	1	0	0	0	1	0	0	0	0
Young 2011	1	1	0	0	1	0	1	0	0	0
Raposo 2014	0	0	0	1	1	1	0	1	0	0
Danese 2023	1	1	1	1	1	0	1	1	0	0
Danese 2023	1	1	1	1	1	0	1	1	0	0
Danese 2023	1	1	1	1	1	0	1	1	0	0
Elbarazi 2023	1	0	1	0	0	1	0	1	0	0
Elbarazi 2023	1	0	1	0	0	1	0	1	0	0

Hovens 2015	1	0	0	0	1	1	0	0	0	0
Hovens 2015	1	0	0	0	1	1	0	0	0	0
Kisely 2020	1	1	0	0	0	1	0	0	1	0
Kisely 2020	1	1	0	0	0	1	0	0	1	1

Neglect & Diabetes mellitus type 2

	Risk of selection bias	Family or household perpetrator	Exposure including ages above 15	Effect size for women and men combined	Alternate outcome definition	Effect size includes men	Unadjusted for age, sex, and at least one other confounder
Widom 2023	1	1	0	0	0	1	1
Kascakova 2022	0	0	1	0	0	1	1
Kascakova 2022	0	0	1	0	0	1	1
Kascakova 2022	0	0	1	0	0	1	1
Kascakova 2022	0	0	1	0	0	1	1
Sanderson 2023	1	0	1	1	1	0	0
Sanderson 2023	1	0	1	1	1	1	0
Zhu 2023	0	0	1	1	0	1	0
Zhu 2023	0	0	1	1	0	0	0
Thomas 2008	1	0	1	0	1	1	0
Duncan 2015	0	1	0	1	0	0	0
Duncan 2015	0	1	0	1	0	0	0
Duncan 2015	0	1	0	1	0	1	0

Duncan 2015	0	1	0	1	0	1	0

**Neglect & Alcohol use disorder**						
	Risk of selection bias	Family or household perpetrator	Effect size for women and men combined	Alternate outcome definition	Effect size includes men	Unadjusted for age, sex, and at least one other confounder
Broekhof 2023	1	1	1	0	0	1
Laflair 2013	0	1	1	1	0	0
Fenton 2013	1	1	0	0	1	0
Fenton 2013	1	1	0	0	1	0
Kascakova 2022	0	0	0	0	1	1
Kascakova 2022	0	0	0	0	1	1
Kascakova 2022	0	0	0	0	1	1
Kascakova 2022	0	0	0	0	1	1
Kisely 2020	1	0	0	1	1	0

**Neglect & Drug use disorders**					
	Representative	Risk of selection bias	Exposure including ages above 15	Exposure limited to below 15	Effect size includes men
Harrington 2011	0	1	1	0	1
Schwartz 2024	0	0	1	0	1
Schwartz 2024	0	0	1	0	1
Huang 2011	0	1	0	1	1
Conroy 2009	1	0	1	0	0

Conroy 2009	1	0	1	0	1
Abajobir 2017	1	1	0	1	1
Abajobir 2017	1	1	0	1	0
**Neglect & Self-harm**					
	Representative	Risk of reverse causation	Risk of selection bias	Family or household perpetrator	Exposure including ages above 15
Kiselydmedres 2022	1	0	1	0	0
Geng 2023	1	1	0	0	1
Brown 1999	1	0	0	1	1
Enns 2006	0	0	1	0	1
Thompson 2019	0	0	1	1	0
Bruffaerts 2010	0	0	1	0	1
Bruffaerts 2010	0	0	1	0	1
Rajapakse 2020	1	1	0	0	1

**Neglect & STIs, excluding HIV**		
	Effect size for women and men combined	Effect size includes men
London 2017	0	1
Wilson 2009	0	1
Wilson 2009	0	1
Wilson 2009	1	0

Wilson 2009	0	1
Haydon 2011	1	1
Haydon 2011	1	1
Haydon 2011	1	0
Haydon 2011	1	0
**Neglect & Schizophrenia**		
	Family or household perpetrator	
Mall 2020	1	
Abajobir 2017	0	
Chatziioannidis 2019	1	
Alkema 2023	0	
Alkema 2023	0	

Section 9: Primary Analysis Funnel Plots

Section 9.1: Primary analysis funnel plots for childhood physical violence and outcomes

Figure S22. Primary analysis funnel plot for childhood physical violence and maternal abortion and miscarriage

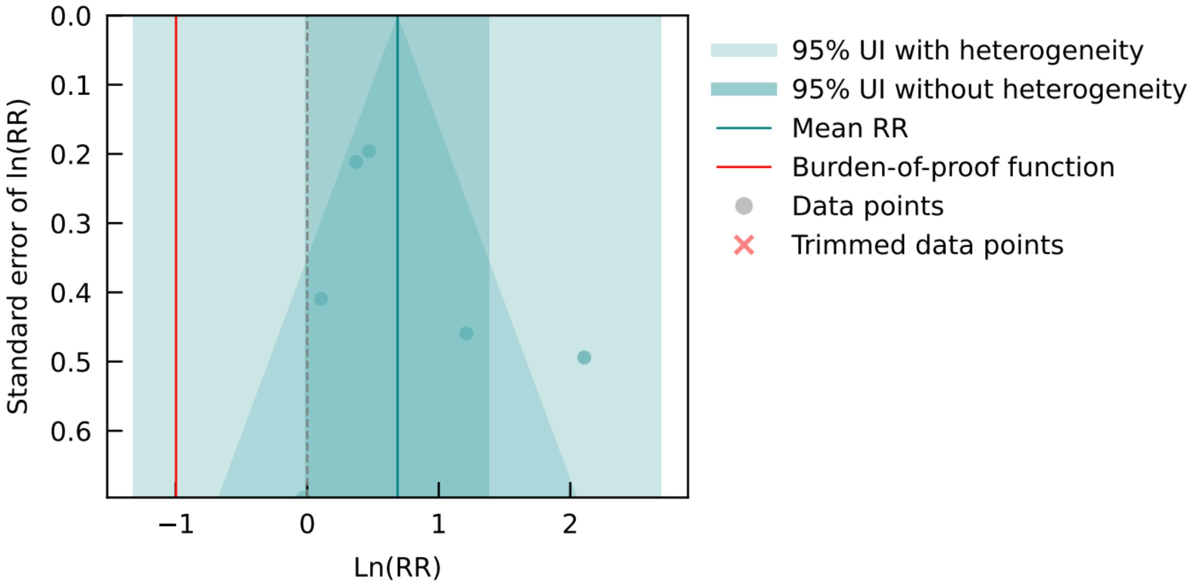

Figure S23. Primary analysis funnel plot for childhood physical violence and alcohol use disorders

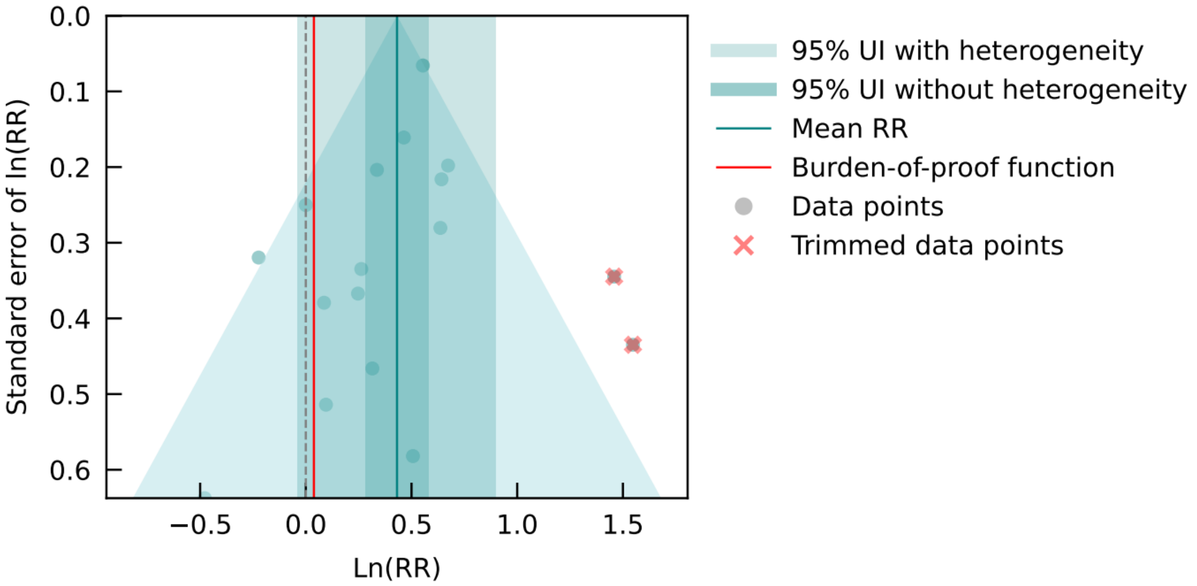

Figure S24. Primary analysis funnel plot for childhood physical violence and anxiety disorders

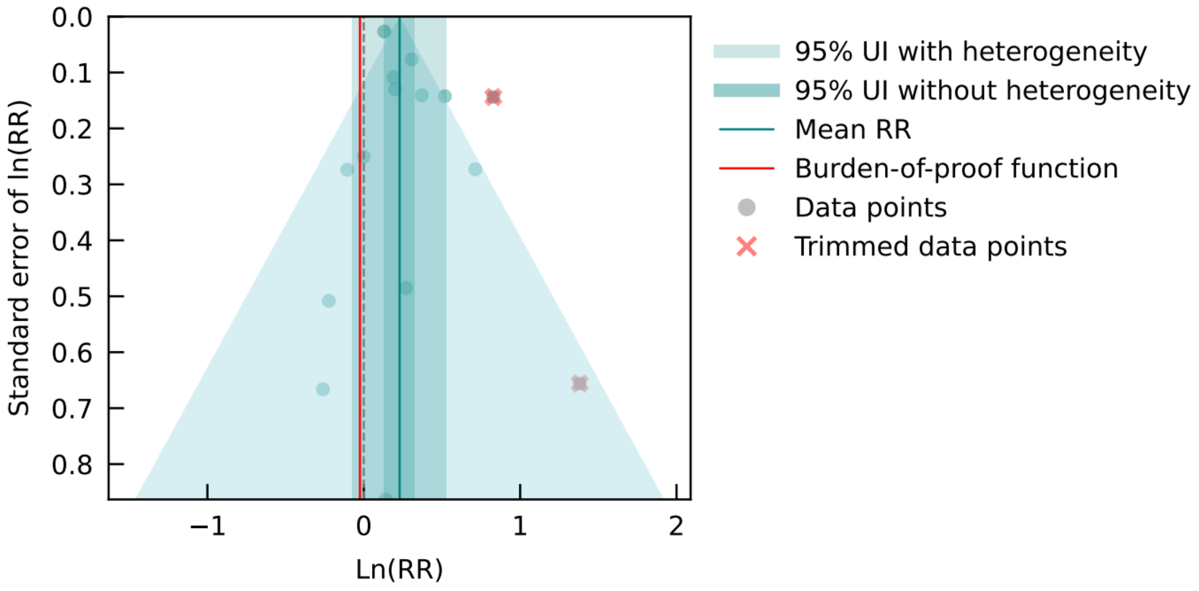

Figure S25. Primary analysis funnel plot for childhood physical violence and asthma

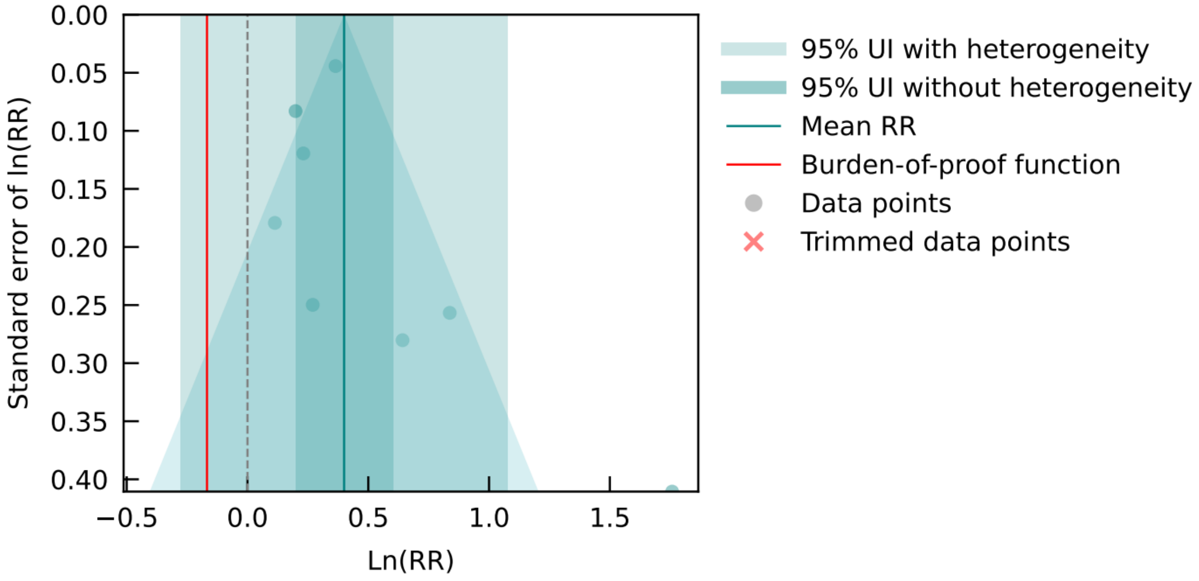

Figure S26. Primary analysis funnel plot for childhood physical violence and major depression disorder

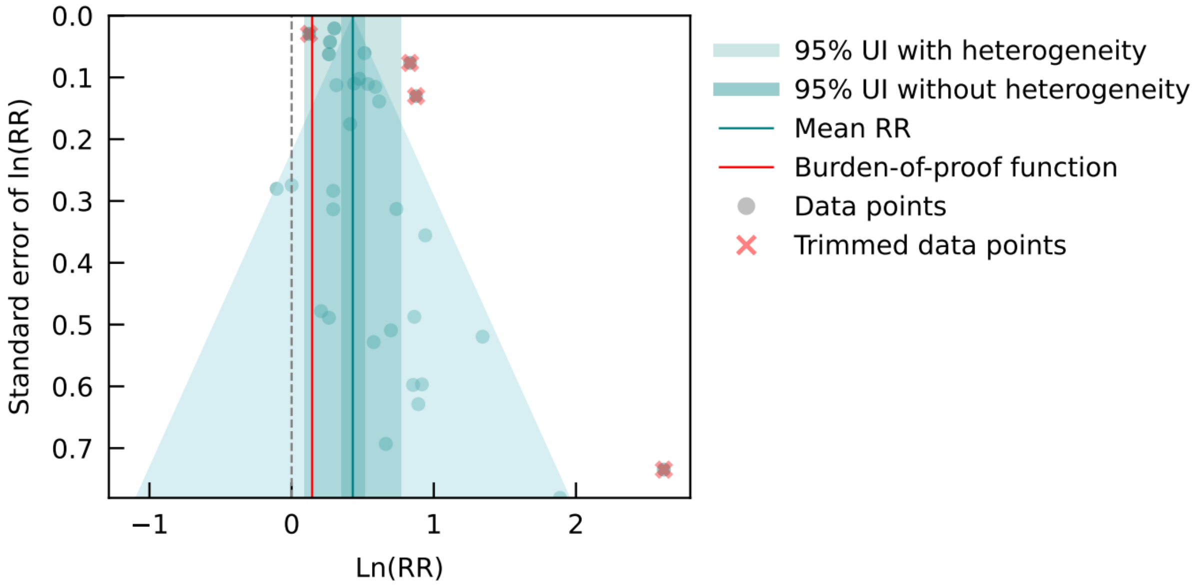

Figure S27. Primary analysis funnel plot for childhood physical violence and diabetes

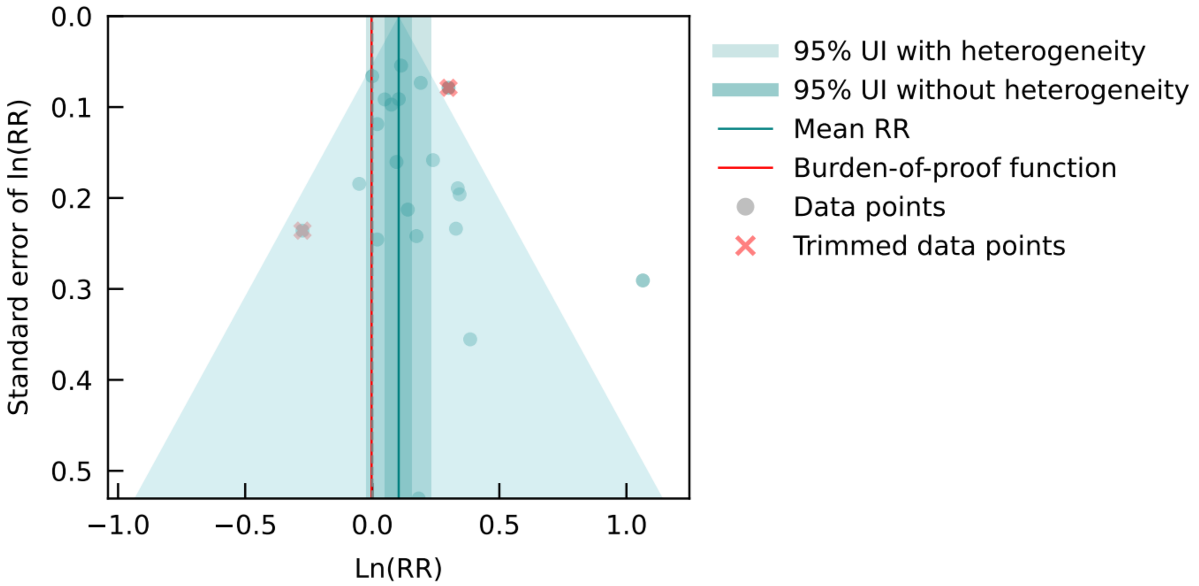

Figure S28. Primary analysis funnel plot for childhood physical violence and drug use disorders

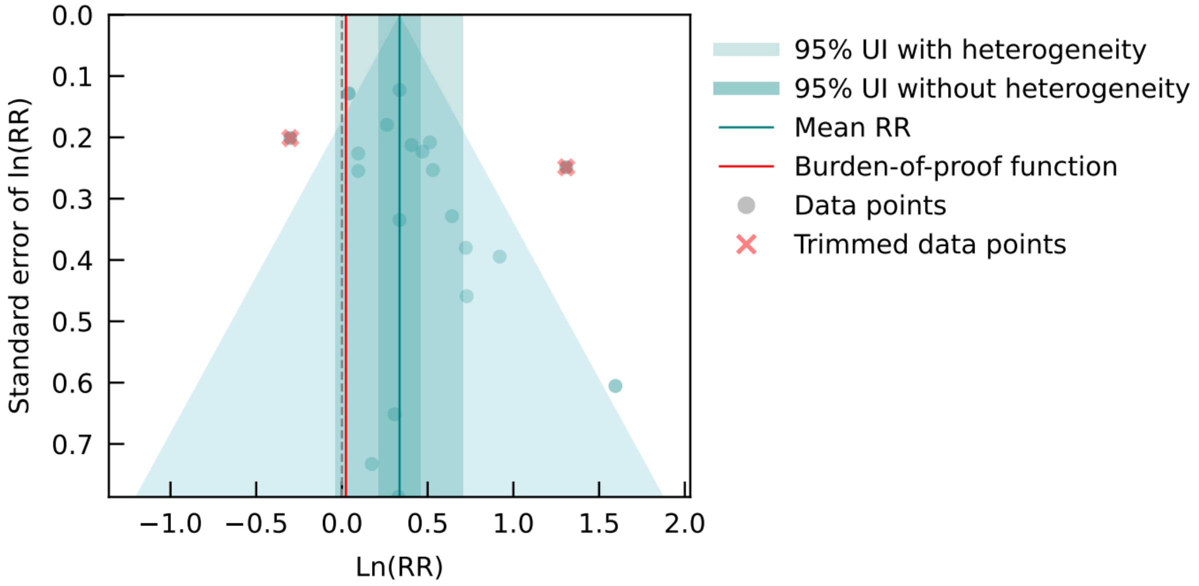

Figure S29. Primary analysis funnel plot for childhood physical violence and eating disorders

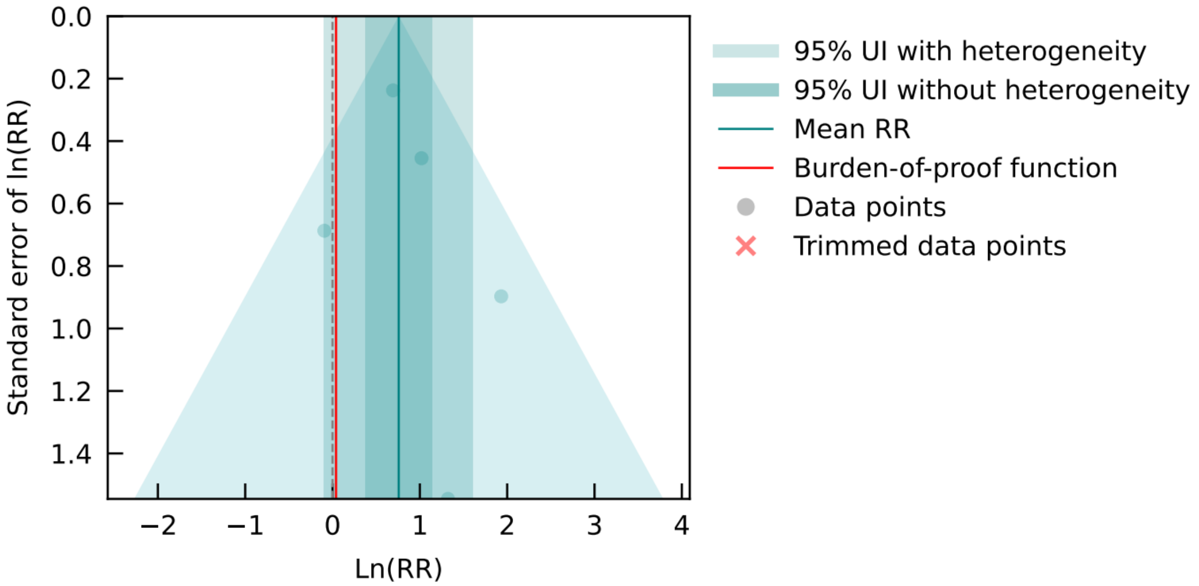

Figure S30. Primary analysis funnel plot for childhood physical violence and gynecological diseases

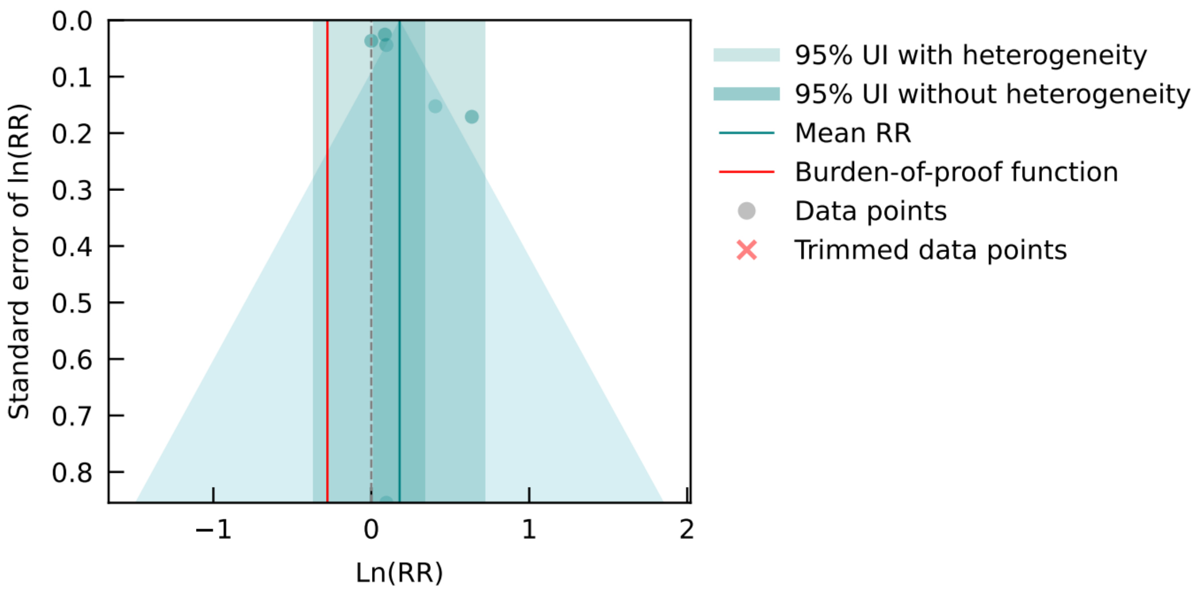

Figure S31. Primary analysis funnel plot for childhood physical violence and ischemic heart disease

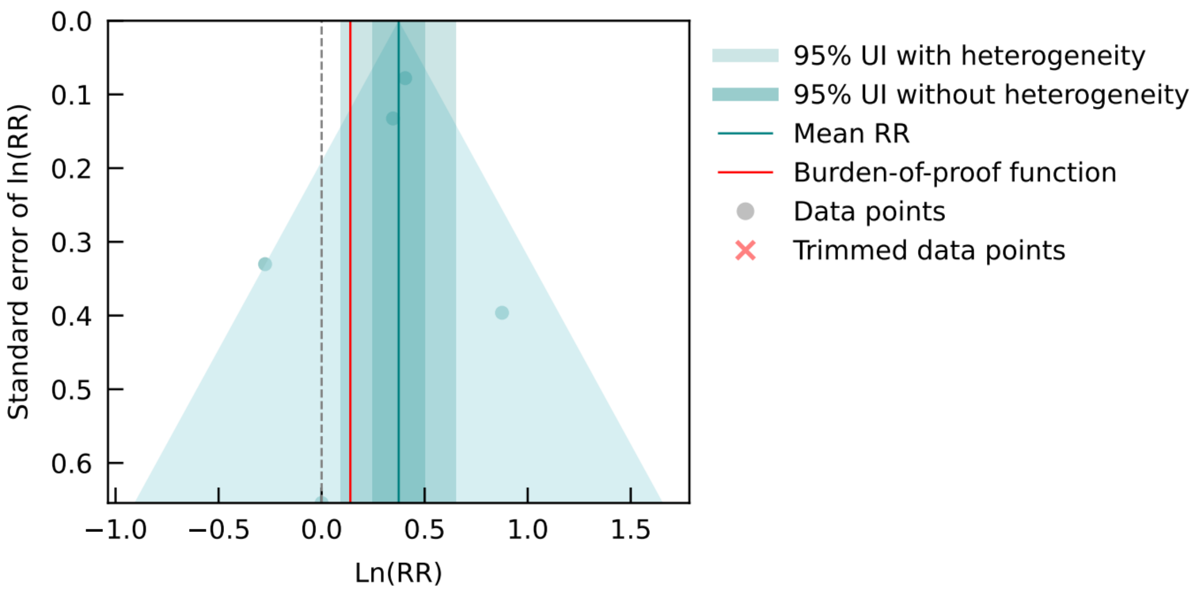

Figure S32. Primary analysis funnel plot for childhood physical violence and migraines

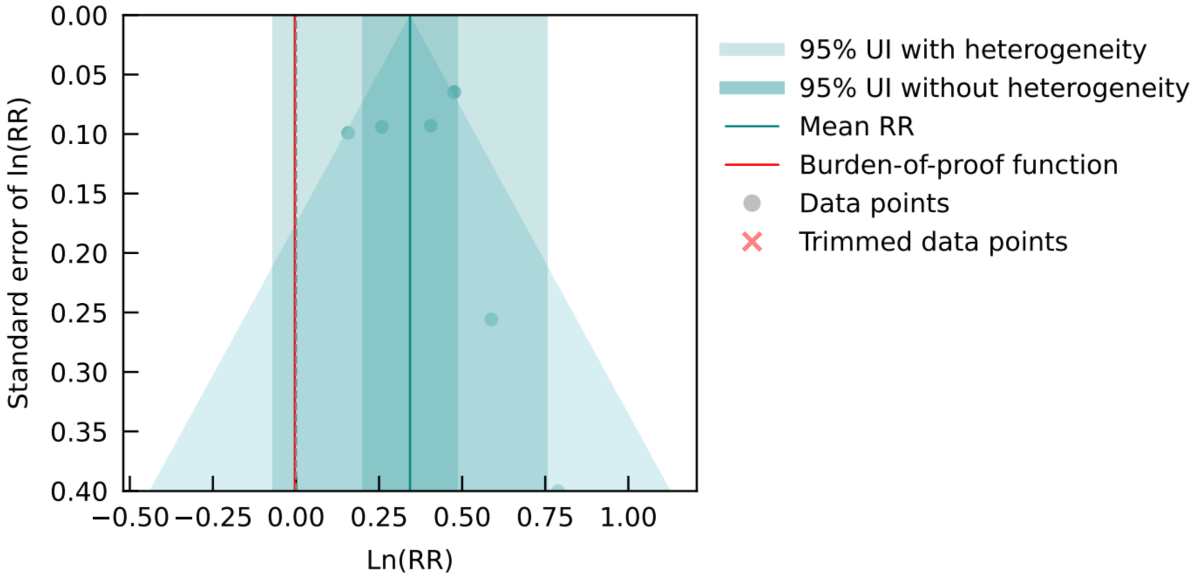

Figure S33. Primary analysis funnel plot for childhood physical violence and schizophrenia

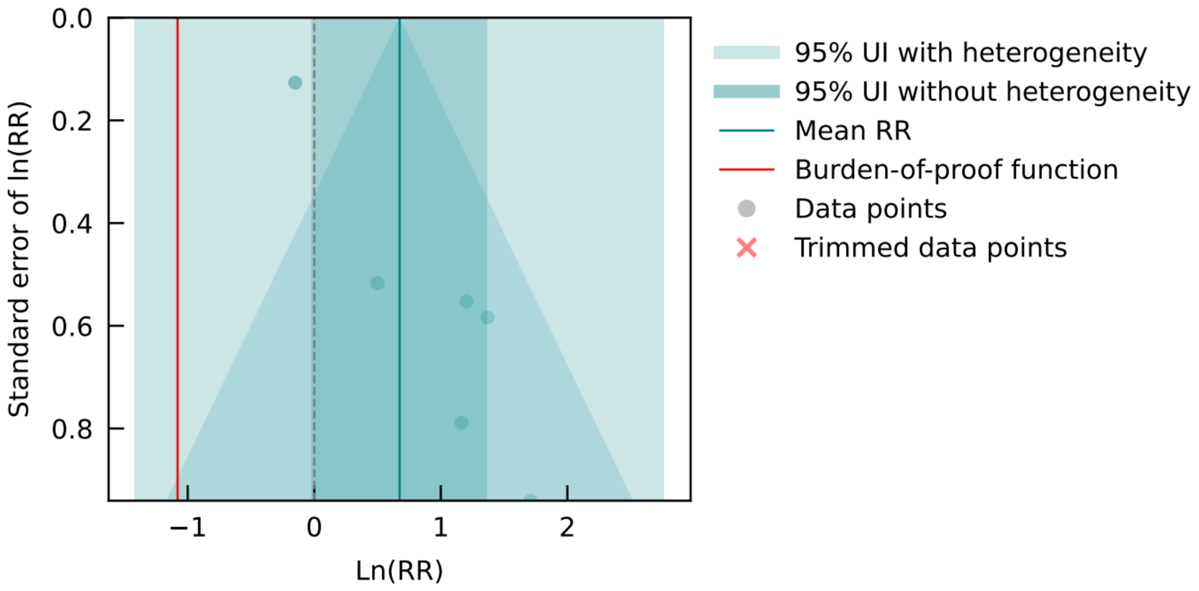

Figure S34. Primary analysis funnel plot for childhood physical violence and self-harm

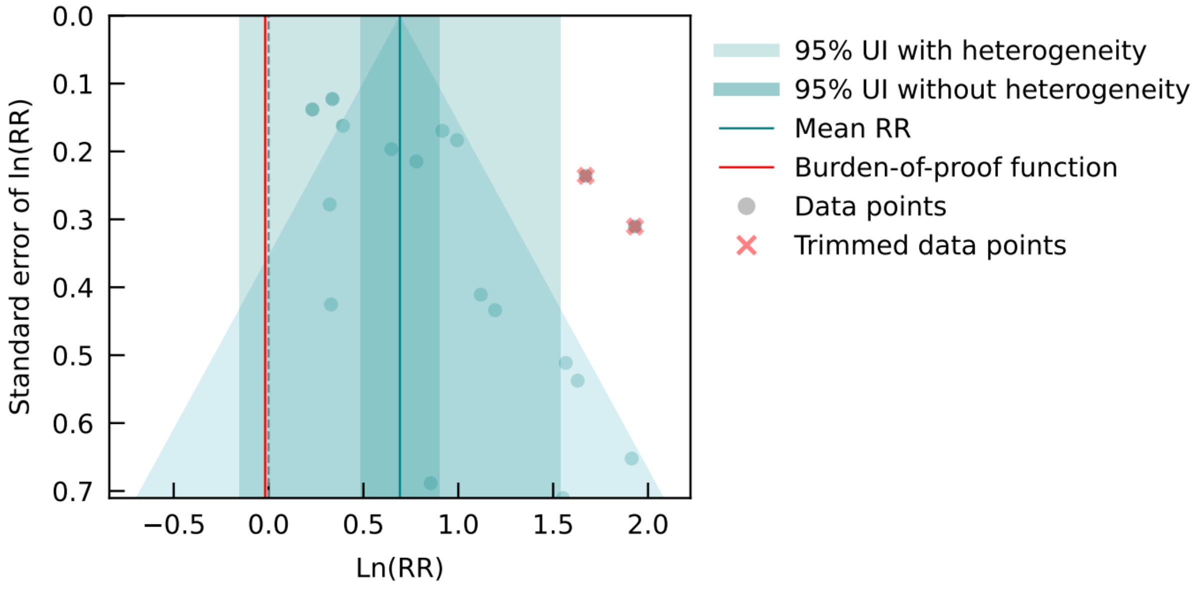

Figure S35. Primary analysis funnel plot for childhood physical violence and sexually transmitted infections (excluding HIV)

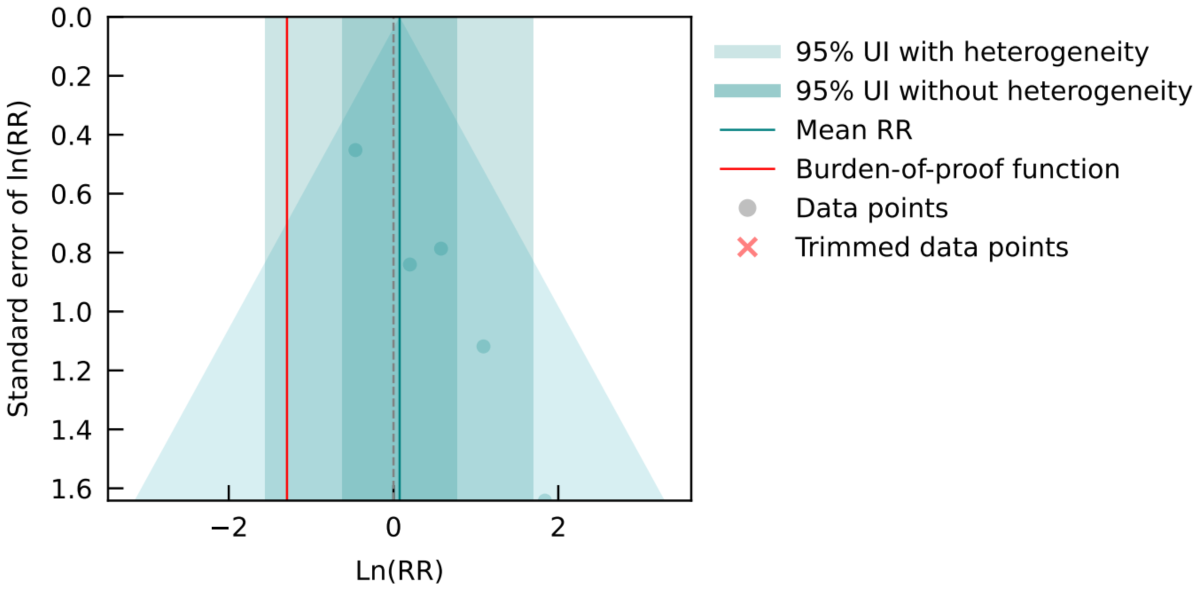

Figure S36. Primary analysis funnel plot for childhood physical violence and stroke

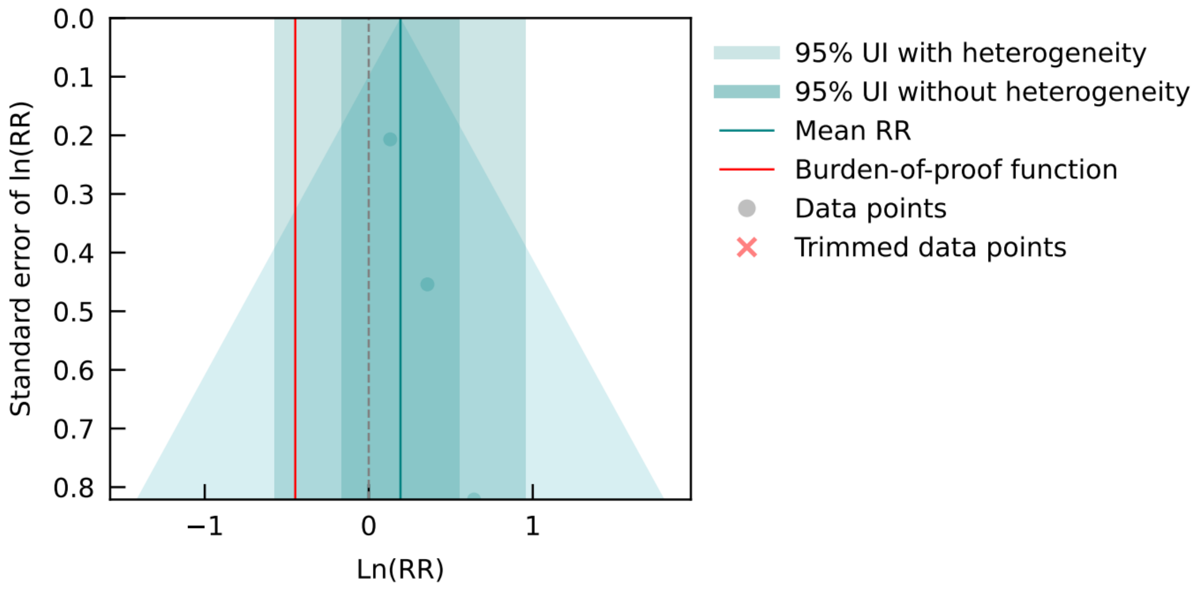

Section 9.2: Primary analysis funnel plots for childhood psychological abuse and outcomes

Figure S37. Primary analysis funnel plot for childhood psychological violence and alcohol use disorder

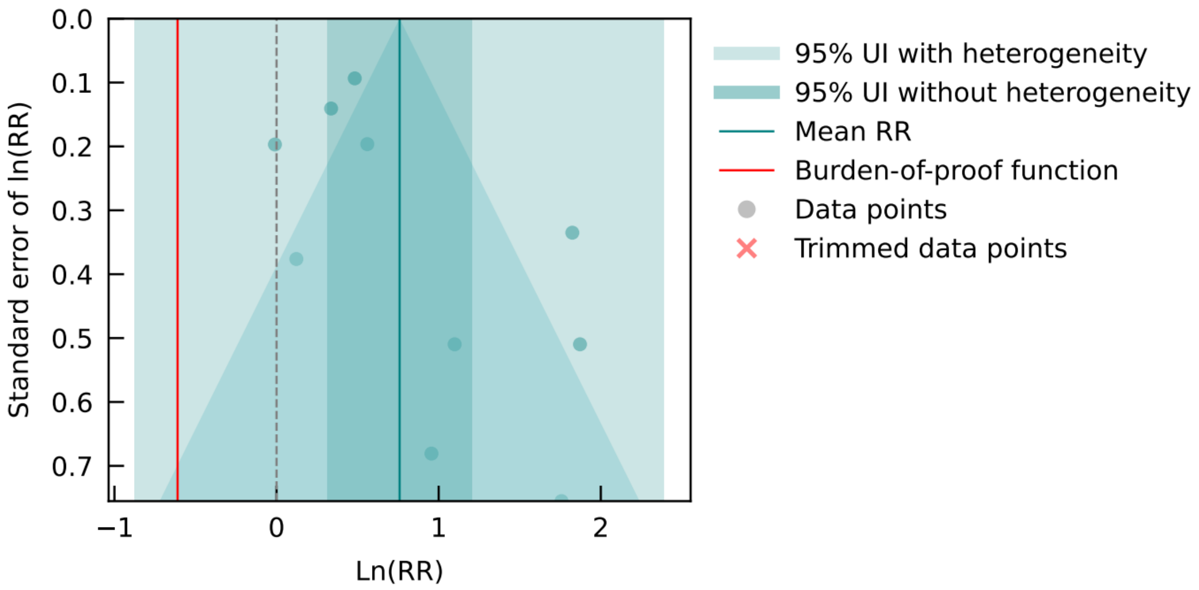

Figure S38. Primary analysis funnel plot for childhood psychological violence and anxiety disorder

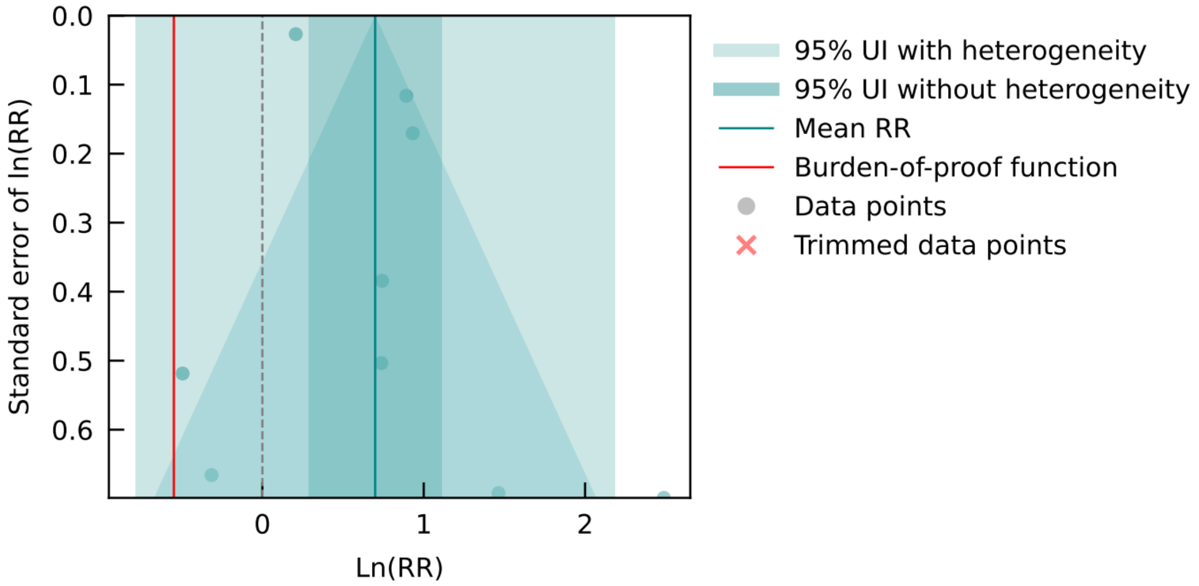

Figure S39. Primary analysis funnel plot for childhood psychological violence and asthma

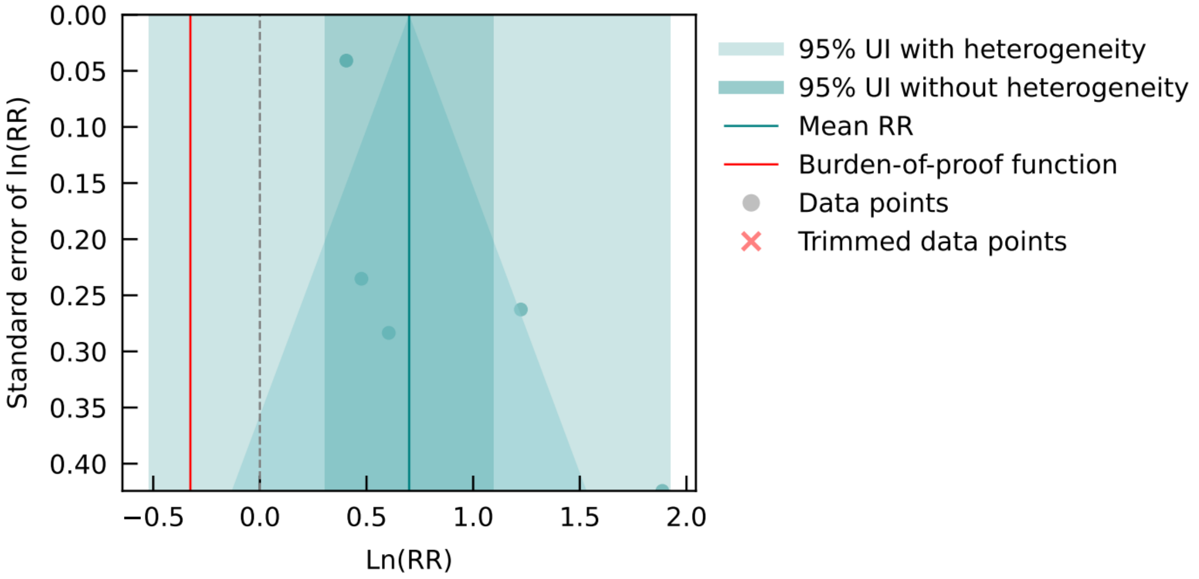

Figure S40. Primary analysis funnel plot for childhood psychological violence and major depression disorder

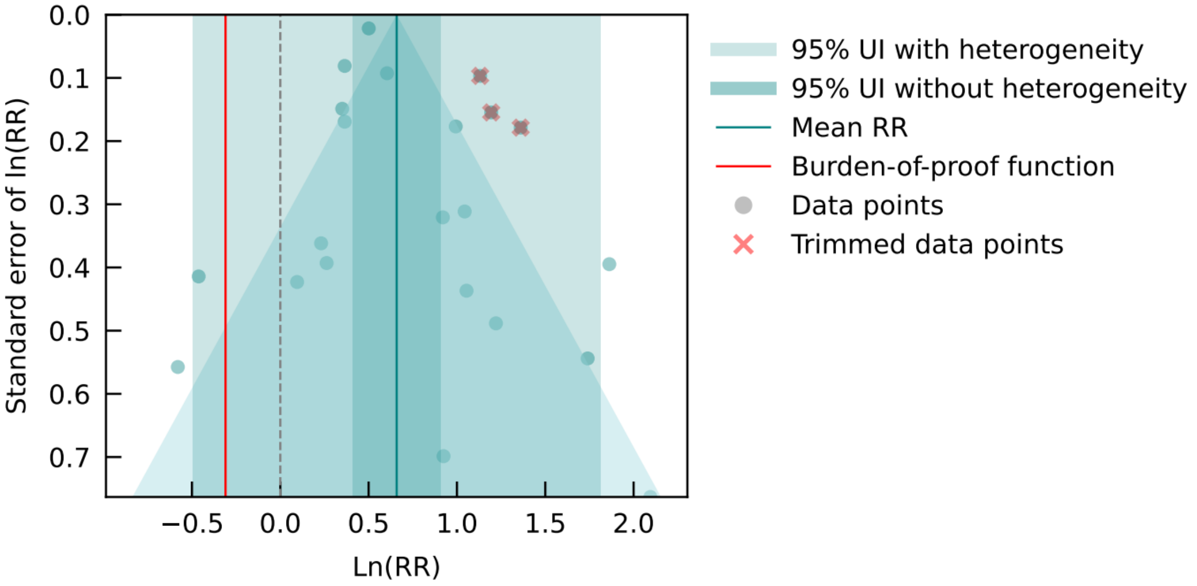

Figure S41. Primary analysis funnel plot for childhood psychological violence and diabetes

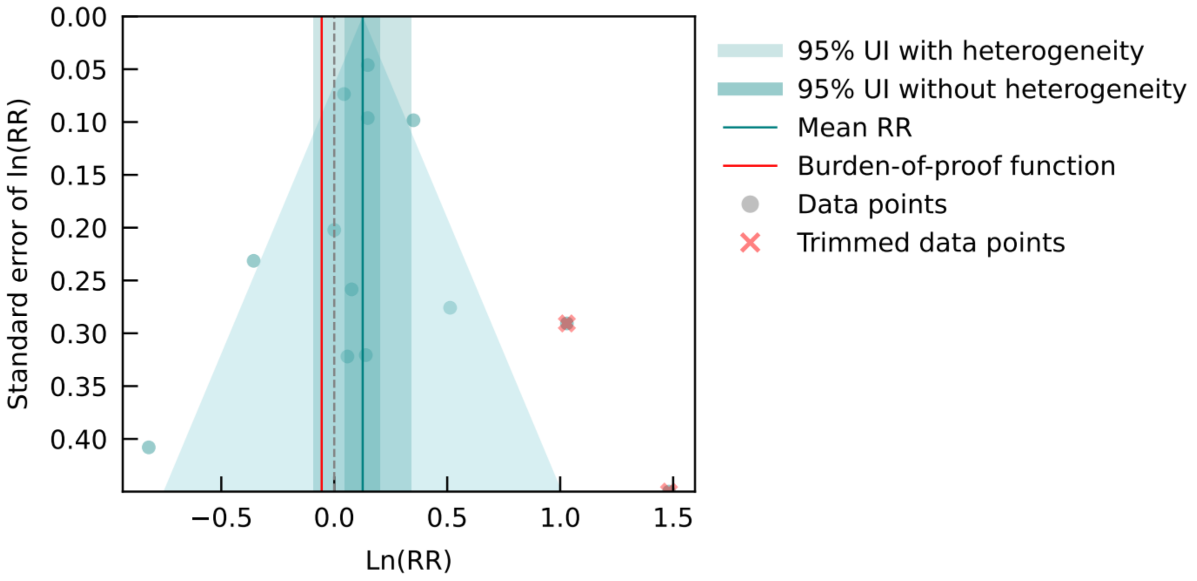

Figure S42. Primary analysis funnel plot for childhood psychological violence and drug use disorders

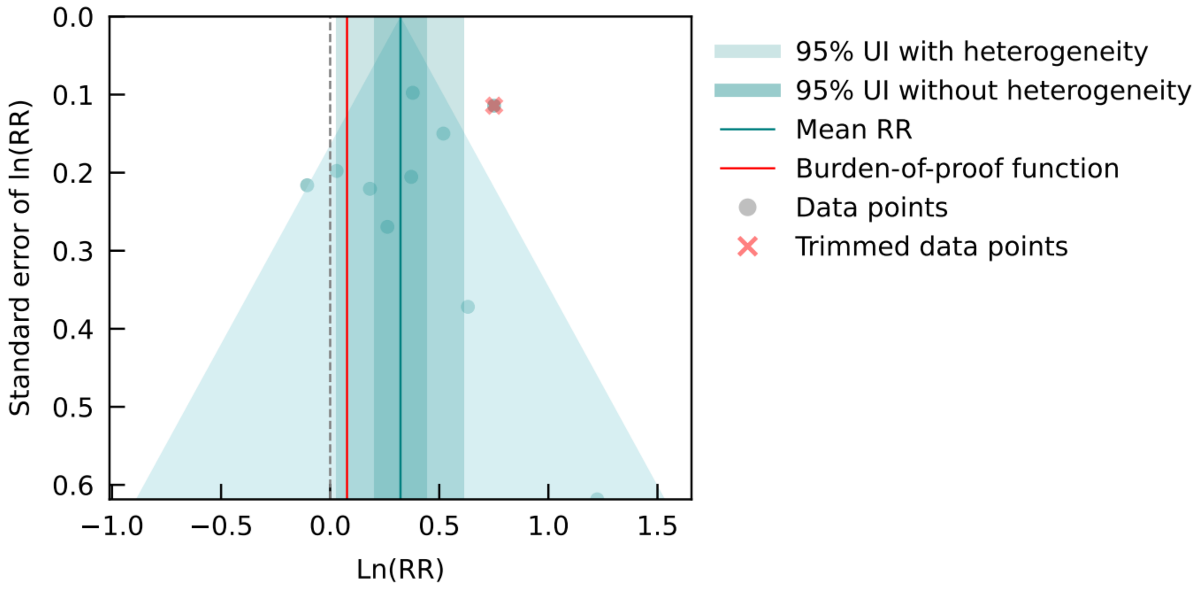

Figure S43. Primary analysis funnel plot for childhood psychological violence and gynecological diseases

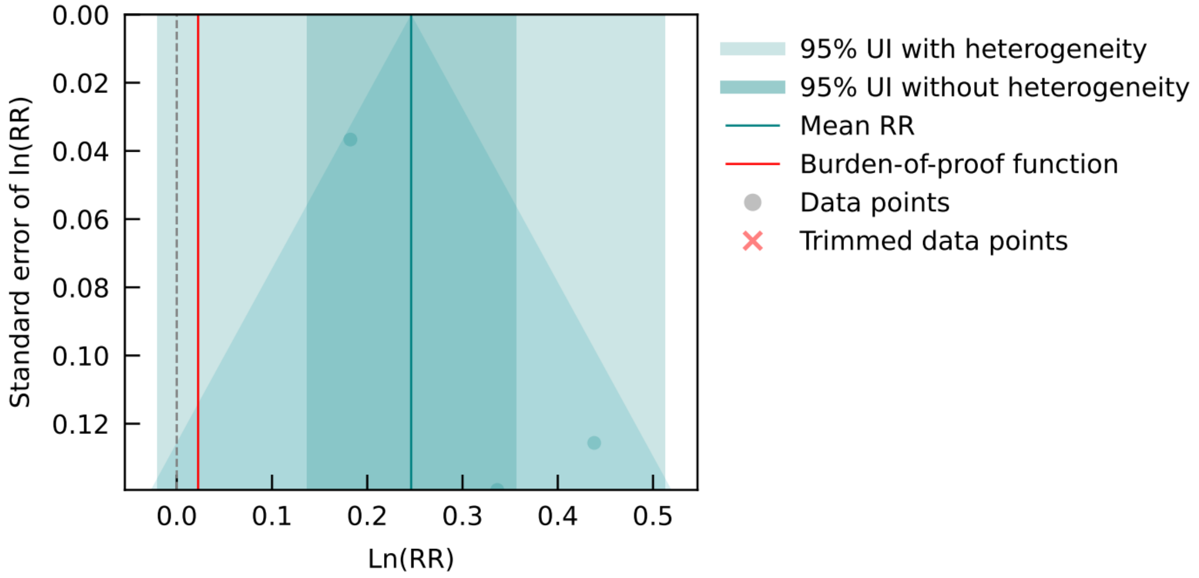

Figure S44. Primary analysis funnel plot for childhood psychological violence and ischemic heart disease

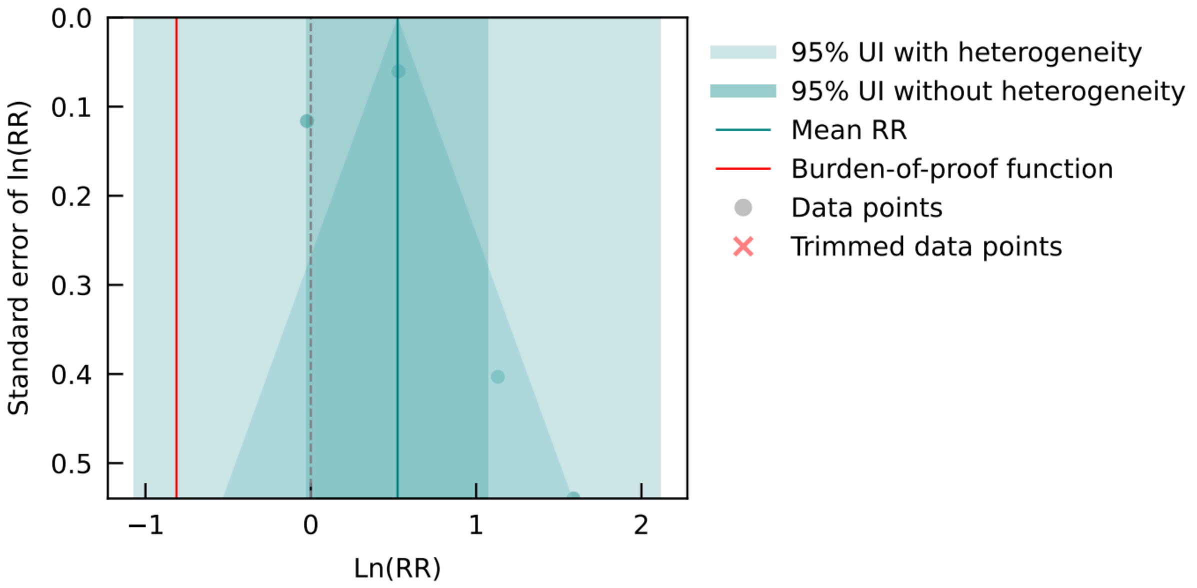

Figure S45. Primary analysis funnel plot for childhood psychological violence and migraines

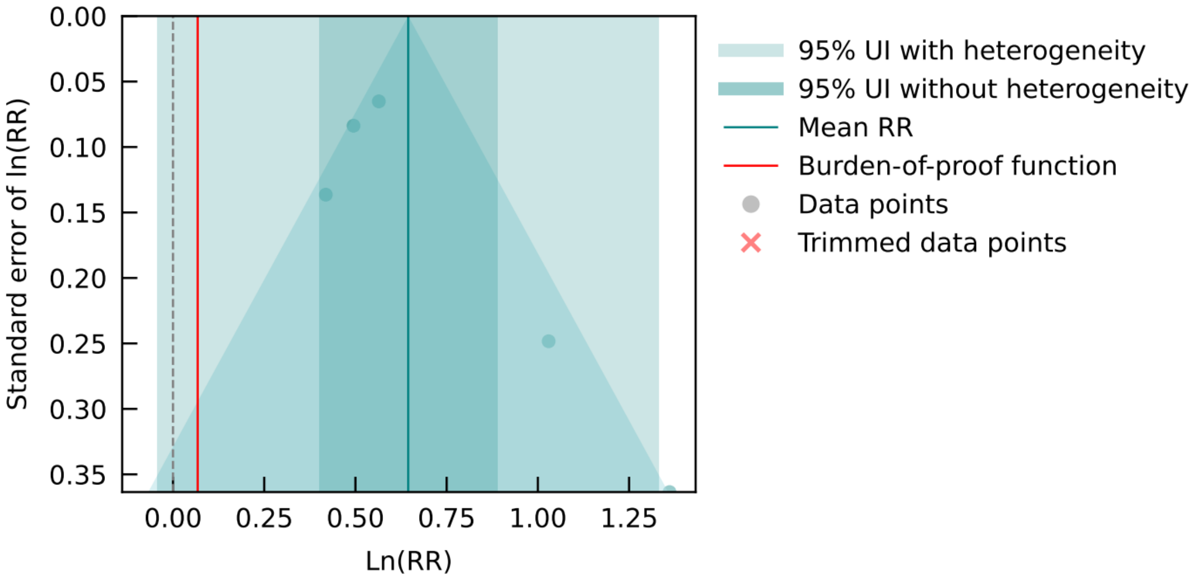

Figure S46. Primary analysis funnel plot for childhood psychological violence and schizophrenia

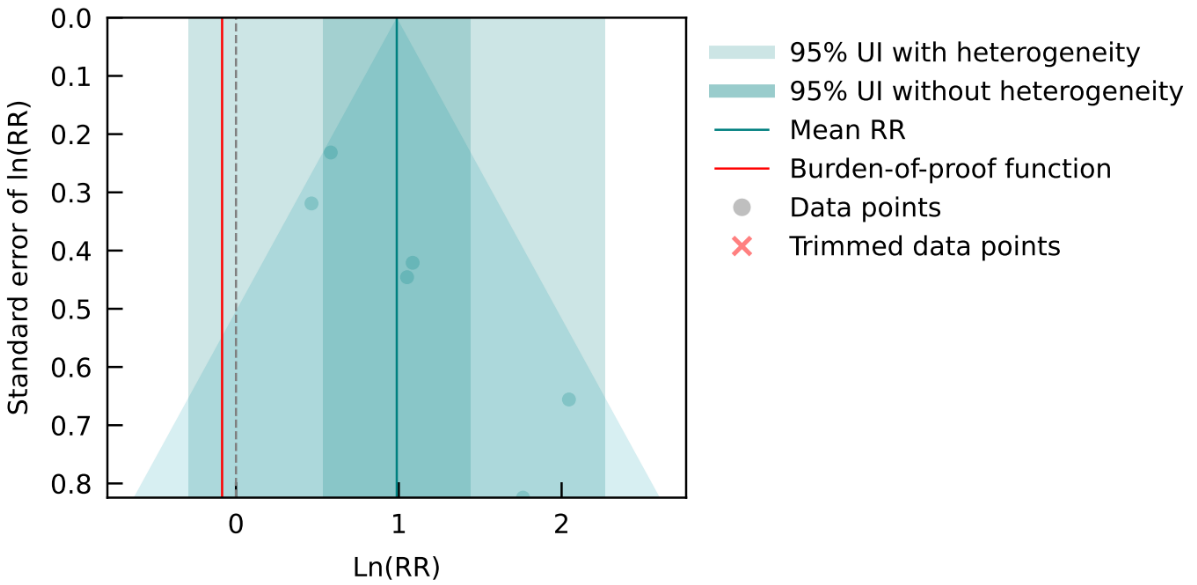

Figure S47. Primary analysis funnel plot for childhood psychological violence and self-harm

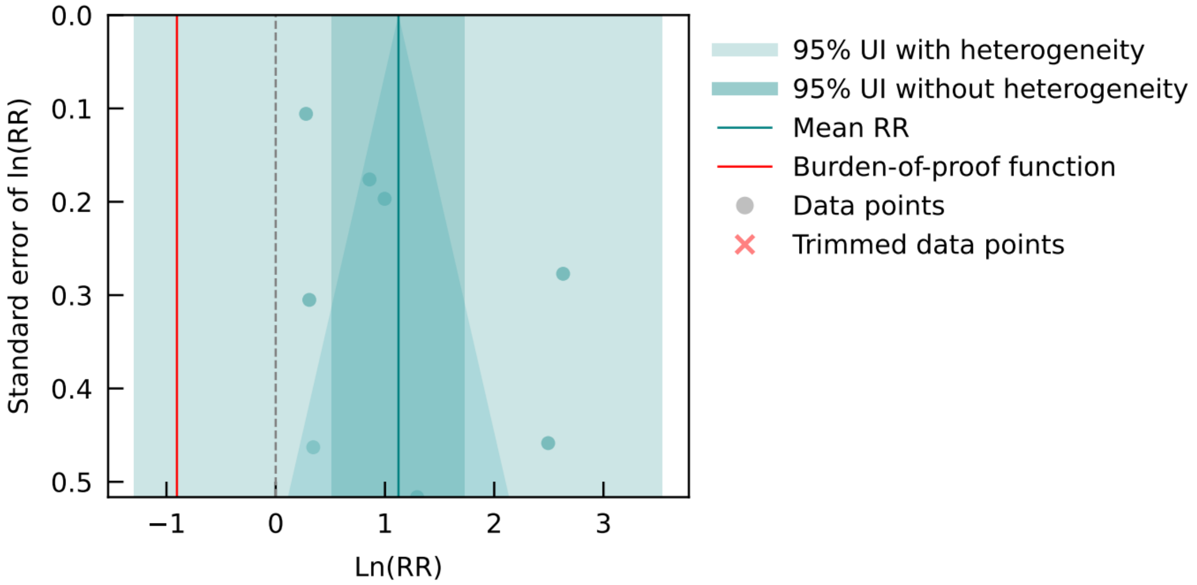

Section 9.3: Primary analysis funnel plots for childhood neglect and outcomes

Figure S48. Primary analysis funnel plot for childhood neglect and alcohol use disorders

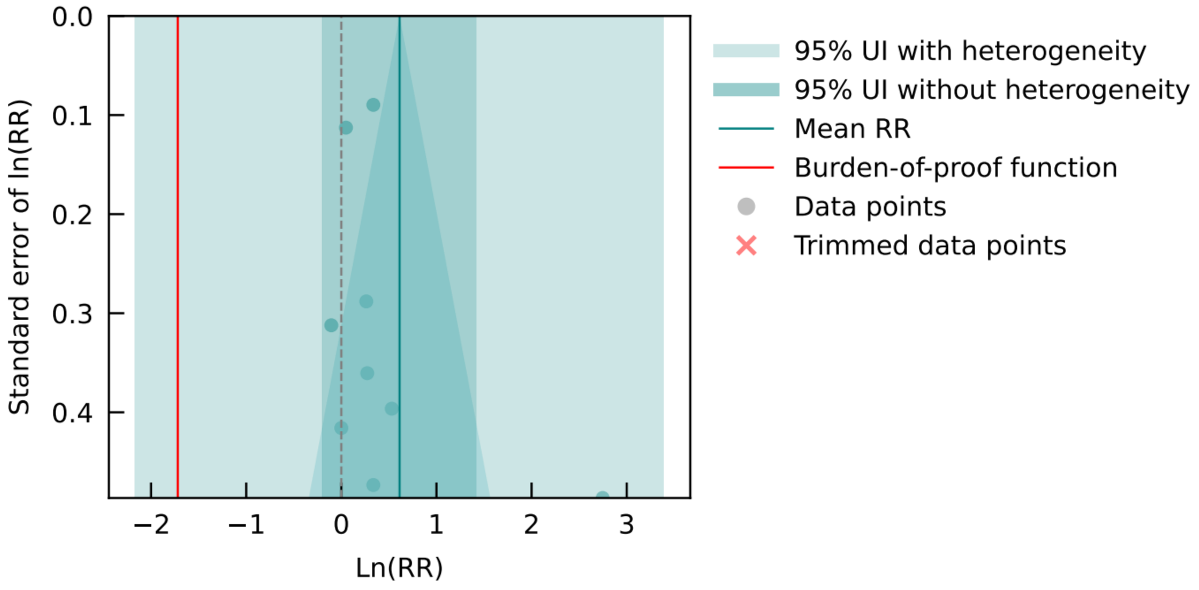

Figure S49. Primary analysis funnel plot for childhood neglect and anxiety disorders

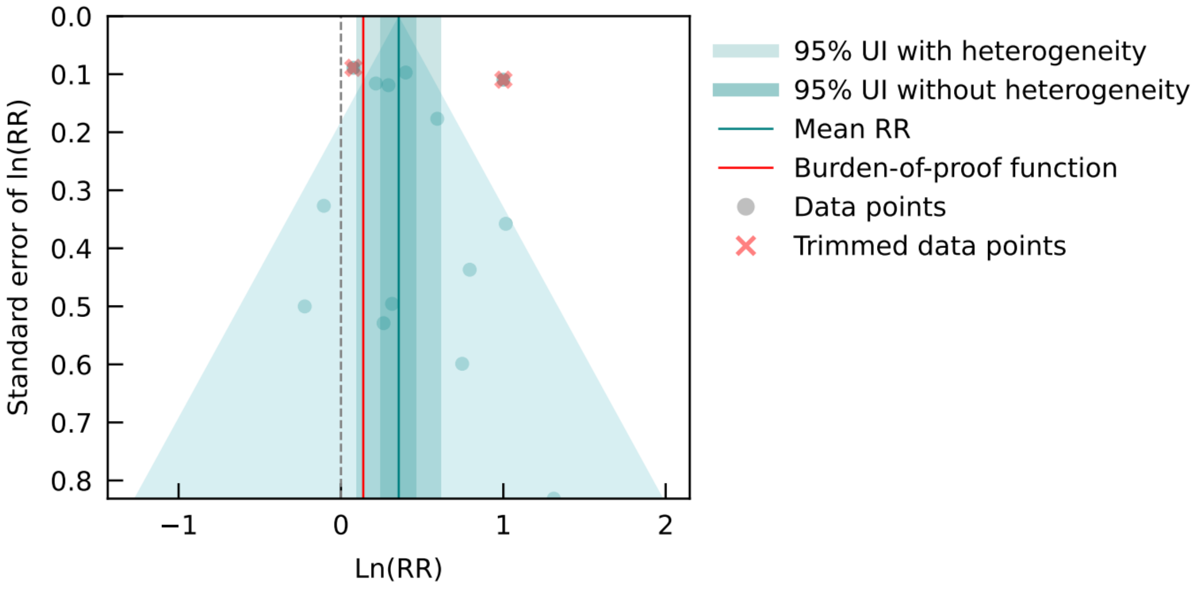

Figure S50. Primary analysis funnel plot for childhood neglect and asthma

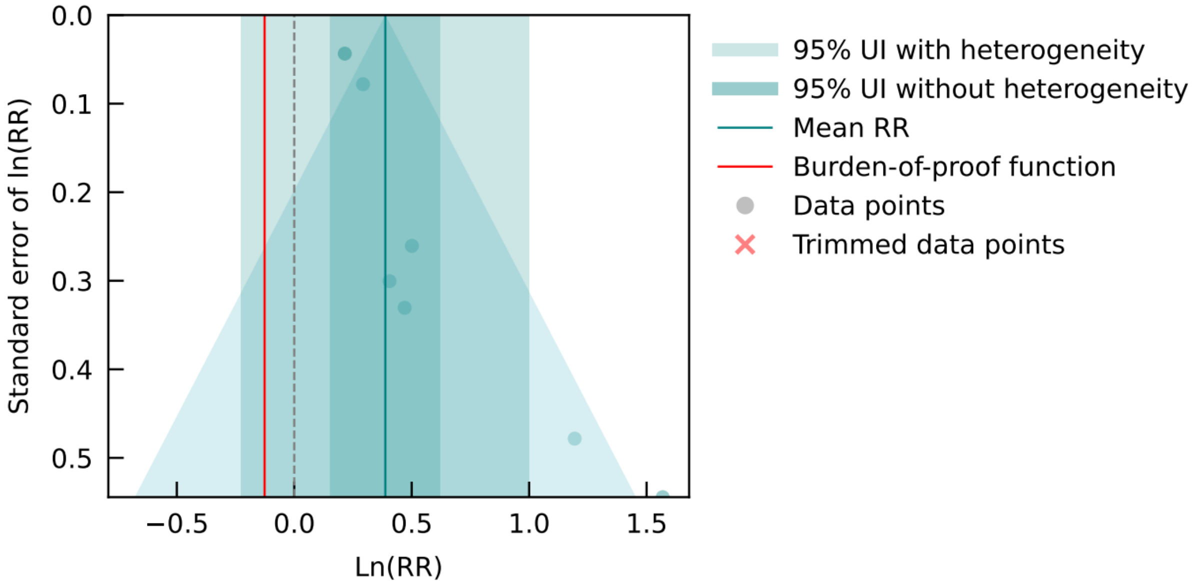

Figure S51. Primary analysis funnel plot for childhood neglect and major depression disorder

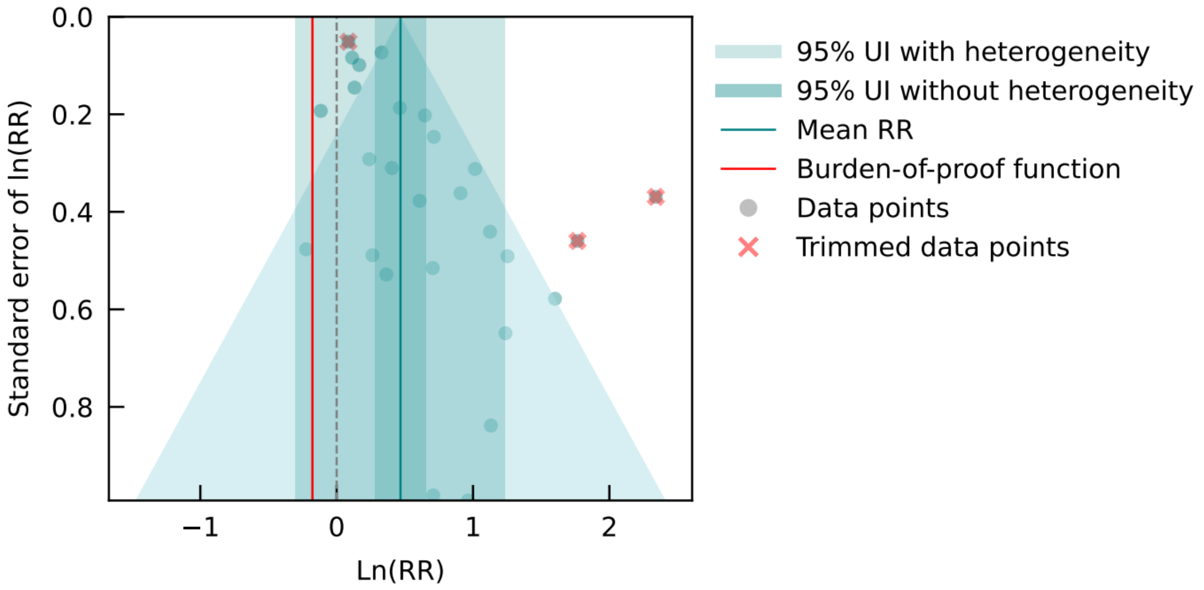

Figure S52. Primary analysis funnel plot for childhood neglect and diabetes

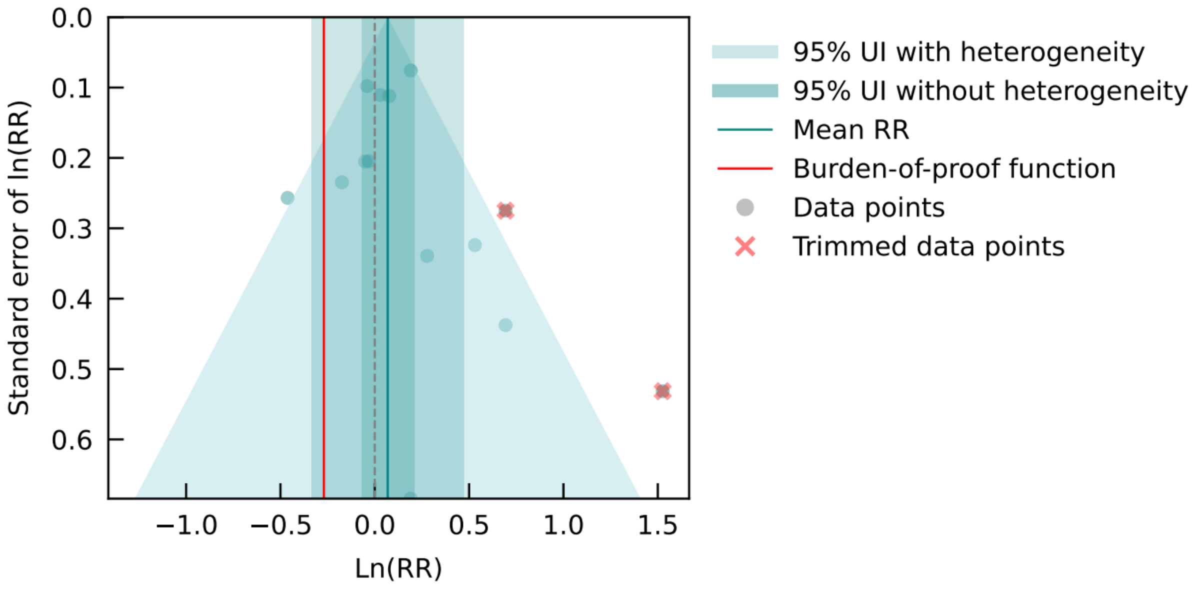

Figure S53. Primary analysis funnel plot for childhood neglect and drug use disorders

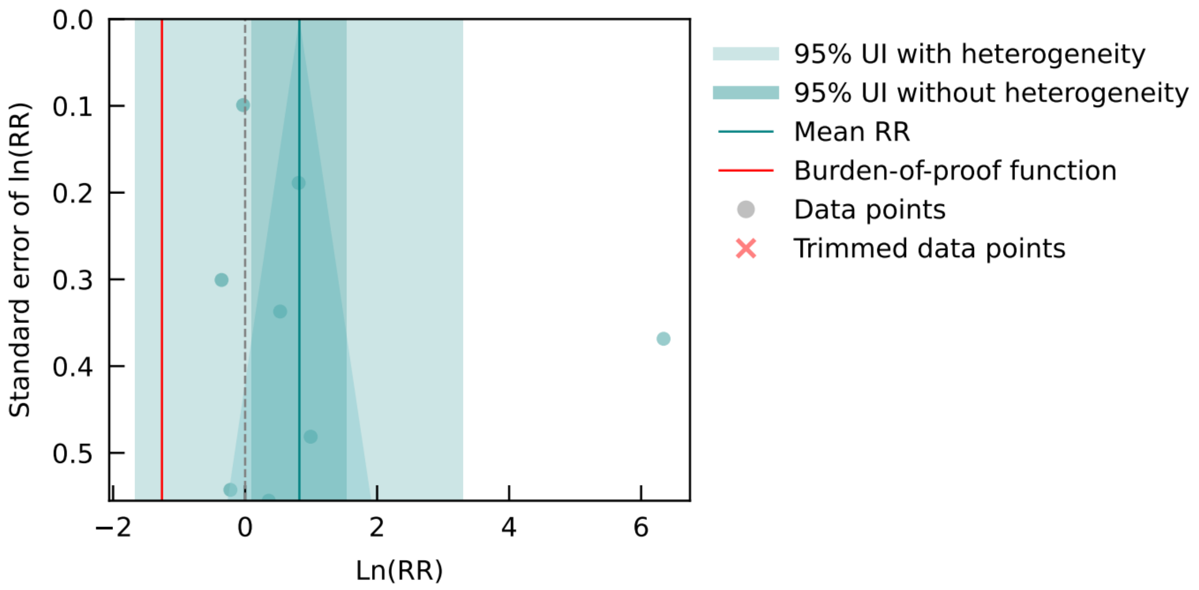

Figure S54. Primary analysis funnel plot for childhood neglect and schizophrenia

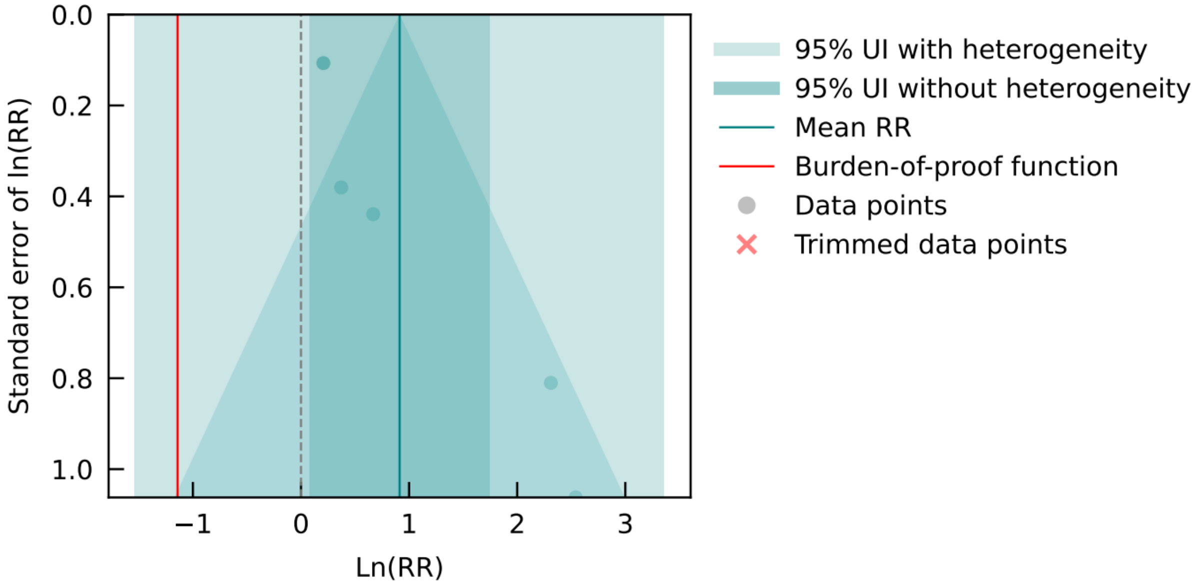

Figure S55. Primary analysis funnel plot for childhood neglect and self-harm

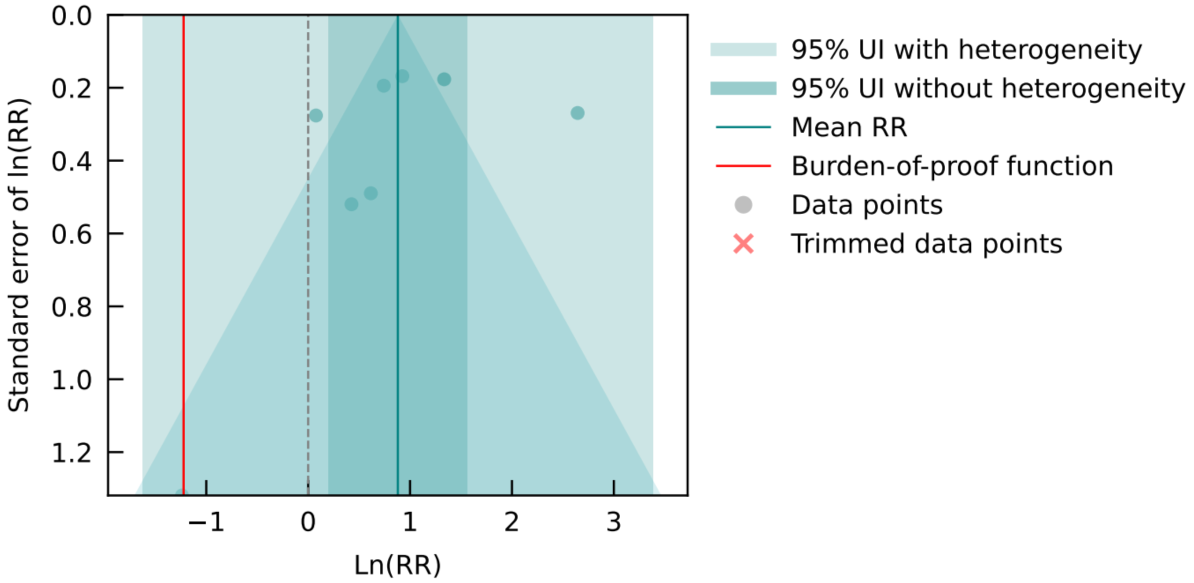

Figure S56. Primary analysis funnel plot for childhood neglect and sexually transmitted infections (excluding HIV)

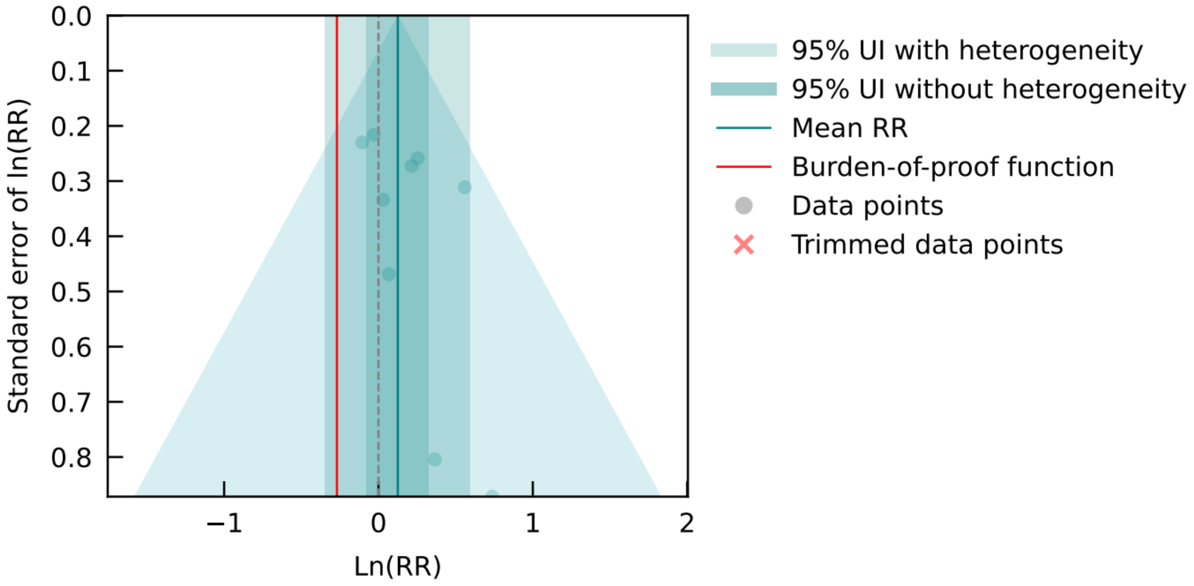

Section 10: Data for the Primary Analyses

In this section, we provide the data inputs used in the primary analyses following data cleaning and adjusting for overlapping observations.

Table S34. Data inputs included in the primary analyses

Study and Year	ln(RR)	Standard error of ln(RR)
**Neglect & Alcohol use disorders**		
Broekhof 2023	2.747271	0.486498
Laflair 2013	-0.10536	0.312188
Fenton 2013	0.336472	0.089695
Fenton 2013	0.04879	0.112695
Kascakova 2022	0	0.415851
Kascakova 2022	0.262364	0.288077
Kascakova 2022	0.336472	0.473396
Kascakova 2022	0.530628	0.396345
Kisely 2020	0.272315	0.36053
**Neglect & Anxiety disorders**		
Tenhave 2019	1.000632	0.109545
Widom 1999	0.593327	0.176823
Fujiwara 2011	-0.10536	0.326769
Young 2011	0.314811	0.495717
Raposo 2014	0.215111	0.116004
Danese 2023	0.076961	0.089019
Danese 2023	0.398776	0.097136
Danese 2023	0.29267	0.118995
Elbarazi 2023	0.262364	0.529009
Elbarazi 2023	-0.22314	0.500132
Hovens 2015	1.015231	0.357802
Hovens 2015	0.792993	0.436851
Kisely 2020	0.746688	0.598983
Kisely 2020	1.311032	0.831317
**Neglect & Asthma**		
Abajobir 2017	0.500775	0.260625
Kascakova 2022	1.568616	0.544363
Kascakova 2022	1.193922	0.47826
Kascakova 2022	0.470004	0.330569
Kascakova 2022	0.405465	0.300488
Han 2022	0.29267	0.07809
Han 2022	0.215111	0.043519
**Neglect & Major depressive disorder**		
Zhang 2023	0.086178	0.051192
Widom 2023	0.647103	0.202213
Widom 2007	0.463734	0.187051
Brown 1999	1.238374	0.649009
Comijs 2013	2.340844	0.36935
Young 2011	1.131402	0.838243
Kisely 2020	1.12493	0.440879

Lemasters 2021	-0.11653	0.193026
Ebert 2019	0.364643	0.528587
Xiao 2022	0.71295	0.246314
Xiao 2022	0.239017	0.292086
Danese 2023	0.113329	0.083909
Danese 2023	0.329304	0.073388
Danese 2023	0.165514	0.099014
Kang 2023	0.963174	0.99197
Kang 2023	0.708036	0.981576
Zhou 2023	1.601607	0.578294
Zhou 2023	0.705076	0.515368
Zhou 2023	1.7661	0.459741
Zhou 2023	0.608678	0.377532
Elbarazi 2023	1.252763	0.490998
Elbarazi 2023	-0.22314	0.476848
Hovens 2015	0.908259	0.361876
Hovens 2015	1.015231	0.312188
Galloeag 2017	0.405465	0.310305
Galloeag 2017	0.262364	0.489011
Houtepen 2020	0.131028	0.145289
**Neglect & Diabetes mellitus type 2**		
Widom 2023	0.19062	0.684203
Kascakova 2022	1.526056	0.531523
Kascakova 2022	0.693147	0.437621
Kascakova 2022	0.530628	0.323949
Kascakova 2022	0.693147	0.274956
Sanderson 2023	0.19062	0.075829
Sanderson 2023	0.076961	0.112083
Zhu 2023	0.029559	0.110508
Zhu 2023	-0.04082	0.097995
Thomas 2008	0.277632	0.339176
Duncan 2015	-0.05129	0.205092
Duncan 2015	-0.03046	0.20468
Duncan 2015	-0.46204	0.256838
Duncan 2015	-0.17435	0.234708
**Neglect & Drug use disorders**		
Harrington 2011	0.81093	0.188878
Schwartz 2024	0.530628	0.33707
Schwartz 2024	-0.22314	0.542624
Huang 2011	-0.03046	0.099147
Conroy 2009	-0.35667	0.300677
Conroy 2009	6.340359	0.368511
Abajobir 2017	0.357674	0.555146
Abajobir 2017	0.993252	0.481395
**Neglect & Schizophrenia**		
Mall 2020	0.207014	0.106814
Abajobir 2017	2.311545	0.810212
Chatziioannidis 2019	2.540814	1.062273
Alkema 2023	0.667829	0.439089
Alkema 2023	0.371564	0.380437

**Neglect & Self-harm**		
Kiselydmedres 2022	0.924259	0.168224
Geng 2023	2.644755	0.269276
Brown 1999	-1.23787	1.319001
Enns 2006	0.425268	0.519814
Thompson 2019	0.076961	0.276177
Bruffaerts 2010	0.741937	0.194423
Bruffaerts 2010	1.335001	0.176823
Rajapakse 2020	0.615186	0.489644
**Neglect & STIs excluding HIV**		
London 2017	-0.03046	0.216147
Wilson 2009	0.067659	0.468746
Wilson 2009	0.364643	0.804206
Wilson 2009	0.254642	0.258655
Wilson 2009	0.737164	0.871775
Haydon 2011	0.215111	0.272686
Haydon 2011	0.029559	0.333884
Haydon 2011	-0.10536	0.230097
Haydon 2011	0.559616	0.311063
**Physical abuse & Maternal abortion and miscarriage**		
Kerkar 2021	0.371564	0.211551
Kerkar 2021	0.470004	0.195979
Abajobir 2018	0.10436	0.409465
Abajobir 2018	-0.03046	0.696408
Demakakos 2020	2.10657	0.494177
Demakakos 2020	1.20896	0.459261
**Physical abuse & Alcohol use disorders**		
Fenton 2013	0.553885	0.065773
Roustit 2009	0.086178	0.379295
Kascakova 2022	0.641854	0.216147
Kascakova 2022	1.458615	0.344798
Najman 2022	0.463734	0.160945
Najman 2022	0.672944	0.197979
Telfar 2023	0.314811	0.46617
Telfar 2023	0.506818	0.581958
Telfar 2023	-0.47804	0.637585
Broekhof 2023	1.547563	0.434885
Broekhof 2023	0.09531	0.514006
Libby 2004	0.24686	0.367188
Libby 2004	0.636577	0.280423
Tanaka 2015	0	0.250212
Tanaka 2015	0.262364	0.334741
Tanaka 2015	-0.22314	0.319582
Tanaka 2015	0.336472	0.203701
**Physical abuse & Anxiety disorders**		
Tenhave 2019	0.828552	0.14381
Bhattarai 2023	0.19062	0.108589
Yu 2023	0.131028	0.026878
Elbarazi 2023	-0.22314	0.508273
Fergusson 2008	0.2	0.13

Widom 1999	0.71295	0.273072
Fujiwara 2011	0	0.250212
Raposo 2014	0.518794	0.142472
Danese 2023	-0.10536	0.273758
Danese 2023	0.371564	0.140764
Danese 2023	0.307485	0.076638
Hovens 2015	-0.26136	0.666191
Hovens 2015	0.139762	0.863159
Kisely 2021	0.270027	0.485239
Kisely 2021	1.381282	0.656052
**Physical abuse & Asthma**		
Njoroge 2023	0.837248	0.256733
Sun 2024	0.198851	0.083016
Abajobir 2017	0.270027	0.249813
Kascakova 2022	1.757858	0.410571
Kascakova 2022	0.641854	0.280258
Han 2022	0.364643	0.044246
Coogan 2013	0.113329	0.179279
Coogan 2013	0.231112	0.119457
**Physical abuse & Major depressive disorder**		
Xiang 2021	0.512824	0.060663
Xiao 2022	0.41211	0.175628
Su 2022	0.476234	0.102143
Merza 2015	0.576613	0.528437
Li 2024	0.270027	0.042616
Bhattarai 2023	0.536493	0.110724
Kang 2023	0.662688	0.693199
Yu 2023	0.300105	0.020721
Widom 2023	0.737164	0.31299
Elbarazi 2023	0.122218	0.029511
Fergusson 2008	0.44	0.11
Roustit 2009	0.29267	0.28355
Widom 2007	0.29267	0.313435
Andrews 1995	0.916291	0.596785
Brown 1999	1.342865	0.519613
Wise 2001	0.875469	0.130313
Mullen 1996	0.940007	0.355632
Comijs 2013	2.618125	0.734754
Kisely 2021	0.86289	0.487439
Ebert 2019	0.698135	0.509368
Danese 2023	0	0.274427
Danese 2023	0.314811	0.112547
Danese 2023	0.262364	0.062628
Zhou 2023	1.887524	0.780458
Zhou 2023	0.891178	0.628801
Chapman 2004	0.832909	0.076557
Chapman 2004	0.587787	0.115302
Hovens 2015	0.207014	0.478317
Hovens 2015	0.854415	0.597668
Galloeag 2017	-0.10536	0.280258

Galloeag 2017	0.262364	0.489011
Houtepen 2020	0.615186	0.138852
**Physical abuse & Diabetes mellitus type 2**		
Afifi 2013	0.019803	0.118715
Monnat 2015	0.075107	0.09735
Njoroge 2023	0.329304	0.233748
Gaston 2023	0.300105	0.078828
Widom 2023	0.385262	0.355412
Thomas 2008	0.019803	0.245615
Kascakova 2022	0.182322	0.53047
Kascakova 2022	1.064711	0.290672
Shields 2016	0.336472	0.18927
Shields 2016	0.09531	0.160359
Zhu 2023	0.10436	0.091263
Zhu 2023	0.04879	0.091568
Rich-Edwards 2010	0	0.066036
Rich-Edwards 2010	0.113329	0.054386
Rich-Edwards 2010	0.19062	0.073388
Duncan 2015	-0.27444	0.235865
Duncan 2015	-0.05129	0.184439
Duncan 2015	0.139762	0.212769
Duncan 2015	0.173953	0.24189
Lown 2019	0.239017	0.15834
Lown 2019	0.34359	0.196013
**Physical abuse & Drug use disorders**		
Scheidell 2018	0.039221	0.128416
Duncan 2008	-0.30111	0.201137
Cohen 2001	1.595339	0.605447
Huang 2011	0.336472	0.122781
Kisely 2021	0.722706	0.380199
Najman 2022	0.262364	0.179611
Najman 2022	0.512824	0.208144
Telfar 2023	0.307485	0.651546
Telfar 2023	0.173953	0.732688
Telfar 2023	0.329304	0.78649
Broekhof 2023	0.641854	0.328534
Broekhof 2023	1.308333	0.248821
Libby 2004	0.920283	0.394422
Libby 2004	0.727549	0.458937
Conroy 2009	0.470004	0.223334
Conroy 2009	0.09531	0.226353
Tanaka 2015	0.336472	0.334741
Tanaka 2015	0.530628	0.253381
Tanaka 2015	0.09531	0.254727
Tanaka 2015	0.405465	0.212477
**Physical abuse & Eating disorders**		
Rayworth 2004	0.693147	0.237642
Andrews 1995	1.931521	0.897068
Mullen 1996	1.018847	0.454834
Talmon 2021	-0.09431	0.686989

Talmon 2021	1.321756	1.54561
**Physical abuse & Gynecological diseases**		
Harris 2018	0.09531	0.043972
Liebermann 2018	0	0.036681
Ito 2021	0.636577	0.17109
Harlow 2005	0.09531	0.854567
Bertone-Johnson 2014	0.405465	0.152509
Boynton-Jarrett 2011	0.086178	0.025648
**Physical abuse & Ischemic heart disease**		
Monnat 2015	0.346423	0.132596
Dong 2004	0.405465	0.077903
Akasaki 2021	-0.27444	0.330288
Kascakova 2022	0	0.654324
Kascakova 2022	0.875469	0.396262
**Physical abuse & Migraine**		
Gelaye 2016	0.157004	0.099014
Karmakar 2017	0.258511	0.093862
Kascakova 2022	0.788457	0.400157
Kascakova 2022	0.587787	0.255944
Brennenstuhl 2015	0.405465	0.093021
Brennenstuhl 2015	0.476234	0.064709
**Physical abuse & Schizophrenia**		
Trotta 2023	-0.15082	0.126476
Alkema 2023	1.368639	0.583694
Mansueto 2022	0.500775	0.516825
Mansueto 2022	1.202972	0.55262
Chatziioannidis 2019	1.708378	0.940569
Chatziioannidis 2019	1.163151	0.788545
**Physical abuse & Self-harm**		
Kiselydmedres 2022	0.779325	0.21487
Salzinger 2007	1.913977	0.652239
Archambault 2023	1.671473	0.236022
Geng 2023	1.930942	0.310567
Calegaro 2023	0.392042	0.162242
Bhattarai 2023	0.336472	0.122781
Roustit 2009	0.329304	0.425435
Brown 1999	0.854415	0.688589
Mullen 1996	1.56653	0.511481
Enns 2006	1.118415	0.410943
Johnson 2002	1.629241	0.537537
Thompson 2019	0.647103	0.196638
Russell 2019	0.231112	0.138163
Bruffaerts 2010	0.916291	0.169637
Bruffaerts 2010	0.993252	0.18345
Fried 2013	0.322083	0.278123
Fried 2013	1.193922	0.433731
Rajapakse 2020	1.551809	0.710408
**Physical abuse & STIs excluding HIV**		
Widom 2012	1.83737	1.641842
Wilson 2009	0.198851	0.840586

Wilson 2009	1.091923	1.11851
Wilson 2009	0.576613	0.786615
London 2017	-0.46204	0.452128
**Physical abuse & Stroke**		
Campbell 2016	0.131028	0.20687
Njoroge 2023	0.357674	0.454183
Goodwin 2004	0.641854	0.821142
**Psychological abuse & Alcohol use disorders**		
Fenton 2013	0.482426	0.093478
Roustit 2009	0.559616	0.196416
Kascakova 2022	1.824549	0.335048
Kascakova 2022	1.098612	0.509718
Najman 2022	0.336472	0.140688
Najman 2022	-0.01005	0.196949
Broekhof 2023	1.871802	0.509718
Broekhof 2023	1.757858	0.755231
Kisely 2021	0.122218	0.376237
Laflair 2013	0.955511	0.680796
**Psychological abuse & Anxiety disorders**		
Tenhave 2019	0.891998	0.116257
Soenke 2010	2.488234	0.698817
Yu 2023	0.207014	0.026878
Elbarazi 2023	0.741937	0.384248
Raposo 2014	0.932164	0.170437
Hovens 2015	-0.4943	0.518602
Hovens 2015	-0.31471	0.665834
Kisely 2021	0.737164	0.503584
Kisely 2021	1.463255	0.691949
**Psychological abuse & Asthma**		
Njoroge 2023	0.604316	0.283477
Abajobir 2017	0.476234	0.235259
Kascakova 2022	1.88707	0.424271
Kascakova 2022	1.223775	0.262658
Han 2022	0.405465	0.040904
**Psychological abuse & Major depressive disorder**		
Xiao 2022	1.360977	0.178672
Su 2022	0.604316	0.09265
Kang 2023	0.924259	0.698679
Zhang 2023	0.364643	0.08105
Yu 2023	0.500775	0.021658
Elbarazi 2023	0.09531	0.423017
Roustit 2009	0.364643	0.169283
Mullen 1996	1.043804	0.311407
Comijs 2013	1.862529	0.394805
Kisely 2021	1.22083	0.488433
Ebert 2019	0.920283	0.320668
Zhou 2023	1.740115	0.543954
Zhou 2023	2.094946	0.763241
Chapman 2004	1.131402	0.096809
Chapman 2004	1.193922	0.154626

Vaeth 2010	1.054312	0.436779
Vaeth 2010	0.231112	0.361746
Hovens 2015	-0.46204	0.414189
Hovens 2015	-0.57982	0.557515
Galloeag 2017	0.993252	0.176823
Galloeag 2017	0.262364	0.392971
Houtepen 2020	0.350657	0.148874
**Psychological abuse & Diabetes mellitus type 2**		
Monnat 2015	0.043059	0.073529
Njoroge 2023	0.076961	0.258418
Gaston 2023	0.14842	0.046109
Kascakova 2022	1.481605	0.449895
Kascakova 2022	1.029619	0.290672
Zhu 2023	0.350657	0.098296
Zhu 2023	0.14842	0.096249
Thomas 2008	0.139762	0.320684
Thomas 2008	0.058269	0.321979
Duncan 2015	0	0.202173
Duncan 2015	0.512824	0.275837
Duncan 2015	-0.35667	0.231418
Duncan 2015	-0.82098	0.408007
**Psychological abuse & Drug use disorders**		
Scheidell 2018	0.378436	0.097702
Schwartz 2024	0.262364	0.269401
Broekhof 2023	1.223775	0.618572
Kisely 2021	0.631272	0.3719
Harrington 2011	0.751416	0.114209
Najman 2022	0.518794	0.149946
Najman 2022	0.371564	0.205405
Conroy 2009	0.182322	0.220663
Conroy 2009	-0.10536	0.216147
Scheidell 2018	0.029559	0.197802
**Psychological abuse & Gynecological diseases**		
Bertone-Johnson 2014	0.336472	0.139424
Liebermann 2018	0.182322	0.03666
Ito 2021	0.438255	0.125632
**Psychological abuse & Ischemic heart disease**		
Monnat 2015	-0.02327	0.116225
Kascakova 2022	1.589235	0.53968
Kascakova 2022	1.131402	0.403176
Dong 2004	0.530628	0.060303
Psychological abuse & Migraine		
Karmakar 2017	0.564177	0.065135
Kascakova 2022	1.360977	0.363523
Kascakova 2022	1.029619	0.248329
Brennenstuhl 2015	0.41871	0.136397
Brennenstuhl 2015	0.494696	0.083729
**Psychological abuse & Schizophrenia**		
Murphy 2020	1.085189	0.420953
Alkema 2023	1.050822	0.445758

Abajobir 2017	1.763017	0.824376
Chatziioannidis 2019	2.045109	0.655787
Trotta 2023	0.582216	0.231561
Trotta 2023	0.463734	0.319069
**Psychological abuse & Self-harm**		
Kiselydmedres 2022	0.996949	0.196815
Roustit 2009	0.307485	0.305087
Mullen 1996	2.494032	0.458543
Enns 2006	0.34359	0.46298
Thompson 2019	0.858662	0.176058
Russell 2019	0.277632	0.105808
Geng 2023	2.630809	0.27714
Rajapakse 2020	1.294727	0.516424
