# Supplementary material for: Health effects associated with exposure of children to physical violence, psychological violence and neglect: a Burden of Proof study
Source: Nat Hum Behav. 2025 Apr 10;9(6):1217–36. doi: 10.1038/s41562-025-02143-3 (PMC12185353; doi:10.1038/s41562-025-02143-3)
Supplement: Supplementary file 2 — Reporting Summary [file 41562_2025_2143_MOESM2_ESM.pdf]

nature portfolio

Corresponding author(s): Luisa S Flor, MSc, PhD

Last updated by author(s): Jan 22, 2025

Reporting Summary

Nature Portfolio wishes to improve the reproducibility of the work that we publish. This form provides structure for consistency and transparency in reporting. For further information on Nature Portfolio policies, see our Editorial Policies and the Editorial Policy Checklist.

Statistics

For all statistical analyses, confirm that the following items are present in the figure legend, table legend, main text, or Methods section.

n/a	Confirmed	
☐	☒	The exact sample size (*n*) for each experimental group/condition, given as a discrete number and unit of measurement
☐	☒	A statement on whether measurements were taken from distinct samples or whether the same sample was measured repeatedly
☐	☒	The statistical test(s) used AND whether they are one- or two-sided<br>*Only common tests should be described solely by name; describe more complex techniques in the Methods section.*
☐	☒	A description of all covariates tested
☐	☒	A description of any assumptions or corrections, such as tests of normality and adjustment for multiple comparisons
☐	☒	A full description of the statistical parameters including central tendency (e.g. means) or other basic estimates (e.g. regression coefficient) AND variation (e.g. standard deviation) or associated estimates of uncertainty (e.g. confidence intervals)
☐	☒	For null hypothesis testing, the test statistic (e.g. *F*, *t*, *r*) with confidence intervals, effect sizes, degrees of freedom and *P* value noted<br>*Give P values as exact values whenever suitable.*
☐	☒	For Bayesian analysis, information on the choice of priors and Markov chain Monte Carlo settings
☒	☐	For hierarchical and complex designs, identification of the appropriate level for tests and full reporting of outcomes
☐	☒	Estimates of effect sizes (e.g. Cohen's *d*, Pearson's *r*), indicating how they were calculated

Our web collection on statistics for biologists contains articles on many of the points above.

Software and code

Policy information about availability of computer code

Data collection	No primary data collection was carried out for this analysis.
Data analysis	All code used for these analyses is publicly available online (https://github.com/ihmeuw-msca/burden-of-proof/).

For manuscripts utilizing custom algorithms or software that are central to the research but not yet described in published literature, software must be made available to editors and reviewers. We strongly encourage code deposition in a community repository (e.g. GitHub). See the Nature Portfolio guidelines for submitting code & software for further information.

Data

Policy information about availability of data

All manuscripts must include a data availability statement. This statement should provide the following information, where applicable:

- Accession codes, unique identifiers, or web links for publicly available datasets
- A description of any restrictions on data availability
- For clinical datasets or third party data, please ensure that the statement adheres to our policy

The findings from this study are supported by data from the published literature. Study characteristics for all input data used in the analyses are provided in Supplementary Table 3. Data points included in each analysis are available in Supplementary Table 34. Input data and a lis of the included studies are also freely available at: https://ghdx.healthdata.org/record/ihme-data/health-effects-vac-bop-ros

Human research participants

Policy information about studies involving human research participants and Sex and Gender in Research.

Reporting on sex and gender	No primary data collection was carried out for this analysis, so the study does not involve human research participants. As stated in the methods overview, our estimates reflect both boys and girls, drawing upon all available data regardless of how or if the input study collected and reported data by sex or gender.
Population characteristics	No primary data collection was carried out for this analysis, so the study does not involve human research participants. The analysis evaluated the effect of childhood physical and psychological abuse and neglect (against both boys and girls) on selected disease endpoints. We accepted all studies regardless of the age of the study sample or target population.
Recruitment	No primary data collection was carried out for this analysis, so we did not recruit participants.
Ethics oversight	This study was approved by the University of Washington IRB Committee (study #9060).

Note that full information on the approval of the study protocol must also be provided in the manuscript.

Field-specific reporting

Please select the one below that is the best fit for your research. If you are not sure, read the appropriate sections before making your selection.

☒ Life sciences ☐ Behavioural & social sciences ☐ Ecological, evolutionary & environmental sciences

For a reference copy of the document with all sections, see nature.com/documents/nr-reporting-summary-flat.pdf

Life sciences study design

All studies must disclose on these points even when the disclosure is negative.

Sample size	The number of studies included was determined through a systematic literature review that included title/abstract screening, full-text screening, and citation searching to identify relevant articles and extract data points used as input to models. Details surrounding the sample size of each included study can be found in Supplementary Table 3 and the number of included studies per risk-outcome pair is reported in Table 2.
Data exclusions	As described in Supplementary Information Section 7.2, reports were excluded based on the following exclusion criteria:<br>Study design: Cross-sectional, ecological, case series or case studies.<br>Participants: Studies conducted in subgroups identified only by convenience sampling or subgroups identified via a shared characteristic that is likely related to risk of exposure to violence or the reported health outcome.<br>Exposure measurement: Studies that report only an aggregate measure of exposure combining exposure to a form of violence with other, non-eligible exposures (e.g., reports a composite ACE score only) were excluded. For these studies, we are unable to disentangle the effect of violence exposure from the effects of other hardships or exposure types, preventing their inclusion in our review.<br>Does not meet minimum reporting criteria: Studies missing essential data, that is, those that do not report effect sizes and uncertainty information (confidence intervals, sample sizes) or the data needed to impute an effect size with uncertainty information.
Replication	This is a meta-analysis of existing studies with many years of cohort and other data. The code and data used are publicly available, and the analyses could theoretically be replicated.
Randomization	This analysis is a meta-analysis of existing studies and thus, there were no experimental groups.
Blinding	N/A. This study was a meta-analyses using existing data and did not the collection of primary data nor experimental/control groups. As such, blinding was not relevant.

Reporting for specific materials, systems and methods

We require information from authors about some types of materials, experimental systems and methods used in many studies. Here, indicate whether each material, system or method listed is relevant to your study. If you are not sure if a list item applies to your research, read the appropriate section before selecting a response.

Materials & experimental systems

n/a	Involved in the study
☒	☐ Antibodies
☒	☐ Eukaryotic cell lines
☒	☐ Palaeontology and archaeology
☒	☐ Animals and other organisms
☒	☐ Clinical data
☒	☐ Dual use research of concern

Methods

n/a	Involved in the study
☒	☐ ChIP-seq
☒	☐ Flow cytometry
☒	☐ MRI-based neuroimaging
